# Supplementary material for: Transcriptomic Analysis Reveals Insect Hormone Biosynthesis Pathway Involved in Desynchronized Development Phenomenon in Hybridized Sibling Species of Tea Geometrids (Ectropis grisescens and Ectropis obliqua)
Source: Insects. 2019 Nov 1;10(11):381. doi: 10.3390/insects10110381 (PMC6920886; doi:10.3390/insects10110381)
Supplement: Supplementary file 1 [file insects-10-00381-s001.pdf]

insects

MDPI

Article

Transcriptomic Analysis Reveals Insect Hormone Biosynthesis Pathway Involved in Desynchronized Development Phenomenon in Hybridized Sibling Species of Tea Geometrids (*Ectropis grisescens* and *Ectropis obliqua)*

Zhibo Wang [1,†], Jiahe Bai [1,†], Yongjian Liu [2], Hong Li [1], Shuai Zhan [2] and Qiang Xiao [1,*]

1 Key Laboratory of Tea Quality and Safety Control, Tea Research Institute, Ministry of Agriculture, Chinese Academy of Agricultural Sciences, Hangzhou 310008, China; wangzhibo9527@163.com (Z.W.); baijhtricaas@163.com (J.B.); hongli_tea@163.com (H.L.)
2 Key Laboratory of Insect Developmental and Evolutionary Biology, Institute of Plant Physiology and Ecology, Shanghai Institutes for Biological Sciences, Chinese Academy of Sciences, Shanghai 200032, China; liuyongjian@sippe.ac.cn (Y.L.); szhan@sibs.ac.cn (S.Z.)
* Correspondence: xqtea@mail.tricaas.com; Tel.: +86-0571-86650801
† These authors contributed equally to this work.

Received: 19 September 2019; Accepted: 24 October 2019; Published: date

Supplementary Materials

Table S1. Collecting information of samples in China.

Populations	Code	Locality	Longitude(°E)/Latltude (°N)	Date (M/Y)
Wuxi	WX	Wuxi/Jiangsu	120.31/31.49	3/2017
Huzhou	HZ	Huzhou/Zhejiang	119.68/30.64	10/2017
Yuhang	YH	Yuhang/Zhejiang	120.27/30.42	10/2017
Liyang	LY	Liyang/Jiangsu	119.39/31.33	2016–2017
Shizizhen	SZZ	Shizizhen/Anhui	119.09/30.59	2016–2017
Tea Research Institute	TRI	Tea Research Institute/Zhejiang	120.08/30.20	6/2017
Lin'an	LA	Lin'an/Zhejiang	119.37/30.16	5/2017
Fuyang	FY	Fuyang/Zhejiang	119.73/29.97	10/2017
Chun'an	CA	Chun'an/Zhejiang	118.67/29.39	10/2017
Yizheng	YZ	Yizheng/Jiangsu	119.40/32.39	3/2017
Jurong	JR	Jurong/Jiangsu	119.16/31.94	8/2017
Nanchang	NC	Nanchang/Jiangxi	115.95/28.52	9/2016
Wuyishan	WYS	Wuyishan/Fujian	118.06/27.98	6/2012
Yingde	YD	Yingde/Guangdong	113.41/24.19	9/2015
Changsha	CS	Changsha/Hunan	112.93/28.23	5/2011
Enshi	ES	Enshi/Hubei	109.48/30.27	8/2016
Xinyang	XY	Xinyang/Henan	114.08/32.15	6/2012
Guilin	GL	Guilin/Guangxi	110.16/25.17	10/2017
Tongren	TR	Tongren/Guizhou	109.18/27.69	6/2017

insects

MDPI

Table S2. Identified numbers and Accession number.

Populations	Code	Identified Numbers	*No. of E. grisescens and E. obliqua*	Accession Number
Wuxi	WX	44	*E. obliqua* (44)	MH286129, MH286130
Huzhou	HZ	26	*E. obliqua* (26)	MH286151
Yuhang	YH	38	*E. obliqua* (38)	MH286152
Liyang	LY	30	*E. obliqua* (27); *E. grisescens* (3)	MH286133, MH286134
Shizizhen	SZZ	184	*E. obliqua* (148); *E. grisescens* (36)	MH271323, MH286127, MH286128
Tea Research Institute	TRI	47	*E. obliqua* (42); *E. grisescens* (5)	MH286149, MH286150
Lin'an	LA	26	*E. obliqua* (11); *E. grisescens* (15)	MH286147, MH286148
Fuyang	FY	28	*E. obliqua* (6); *E. grisescens* (22)	MH286145, MH286146
Chun'an	CA	32	*E. grisescens* (32)	MH286153
Yizheng	YZ	68	*E. grisescens* (68)	MH286131, MH286132
Jurong	JR	32	*E. grisescens* (32)	MH286135, MH286136
Nanchang	NC	19	*E. grisescens* (19)	MH286139
Wuyishan	WYS	25	*E. grisescens* (25)	MH286137
Yingde	YD	10	*E. grisescens* (10)	MH286143
Changsha	CS	23	*E. grisescens* (23)	MH286144
Enshi	ES	34	*E. grisescens* (34)	MH286140
Xinyang	XY	21	*E. grisescens* (21)	MH286138
Guilin	GL	28	*E. grisescens* (28)	MH286142
Tongren	TR	42	*E. grisescens* (42)	MH286141

Note: The numbers in ( ) represent the number of *E. grisescens and E. obliqua.*

insects

MDPI

Table S3. Gene Ontology terms enriched in C-vs-L group.

ID	Description	GeneRatio	BgRatio	*p* Value	*p* Adjust	*q* Value	geneID	Count
GO:0031514	motile cilium	49/1853	156/22442	7.02×10^{-17}	2.11×10^{-13}	1.99×10^{-13}	TRINITY_DN6620_c0_g1/TRINITY_DN5141_c0_g1/TRINITY_DN8072_c0_g1/TRINITY_DN13193_c0_g1/TRINITY_DN7072_c0_g1/TRINITY_DN1579_c0_g1/TRINITY_DN3895_c0_g2/TRINITY_DN10548_c0_g1/TRINITY_DN7997_c0_g1/TRINITY_DN8542_c0_g1/TRINITY_DN4420_c0_g1/TRINITY_DN41571_c0_g2/TRINITY_DN4119_c0_g1/TRINITY_DN8480_c0_g1/TRINITY_DN8467_c0_g1/TRINITY_DN6225_c1_g1/TRINITY_DN33652_c0_g1/TRINITY_DN9691_c0_g1/TRINITY_DN13308_c0_g1/TRINITY_DN5409_c0_g1/TRINITY_DN11109_c0_g1/TRINITY_DN12895_c0_g1/TRINITY_DN12881_c0_g1/TRINITY_DN12379_c0_g1/TRINITY_DN8728_c0_g1/TRINITY_DN67550_c0_g1/TRINITY_DN14529_c0_g1/TRINITY_DN19684_c0_g1/TRINITY_DN16772_c0_g1/TRINITY_DN14367_c0_g1/TRINITY_DN6439_c0_g2/TRINITY_DN8012_c1_g1/TRINITY_DN15340_c0_g2/TRINI	49

GO:0004252	serine-type endopeptidase activity	84/1853	396/22442	5.34 × 10^{-16}	8.03 × 10^{-13}	7.56 × 10^{-13}	TY_DN23273_c0_g1/TRINITY_DN16994_c0_g1/TRINITY_DN5804_c0_g1/TRINITY_DN21369_c0_g1/TRINITY_DN8676_c0_g1/TRINITY_DN1105_c0_g1/TRINITY_DN28402_c0_g1/TRINITY_DN5776_c0_g1/TRINITY_DN26604_c0_g1/TRINITY_DN15362_c0_g2/TRINITY_DN33652_c1_g1/TRINITY_DN18814_c0_g1/TRINITY_DN22894_c0_g1/TRINITY_DN12487_c0_g1/TRINITY_DN10924_c0_g1/TRINITY_DN11321_c0_g1 TRINITY_DN3813_c0_g1/TRINITY_DN1405_c0_g2/TRINITY_DN5337_c1_g1/TRINITY_DN9772_c1_g1/TRINITY_DN3589_c0_g1/TRINITY_DN2935_c0_g1/TRINITY_DN5796_c0_g1/TRINITY_DN8858_c0_g1/TRINITY_DN1551_c0_g1/TRINITY_DN9120_c0_g2/TRINITY_DN872_c0_g1/TRINITY_DN647_c0_g1/TRINITY_DN10815_c1_g1/TRINITY_DN6769_c0_g1/TRINITY_DN418_c0_g1/TRINITY_DN5930_c0_g1/TRINITY_DN1217_c0_g1/TRINITY_DN8315_c0_g1/TRINITY_DN3542_c0_g1/TRINITY_DN20580_c0_g1/TRINITY_DN3480_c0_g1/T	84

insects

RINITY_DN418_c0_g3/TRINITY_DN418_c0_g2/TRINITY_DN30633_c0_g1/TRINITY_DN475_c0_g1/TRINITY_DN41578_c1_g1/TRINITY_DN1255_c1_g1/TRINITY_DN20566_c0_g1/TRINITY_DN500_c1_g2/TRINITY_DN9607_c1_g1/TRINITY_DN1255_c0_g1/TRINITY_DN872_c0_g2/TRINITY_DN2094_c0_g1/TRINITY_DN44735_c0_g1/TRINITY_DN59849_c0_g1/TRINITY_DN3486_c0_g1/TRINITY_DN14923_c0_g1/TRINITY_DN4466_c0_g1/TRINITY_DN425_c0_g1/TRINITY_DN20920_c0_g1/TRINITY_DN20632_c0_g1/TRINITY_DN45024_c0_g1/TRINITY_DN2406_c0_g1/TRINITY_DN20632_c0_g2/TRINITY_DN23730_c0_g1/TRINITY_DN1367_c0_g1/TRINITY_DN21632_c0_g2/TRINITY_DN18386_c1_g1/TRINITY_DN461_c0_g2/TRINITY_DN7230_c0_g1/TRINITY_DN3554_c0_g1/TRINITY_DN7230_c0_g2/TRINITY_DN25822_c0_g1/TRINITY_DN551_c1_g1/TRINITY_DN850_c0_g1/TRINITY_DN18300_c0_g1/TRINITY_DN1446_c0_g1/TRINITY_DN5369_c0_g3/TRI

							NITY_DN43117_c0_g2/TRINITY_DN2168_c0_g1/TRINITY_DN5331_c0_g1/TRINITY_DN3333_c0_g1/TRINITY_DN55174_c0_g1/TRINITY_DN326_c1_g1/TRINITY_DN1709_c0_g2/TRINITY_DN418_c3_g1/TRINITY_DN51634_c0_g2/TRINITY_DN27965_c0_g1/TRINITY_DN34089_c0_g1/TRINITY_DN21874_c0_g1/TRINITY_DN22_c2_g1/TRINITY_DN418_c0_g4/TRINITY_DN14404_c0_g1/TRINITY_DN12345_c0_g1/TRINITY_DN15542_c0_g1/TRINITY_DN7353_c0_g1/TRINITY_DN3523_c0_g1/TRINITY_DN12351_c0_g1/TRINITY_DN1801_c0_g1/TRINITY_DN14404_c1_g1/TRINITY_DN75_c0_g1/TRINITY_DN1405_c0_g3/TRINITY_DN54378_c0_g1/TRINITY_DN75_c0_g2	
GO:0005549	odorant binding	24/1853	54/22442	1.07×10^{-12}	1.07×10^{-9}	1.01×10^{-9}	TRINITY_DN2704_c0_g1/TRINITY_DN1792_c0_g1/TRINITY_DN1_c7_g1/TRINITY_DN1532_c0_g1/TRINITY_DN10105_c1_g1/TRINITY_DN8502_c0_g1/TRINITY_DN5279_c0_g1/TRINITY_DN8423_c0_g1/TRINITY_DN2119_c0_g1/TRINITY_DN13558_c0_g1/TRINITY_DN	24

							18645_c0_g1/TRINITY_DN10865_c0_g1/TRINITY_DN58768_c0_g3/TRINITY_DN13068_c0_g1/TRINITY_DN19984_c0_g1/TRINITY_DN7245_c0_g1/TRINITY_DN25958_c0_g1/TRINITY_DN22639_c0_g1/TRINITY_DN28118_c0_g2/TRINITY_DN6318_c0_g1/TRINITY_DN25804_c0_g1/TRINITY_DN17621_c0_g2/TRINITY_DN2473_c0_g2/TRINITY_DN69114_c0_g1	
GO:0036126	sperm flagellum	19/1853	36/22442	5.26×10^{-12}	3.95×10^{-9}	3.72×10^{-9}	TRINITY_DN2720_c1_g1/TRINITY_DN16194_c0_g1/TRINITY_DN5141_c0_g1/TRINITY_DN3290_c0_g1/TRINITY_DN7072_c0_g1/TRINITY_DN12837_c0_g1/TRINITY_DN4647_c0_g1/TRINITY_DN10099_c0_g1/TRINITY_DN2468_c0_g1/TRINITY_DN8480_c0_g1/TRINITY_DN8467_c0_g1/TRINITY_DN6225_c1_g1/TRINITY_DN6785_c0_g1/TRINITY_DN12881_c0_g1/TRINITY_DN13060_c0_g1/TRINITY_DN6439_c1_g2/TRINITY_DN28846_c0_g1/TRINITY_DN21369_c0_g1/TRINITY_DN10924_c0_g1	19
GO:0007288	sperm axoneme assembly	12/1853	19/22442	2.82×10^{-9}	1.70×10^{-6}	1.60×10^{-6}	TRINITY_DN12837_c0_g1/TRINITY_DN8480_c0_g1/TRINITY_DN19594_c0_g1/TRINITY_	12

							DN6225_c1_g1/TRINITY_DN8472_c0_g1/TRINITY_DN4107_c0_g1/TRINITY_DN12881_c0_g1/TRINITY_DN8728_c0_g1/TRINITY_DN19161_c0_g1/TRINITY_DN6439_c1_g2/TRINITY_DN15362_c0_g2/TRINITY_DN10924_c0_g1	
GO:0004177	aminopeptidase activity	20/1853	54/22442	4.06×10^{-9}	2.04×10^{-6}	1.92×10^{-6}	TRINITY_DN647_c0_g1/TRINITY_DN1217_c0_g1/TRINITY_DN15191_c0_g1/TRINITY_DN8267_c0_g1/TRINITY_DN18930_c0_g1/TRINITY_DN703_c1_g3/TRINITY_DN703_c1_g1/TRINITY_DN2723_c0_g1/TRINITY_DN5123_c0_g1/TRINITY_DN47563_c0_g1/TRINITY_DN13855_c0_g1/TRINITY_DN3835_c1_g1/TRINITY_DN1346_c0_g1/TRINITY_DN14581_c0_g1/TRINITY_DN1234_c0_g1/TRINITY_DN864_c0_g1/TRINITY_DN21874_c0_g1/TRINITY_DN3861_c0_g1/TRINITY_DN6125_c0_g1/TRINITY_DN1107_c0_g1	20
GO:0003351	epithelial cilium movement	12/1853	20/22442	6.52×10^{-9}	2.80×10^{-6}	2.64×10^{-6}	TRINITY_DN8072_c0_g1/TRINITY_DN7072_c0_g1/TRINITY_DN12837_c0_g1/TRINITY_DN19594_c0_g1/TRINITY_DN8472_c0_g1/TRINITY_DN6785_c0_g1/TRINITY_DN67550_c0	12

							_g1/TRINITY_DN6439_c1_g2/TRINITY_DN2566_c4_g1/TRINITY_DN21369_c0_g1/TRINITY_DN43314_c0_g2/TRINITY_DN15433_c0_g1	
GO:0036156	inner dynein arm	10/1853	14/22442	1.06 × 10^{-8}	3.97 × 10^{-6}	3.74 × 10^{-6}	TRINITY_DN12837_c0_g1/TRINITY_DN59265_c1_g1/TRINITY_DN12688_c0_g1/TRINITY_DN6439_c1_g2/TRINITY_DN6439_c0_g2/TRINITY_DN17632_c0_g1/TRINITY_DN1105_c0_g1/TRINITY_DN28402_c0_g1/TRINITY_DN6439_c0_g3/TRINITY_DN6439_c1_g1	10
GO:0005930	axoneme	35/1853	157/22442	4.75 × 10^{-8}	1.59 × 10^{-5}	1.50 × 10^{-5}	TRINITY_DN6235_c0_g1/TRINITY_DN16194_c0_g1/TRINITY_DN7072_c0_g1/TRINITY_DN2925_c0_g1/TRINITY_DN12837_c0_g1/TRINITY_DN7997_c0_g1/TRINITY_DN4420_c0_g1/TRINITY_DN16707_c0_g1/TRINITY_DN41571_c0_g2/TRINITY_DN8467_c0_g1/TRINITY_DN9691_c0_g1/TRINITY_DN13308_c0_g1/TRINITY_DN12895_c0_g1/TRINITY_DN12379_c0_g1/TRINITY_DN8728_c0_g1/TRINITY_DN13060_c0_g1/TRINITY_DN14529_c0_g1/TRINITY_DN19684_c0_g1/TRINITY_DN59265_c1_g1/TRINITY_DN6439_c1_g2/TRINITY_DN	35

							28846_c0_g1/TRINITY_DN12493_c0_g1/TRINITY_DN15340_c0_g2/TRINITY_DN21369_c0_g1/TRINITY_DN32303_c0_g1/TRINITY_DN19322_c0_g1/TRINITY_DN28402_c0_g1/TRINITY_DN5776_c0_g1/TRINITY_DN15362_c0_g2/TRINITY_DN11245_c0_g1/TRINITY_DN12487_c0_g1/TRINITY_DN10924_c0_g1/TRINITY_DN21874_c0_g1/TRINITY_DN3830_c0_g1/TRINITY_DN11321_c0_g1	
GO:0036159	inner dynein arm assembly	14/1853	32/22442	7.39×10^{-8}	2.22×10^{-5}	2.09×10^{-5}	TRINITY_DN6235_c0_g1/TRINITY_DN3290_c0_g1/TRINITY_DN12837_c0_g1/TRINITY_DN7221_c0_g2/TRINITY_DN59265_c1_g1/TRINITY_DN12688_c0_g1/TRINITY_DN6439_c1_g2/TRINITY_DN17632_c0_g1/TRINITY_DN2566_c4_g1/TRINITY_DN28402_c0_g1/TRINITY_DN6439_c0_g3/TRINITY_DN6439_c1_g1/TRINITY_DN5659_c0_g1/TRINITY_DN3830_c0_g1	14
GO:0042073	intraciliary transport	11/1853	20/22442	9.84×10^{-8}	2.47×10^{-5}	2.33×10^{-5}	TRINITY_DN16707_c0_g1/TRINITY_DN4955_c0_g1/TRINITY_DN13058_c0_g1/TRINITY_DN8676_c0_g1/TRINITY_DN15433_c0_g1/TRINITY_DN15601_c0_g1/TRINITY_DN11245_c	11

							0_g1/TRINITY_DN12487_c0_g1/TRINITY_DN28353_c0_g1/TRINITY_DN24749_c0_g1/TRINITY_DN10229_c0_g2	
GO:0008234	cysteine-type peptidase activity	19/1853	58/22442	9.87×10^{-8}	2.47×10^{-5}	2.33×10^{-5}	TRINITY_DN647_c0_g1/TRINITY_DN1217_c0_g1/TRINITY_DN10957_c0_g1/TRINITY_DN16716_c0_g1/TRINITY_DN703_c1_g3/TRINITY_DN703_c1_g1/TRINITY_DN4321_c0_g1/TRINITY_DN2564_c0_g3/TRINITY_DN13855_c0_g1/TRINITY_DN8634_c0_g2/TRINITY_DN8634_c0_g1/TRINITY_DN430_c0_g1/TRINITY_DN26084_c0_g1/TRINITY_DN2564_c0_g2/TRINITY_DN14581_c0_g1/TRINITY_DN9223_c0_g1/TRINITY_DN1587_c0_g1/TRINITY_DN21874_c0_g1/TRINITY_DN6608_c0_g3	19
GO:0060285	cilium-dependent cell motility	18/1853	54/22442	1.58×10^{-7}	3.50×10^{-5}	3.30×10^{-5}	TRINITY_DN6620_c0_g1/TRINITY_DN13077_c0_g1/TRINITY_DN7997_c0_g1/TRINITY_DN4420_c0_g1/TRINITY_DN41571_c0_g2/TRINITY_DN12379_c0_g1/TRINITY_DN14529_c0_g1/TRINITY_DN11326_c0_g1/TRINITY_DN12688_c0_g1/TRINITY_DN17632_c0_g1/TRINITY_DN15340_c0_g2/TRINITY_DN6620_c0_g2/TRINITY_	18

insects MDPI

							DN32303_c0_g1/TRINITY_DN6439_c1_g1/TRINITY_DN15362_c0_g2/TRINITY_DN47966_c0_g1/TRINITY_DN3830_c0_g1/TRINITY_DN15707_c0_g1	
GO:0030992	intraciliary transport particle B	10/1853	17/22442	1.63×10^{-7}	3.50×10^{-5}	3.30×10^{-5}	TRINITY_DN11712_c0_g1/TRINITY_DN13058_c0_g1/TRINITY_DN8676_c0_g1/TRINITY_DN25714_c0_g1/TRINITY_DN15433_c0_g1/TRINITY_DN15601_c0_g1/TRINITY_DN11245_c0_g1/TRINITY_DN12487_c0_g1/TRINITY_DN10924_c0_g1/TRINITY_DN24749_c0_g1	10
GO:0004197	cysteine-type endopeptidase activity	22/1853	78/22442	2.00×10^{-7}	4.01×10^{-5}	3.78×10^{-5}	TRINITY_DN4347_c0_g1/TRINITY_DN647_c0_g1/TRINITY_DN1217_c0_g1/TRINITY_DN10957_c0_g1/TRINITY_DN16716_c0_g1/TRINITY_DN703_c1_g3/TRINITY_DN703_c1_g1/TRINITY_DN4321_c0_g1/TRINITY_DN650_c0_g1/TRINITY_DN13855_c0_g1/TRINITY_DN8634_c0_g2/TRINITY_DN8634_c0_g1/TRINITY_DN8465_c0_g1/TRINITY_DN430_c0_g1/TRINITY_DN14581_c0_g1/TRINITY_DN4935_c0_g1/TRINITY_DN5697_c0_g1/TRINITY_DN3997_c0_g1/TRINITY_DN1587_c0_g1/TRINITY_DN9554_c0_g	22

							1/TRINITY_DN21874_c0_g1/TRINITY_DN6608_c0_g3	
GO:0060294	cilium movement involved in cell motility	10/1853	18/22442	3.39×10^{-7}	6.37×10^{-5}	6.00×10^{-5}	TRINITY_DN2720_c1_g1/TRINITY_DN3290_c0_g1/TRINITY_DN7072_c0_g1/TRINITY_DN12837_c0_g1/TRINITY_DN4647_c0_g1/TRINITY_DN2468_c0_g1/TRINITY_DN12895_c0_g1/TRINITY_DN6439_c1_g2/TRINITY_DN21369_c0_g1/TRINITY_DN28402_c0_g1	10
GO:0042302	structural constituent of cuticle	37/1853	191/22442	8.56×10^{-7}	0.000151	0.000143	TRINITY_DN1000_c0_g1/TRINITY_DN3123_c0_g1/TRINITY_DN1389_c0_g1/TRINITY_DN3277_c0_g1/TRINITY_DN4192_c0_g1/TRINITY_DN3218_c0_g1/TRINITY_DN6515_c0_g1/TRINITY_DN4040_c0_g2/TRINITY_DN3156_c1_g1/TRINITY_DN1263_c0_g1/TRINITY_DN505_c0_g1/TRINITY_DN23502_c0_g2/TRINITY_DN8020_c0_g1/TRINITY_DN49735_c0_g1/TRINITY_DN7657_c0_g1/TRINITY_DN5899_c0_g1/TRINITY_DN7142_c1_g1/TRINITY_DN6985_c0_g1/TRINITY_DN13464_c0_g2/TRINITY_DN6233_c0_g1/TRINITY_DN41872_c0_g1/TRINITY_DN3161_c0_g3/TRINITY_DN776_c0_g1/TRINITY_DN7253_c0_g2/TRINITY_	37

GO:0031090	organelle membrane	49/1853	288/22442	9.55×10^{-7}	0.00016	0.00015	DN12320_c1_g1/TRINITY_DN21849_c0_g1/TRINITY_DN8077_c0_g1/TRINITY_DN639_c0_g1/TRINITY_DN22449_c0_g1/TRINITY_DN8498_c0_g1/TRINITY_DN48108_c0_g1/TRINITY_DN3150_c0_g1/TRINITY_DN2999_c1_g1/TRINITY_DN10645_c0_g1/TRINITY_DN5336_c0_g1/TRINITY_DN35289_c0_g1/TRINITY_DN617_c0_g1TRINITY_DN11877_c0_g2/TRINITY_DN2352_c0_g2/TRINITY_DN5701_c0_g1/TRINITY_DN2531_c2_g1/TRINITY_DN53219_c0_g1/TRINITY_DN2754_c0_g1/TRINITY_DN5701_c0_g2/TRINITY_DN16034_c0_g1/TRINITY_DN2955_c0_g2/TRINITY_DN8292_c0_g1/TRINITY_DN36608_c0_g1/TRINITY_DN54392_c0_g2/TRINITY_DN461_c0_g1/TRINITY_DN1949_c0_g1/TRINITY_DN5766_c0_g2/TRINITY_DN42315_c0_g1/TRINITY_DN2871_c0_g2/TRINITY_DN482_c0_g1/TRINITY_DN35617_c0_g1/TRINITY_DN16074_c0_g1/TRINITY_DN6715_c0_g1/TRINITY_DN18271_c1_g2/TRINITY_DN19412_c0_g1/TRINITY_DN13080_c1_g2/TR	49

							INITY_DN1029_c0_g1/TRINITY_DN4749_c0_g3/TRINITY_DN30098_c0_g2/TRINITY_DN18210_c0_g1/TRINITY_DN15993_c0_g1/TRINITY_DN30772_c0_g1/TRINITY_DN2041_c0_g3/TRINITY_DN65320_c0_g1/TRINITY_DN4798_c0_g1/TRINITY_DN21883_c1_g1/TRINITY_DN1316_c6_g1/TRINITY_DN1812_c0_g1/TRINITY_DN1062_c0_g1/TRINITY_DN13796_c0_g3/TRINITY_DN428_c3_g1/TRINITY_DN32222_c0_g1/TRINITY_DN892_c0_g2/TRINITY_DN13080_c1_g3/TRINITY_DN1812_c0_g3/TRINITY_DN11902_c0_g1/TRINITY_DN8820_c0_g1/TRINITY_DN2352_c0_g1/TRINITY_DN9739_c0_g1/TRINITY_DN2801_c0_g1/TRINITY_DN22316_c0_g2	
GO:0006811	ion transport	40/1853	217/22442	1.19×10^{-6}	0.000189	0.000178	TRINITY_DN4901_c3_g1/TRINITY_DN35198_c0_g1/TRINITY_DN2080_c0_g1/TRINITY_DN10272_c0_g1/TRINITY_DN8811_c0_g1/TRINITY_DN8542_c0_g1/TRINITY_DN19996_c0_g1/TRINITY_DN10962_c0_g1/TRINITY_DN28824_c0_g1/TRINITY_DN13967_c0_g1/TRINITY_DN10884_c0_g1/TRINITY	40

insects MDPI

							_DN17770_c0_g1/TRINITY_DN33878_c0_g1/TRINITY_DN9224_c0_g1/TRINITY_DN45783_c0_g1/TRINITY_DN23684_c0_g1/TRINITY_DN10534_c0_g1/TRINITY_DN868_c0_g1/TRINITY_DN8089_c0_g1/TRINITY_DN12399_c0_g1/TRINITY_DN8087_c0_g1/TRINITY_DN20304_c0_g1/TRINITY_DN11221_c0_g1/TRINITY_DN8390_c0_g1/TRINITY_DN1764_c0_g1/TRINITY_DN2668_c0_g1/TRINITY_DN1074_c0_g2/TRINITY_DN15560_c0_g1/TRINITY_DN11348_c0_g1/TRINITY_DN40621_c0_g1/TRINITY_DN2003_c0_g2/TRINITY_DN12312_c0_g2/TRINITY_DN34051_c0_g1/TRINITY_DN6216_c0_g1/TRINITY_DN10030_c0_g1/TRINITY_DN2559_c1_g1/TRINITY_DN1576_c2_g1/TRINITY_DN473_c0_g1/TRINITY_DN9651_c0_g1/TRINITY_DN8819_c1_g1	
GO:0016705	oxidoreductase activity, with incorporation or reduction of molecular oxygen	40/1853	218/22442	1.34×10^{-6}	0.000202	0.00019	TRINITY_DN2352_c0_g2/TRINITY_DN2531_c2_g1/TRINITY_DN2754_c0_g1/TRINITY_DN2955_c0_g2/TRINITY_DN12668_c0_g1/TRINITY_DN10997_c0_g1/TRINITY_DN36608_c0_g1/TRINITY_DN54392_c0_g2	40

insects MDPI

							/TRINITY_DN461_c0_g1/TRINITY_DN1949_c0_g1/TRINITY_DN14942_c0_g2/TRINITY_DN29854_c0_g1/TRINITY_DN42315_c0_g1/TRINITY_DN2871_c0_g2/TRINITY_DN482_c0_g1/TRINITY_DN35617_c0_g1/TRINITY_DN16074_c0_g1/TRINITY_DN6715_c0_g1/TRINITY_DN18271_c1_g2/TRINITY_DN19412_c0_g1/TRINITY_DN13080_c1_g2/TRINITY_DN1029_c0_g1/TRINITY_DN4749_c0_g3/TRINITY_DN30098_c0_g2/TRINITY_DN18210_c0_g1/TRINITY_DN12260_c0_g1/TRINITY_DN30772_c0_g1/TRINITY_DN2041_c0_g3/TRINITY_DN21883_c1_g1/TRINITY_DN1316_c6_g1/TRINITY_DN1062_c0_g1/TRINITY_DN13796_c0_g3/TRINITY_DN1609_c0_g1/TRINITY_DN32222_c0_g1/TRINITY_DN13080_c1_g3/TRINITY_DN11902_c0_g1/TRINITY_DN2352_c0_g1/TRINITY_DN9739_c0_g1/TRINITY_DN2801_c0_g1/TRINITY_DN22316_c0_g2	
GO:0035336	long-chain fatty-acyl-CoA metabolic process	12/1853	30/22442	2.05×10^{-6}	0.00028	0.000264	TRINITY_DN10817_c0_g1/TRINITY_DN6954_c0_g1/TRINITY_DN1143_c0_g3/TRINITY_D	12

insects MDPI

							N18480_c0_g1/TRINITY_DN19122_c0_g1/TRINITY_DN32057_c0_g1/TRINITY_DN584_c1_g1/TRINITY_DN2762_c0_g1/TRINITY_DN1490_c0_g1/TRINITY_DN11864_c1_g1/TRINITY_DN30995_c0_g2/TRINITY_DN10734_c0_g2	
GO:0080019	fatty-acyl-CoA reductase (alcohol-forming) activity	12/1853	30/22442	2.05×10^{-6}	0.00028	0.000264	TRINITY_DN10817_c0_g1/TRINITY_DN6954_c0_g1/TRINITY_DN1143_c0_g3/TRINITY_DN18480_c0_g1/TRINITY_DN19122_c0_g1/TRINITY_DN32057_c0_g1/TRINITY_DN584_c1_g1/TRINITY_DN2762_c0_g1/TRINITY_DN1490_c0_g1/TRINITY_DN11864_c1_g1/TRINITY_DN30995_c0_g2/TRINITY_DN10734_c0_g2	12
GO:0102965	alcohol-forming fatty acyl-CoA reductase activity	10/1853	21/22442	2.17×10^{-6}	0.000284	0.000267	TRINITY_DN10817_c0_g1/TRINITY_DN6954_c0_g1/TRINITY_DN1143_c0_g3/TRINITY_DN18480_c0_g1/TRINITY_DN19122_c0_g1/TRINITY_DN584_c1_g1/TRINITY_DN2762_c0_g1/TRINITY_DN1490_c0_g1/TRINITY_DN11864_c1_g1/TRINITY_DN10734_c0_g2	10
GO:0005858	axonemal dynein complex	16/1853	52/22442	2.57×10^{-6}	0.000323	0.000304	TRINITY_DN6620_c0_g1/TRINITY_DN16194_c0_g1/TRINITY_DN13077_c0_g1/TRINITY_DN4420_c0_g1/TRINITY_DN	16

							41571_c0_g2/TRINITY_DN11109_c0_g1/TRINITY_DN12379_c0_g1/TRINITY_DN14529_c0_g1/TRINITY_DN12688_c0_g1/TRINITY_DN17632_c0_g1/TRINITY_DN15340_c0_g2/TRINITY_DN6620_c0_g2/TRINITY_DN32303_c0_g1/TRINITY_DN6439_c1_g1/TRINITY_DN47966_c0_g1/TRINITY_DN15707_c0_g1	
GO:0010025	wax biosynthetic process	12/1853	31/22442	3.09×10^{-6}	0.000372	0.00035	TRINITY_DN10817_c0_g1/TRINITY_DN6954_c0_g1/TRINITY_DN1143_c0_g3/TRINITY_DN18480_c0_g1/TRINITY_DN19122_c0_g1/TRINITY_DN32057_c0_g1/TRINITY_DN584_c1_g1/TRINITY_DN2762_c0_g1/TRINITY_DN1490_c0_g1/TRINITY_DN11864_c1_g1/TRINITY_DN30995_c0_g2/TRINITY_DN10734_c0_g2	12
GO:0022857	transmembrane transporter activity	53/1853	336/22442	3.72×10^{-6}	0.00043	0.000405	TRINITY_DN19255_c0_g1/TRINITY_DN9456_c0_g1/TRINITY_DN1216_c0_g1/TRINITY_DN2058_c3_g1/TRINITY_DN10337_c0_g1/TRINITY_DN11659_c0_g1/TRINITY_DN1667_c0_g1/TRINITY_DN9625_c0_g1/TRINITY_DN9935_c0_g1/TRINITY_DN4527_c1_g2/TRINITY_DN10962_c0_g1/TRINITY_DN	53

insects MDPI

7015_c0_g1/TRINITY_DN3034
2_c0_g1/TRINITY_DN13_c0_g
2/TRINITY_DN1243_c0_g1/TR
INITY_DN2310_c0_g1/TRINIT
Y_DN498_c0_g1/TRINITY_D
N22997_c0_g1/TRINITY_DN4
5783_c0_g1/TRINITY_DN1552
_c0_g1/TRINITY_DN7502_c1_
g1/TRINITY_DN35060_c1_g1/
TRINITY_DN32832_c0_g1/TRI
NITY_DN868_c0_g1/TRINITY
_DN20800_c0_g1/TRINITY_D
N22311_c0_g1/TRINITY_DN2
606_c0_g1/TRINITY_DN8087_
c0_g1/TRINITY_DN3238_c0_g
1/TRINITY_DN44158_c0_g1/T
RINITY_DN16973_c0_g1/TRI
NITY_DN11271_c0_g3/TRINI
TY_DN2668_c0_g1/TRINITY_
DN16399_c0_g1/TRINITY_DN
8582_c0_g3/TRINITY_DN3405
1_c0_g1/TRINITY_DN30018_c
0_g1/TRINITY_DN21797_c0_g
1/TRINITY_DN40711_c0_g1/T
RINITY_DN5851_c0_g1/TRIN
ITY_DN7339_c1_g1/TRINITY_
DN31352_c0_g1/TRINITY_DN
14345_c0_g2/TRINITY_DN100
30_c0_g1/TRINITY_DN3620_c
0_g1/TRINITY_DN1774_c0_g1
/TRINITY_DN1576_c2_g1/TRI
NITY_DN2356_c0_g1/TRINIT

							Y_DN56286_c0_g2/TRINITY_DN7624_c0_g1/TRINITY_DN473_c0_g1/TRINITY_DN28310_c0_g1/TRINITY_DN598_c0_g1	
GO:0008582	regulation of synaptic growth at neuromuscular junction	12/1853	32/22442	4.57×10^{-6}	0.000509	0.00048	TRINITY_DN144_c0_g1/TRINITY_DN4163_c0_g1/TRINITY_DN144_c1_g1/TRINITY_DN6005_c0_g1/TRINITY_DN2121_c1_g1/TRINITY_DN2121_c0_g1/TRINITY_DN7230_c0_g1/TRINITY_DN7230_c0_g2/TRINITY_DN11661_c0_g1/TRINITY_DN2559_c1_g1/TRINITY_DN3888_c0_g1/TRINITY_DN5168_c0_g1	12
GO:0005929	cilium	42/1853	249/22442	6.92×10^{-6}	0.000743	0.0007	TRINITY_DN16194_c0_g1/TRINITY_DN34305_c0_g1/TRINITY_DN7072_c0_g1/TRINITY_DN767_c0_g1/TRINITY_DN4270_c0_g1/TRINITY_DN5001_c0_g1/TRINITY_DN11395_c0_g1/TRINITY_DN3572_c0_g1/TRINITY_DN16707_c0_g1/TRINITY_DN6822_c0_g1/TRINITY_DN7398_c0_g1/TRINITY_DN10894_c0_g1/TRINITY_DN12881_c0_g1/TRINITY_DN10894_c0_g2/TRINITY_DN4955_c0_g1/TRINITY_DN12688_c0_g1/TRINITY_DN23026_c0_g1/TRINITY_DN18583_c0_g1/TRINITY_DN17632_c0_g1/TRINITY_D	42

insects

							N11712_c0_g1/TRINITY_DN13058_c0_g1/TRINITY_DN21369_c0_g1/TRINITY_DN8676_c0_g1/TRINITY_DN25714_c0_g1/TRINITY_DN6439_c1_g1/TRINITY_DN43314_c0_g2/TRINITY_DN15433_c0_g1/TRINITY_DN18447_c0_g1/TRINITY_DN15601_c0_g1/TRINITY_DN15233_c1_g1/TRINITY_DN11245_c0_g1/TRINITY_DN8698_c0_g1/TRINITY_DN12487_c0_g1/TRINITY_DN13283_c0_g1/TRINITY_DN28353_c0_g1/TRINITY_DN10924_c0_g1/TRINITY_DN7620_c1_g2/TRINITY_DN24749_c0_g1/TRINITY_DN10229_c0_g2/TRINITY_DN11058_c0_g1/TRINITY_DN24785_c0_g2/TRINITY_DN11321_c0_g1	
GO:0016616	oxidoreductase activity, with incorporation or reduction of molecular oxygen, acting on the CH-OH group of donors, NAD or NADP as acceptor	18/1853	71/22442	1.31×10^{-5}	0.001354	0.001276	TRINITY_DN10817_c0_g1/TRINITY_DN6954_c0_g1/TRINITY_DN2321_c0_g1/TRINITY_DN9813_c0_g1/TRINITY_DN9846_c0_g1/TRINITY_DN2112_c0_g1/TRINITY_DN1143_c0_g3/TRINITY_DN8204_c0_g1/TRINITY_DN12651_c0_g2/TRINITY_DN12651_c0_g1/TRINITY_DN19122_c0_g1/TRINITY_DN584_c1_g1/TRINITY_DN578_c0_g1/TRINITY_DN1490_c0_	18

insects

							g1/TRINITY_DN7223_c0_g1/TRINITY_DN2542_c0_g1/TRINITY_DN12945_c0_g2/TRINITY_DN30995_c0_g2	
GO:0007586	digestion	16/1853	59/22442	1.56×10^{-5}	0.00156	0.00147	TRINITY_DN3813_c0_g1/TRINITY_DN4067_c0_g1/TRINITY_DN4735_c0_g1/TRINITY_DN9371_c0_g1/TRINITY_DN872_c0_g1/TRINITY_DN9647_c0_g1/TRINITY_DN73059_c0_g1/TRINITY_DN425_c0_g1/TRINITY_DN21632_c0_g2/TRINITY_DN461_c0_g2/TRINITY_DN25822_c0_g1/TRINITY_DN17676_c0_g1/TRINITY_DN51634_c0_g2/TRINITY_DN12351_c0_g1/TRINITY_DN54378_c0_g1/TRINITY_DN75_c0_g2	16
GO:0006030	chitin metabolic process	18/1853	72/22442	1.61×10^{-5}	0.00156	0.00147	TRINITY_DN7331_c0_g1/TRINITY_DN381_c0_g1/TRINITY_DN384_c0_g1/TRINITY_DN2191_c0_g1/TRINITY_DN3135_c6_g1/TRINITY_DN5490_c0_g2/TRINITY_DN59401_c0_g1/TRINITY_DN30944_c0_g1/TRINITY_DN3135_c5_g1/TRINITY_DN4096_c0_g1/TRINITY_DN1563_c0_g1/TRINITY_DN725_c0_g1/TRINITY_DN11363_c0_g1/TRINITY_DN6870_c0_g1/TRINITY_DN16595_c0_g1/TRINITY_DN23642_c0_g1/TRINI	18

							TY_DN1022_c0_g2/TRINITY_DN813_c0_g2	
GO:0007224	smoothened signaling pathway	15/1853	54/22442	2.10 × 10^{-5}	0.00197	0.001855	TRINITY_DN22101_c0_g4/TRINITY_DN3460_c0_g1/TRINITY_DN11712_c0_g1/TRINITY_DN16994_c0_g1/TRINITY_DN13058_c0_g1/TRINITY_DN8676_c0_g1/TRINITY_DN15436_c0_g1/TRINITY_DN19590_c1_g2/TRINITY_DN6113_c0_g1/TRINITY_DN12487_c0_g1/TRINITY_DN13283_c0_g1/TRINITY_DN10924_c0_g1/TRINITY_DN2864_c0_g1/TRINITY_DN10229_c0_g2/TRINITY_DN2594_c0_g1	15
GO:0005230	extracellular ligand-gated ion channel activity	10/1853	26/22442	2.22 × 10^{-5}	0.002026	0.001909	TRINITY_DN35198_c0_g1/TRINITY_DN2080_c0_g1/TRINITY_DN10272_c0_g1/TRINITY_DN8811_c0_g1/TRINITY_DN19996_c0_g1/TRINITY_DN28824_c0_g1/TRINITY_DN11221_c0_g1/TRINITY_DN1074_c0_g2/TRINITY_DN12312_c0_g2/TRINITY_DN8819_c1_g1	10
GO:0004553	hydrolase activity, hydrolyzing O-glycosyl compounds	19/1853	83/22442	3.61 × 10^{-5}	0.00319	0.003005	TRINITY_DN745_c0_g1/TRINITY_DN12857_c0_g1/TRINITY_DN4232_c0_g2/TRINITY_DN15210_c0_g1/TRINITY_DN12107_c0_g1/TRINITY_DN752_c0_g1/TRINITY_DN47223_c0_g1/TRINITY_DN29361_c0_g1/TR	19

							INITY_DN71241_c0_g1/TRINITY_DN4454_c1_g1/TRINITY_DN487_c0_g1/TRINITY_DN4956_c0_g1/TRINITY_DN4423_c0_g1/TRINITY_DN96_c0_g1/TRINITY_DN8357_c0_g1/TRINITY_DN58410_c0_g1/TRINITY_DN3530_c2_g1/TRINITY_DN2001_c0_g2/TRINITY_DN1319_c1_g1	
GO:0006583	melanin biosynthetic process from tyrosine	8/1853	18/22442	4.38×10^{-5}	0.003762	0.003544	TRINITY_DN2618_c0_g1/TRINITY_DN9698_c0_g1/TRINITY_DN49_c0_g4/TRINITY_DN912_c0_g1/TRINITY_DN23099_c0_g1/TRINITY_DN1808_c0_g2/TRINITY_DN28190_c0_g1/TRINITY_DN14599_c0_g1	8
GO:0098826	endoplasmic reticulum tubular network membrane	8/1853	19/22442	7.02×10^{-5}	0.005341	0.005031	TRINITY_DN863_c0_g1/TRINITY_DN144_c0_g3/TRINITY_DN1911_c0_g1/TRINITY_DN144_c1_g2/TRINITY_DN144_c1_g1/TRINITY_DN12319_c0_g1/TRINITY_DN14988_c0_g1/TRINITY_DN2121_c0_g1	8
GO:1990809	endoplasmic reticulum tubular network membrane organization	8/1853	19/22442	7.02×10^{-5}	0.005341	0.005031	TRINITY_DN863_c0_g1/TRINITY_DN144_c0_g3/TRINITY_DN1911_c0_g1/TRINITY_DN144_c1_g2/TRINITY_DN144_c1_g1/TRINITY_DN12319_c0_g1/TRINITY_DN14988_c0_g1/TRINITY_DN2121_c0_g1	8

GO:0102336	3-oxo-arachidoyl-CoA synthase activity	9/1853	24/22442	7.28×10^{-5}	0.005341	0.005031	TRINITY_DN22698_c0_g1/TRINITY_DN8883_c1_g1/TRINITY_DN17518_c0_g1/TRINITY_DN79156_c0_g1/TRINITY_DN10836_c0_g1/TRINITY_DN34038_c0_g1/TRINITY_DN6234_c0_g1/TRINITY_DN1615_c0_g1/TRINITY_DN1517_c0_g3	9
GO:0102337	3-oxo-cerotoyl-CoA synthase activity	9/1853	24/22442	7.28×10^{-5}	0.005341	0.005031	TRINITY_DN22698_c0_g1/TRINITY_DN8883_c1_g1/TRINITY_DN17518_c0_g1/TRINITY_DN79156_c0_g1/TRINITY_DN10836_c0_g1/TRINITY_DN34038_c0_g1/TRINITY_DN6234_c0_g1/TRINITY_DN1615_c0_g1/TRINITY_DN1517_c0_g3	9
GO:0102338	3-oxo-lignoceronyl-CoA synthase activity	9/1853	24/22442	7.28×10^{-5}	0.005341	0.005031	TRINITY_DN22698_c0_g1/TRINITY_DN8883_c1_g1/TRINITY_DN17518_c0_g1/TRINITY_DN79156_c0_g1/TRINITY_DN10836_c0_g1/TRINITY_DN34038_c0_g1/TRINITY_DN6234_c0_g1/TRINITY_DN1615_c0_g1/TRINITY_DN1517_c0_g3	9
GO:0102756	very-long-chain 3-ketoacyl-CoA synthase activity	9/1853	24/22442	7.28×10^{-5}	0.005341	0.005031	TRINITY_DN22698_c0_g1/TRINITY_DN8883_c1_g1/TRINITY_DN17518_c0_g1/TRINITY_DN79156_c0_g1/TRINITY_DN10836_c0_g1/TRINITY_DN34038_c0_g1/TRINITY_DN6234_c0_g1/TRINITY_DN1615_c0_g1/TRINITY_DN1517_c0_g3	9

GO:0016614	oxidoreductase activity, acting on CH-OH group of donors	14/1853	54/22442	9.01×10^{-5}	0.006448	0.006074	TRINITY_DN6443_c0_g1/TRINITY_DN2871_c0_g1/TRINITY_DN3880_c0_g1/TRINITY_DN11082_c1_g1/TRINITY_DN13622_c0_g1/TRINITY_DN2937_c1_g1/TRINITY_DN25056_c0_g1/TRINITY_DN2937_c0_g1/TRINITY_DN7660_c0_g1/TRINITY_DN2937_c0_g2/TRINITY_DN10101_c0_g1/TRINITY_DN22329_c0_g1/TRINITY_DN102432_c0_g1/TRINITY_DN40191_c0_g1	14
GO:0006629	lipid metabolic process	36/1853	225/22442	9.42×10^{-5}	0.006587	0.006205	TRINITY_DN11877_c0_g2/TRINITY_DN10817_c0_g1/TRINITY_DN6954_c0_g1/TRINITY_DN4067_c0_g1/TRINITY_DN53219_c0_g1/TRINITY_DN4735_c0_g1/TRINITY_DN9371_c0_g1/TRINITY_DN150_c0_g1/TRINITY_DN16034_c0_g1/TRINITY_DN4708_c1_g1/TRINITY_DN73059_c0_g1/TRINITY_DN6686_c0_g2/TRINITY_DN6729_c1_g1/TRINITY_DN19122_c0_g1/TRINITY_DN32057_c0_g1/TRINITY_DN781_c0_g1/TRINITY_DN19837_c0_g1/TRINITY_DN9700_c0_g1/TRINITY_DN7128_c0_g1/TRINITY_DN12204_c0_g1/TRINITY_DN818_c0_g1/TRINITY_DN5280_c1_g	36

							1/TRINITY_DN1490_c0_g1/TRINITY_DN11223_c0_g2/TRINITY_DN2499_c0_g1/TRINITY_DN17676_c0_g1/TRINITY_DN5909_c1_g1/TRINITY_DN1812_c0_g1/TRINITY_DN2236_c0_g1/TRINITY_DN1719_c0_g1/TRINITY_DN30995_c0_g2/TRINITY_DN21301_c0_g1/TRINITY_DN3191_c0_g1/TRINITY_DN1812_c0_g3/TRINITY_DN8820_c0_g1/TRINITY_DN1719_c0_g4	
GO:0005779	integral component of peroxisomal membrane	11/1853	37/22442	0.000134	0.009178	0.008646	TRINITY_DN10817_c0_g1/TRINITY_DN6954_c0_g1/TRINITY_DN1143_c0_g3/TRINITY_DN18480_c0_g1/TRINITY_DN19122_c0_g1/TRINITY_DN584_c1_g1/TRINITY_DN2762_c0_g1/TRINITY_DN1490_c0_g1/TRINITY_DN11864_c1_g1/TRINITY_DN7282_c0_g2/TRINITY_DN10734_c0_g2	11
GO:0070330	aromatase activity	18/1853	84/22442	0.000142	0.009512	0.008961	TRINITY_DN2531_c2_g1/TRINITY_DN2955_c0_g2/TRINITY_DN54392_c0_g2/TRINITY_DN1949_c0_g1/TRINITY_DN2871_c0_g2/TRINITY_DN482_c0_g1/TRINITY_DN35617_c0_g1/TRINITY_DN18271_c1_g2/TRINITY_DN19412_c0_g1/TRINITY_DN13080_c1_g2/TRINI	18

							TY_DN1029_c0_g1/TRINITY_DN4749_c0_g3/TRINITY_DN18210_c0_g1/TRINITY_DN30772_c0_g1/TRINITY_DN21883_c1_g1/TRINITY_DN1316_c6_g1/TRINITY_DN2801_c0_g1/TRINITY_DN22316_c0_g2	
GO:0018095	protein polyglutamylation	7/1853	16/22442	0.000152	0.009918	0.009343	TRINITY_DN5400_c0_g1/TRINITY_DN19594_c0_g1/TRINITY_DN8472_c0_g1/TRINITY_DN14808_c1_g1/TRINITY_DN8698_c0_g1/TRINITY_DN38209_c0_g1/TRINITY_DN24749_c0_g1	7
GO:0016042	lipid catabolic process	25/1853	139/22442	0.000169	0.010823	0.010195	TRINITY_DN4067_c0_g1/TRINITY_DN4735_c0_g1/TRINITY_DN9371_c0_g1/TRINITY_DN1717_c4_g1/TRINITY_DN7275_c0_g1/TRINITY_DN4708_c1_g1/TRINITY_DN3261_c0_g1/TRINITY_DN23560_c0_g1/TRINITY_DN4369_c0_g1/TRINITY_DN16662_c0_g1/TRINITY_DN73059_c0_g1/TRINITY_DN2904_c0_g1/TRINITY_DN114_c0_g1/TRINITY_DN19837_c0_g1/TRINITY_DN20592_c0_g1/TRINITY_DN1754_c0_g1/TRINITY_DN15069_c0_g1/TRINITY_DN4581_c0_g1/TRINITY_DN5280_c1_g1/TRINITY_DN4736_c0_g3/TRINITY_DN17676	25

							_c0_g1/TRINITY_DN1537_c0_g2/TRINITY_DN51922_c0_g1/TRINITY_DN7_c0_g2/TRINITY_DN7445_c0_g1	
GO:0010248	establishment or maintenance of transmembrane electrochemical gradient	6/1853	12/22442	0.000188	0.011724	0.011044	TRINITY_DN10962_c0_g1/TRINITY_DN21424_c0_g1/TRINITY_DN3457_c0_g1/TRINITY_DN28307_c0_g3/TRINITY_DN5429_c0_g1/TRINITY_DN4595_c0_g1	6
GO:0030246	carbohydrate binding	29/1853	173/22442	0.000192	0.011724	0.011044	TRINITY_DN97_c0_g1/TRINITY_DN2137_c0_g2/TRINITY_DN2137_c0_g1/TRINITY_DN2898_c0_g1/TRINITY_DN97_c1_g1/TRINITY_DN5639_c0_g1/TRINITY_DN8621_c0_g1/TRINITY_DN5930_c0_g1/TRINITY_DN2108_c0_g1/TRINITY_DN15974_c0_g1/TRINITY_DN752_c0_g1/TRINITY_DN20294_c1_g1/TRINITY_DN47223_c0_g1/TRINITY_DN6352_c0_g1/TRINITY_DN7894_c0_g2/TRINITY_DN702_c0_g1/TRINITY_DN71241_c0_g1/TRINITY_DN6251_c0_g1/TRINITY_DN4956_c0_g1/TRINITY_DN4423_c0_g1/TRINITY_DN96_c0_g1/TRINITY_DN4495_c0_g1/TRINITY_DN8979_c0_g1/TRINITY_DN22087_c0_g2/TRINITY_DN4703_c0_g1/TRINITY_DN58410	29

							_c0_g1/TRINITY_DN6784_c0_g1/TRINITY_DN6876_c0_g1/TRINITY_DN22087_c0_g1	
GO:0008237	metallopeptidase activity	18/1853	86/22442	0.000195	0.011724	0.011044	TRINITY_DN5781_c0_g1/TRINITY_DN7271_c0_g1/TRINITY_DN15191_c0_g1/TRINITY_DN8267_c0_g1/TRINITY_DN1448_c0_g2/TRINITY_DN7996_c0_g1/TRINITY_DN18930_c0_g1/TRINITY_DN2723_c0_g1/TRINITY_DN47563_c0_g1/TRINITY_DN8234_c0_g1/TRINITY_DN1346_c0_g1/TRINITY_DN864_c0_g1/TRINITY_DN29497_c0_g2/TRINITY_DN5615_c0_g1/TRINITY_DN1076_c0_g1/TRINITY_DN6125_c0_g1/TRINITY_DN1107_c0_g1/TRINITY_DN14597_c0_g1	18
GO:0003777	microtubule motor activity	24/1853	134/22442	0.000241	0.014236	0.01341	TRINITY_DN6620_c0_g1/TRINITY_DN16194_c0_g1/TRINITY_DN13077_c0_g1/TRINITY_DN12837_c0_g1/TRINITY_DN33652_c0_g1/TRINITY_DN12688_c0_g1/TRINITY_DN6439_c1_g2/TRINITY_DN23026_c0_g1/TRINITY_DN17632_c0_g1/TRINITY_DN15340_c0_g2/TRINITY_DN23273_c0_g1/TRINITY_DN5804_c0_g1/TRINITY_DN6620_c0_g2/TRINITY_DN32303_c0_g1/TRINITY_DN1105	24

							_c0_g1/TRINITY_DN28402_c0_g1/TRINITY_DN6439_c1_g1/TRINITY_DN33652_c1_g1/TRINITY_DN22894_c0_g1/TRINITY_DN47966_c0_g1/TRINITY_DN3140_c1_g1/TRINITY_DN21521_c0_g1/TRINITY_DN11321_c0_g1/TRINITY_DN15707_c0_g1	
GO:0050662	coenzyme binding	16/1853	74/22442	0.000294	0.016808	0.015833	TRINITY_DN10817_c0_g1/TRINITY_DN6954_c0_g1/TRINITY_DN1123_c1_g1/TRINITY_DN1143_c0_g3/TRINITY_DN18480_c0_g1/TRINITY_DN19122_c0_g1/TRINITY_DN584_c1_g1/TRINITY_DN5522_c0_g1/TRINITY_DN2762_c0_g1/TRINITY_DN7223_c0_g1/TRINITY_DN11864_c1_g1/TRINITY_DN3621_c0_g1/TRINITY_DN30995_c0_g2/TRINITY_DN22520_c0_g1/TRINITY_DN27557_c0_g1/TRINITY_DN10734_c0_g2	16
GO:0034446	substrate adhesion-dependent cell spreading	10/1853	34/22442	0.000298	0.016808	0.015833	TRINITY_DN9038_c0_g1/TRINITY_DN13801_c0_g1/TRINITY_DN13223_c0_g1/TRINITY_DN12075_c0_g1/TRINITY_DN1352_c0_g1/TRINITY_DN1295_c0_g1/TRINITY_DN1715_c0_g1/TRINITY_DN5591_c2_g1/TRINITY_DN8671_c0_g1/TRINITY_DN12050_c0_g1	10

GO:0008340	determination of adult lifespan	23/1853	128/22442	0.000307	0.016808	0.015833	TRINITY_DN3754_c0_g1/TRINITY_DN3172_c0_g1/TRINITY_DN9232_c0_g1/TRINITY_DN8249_c0_g1/TRINITY_DN60_c0_g2/TRINITY_DN13801_c0_g1/TRINITY_DN48546_c0_g1/TRINITY_DN8400_c0_g1/TRINITY_DN124461_c0_g1/TRINITY_DN318_c0_g1/TRINITY_DN22284_c0_g1/TRINITY_DN16714_c0_g1/TRINITY_DN29492_c0_g1/TRINITY_DN7230_c0_g1/TRINITY_DN7230_c0_g2/TRINITY_DN5261_c0_g1/TRINITY_DN29892_c0_g1/TRINITY_DN10137_c0_g1/TRINITY_DN27419_c0_g1/TRINITY_DN2393_c0_g1/TRINITY_DN17400_c0_g1/TRINITY_DN10138_c0_g1/TRINITY_DN33651_c0_g1	23
GO:0005975	carbohydrate metabolic process	43/1853	302/22442	0.000314	0.016808	0.015833	TRINITY_DN745_c0_g1/TRINITY_DN2898_c0_g1/TRINITY_DN9813_c0_g1/TRINITY_DN9846_c0_g1/TRINITY_DN16732_c0_g1/TRINITY_DN12857_c0_g1/TRINITY_DN2112_c0_g1/TRINITY_DN4232_c0_g2/TRINITY_DN15210_c0_g1/TRINITY_DN8204_c0_g1/TRINITY_DN12107_c0_g1/TRINITY_DN12651_c0_g2/TRINITY_DN259	43

							6_c0_g1/TRINITY_DN12651_c0_g1/TRINITY_DN752_c0_g1/TRINITY_DN21888_c0_g1/TRINITY_DN47223_c0_g1/TRINITY_DN5917_c0_g1/TRINITY_DN29361_c0_g1/TRINITY_DN71241_c0_g1/TRINITY_DN4454_c1_g1/TRINITY_DN487_c0_g1/TRINITY_DN16048_c0_g1/TRINITY_DN5138_c0_g1/TRINITY_DN4956_c0_g1/TRINITY_DN54367_c0_g2/TRINITY_DN4423_c0_g1/TRINITY_DN96_c0_g1/TRINITY_DN4495_c0_g1/TRINITY_DN4096_c0_g1/TRINITY_DN3543_c0_g1/TRINITY_DN8357_c0_g1/TRINITY_DN1978_c0_g3/TRINITY_DN10314_c0_g1/TRINITY_DN58410_c0_g1/TRINITY_DN65013_c0_g1/TRINITY_DN3530_c2_g1/TRINITY_DN29983_c0_g1/TRINITY_DN2001_c0_g2/TRINITY_DN1319_c1_g1/TRINITY_DN9962_c0_g1/TRINITY_DN4401_c0_g2/TRINITY_DN10882_c0_g1	
GO:0030286	dynein complex	32/1853	204/22442	0.000323	0.016808	0.015833	TRINITY_DN6620_c0_g1/TRINITY_DN16194_c0_g1/TRINITY_DN34305_c0_g1/TRINITY_DN1579_c0_g1/TRINITY_DN13077_c0_g1/TRINITY_DN128	32

							37_c0_g1/TRINITY_DN3572_c0_g1/TRINITY_DN33652_c0_g1/TRINITY_DN18257_c0_g1/TRINITY_DN12688_c0_g1/TRINITY_DN6439_c1_g2/TRINITY_DN23026_c0_g1/TRINITY_DN18583_c0_g1/TRINITY_DN6439_c0_g2/TRINITY_DN17632_c0_g1/TRINITY_DN15340_c0_g2/TRINITY_DN23273_c0_g1/TRINITY_DN5804_c0_g1/TRINITY_DN6620_c0_g2/TRINITY_DN32303_c0_g1/TRINITY_DN1105_c0_g1/TRINITY_DN19322_c0_g1/TRINITY_DN48117_c0_g1/TRINITY_DN6439_c0_g3/TRINITY_DN26604_c0_g1/TRINITY_DN6439_c1_g1/TRINITY_DN33652_c1_g1/TRINITY_DN22894_c0_g1/TRINITY_DN47966_c0_g1/TRINITY_DN11058_c0_g1/TRINITY_DN11321_c0_g1/TRINITY_DN15707_c0_g1	
GO:0030570	pectate lyase activity	6/1853	13/22442	0.000324	0.016808	0.015833	TRINITY_DN13286_c0_g1/TRINITY_DN5977_c0_g1/TRINITY_DN1745_c1_g1/TRINITY_DN15007_c0_g2/TRINITY_DN2777_c0_g1/TRINITY_DN4884_c0_g1	6
GO:0071711	basement membrane organization	6/1853	13/22442	0.000324	0.016808	0.015833	TRINITY_DN13223_c0_g1/TRINITY_DN1556_c0_g3/TRINIT	6

							Y_DN1352_c0_g1/TRINITY_DN1295_c0_g1/TRINITY_DN1715_c0_g1/TRINITY_DN12050_c0_g1	
GO:0003824	catalytic activity	39/1853	267/22442	0.000349	0.017775	0.016744	TRINITY_DN7803_c0_g1/TRINITY_DN10817_c0_g1/TRINITY_DN6954_c0_g1/TRINITY_DN1716_c0_g1/TRINITY_DN1123_c1_g1/TRINITY_DN13977_c0_g1/TRINITY_DN8030_c0_g1/TRINITY_DN4248_c0_g1/TRINITY_DN8735_c0_g1/TRINITY_DN1143_c0_g3/TRINITY_DN5340_c0_g3/TRINITY_DN14080_c0_g1/TRINITY_DN18480_c0_g1/TRINITY_DN19122_c0_g1/TRINITY_DN584_c1_g1/TRINITY_DN4357_c0_g1/TRINITY_DN1548_c2_g3/TRINITY_DN12704_c0_g1/TRINITY_DN19102_c0_g1/TRINITY_DN54367_c0_g2/TRINITY_DN28150_c0_g1/TRINITY_DN5522_c0_g1/TRINITY_DN9699_c1_g1/TRINITY_DN2762_c0_g1/TRINITY_DN278_c1_g1/TRINITY_DN7223_c0_g1/TRINITY_DN10314_c0_g1/TRINITY_DN16342_c0_g1/TRINITY_DN11864_c1_g1/TRINITY_DN6036_c0_g1/TRINITY_DN788_c0_g1/TRINITY_DN3621_c0_g1/TRINIT	39

							Y_DN7_c0_g2/TRINITY_DN1567_c0_g1/TRINITY_DN30995_c0_g2/TRINITY_DN3191_c0_g1/TRINITY_DN22520_c0_g1/TRINITY_DN27557_c0_g1/TRINITY_DN10734_c0_g2	
GO:0004356	glutamate-ammonia ligase activity	7/1853	18/22442	0.000364	0.017951	0.01691	TRINITY_DN6327_c0_g1/TRINITY_DN13857_c0_g1/TRINITY_DN20347_c0_g1/TRINITY_DN25935_c0_g1/TRINITY_DN35034_c0_g4/TRINITY_DN3033_c0_g2/TRINITY_DN37526_c0_g1	7
GO:0006542	glutamine biosynthetic process	7/1853	18/22442	0.000364	0.017951	0.01691	TRINITY_DN6327_c0_g1/TRINITY_DN13857_c0_g1/TRINITY_DN20347_c0_g1/TRINITY_DN25935_c0_g1/TRINITY_DN35034_c0_g4/TRINITY_DN3033_c0_g2/TRINITY_DN37526_c0_g1	7
GO:0005764	lysosome	45/1853	323/22442	0.000376	0.018148	0.017095	TRINITY_DN19567_c0_g1/TRINITY_DN647_c0_g1/TRINITY_DN1217_c0_g1/TRINITY_DN2286_c0_g3/TRINITY_DN14089_c0_g1/TRINITY_DN7146_c0_g1/TRINITY_DN10957_c0_g1/TRINITY_DN16716_c0_g1/TRINITY_DN2596_c0_g1/TRINITY_DN19631_c0_g1/TRINITY_DN9929_c0_g1/TRINITY_DN9224_c0_g1/TRINITY_DN20294_c1_g1/TRINITY_DN10534_c0	45

							_g1/TRINITY_DN114_c0_g1/TRINITY_DN4321_c0_g1/TRINITY_DN702_c0_g1/TRINITY_DN14025_c0_g1/TRINITY_DN21401_c0_g1/TRINITY_DN8634_c0_g2/TRINITY_DN2886_c2_g1/TRINITY_DN8634_c0_g1/TRINITY_DN430_c0_g1/TRINITY_DN4956_c0_g1/TRINITY_DN1742_c0_g1/TRINITY_DN25822_c0_g1/TRINITY_DN8357_c0_g1/TRINITY_DN4578_c0_g2/TRINITY_DN11686_c0_g1/TRINITY_DN34051_c0_g1/TRINITY_DN29497_c0_g2/TRINITY_DN1396_c0_g1/TRINITY_DN9840_c0_g2/TRINITY_DN2236_c0_g1/TRINITY_DN3402_c0_g1/TRINITY_DN1587_c0_g1/TRINITY_DN21874_c0_g1/TRINITY_DN3079_c0_g1/TRINITY_DN2788_c2_g1/TRINITY_DN6894_c0_g1/TRINITY_DN17400_c0_g1/TRINITY_DN1319_c1_g1/TRINITY_DN1507_c0_g1/TRINITY_DN6608_c0_g3/TRINITY_DN17065_c0_g1	
GO:0015574	trehalose transmembrane transporter activity	17/1853	83/22442	0.00038	0.018148	0.017095	TRINITY_DN2058_c3_g1/TRINITY_DN9935_c0_g1/TRINITY_DN7015_c0_g1/TRINITY_DN2310_c0_g1/TRINITY_DN22997_c0_g1/TRINITY_DN7502_	17

							c1_g1/TRINITY_DN35060_c1_g1/TRINITY_DN32832_c0_g1/TRINITY_DN20800_c0_g1/TRINITY_DN44158_c0_g1/TRINITY_DN16399_c0_g1/TRINITY_DN30018_c0_g1/TRINITY_DN31352_c0_g1/TRINITY_DN14345_c0_g2/TRINITY_DN56286_c0_g2/TRINITY_DN7624_c0_g1/TRINITY_DN598_c0_g1	
GO:0008611	ether lipid biosynthetic process	13/1853	55/22442	0.000428	0.020129	0.018962	TRINITY_DN10817_c0_g1/TRINITY_DN6954_c0_g1/TRINITY_DN1143_c0_g3/TRINITY_DN61713_c0_g1/TRINITY_DN18480_c0_g1/TRINITY_DN19122_c0_g1/TRINITY_DN584_c1_g1/TRINITY_DN38156_c0_g1/TRINITY_DN18333_c0_g1/TRINITY_DN2762_c0_g1/TRINITY_DN1490_c0_g1/TRINITY_DN11864_c1_g1/TRINITY_DN10734_c0_g2	13
GO:0007286	spermatid development	12/1853	49/22442	0.0005	0.023114	0.021774	TRINITY_DN7175_c0_g1/TRINITY_DN5268_c0_g1/TRINITY_DN5141_c0_g1/TRINITY_DN5001_c0_g1/TRINITY_DN4202_c0_g1/TRINITY_DN8480_c0_g1/TRINITY_DN6225_c1_g1/TRINITY_DN6785_c0_g1/TRINITY_DN5776_c0_g1/TRINITY_DN258_c0_g2/TRINITY_D	12

							N7088_c0_g1/TRINITY_DN11156_c0_g1	
GO:0042811	pheromone biosynthetic process	6/1853	14/22442	0.000528	0.023686	0.022313	TRINITY_DN6729_c1_g1/TRINITY_DN560_c1_g2/TRINITY_DN9700_c0_g1/TRINITY_DN1719_c0_g1/TRINITY_DN13630_c0_g1/TRINITY_DN1719_c0_g4	6
GO:0050062	long-chain-fatty-acyl-CoA reductase activity	6/1853	14/22442	0.000528	0.023686	0.022313	TRINITY_DN10817_c0_g1/TRINITY_DN6954_c0_g1/TRINITY_DN19122_c0_g1/TRINITY_DN32057_c0_g1/TRINITY_DN1490_c0_g1/TRINITY_DN30995_c0_g2	6
GO:0008061	chitin binding	19/1853	101/22442	0.000551	0.024359	0.022946	TRINITY_DN7331_c0_g1/TRINITY_DN381_c0_g1/TRINITY_DN384_c0_g1/TRINITY_DN2191_c0_g1/TRINITY_DN3135_c6_g1/TRINITY_DN5490_c0_g2/TRINITY_DN59401_c0_g1/TRINITY_DN30944_c0_g1/TRINITY_DN16048_c0_g1/TRINITY_DN3135_c5_g1/TRINITY_DN4096_c0_g1/TRINITY_DN1563_c0_g1/TRINITY_DN725_c0_g1/TRINITY_DN11363_c0_g1/TRINITY_DN6870_c0_g1/TRINITY_DN16595_c0_g1/TRINITY_DN23642_c0_g1/TRINITY_DN1022_c0_g2/TRINITY_DN813_c0_g2	19

GO:0015771	trehalose transport	17/1853	86/22442	0.000586	0.025554	0.024072	TRINITY_DN2058_c3_g1/TRINITY_DN9935_c0_g1/TRINITY_DN7015_c0_g1/TRINITY_DN2310_c0_g1/TRINITY_DN22997_c0_g1/TRINITY_DN7502_c1_g1/TRINITY_DN35060_c1_g1/TRINITY_DN32832_c0_g1/TRINITY_DN20800_c0_g1/TRINITY_DN44158_c0_g1/TRINITY_DN16399_c0_g1/TRINITY_DN30018_c0_g1/TRINITY_DN31352_c0_g1/TRINITY_DN14345_c0_g2/TRINITY_DN56286_c0_g2/TRINITY_DN7624_c0_g1/TRINITY_DN598_c0_g1	17
GO:0003854	3-beta-hydroxy-delta5-steroid dehydrogenase activity	8/1853	25/22442	0.000641	0.027531	0.025934	TRINITY_DN10817_c0_g1/TRINITY_DN6954_c0_g1/TRINITY_DN1143_c0_g3/TRINITY_DN19122_c0_g1/TRINITY_DN584_c1_g1/TRINITY_DN1490_c0_g1/TRINITY_DN7223_c0_g1/TRINITY_DN30995_c0_g2	8
GO:0006633	fatty acid biosynthetic process	23/1853	136/22442	0.000745	0.031553	0.029723	TRINITY_DN11877_c0_g2/TRINITY_DN1716_c0_g1/TRINITY_DN1539_c0_g1/TRINITY_DN61713_c0_g1/TRINITY_DN6729_c1_g1/TRINITY_DN8292_c0_g1/TRINITY_DN9700_c0_g1/TRINITY_DN17360_c0_g2/TRINITY_DN38156_c0_g1/TRINITY_DN18333_c0_g1/TRINITY_DN79156_c0_g1/TRINITY	23

insects MDPI

							_DN53472_c0_g1/TRINITY_DN10836_c0_g1/TRINITY_DN6063_c0_g2/TRINITY_DN1817_c0_g1/TRINITY_DN25253_c0_g1/TRINITY_DN6234_c0_g1/TRINITY_DN1615_c0_g1/TRINITY_DN1517_c0_g3/TRINITY_DN1719_c0_g1/TRINITY_DN51922_c0_g1/TRINITY_DN12865_c0_g1/TRINITY_DN1719_c0_g4	
GO:0004806	triglyceride lipase activity	15/1853	73/22442	0.000793	0.03281	0.030907	TRINITY_DN9371_c0_g1/TRINITY_DN1539_c0_g1/TRINITY_DN4708_c1_g1/TRINITY_DN3261_c0_g1/TRINITY_DN23560_c0_g1/TRINITY_DN4369_c0_g3/TRINITY_DN20592_c0_g1/TRINITY_DN5280_c1_g1/TRINITY_DN4736_c0_g3/TRINITY_DN2499_c0_g1/TRINITY_DN1537_c0_g2/TRINITY_DN1126_c0_g2/TRINITY_DN12899_c0_g1/TRINITY_DN7445_c0_g1/TRINITY_DN3191_c0_g1	15
GO:0005890	sodium:potassium-exchanging ATPase complex	6/1853	15/22442	0.000818	0.03281	0.030907	TRINITY_DN21424_c0_g1/TRINITY_DN8400_c0_g1/TRINITY_DN3457_c0_g1/TRINITY_DN28307_c0_g3/TRINITY_DN5429_c0_g1/TRINITY_DN4595_c0_g1	6
GO:0006565	L-serine catabolic process	6/1853	15/22442	0.000818	0.03281	0.030907	TRINITY_DN4912_c0_g1/TRINITY_DN9212_c0_g1/TRINIT	6

							Y_DN21275_c0_g1/TRINITY_DN6496_c0_g1/TRINITY_DN8413_c0_g1/TRINITY_DN1251_c0_g1	
GO:0097067	cellular response to thyroid hormone stimulus	6/1853	15/22442	0.000818	0.03281	0.030907	TRINITY_DN647_c0_g1/TRINITY_DN1217_c0_g1/TRINITY_DN10957_c0_g1/TRINITY_DN4321_c0_g1/TRINITY_DN1587_c0_g1/TRINITY_DN21874_c0_g1	6
GO:0007029	endoplasmic reticulum organization	14/1853	66/22442	0.000842	0.033322	0.03139	TRINITY_DN863_c0_g1/TRINITY_DN144_c0_g3/TRINITY_DN1911_c0_g1/TRINITY_DN144_c0_g1/TRINITY_DN4163_c0_g1/TRINITY_DN144_c1_g2/TRINITY_DN144_c1_g1/TRINITY_DN12319_c0_g1/TRINITY_DN14988_c0_g1/TRINITY_DN2121_c1_g1/TRINITY_DN2121_c0_g1/TRINITY_DN997_c2_g1/TRINITY_DN85_c1_g1/TRINITY_DN5085_c0_g1	14
GO:0097546	ciliary base	8/1853	26/22442	0.000859	0.033556	0.03161	TRINITY_DN13060_c0_g1/TRINITY_DN28846_c0_g1/TRINITY_DN8012_c1_g1/TRINITY_DN8676_c0_g1/TRINITY_DN16966_c0_g2/TRINITY_DN11245_c0_g1/TRINITY_DN28353_c0_g1/TRINITY_DN10924_c0_g1	8
GO:0097228	sperm principal piece	7/1853	21/22442	0.001067	0.041141	0.038755	TRINITY_DN6620_c0_g1/TRINITY_DN1579_c0_g1/TRINIT	7

							Y_DN20426_c0_g1/TRINITY_DN15340_c0_g2/TRINITY_DN26604_c0_g1/TRINITY_DN12487_c0_g1/TRINITY_DN5700_c0_g1	
GO:0035002	liquid clearance, open tracheal system	8/1853	27/22442	0.001133	0.041816	0.039391	TRINITY_DN10817_c0_g1/TRINITY_DN6954_c0_g1/TRINITY_DN19122_c0_g1/TRINITY_DN32057_c0_g1/TRINITY_DN14671_c0_g1/TRINITY_DN18560_c0_g1/TRINITY_DN1490_c0_g1/TRINITY_DN30995_c0_g2	8
GO:0048691	positive regulation of axon extension involved in regeneration	8/1853	27/22442	0.001133	0.041816	0.039391	TRINITY_DN144_c0_g1/TRINITY_DN4163_c0_g1/TRINITY_DN144_c1_g1/TRINITY_DN2121_c1_g1/TRINITY_DN6686_c0_g2/TRINITY_DN2121_c0_g1/TRINITY_DN882_c0_g1/TRINITY_DN16980_c0_g1	8
GO:0032561	guanyl ribonucleotide binding	5/1853	11/22442	0.001154	0.041816	0.039391	TRINITY_DN144_c0_g1/TRINITY_DN4163_c0_g1/TRINITY_DN144_c1_g1/TRINITY_DN2121_c1_g1/TRINITY_DN2121_c0_g1	5
GO:0042834	peptidoglycan binding	5/1853	11/22442	0.001154	0.041816	0.039391	TRINITY_DN9869_c0_g1/TRINITY_DN671_c2_g1/TRINITY_DN16122_c0_g1/TRINITY_DN10138_c0_g1/TRINITY_DN3630_c1_g1	5
GO:0050707	regulation of cytokine secretion	5/1853	11/22442	0.001154	0.041816	0.039391	TRINITY_DN7701_c0_g1/TRINITY_DN10572_c0_g1/TRINI	5

							TY_DN19631_c0_g1/TRINITY_DN10572_c0_g2/TRINITY_DN8944_c0_g1	
GO:0031114	regulation of microtubule depolymerization	6/1853	16/22442	0.001218	0.043613	0.041084	TRINITY_DN144_c0_g1/TRINITY_DN4163_c0_g1/TRINITY_DN144_c1_g1/TRINITY_DN6005_c0_g1/TRINITY_DN2121_c1_g1/TRINITY_DN2121_c0_g1	6
GO:0001669	acrosomal vesicle	11/1853	47/22442	0.001271	0.044949	0.042342	TRINITY_DN7175_c0_g1/TRINITY_DN5141_c0_g1/TRINITY_DN10099_c0_g1/TRINITY_DN20426_c0_g1/TRINITY_DN13060_c0_g1/TRINITY_DN28846_c0_g1/TRINITY_DN16994_c0_g1/TRINITY_DN14923_c0_g1/TRINITY_DN21874_c0_g1/TRINITY_DN22278_c0_g1/TRINITY_DN23267_c0_g1	11
GO:0036158	outer dynein arm assembly	17/1853	92/22442	0.001296	0.045306	0.042679	TRINITY_DN6620_c0_g1/TRINITY_DN6235_c0_g1/TRINITY_DN16194_c0_g1/TRINITY_DN1579_c0_g1/TRINITY_DN3572_c0_g1/TRINITY_DN7221_c0_g2/TRINITY_DN18257_c0_g1/TRINITY_DN15340_c0_g2/TRINITY_DN6620_c0_g2/TRINITY_DN2566_c4_g1/TRINITY_DN32303_c0_g1/TRINITY_DN19322_c0_g1/TRINITY_DN26604_c0_g1/TRINITY_DN47966_c0_g1/TRINITY_DN5659_c	17

							0_g1/TRINITY_DN38209_c0_g1/TRINITY_DN15707_c0_g1	

Table S4. Gene Ontology terms enriched in E-vs-F group.

ID	Description	GeneRatio	BgRatio	*p* Value	*p* Adjust	*q* Value	geneID	Count
GO:0042302	structural constituent of cuticle	68/2626	180/33142	3.39×10^{-29}	9.58×10^{-26}	7.17×10^{-26}	TRINITY_DN2795_c0_g2/TRINITY_DN5184_c1_g1/TRINITY_DN20596_c0_g1/TRINITY_DN7961_c0_g2/TRINITY_DN161_c0_g1/TRINITY_DN6070_c0_g1/TRINITY_DN24_c0_g1/TRINITY_DN5377_c0_g1/TRINITY_DN17428_c0_g1/TRINITY_DN7961_c0_g1/TRINITY_DN915_c0_g2/TRINITY_DN15986_c0_g1/TRINITY_DN9066_c0_g1/TRINITY_DN2870_c0_g1/TRINITY_DN14862_c0_g1/TRINITY_DN10047_c0_g1/TRINITY_DN17634_c0_g1/TRINITY_DN6262_c0_g1/TRINITY_DN9469_c0_g1/TRINITY_DN13386_c0_g1/TRINITY_DN15722_c0_g1/TRINITY_DN1933_c0_g1/TRINITY_DN4167_c1_g1/TRINITY_DN6963_c0_g1/TRINITY_DN38125_c0_g1/TRINITY_DN82200_c0_g1/TRINITY_DN75996_c0_g1/TRINITY_DN2659_c0_g1/TRINITY_DN433_c0_g1/TRINITY_DN13177_c0_g2/	68

TRINITY_DN13904_c0_g1/TRI
NITY_DN3714_c5_g1/TRINIT
Y_DN5509_c0_g1/TRINITY_D
N364_c1_g1/TRINITY_DN257
43_c0_g1/TRINITY_DN25954_
c0_g1/TRINITY_DN35961_c0_
g1/TRINITY_DN32176_c0_g1/
TRINITY_DN74542_c0_g1/TRI
NITY_DN22706_c0_g1/TRINI
TY_DN7380_c0_g1/TRINITY_
DN77377_c0_g1/TRINITY_DN
5920_c0_g1/TRINITY_DN8961
_c0_g2/TRINITY_DN8307_c0_
g2/TRINITY_DN30143_c0_g1/
TRINITY_DN10158_c0_g1/TRI
NITY_DN8961_c0_g1/TRINIT
Y_DN3484_c0_g2/TRINITY_D
N7367_c0_g1/TRINITY_DN22
725_c0_g1/TRINITY_DN62780
_c0_g1/TRINITY_DN19318_c0
_g1/TRINITY_DN915_c0_g1/T
RINITY_DN4613_c0_g1/TRIN
ITY_DN4642_c0_g1/TRINITY_
DN21556_c0_g1/TRINITY_DN
46633_c0_g1/TRINITY_DN838
_c0_g1/TRINITY_DN15897_c0
_g1/TRINITY_DN45404_c0_g1
/TRINITY_DN6480_c0_g1/TRI
NITY_DN27704_c0_g1/TRINI
TY_DN9091_c3_g1/TRINITY_
DN35961_c2_g1/TRINITY_DN
75869_c0_g1/TRINITY_DN303

							21_c0_g1/TRINITY_DN15736_c0_g1	
GO:0031514	motile cilium	46/2626	86/33142	3.51 × 10^{-28}	4.95 × 10^{-25}	3.71 × 10^{-25}	TRINITY_DN6657_c0_g1/TRINITY_DN2841_c0_g1/TRINITY_DN7740_c0_g1/TRINITY_DN3375_c0_g1/TRINITY_DN2752_c0_g1/TRINITY_DN15863_c0_g1/TRINITY_DN4544_c0_g1/TRINITY_DN2729_c0_g1/TRINITY_DN9451_c0_g1/TRINITY_DN7530_c0_g1/TRINITY_DN13410_c0_g1/TRINITY_DN19269_c0_g1/TRINITY_DN6323_c1_g1/TRINITY_DN25521_c0_g1/TRINITY_DN3077_c2_g1/TRINITY_DN11340_c0_g1/TRINITY_DN25774_c0_g1/TRINITY_DN36928_c0_g1/TRINITY_DN38928_c0_g2/TRINITY_DN10076_c0_g1/TRINITY_DN9195_c0_g1/TRINITY_DN1909_c0_g1/TRINITY_DN6380_c0_g1/TRINITY_DN12344_c0_g1/TRINITY_DN3767_c0_g1/TRINITY_DN10356_c0_g1/TRINITY_DN306_c1_g2/TRINITY_DN7652_c0_g1/TRINITY_DN3665_c0_g1/TRINITY_DN8675_c0_g2/TRINITY_DN9168_c0_g2/TRINITY_DN10366_c0_g1/TRINITY_DN10133_c0_g1/TRINITY_DN16784_c0_g1/TRINITY_D	46

							N4533_c4_g1/TRINITY_DN35591_c0_g1/TRINITY_DN5840_c0_g1/TRINITY_DN30782_c0_g2/TRINITY_DN28131_c0_g1/TRINITY_DN59259_c0_g1/TRINITY_DN15794_c0_g1/TRINITY_DN29956_c0_g1/TRINITY_DN3427_c0_g1/TRINITY_DN5578_c0_g1/TRINITY_DN8113_c0_g2/TRINITY_DN63431_c0_g1	
GO:0005929	cilium	53/2626	120/33142	5.67×10^{-27}	5.34×10^{-24}	4.00×10^{-24}	TRINITY_DN6657_c0_g1/TRINITY_DN17778_c0_g1/TRINITY_DN11509_c0_g1/TRINITY_DN6327_c1_g1/TRINITY_DN7196_c0_g1/TRINITY_DN9996_c0_g1/TRINITY_DN7740_c0_g1/TRINITY_DN5006_c0_g2/TRINITY_DN5849_c0_g2/TRINITY_DN30087_c1_g2/TRINITY_DN4774_c0_g1/TRINITY_DN14031_c0_g1/TRINITY_DN13313_c0_g1/TRINITY_DN3071_c0_g1/TRINITY_DN954_c0_g1/TRINITY_DN20653_c0_g1/TRINITY_DN9720_c0_g1/TRINITY_DN36172_c0_g1/TRINITY_DN4043_c0_g1/TRINITY_DN19269_c0_g1/TRINITY_DN1979_c0_g1/TRINITY_DN4002_c0_g3/TRINITY_DN9930_c0_g2/TRINITY_DN3762_c0_g1/TRIN	53

							ITY_DN11340_c0_g1/TRINITY_DN16331_c0_g1/TRINITY_DN7145_c0_g1/TRINITY_DN13406_c0_g1/TRINITY_DN3083_c0_g1/TRINITY_DN7131_c0_g1/TRINITY_DN2415_c2_g1/TRINITY_DN4208_c0_g1/TRINITY_DN5875_c0_g1/TRINITY_DN20653_c0_g4/TRINITY_DN306_c1_g2/TRINITY_DN2153_c0_g1/TRINITY_DN6081_c1_g2/TRINITY_DN11027_c0_g1/TRINITY_DN489_c2_g1/TRINITY_DN3665_c0_g1/TRINITY_DN5968_c0_g1/TRINITY_DN6839_c2_g1/TRINITY_DN9168_c0_g2/TRINITY_DN14798_c0_g1/TRINITY_DN10366_c0_g1/TRINITY_DN7277_c2_g1/TRINITY_DN12092_c0_g3/TRINITY_DN4002_c0_g1/TRINITY_DN6282_c0_g1/TRINITY_DN59259_c0_g1/TRINITY_DN20653_c0_g2/TRINITY_DN26727_c0_g1/TRINITY_DN13780_c0_g1 TRINITY_DN5310_c0_g2/TRINITY_DN21399_c0_g2/TRINITY_DN9170_c1_g1/TRINITY_DN4032_c0_g1/TRINITY_DN32305_c0_g1/TRINITY_DN7371_c0_g1/TRINITY_DN6305_c1_g1/TRINITY_DN2489_c0_g1/T	
GO:0030054	cell junction	81/2626	310/33142	3.01×10^{-22}	2.12×10^{-19}	1.59×10^{-19}		81

insects

MDPI

RINITY_DN11936_c0_g1/TRI
NITY_DN13673_c0_g1/TRINI
TY_DN9219_c0_g1/TRINITY_
DN2738_c0_g1/TRINITY_DN1
120_c0_g2/TRINITY_DN28373
_c0_g1/TRINITY_DN9375_c0_
g1/TRINITY_DN8217_c0_g1/T
RINITY_DN5859_c0_g1/TRIN
ITY_DN7277_c1_g1/TRINITY_
DN27259_c0_g1/TRINITY_DN
7737_c0_g1/TRINITY_DN8355
_c0_g1/TRINITY_DN2161_c0_
g1/TRINITY_DN2207_c0_g1/T
RINITY_DN4958_c0_g1/TRIN
ITY_DN1316_c0_g1/TRINITY_
DN1482_c0_g1/TRINITY_DN4
477_c0_g1/TRINITY_DN2415_
c2_g1/TRINITY_DN740_c0_g1
/TRINITY_DN15308_c0_g2/TR
INITY_DN3363_c0_g1/TRINIT
Y_DN2598_c0_g2/TRINITY_D
N11938_c0_g1/TRINITY_DN1
765_c2_g1/TRINITY_DN1420_
c0_g1/TRINITY_DN2868_c0_g
1/TRINITY_DN10018_c0_g1/T
RINITY_DN3816_c0_g1/TRIN
ITY_DN1614_c0_g1/TRINITY_
DN19635_c0_g1/TRINITY_DN
25715_c0_g1/TRINITY_DN821
7_c1_g1/TRINITY_DN3554_c0
_g1/TRINITY_DN4620_c0_g1/
TRINITY_DN7045_c1_g1/TRI

NITY_DN84830_c0_g1/TRINI
TY_DN35269_c0_g1/TRINITY
_DN19224_c0_g1/TRINITY_D
N13011_c0_g1/TRINITY_DN7
054_c0_g1/TRINITY_DN9935_
c1_g1/TRINITY_DN6093_c0_g
2/TRINITY_DN9041_c0_g1/TR
INITY_DN2120_c1_g1/TRINIT
Y_DN10575_c2_g1/TRINITY_
DN2746_c0_g1/TRINITY_DN2
165_c3_g1/TRINITY_DN1800_
c0_g1/TRINITY_DN12127_c0_
g1/TRINITY_DN898_c0_g1/TR
INITY_DN10400_c0_g1/TRINI
TY_DN1482_c0_g2/TRINITY_
DN2938_c3_g1/TRINITY_DN1
5420_c0_g1/TRINITY_DN1742
2_c0_g2/TRINITY_DN7687_c0
_g1/TRINITY_DN7200_c0_g2/
TRINITY_DN273_c1_g1/TRIN
ITY_DN8569_c0_g1/TRINITY_
DN7523_c0_g1/TRINITY_DN9
041_c0_g2/TRINITY_DN12650
_c0_g1/TRINITY_DN428_c0_g
1/TRINITY_DN4000_c0_g2/TR
INITY_DN7084_c0_g1/TRINIT
Y_DN1908_c0_g1/TRINITY_D
N13354_c0_g1/TRINITY_DN4
674_c0_g1/TRINITY_DN1133_
c1_g2/TRINITY_DN21399_c0_
g1/TRINITY_DN18471_c0_g2

insects MDPI

GO:0060271	cilium assembly	44/2626	112/33142	3.48×10^{-20}	1.97×10^{-17}	1.47×10^{-17}	TRINITY_DN6657_c0_g1/TRINITY_DN11509_c0_g1/TRINITY_DN15240_c0_g1/TRINITY_DN19725_c0_g1/TRINITY_DN11686_c1_g1/TRINITY_DN2051_c0_g1/TRINITY_DN3375_c0_g1/TRINITY_DN5006_c0_g2/TRINITY_DN11896_c0_g2/TRINITY_DN9451_c0_g1/TRINITY_DN42941_c0_g1/TRINITY_DN406_c0_g3/TRINITY_DN5049_c0_g1/TRINITY_DN6610_c0_g1/TRINITY_DN21912_c0_g1/TRINITY_DN19269_c0_g1/TRINITY_DN2999_c0_g1/TRINITY_DN9930_c0_g2/TRINITY_DN3762_c0_g1/TRINITY_DN11340_c0_g1/TRINITY_DN16331_c0_g1/TRINITY_DN13406_c0_g1/TRINITY_DN4988_c2_g1/TRINITY_DN5875_c0_g1/TRINITY_DN18134_c0_g1/TRINITY_DN2725_c0_g1/TRINITY_DN306_c1_g2/TRINITY_DN2153_c0_g1/TRINITY_DN406_c0_g2/TRINITY_DN5914_c0_g1/TRINITY_DN130_c0_g1/TRINITY_DN15988_c0_g2/TRINITY_DN5968_c0_g1/TRINITY_DN4554_c0_g1/TRINITY_DN9168_c0_g2/TRINITY_DN10366_c0_g1/TRINITY_DN2281_	44

GO:0004252	serine-type endopeptidase activity	79/2626	354/33142	2.49 × 10^{-17}	1.17 × 10^{-14}	8.78 × 10^{-15}	c0_g1/TRINITY_DN36845_c0_g1/TRINITY_DN16784_c0_g1/TRINITY_DN7067_c0_g1/TRINITY_DN28131_c0_g1/TRINITY_DN42565_c0_g1/TRINITY_DN16662_c0_g1/TRINITY_DN8113_c0_g2 TRINITY_DN56210_c0_g1/TRINITY_DN6550_c0_g1/TRINITY_DN2384_c0_g1/TRINITY_DN5674_c3_g1/TRINITY_DN7576_c1_g1/TRINITY_DN10564_c0_g1/TRINITY_DN19896_c0_g1/TRINITY_DN6113_c0_g1/TRINITY_DN3388_c0_g1/TRINITY_DN256_c0_g1/TRINITY_DN14789_c0_g1/TRINITY_DN3355_c0_g1/TRINITY_DN9374_c0_g1/TRINITY_DN3133_c1_g2/TRINITY_DN4783_c0_g1/TRINITY_DN11818_c0_g1/TRINITY_DN26373_c0_g1/TRINITY_DN10126_c0_g1/TRINITY_DN12136_c0_g2/TRINITY_DN13505_c0_g1/TRINITY_DN15335_c0_g1/TRINITY_DN4400_c1_g1/TRINITY_DN4711_c0_g2/TRINITY_DN6790_c0_g1/TRINITY_DN9354_c0_g1/TRINITY_DN3503_c0_g1/TRINITY_DN309_c0_g1/TRINITY_DN1350_c0_g2/TRINITY_DN21131_	79

c0_g2/TRINITY_DN12260_c0_
g1/TRINITY_DN11048_c0_g1/
TRINITY_DN23953_c0_g1/TRI
NITY_DN6641_c1_g1/TRINIT
Y_DN31763_c0_g1/TRINITY_
DN19263_c1_g1/TRINITY_DN
8177_c0_g1/TRINITY_DN3312
_c0_g1/TRINITY_DN30190_c0
_g1/TRINITY_DN32456_c0_g2
/TRINITY_DN8587_c0_g1/TRI
NITY_DN6108_c3_g1/TRINIT
Y_DN19398_c0_g2/TRINITY_
DN14570_c0_g1/TRINITY_DN
18051_c0_g1/TRINITY_DN101
59_c1_g1/TRINITY_DN45371_
c0_g1/TRINITY_DN9755_c0_g
1/TRINITY_DN15112_c0_g1/T
RINITY_DN14429_c0_g1/TRI
NITY_DN6641_c0_g2/TRINIT
Y_DN7955_c1_g1/TRINITY_D
N39494_c0_g1/TRINITY_DN7
608_c0_g1/TRINITY_DN1818_
c2_g1/TRINITY_DN2734_c0_g
1/TRINITY_DN5682_c0_g1/TR
INITY_DN800_c0_g2/TRINIT
Y_DN9798_c0_g1/TRINITY_D
N9626_c0_g2/TRINITY_DN66
41_c0_g1/TRINITY_DN76414_
c0_g1/TRINITY_DN9853_c0_g
1/TRINITY_DN20525_c0_g1/T
RINITY_DN7570_c0_g1/TRIN
ITY_DN7189_c1_g1/TRINITY_

							DN45562_c0_g1/TRINITY_DN32069_c0_g1/TRINITY_DN46894_c1_g1/TRINITY_DN13768_c0_g1/TRINITY_DN9924_c0_g1/TRINITY_DN10573_c0_g1/TRINITY_DN1216_c0_g1/TRINITY_DN22425_c0_g1/TRINITY_DN5802_c0_g1/TRINITY_DN1480_c0_g2/TRINITY_DN138_c1_g1/TRINITY_DN2961_c0_g1/TRINITY_DN40263_c0_g1/TRINITY_DN19327_c1_g1	
GO:0005930	axoneme	30/2626	69/33142	1.16×10^{-15}	4.70×10^{-13}	3.52×10^{-13}	TRINITY_DN11509_c0_g1/TRINITY_DN6327_c1_g1/TRINITY_DN15240_c0_g1/TRINITY_DN7740_c0_g1/TRINITY_DN2752_c0_g1/TRINITY_DN9451_c0_g1/TRINITY_DN1979_c0_g2/TRINITY_DN4043_c0_g1/TRINITY_DN3077_c2_g1/TRINITY_DN25774_c0_g1/TRINITY_DN12886_c0_g1/TRINITY_DN7131_c0_g1/TRINITY_DN10076_c0_g1/TRINITY_DN9195_c0_g1/TRINITY_DN18134_c0_g1/TRINITY_DN15113_c0_g1/TRINITY_DN12344_c0_g1/TRINITY_DN306_c1_g2/TRINITY_DN5914_c0_g1/TRINITY_DN5968_c0_g1/TRINITY_DN8872_c0_g1/TRINITY_DN10133_c0_g1/TRINITY_DN16784_c0_g1/T	30

							RINITY_DN5840_c0_g1/TRINITY_DN9647_c0_g1/TRINITY_DN28131_c0_g1/TRINITY_DN59259_c0_g1/TRINITY_DN3427_c0_g1/TRINITY_DN13780_c0_g1/TRINITY_DN63431_c0_g1	
GO:0005874	microtubule	76/2626	361/33142	2.89 × 10^{-15}	1.02 × 10^{-12}	7.64 × 10^{-13}	TRINITY_DN3700_c0_g3/TRINITY_DN3245_c0_g1/TRINITY_DN19161_c0_g1/TRINITY_DN10881_c0_g1/TRINITY_DN19725_c0_g1/TRINITY_DN9561_c0_g1/TRINITY_DN7740_c0_g1/TRINITY_DN4223_c1_g1/TRINITY_DN12450_c0_g2/TRINITY_DN456_c0_g1/TRINITY_DN9451_c0_g1/TRINITY_DN16186_c0_g1/TRINITY_DN5849_c0_g2/TRINITY_DN1979_c0_g2/TRINITY_DN16674_c1_g3/TRINITY_DN2588_c0_g1/TRINITY_DN13313_c0_g1/TRINITY_DN6610_c0_g1/TRINITY_DN16674_c1_g2/TRINITY_DN1265_c0_g1/TRINITY_DN3090_c1_g1/TRINITY_DN4759_c0_g1/TRINITY_DN5173_c0_g1/TRINITY_DN5811_c0_g1/TRINITY_DN552_c0_g1/TRINITY_DN1979_c0_g1/TRINITY_DN4543_c0_g2/TRINITY_DN233_c0_g1/TRINITY_DN3083_c0_g1	76

/TRINITY_DN12886_c0_g1/TR
INITY_DN1979_c1_g1/TRINIT
Y_DN2415_c2_g1/TRINITY_D
N16168_c0_g1/TRINITY_DN4
208_c0_g1/TRINITY_DN3597_
c0_g2/TRINITY_DN18134_c0_
g1/TRINITY_DN1909_c0_g1/T
RINITY_DN2725_c0_g1/TRIN
ITY_DN4474_c0_g1/TRINITY_
DN3767_c0_g1/TRINITY_DN1
0356_c0_g1/TRINITY_DN1513
3_c0_g1/TRINITY_DN489_c2_
g1/TRINITY_DN7652_c0_g1/T
RINITY_DN18791_c0_g1/TRI
NITY_DN2372_c0_g1/TRINIT
Y_DN9716_c0_g1/TRINITY_D
N7469_c2_g1/TRINITY_DN59
68_c0_g1/TRINITY_DN436_c2
_g1/TRINITY_DN953_c0_g1/T
RINITY_DN7691_c1_g1/TRIN
ITY_DN12133_c0_g1/TRINITY
_DN14798_c0_g1/TRINITY_D
N2869_c2_g1/TRINITY_DN10
366_c0_g1/TRINITY_DN15544
_c0_g1/TRINITY_DN26007_c0
_g2/TRINITY_DN1107_c1_g1/
TRINITY_DN35591_c0_g1/TRI
NITY_DN31689_c0_g1/TRINI
TY_DN6282_c0_g1/TRINITY_
DN9647_c0_g1/TRINITY_DN6
818_c0_g2/TRINITY_DN25627
_c0_g1/TRINITY_DN59259_c0

insects

MDPI

							_g1/TRINITY_DN27115_c0_g1/TRINITY_DN12005_c0_g1/TRINITY_DN5922_c0_g1/TRINITY_DN29956_c0_g1/TRINITY_DN5578_c0_g1/TRINITY_DN13780_c0_g1/TRINITY_DN39491_c0_g1/TRINITY_DN15080_c0_g1/TRINITY_DN3383_c0_g1/TRINITY_DN18471_c0_g2	
GO:0004867	serine-type endopeptidase inhibitor activity	30/2626	72/33142	4.78×10^{-15}	1.50×10^{-12}	1.12×10^{-12}	TRINITY_DN398_c0_g1/TRINITY_DN7571_c0_g1/TRINITY_DN20619_c0_g1/TRINITY_DN5613_c1_g1/TRINITY_DN6082_c1_g2/TRINITY_DN16500_c1_g1/TRINITY_DN4861_c0_g1/TRINITY_DN5323_c0_g1/TRINITY_DN3123_c0_g1/TRINITY_DN2738_c0_g1/TRINITY_DN1880_c2_g2/TRINITY_DN3923_c0_g1/TRINITY_DN14227_c0_g1/TRINITY_DN13407_c0_g1/TRINITY_DN81482_c0_g2/TRINITY_DN1880_c2_g1/TRINITY_DN24544_c0_g1/TRINITY_DN4603_c1_g1/TRINITY_DN1510_c0_g1/TRINITY_DN29610_c0_g1/TRINITY_DN636_c0_g1/TRINITY_DN1696_c0_g3/TRINITY_DN44040_c0_g1/TRINITY_DN18049_c0_g1/TRINITY_DN10648_c0_g1/TRINITY_DN3717_c0_g1/TRINITY_	30

							DN7326_c0_g1/TRINITY_DN56782_c0_g1/TRINITY_DN20754_c0_g1/TRINITY_DN14093_c0_g1	
GO:0030992	intraciliary transport particle B	14/2626	16/33142	3.83 × 10^{-14}	1.08 × 10^{-11}	8.11 × 10^{-12}	TRINITY_DN11509_c0_g1/TRINITY_DN6327_c1_g1/TRINITY_DN7196_c0_g1/TRINITY_DN5006_c0_g2/TRINITY_DN19269_c0_g1/TRINITY_DN4002_c0_g3/TRINITY_DN9930_c0_g2/TRINITY_DN11340_c0_g1/TRINITY_DN7145_c0_g1/TRINITY_DN13406_c0_g1/TRINITY_DN306_c1_g2/TRINITY_DN2153_c0_g1/TRINITY_DN9168_c0_g2/TRINITY_DN4002_c0_g1	14
GO:0045202	synapse	51/2626	204/33142	1.01 × 10^{-13}	2.60 × 10^{-11}	1.95 × 10^{-11}	TRINITY_DN21399_c0_g2/TRINITY_DN32305_c0_g1/TRINITY_DN7371_c0_g1/TRINITY_DN3245_c0_g1/TRINITY_DN1487_c0_g2/TRINITY_DN2489_c0_g1/TRINITY_DN13673_c0_g1/TRINITY_DN6174_c0_g1/TRINITY_DN9219_c0_g1/TRINITY_DN2738_c0_g1/TRINITY_DN47_c0_g1/TRINITY_DN9375_c0_g1/TRINITY_DN7277_c1_g1/TRINITY_DN2207_c0_g1/TRINITY_DN4958_c0_g1/TRINITY_DN10760_c0_g1/TRINITY_DN9333_c0_g1/TRINITY_	51

							DN2415_c2_g1/TRINITY_DN740_c0_g1/TRINITY_DN15308_c0_g2/TRINITY_DN480_c0_g1/TRINITY_DN2598_c0_g2/TRINITY_DN1420_c0_g1/TRINITY_DN10018_c0_g1/TRINITY_DN3816_c0_g1/TRINITY_DN8217_c1_g1/TRINITY_DN3554_c0_g1/TRINITY_DN11154_c0_g1/TRINITY_DN19224_c0_g1/TRINITY_DN13011_c0_g1/TRINITY_DN2120_c1_g1/TRINITY_DN2746_c0_g1/TRINITY_DN19691_c1_g1/TRINITY_DN2938_c3_g1/TRINITY_DN55372_c0_g1/TRINITY_DN15420_c0_g1/TRINITY_DN17422_c0_g2/TRINITY_DN7687_c0_g1/TRINITY_DN5274_c0_g1/TRINITY_DN7200_c0_g2/TRINITY_DN11061_c0_g2/TRINITY_DN7523_c0_g1/TRINITY_DN44537_c0_g1/TRINITY_DN16058_c0_g1/TRINITY_DN52030_c0_g1/TRINITY_DN4000_c0_g2/TRINITY_DN7084_c0_g1/TRINITY_DN1908_c0_g1/TRINITY_DN2961_c0_g1/TRINITY_DN13354_c0_g1/TRINITY_DN1133_c1_g2	
GO:0043231	intracellular membrane-bounded organelle	65/2626	316/33142	8.81×10^{-13}	2.07×10^{-10}	1.55×10^{-10}	TRINITY_DN7814_c0_g2/TRINITY_DN32009_c0_g1/TRINI	65

TY_DN16600_c0_g1/TRINITY
_DN7371_c0_g1/TRINITY_DN
72971_c0_g1/TRINITY_DN645
5_c0_g1/TRINITY_DN8493_c0
_g1/TRINITY_DN2078_c0_g1/
TRINITY_DN43642_c0_g1/TRI
NITY_DN16979_c0_g1/TRINI
TY_DN77269_c0_g1/TRINITY
_DN11954_c0_g1/TRINITY_D
N5216_c1_g1/TRINITY_DN28
48_c1_g1/TRINITY_DN5364_c
0_g1/TRINITY_DN7048_c0_g1
/TRINITY_DN5006_c0_g2/TRI
NITY_DN5086_c0_g1/TRINIT
Y_DN2476_c1_g1/TRINITY_D
N23485_c0_g1/TRINITY_DN1
6861_c0_g1/TRINITY_DN2557
_c0_g2/TRINITY_DN27271_c0
_g1/TRINITY_DN23263_c0_g1
/TRINITY_DN13963_c0_g1/TR
INITY_DN15487_c0_g1/TRINI
TY_DN5170_c0_g1/TRINITY_
DN4826_c1_g1/TRINITY_DN5
087_c0_g1/TRINITY_DN8718_
c0_g1/TRINITY_DN10011_c0_
g1/TRINITY_DN17565_c0_g1/
TRINITY_DN28084_c0_g1/TRI
NITY_DN4160_c0_g1/TRINIT
Y_DN10583_c0_g1/TRINITY_
DN9492_c0_g1/TRINITY_DN3
614_c1_g1/TRINITY_DN271_c
0_g1/TRINITY_DN974_c0_g1/

insects

							TRINITY_DN9492_c0_g2/TRINITY_DN2095_c0_g1/TRINITY_DN24147_c0_g1/TRINITY_DN7045_c1_g1/TRINITY_DN8284_c0_g1/TRINITY_DN656_c0_g1/TRINITY_DN9853_c0_g1/TRINITY_DN6603_c0_g1/TRINITY_DN20754_c0_g1/TRINITY_DN15324_c1_g1/TRINITY_DN13235_c0_g1/TRINITY_DN898_c0_g1/TRINITY_DN7713_c1_g1/TRINITY_DN13226_c0_g1/TRINITY_DN9897_c0_g1/TRINITY_DN1200_c2_g1/TRINITY_DN9937_c0_g1/TRINITY_DN2006_c1_g2/TRINITY_DN12650_c0_g1/TRINITY_DN901_c0_g1/TRINITY_DN11447_c0_g1/TRINITY_DN43641_c0_g1/TRINITY_DN333_c0_g1/TRINITY_DN6505_c0_g1/TRINITY_DN579_c0_g1/TRINITY_DN901_c0_g2	
GO:0009986	cell surface	52/2626	227/33142	2.30×10^{-12}	4.89×10^{-10}	3.66×10^{-10}	TRINITY_DN21789_c0_g1/TRINITY_DN5056_c0_g1/TRINITY_DN9277_c0_g1/TRINITY_DN2398_c2_g1/TRINITY_DN1949_c0_g1/TRINITY_DN22536_c0_g1/TRINITY_DN18454_c0_g1/TRINITY_DN9219_c0_g1/TRINITY_DN1568_c0_g1/TRINITY_DN2738_c0_g1/TRINITY_	52

insects

DN5364_c0_g1/TRINITY_DN7
048_c0_g1/TRINITY_DN15486
_c0_g2/TRINITY_DN4514_c0_
g1/TRINITY_DN7743_c0_g1/T
RINITY_DN5086_c0_g1/TRIN
ITY_DN14877_c1_g1/TRINITY
_DN9375_c0_g1/TRINITY_DN
8257_c0_g1/TRINITY_DN6108
_c0_g1/TRINITY_DN722_c1_g
1/TRINITY_DN740_c0_g1/TRI
NITY_DN15308_c0_g2/TRINI
TY_DN1902_c0_g1/TRINITY_
DN4441_c3_g1/TRINITY_DN2
598_c0_g2/TRINITY_DN1614_
c0_g1/TRINITY_DN13726_c0_
g1/TRINITY_DN8278_c2_g1/T
RINITY_DN7045_c1_g1/TRIN
ITY_DN10569_c0_g2/TRINITY
_DN2292_c2_g1/TRINITY_DN
9780_c0_g1/TRINITY_DN2075
4_c0_g1/TRINITY_DN19100_c
0_g1/TRINITY_DN77356_c0_g
1/TRINITY_DN9158_c3_g1/TR
INITY_DN26376_c0_g1/TRINI
TY_DN9521_c0_g1/TRINITY_
DN709_c0_g1/TRINITY_DN76
87_c0_g1/TRINITY_DN5274_c
0_g1/TRINITY_DN3902_c0_g1
/TRINITY_DN273_c1_g1/TRI
NITY_DN23609_c0_g1/TRINI
TY_DN7523_c0_g1/TRINITY_
DN12958_c0_g1/TRINITY_DN

insects MDPI

							1480_c0_g2/TRINITY_DN333_c0_g1/TRINITY_DN42475_c0_g1/TRINITY_DN13354_c0_g1/TRINITY_DN14134_c0_g1	
GO:0004930	G protein-coupled receptor activity	40/2626	147/33142	2.49×10^{-12}	4.89×10^{-10}	3.66×10^{-10}	TRINITY_DN2217_c0_g1/TRINITY_DN14379_c0_g1/TRINITY_DN2244_c1_g1/TRINITY_DN39639_c3_g1/TRINITY_DN938_c2_g1/TRINITY_DN7051_c0_g1/TRINITY_DN10577_c0_g1/TRINITY_DN36816_c0_g1/TRINITY_DN1709_c0_g1/TRINITY_DN17903_c0_g1/TRINITY_DN18021_c0_g2/TRINITY_DN41433_c0_g1/TRINITY_DN1451_c0_g2/TRINITY_DN19321_c0_g1/TRINITY_DN22381_c0_g1/TRINITY_DN2601_c0_g1/TRINITY_DN10201_c0_g1/TRINITY_DN1486_c0_g1/TRINITY_DN27368_c0_g1/TRINITY_DN1451_c0_g1/TRINITY_DN31435_c0_g1/TRINITY_DN43399_c0_g1/TRINITY_DN84830_c0_g1/TRINITY_DN33474_c0_g1/TRINITY_DN27045_c0_g1/TRINITY_DN9642_c1_g2/TRINITY_DN28222_c0_g1/TRINITY_DN58254_c0_g1/TRINITY_DN3032_c1_g2/TRINITY_DN446_c1_g1/TRINITY_DN874_c0_g1/TRINITY_DN3743_c1	40

							_g1/TRINITY_DN11648_c0_g1/TRINITY_DN1001_c0_g1/TRINITY_DN48880_c0_g1/TRINITY_DN58043_c0_g1/TRINITY_DN15502_c0_g1/TRINITY_DN2285_c0_g1/TRINITY_DN864_c0_g3/TRINITY_DN14134_c0_g1	
GO:0036126	sperm flagellum	19/2626	36/33142	2.60×10^{-12}	4.89×10^{-10}	3.66×10^{-10}	TRINITY_DN19725_c0_g1/TRINITY_DN11686_c1_g1/TRINITY_DN7740_c0_g1/TRINITY_DN15863_c0_g1/TRINITY_DN4544_c0_g1/TRINITY_DN1979_c0_g2/TRINITY_DN6610_c0_g1/TRINITY_DN36928_c0_g1/TRINITY_DN6380_c0_g1/TRINITY_DN2725_c0_g1/TRINITY_DN12344_c0_g1/TRINITY_DN306_c1_g2/TRINITY_DN5968_c0_g1/TRINITY_DN2331_c0_g1/TRINITY_DN9168_c0_g2/TRINITY_DN10366_c0_g1/TRINITY_DN7067_c0_g1/TRINITY_DN59259_c0_g1/TRINITY_DN16662_c0_g1	19
GO:0007186	G protein-coupled receptor signaling pathway	46/2626	191/33142	6.61×10^{-12}	1.17×10^{-9}	8.73×10^{-10}	TRINITY_DN72971_c0_g1/TRINITY_DN2217_c0_g1/TRINITY_DN14379_c0_g1/TRINITY_DN39639_c3_g1/TRINITY_DN938_c2_g1/TRINITY_DN834_c1_g1/TRINITY_DN7051_c0_g1/TRINITY_DN10577_c0_g1/TR	46

insects

INITY_DN36816_c0_g1/TRINI
TY_DN7048_c0_g1/TRINITY_
DN1709_c0_g1/TRINITY_DN2
0653_c0_g1/TRINITY_DN1790
3_c0_g1/TRINITY_DN18021_c
0_g2/TRINITY_DN41433_c0_g
1/TRINITY_DN4826_c1_g1/TR
INITY_DN1229_c2_g1/TRINIT
Y_DN19321_c0_g1/TRINITY_
DN22381_c0_g1/TRINITY_DN
2601_c0_g1/TRINITY_DN1020
1_c0_g1/TRINITY_DN1486_c0
_g1/TRINITY_DN27368_c0_g1
/TRINITY_DN31435_c0_g1/TR
INITY_DN84830_c0_g1/TRINI
TY_DN33474_c0_g1/TRINITY
_DN5120_c0_g1/TRINITY_DN
9642_c1_g2/TRINITY_DN2822
2_c0_g1/TRINITY_DN6839_c2
_g1/TRINITY_DN58254_c0_g1
/TRINITY_DN6910_c0_g1/TRI
NITY_DN3032_c1_g2/TRINIT
Y_DN446_c1_g1/TRINITY_D
N874_c0_g1/TRINITY_DN374
3_c1_g1/TRINITY_DN11648_c
0_g1/TRINITY_DN1001_c0_g1
/TRINITY_DN48880_c0_g1/TR
INITY_DN58043_c0_g1/TRINI
TY_DN12650_c0_g1/TRINITY
_DN15502_c0_g1/TRINITY_D
N10344_c0_g1/TRINITY_DN2
285_c0_g1/TRINITY_DN864_c

							0_g3/TRINITY_DN14134_c0_g1	
GO:0016324	apical plasma membrane	45/2626	185/33142	7.74 × 10^{-12}	1.29 × 10^{-9}	9.63 × 10^{-10}	TRINITY_DN7437_c0_g1/TRINITY_DN1748_c0_g1/TRINITY_DN1990_c0_g1/TRINITY_DN11631_c0_g3/TRINITY_DN47_c0_g1/TRINITY_DN4514_c0_g1/TRINITY_DN388_c0_g1/TRINITY_DN7743_c0_g1/TRINITY_DN14877_c1_g1/TRINITY_DN1709_c0_g1/TRINITY_DN7737_c0_g1/TRINITY_DN20881_c0_g2/TRINITY_DN3305_c0_g1/TRINITY_DN9333_c0_g1/TRINITY_DN480_c0_g1/TRINITY_DN1902_c0_g1/TRINITY_DN3363_c0_g1/TRINITY_DN1852_c2_g1/TRINITY_DN6810_c0_g1/TRINITY_DN1773_c1_g2/TRINITY_DN7880_c0_g1/TRINITY_DN7045_c1_g1/TRINITY_DN5795_c3_g1/TRINITY_DN6705_c0_g2/TRINITY_DN93_c0_g1/TRINITY_DN6365_c0_g1/TRINITY_DN19729_c0_g3/TRINITY_DN22905_c0_g1/TRINITY_DN1060_c1_g1/TRINITY_DN11886_c0_g1/TRINITY_DN1384_c0_g1/TRINITY_DN9521_c0_g1/TRINITY_DN8644_c0_g1/TRINITY_DN8569_c0_g1/TRINITY_DN10063_c0_g1/T	45

							RINITY_DN4384_c0_g1/TRINITY_DN216_c0_g3/TRINITY_DN58043_c0_g1/TRINITY_DN1941_c0_g1/TRINITY_DN6505_c0_g1/TRINITY_DN10346_c1_g1/TRINITY_DN7471_c0_g1/TRINITY_DN18007_c0_g1/TRINITY_DN17100_c0_g1/TRINITY_DN1119_c0_g1	
GO:0060285	cilium-dependent cell motility	14/2626	20/33142	9.13×10^{-12}	1.43×10^{-9}	1.07×10^{-9}	TRINITY_DN9451_c0_g1/TRINITY_DN1979_c0_g1/TRINITY_DN3077_c2_g1/TRINITY_DN4543_c0_g2/TRINITY_DN1197_c0_g1/TRINITY_DN3083_c0_g1/TRINITY_DN10076_c0_g1/TRINITY_DN8872_c0_g1/TRINITY_DN10133_c0_g1/TRINITY_DN16784_c0_g1/TRINITY_DN31689_c0_g1/TRINITY_DN9647_c0_g1/TRINITY_DN28131_c0_g1/TRINITY_DN3427_c0_g1	14
GO:0070062	extracellular exosome	67/2626	350/33142	1.22×10^{-11}	1.82×10^{-9}	1.36×10^{-9}	TRINITY_DN3991_c0_g1/TRINITY_DN7814_c0_g2/TRINITY_DN7437_c0_g1/TRINITY_DN72971_c0_g1/TRINITY_DN8746_c0_g3/TRINITY_DN2078_c0_g1/TRINITY_DN205_c0_g1/TRINITY_DN5613_c1_g1/TRINITY_DN11686_c1_g1/TRINITY_DN5886_c0_g1/TRINITY_DN5826_c0_g1/TRINITY_DN3	67

insects

MDPI

923_c0_g1/TRINITY_DN15748
_c0_g1/TRINITY_DN1120_c0_
g2/TRINITY_DN12607_c0_g1/
TRINITY_DN200_c0_g2/TRIN
ITY_DN8257_c0_g1/TRINITY_
DN2898_c0_g1/TRINITY_DN1
0137_c0_g1/TRINITY_DN306_
c2_g1/TRINITY_DN4826_c1_g
1/TRINITY_DN1338_c0_g1/TR
INITY_DN9333_c0_g1/TRINIT
Y_DN17565_c0_g1/TRINITY_
DN755_c1_g1/TRINITY_DN19
02_c0_g1/TRINITY_DN5262_c
1_g1/TRINITY_DN1610_c0_g1
/TRINITY_DN7955_c1_g1/TRI
NITY_DN2682_c0_g1/TRINIT
Y_DN1178_c2_g1/TRINITY_D
N271_c0_g1/TRINITY_DN131
38_c0_g1/TRINITY_DN12134_
c0_g1/TRINITY_DN10569_c0_
g2/TRINITY_DN8625_c0_g1/T
RINITY_DN19224_c0_g1/TRI
NITY_DN7326_c0_g1/TRINIT
Y_DN656_c0_g1/TRINITY_D
N56782_c0_g1/TRINITY_DN1
5289_c0_g1/TRINITY_DN9780
_c0_g1/TRINITY_DN16751_c0
_g1/TRINITY_DN953_c0_g1/T
RINITY_DN20484_c0_g3/TRI
NITY_DN6745_c0_g1/TRINIT
Y_DN4554_c0_g1/TRINITY_D
N11144_c0_g1/TRINITY_DN4

insects MDPI

							858_c1_g1/TRINITY_DN22607_c0_g1/TRINITY_DN2281_c0_g1/TRINITY_DN19600_c0_g1/TRINITY_DN4326_c0_g1/TRINITY_DN5274_c0_g1/TRINITY_DN5431_c0_g1/TRINITY_DN5956_c0_g2/TRINITY_DN216_c0_g3/TRINITY_DN12958_c0_g1/TRINITY_DN4839_c0_g1/TRINITY_DN39318_c0_g1/TRINITY_DN40189_c0_g1/TRINITY_DN12338_c0_g1/TRINITY_DN51_c0_g1/TRINITY_DN11599_c0_g1/TRINITY_DN6300_c0_g1/TRINITY_DN6541_c1_g2/TRINITY_DN4487_c3_g2	
GO:0045087	innate immune response	42/2626	170/33142	2.22×10^{-11}	3.14×10^{-9}	2.35×10^{-9}	TRINITY_DN33530_c0_g1/TRINITY_DN15601_c0_g3/TRINITY_DN945_c0_g1/TRINITY_DN6944_c0_g1/TRINITY_DN1893_c0_g1/TRINITY_DN6113_c0_g1/TRINITY_DN6481_c0_g1/TRINITY_DN3487_c1_g1/TRINITY_DN10831_c0_g1/TRINITY_DN11325_c0_g1/TRINITY_DN6931_c0_g1/TRINITY_DN38512_c0_g1/TRINITY_DN22536_c0_g1/TRINITY_DN402_c1_g1/TRINITY_DN972_c0_g1/TRINITY_DN8352_c0_g1/TRINITY_DN4514_c0_g1/TRINITY_DN15618_c0_g1/TRINITY_DN	42

insects

MDPI

							9354_c0_g1/TRINITY_DN4097_c0_g1/TRINITY_DN5176_c0_g1/TRINITY_DN5049_c0_g1/TRINITY_DN4768_c0_g1/TRINITY_DN19263_c1_g1/TRINITY_DN19246_c0_g1/TRINITY_DN306_c2_g1/TRINITY_DN1338_c0_g1/TRINITY_DN14570_c0_g1/TRINITY_DN479_c0_g2/TRINITY_DN25583_c0_g1/TRINITY_DN16383_c1_g1/TRINITY_DN755_c1_g1/TRINITY_DN6677_c0_g1/TRINITY_DN9798_c0_g1/TRINITY_DN13662_c0_g1/TRINITY_DN2292_c2_g1/TRINITY_DN15618_c0_g2/TRINITY_DN7189_c1_g1/TRINITY_DN8043_c0_g1/TRINITY_DN7944_c0_g1/TRINITY_DN8352_c0_g2/TRINITY_DN1439_c0_g1	
GO:0003351	epithelial cilium movement	13/2626	18/33142	2.77×10^{-11}	3.72×10^{-9}	2.78×10^{-9}	TRINITY_DN6657_c0_g1/TRINITY_DN6327_c1_g1/TRINITY_DN7740_c0_g1/TRINITY_DN1979_c0_g2/TRINITY_DN7530_c0_g1/TRINITY_DN16168_c0_g1/TRINITY_DN7652_c0_g1/TRINITY_DN3665_c0_g1/TRINITY_DN15544_c0_g1/TRINITY_DN3299_c0_g1/TRINITY_DN7067_c0_g1/TRINITY_DN5	13

							9259_c0_g1/TRINITY_DN26727_c0_g1	
GO:0005813	centrosome	44/2626	187/33142	4.29×10^{-11}	5.51×10^{-9}	4.13×10^{-9}	TRINITY_DN13157_c0_g1/TRINITY_DN6327_c1_g1/TRINITY_DN11936_c0_g1/TRINITY_DN6174_c0_g1/TRINITY_DN12450_c0_g2/TRINITY_DN5006_c0_g2/TRINITY_DN16186_c0_g1/TRINITY_DN2397_c0_g1/TRINITY_DN3071_c0_g1/TRINITY_DN3296_c0_g1/TRINITY_DN5173_c0_g1/TRINITY_DN19269_c0_g1/TRINITY_DN552_c0_g1/TRINITY_DN7556_c0_g1/TRINITY_DN4002_c0_g3/TRINITY_DN4644_c0_g1/TRINITY_DN9930_c0_g2/TRINITY_DN3762_c0_g1/TRINITY_DN11340_c0_g1/TRINITY_DN7145_c0_g1/TRINITY_DN233_c0_g1/TRINITY_DN13406_c0_g1/TRINITY_DN12886_c0_g1/TRINITY_DN7131_c0_g1/TRINITY_DN4474_c0_g1/TRINITY_DN306_c1_g2/TRINITY_DN2153_c0_g1/TRINITY_DN489_c2_g1/TRINITY_DN1117_c1_g1/TRINITY_DN2107_c2_g1/TRINITY_DN93_c0_g1/TRINITY_DN5312_c0_g1/TRINITY_DN9168_c0_g2/TRINITY_DN3299_c0_g1/TRINITY_DN1107_c1_g	44

							1/TRINITY_DN4002_c0_g1/TRINITY_DN2006_c1_g2/TRINITY_DN3249_c0_g1/TRINITY_DN637_c0_g1/TRINITY_DN25627_c0_g1/TRINITY_DN23722_c0_g1/TRINITY_DN1908_c0_g1/TRINITY_DN3810_c0_g1/TRINITY_DN16662_c0_g1	
GO:0030424	axon	50/2626	241/33142	2.64×10^{-10}	3.24×10^{-8}	2.42×10^{-8}	TRINITY_DN7437_c0_g1/TRINITY_DN21399_c0_g2/TRINITY_DN32305_c0_g1/TRINITY_DN8323_c0_g1/TRINITY_DN16600_c0_g1/TRINITY_DN7371_c0_g1/TRINITY_DN3446_c0_g1/TRINITY_DN7807_c0_g1/TRINITY_DN3245_c0_g1/TRINITY_DN16444_c1_g1/TRINITY_DN1487_c0_g2/TRINITY_DN402_c1_g1/TRINITY_DN11100_c0_g1/TRINITY_DN16186_c0_g1/TRINITY_DN2868_c0_g2/TRINITY_DN13394_c0_g1/TRINITY_DN4811_c0_g1/TRINITY_DN6076_c0_g1/TRINITY_DN8355_c0_g1/TRINITY_DN2531_c0_g1/TRINITY_DN233_c0_g1/TRINITY_DN8012_c0_g1/TRINITY_DN10018_c0_g1/TRINITY_DN3816_c0_g1/TRINITY_DN8217_c1_g1/TRINITY_DN800_c0_g2/TRINITY_DN130_c0_g1/TRINITY_DN462	50

							0_c0_g1/TRINITY_DN3665_c0_g1/TRINITY_DN1035_c0_g3/TRINITY_DN10569_c0_g2/TRINITY_DN7898_c0_g1/TRINITY_DN35269_c0_g1/TRINITY_DN8599_c1_g1/TRINITY_DN2475_c0_g1/TRINITY_DN12452_c0_g1/TRINITY_DN6745_c0_g1/TRINITY_DN2746_c0_g1/TRINITY_DN2165_c3_g1/TRINITY_DN13362_c0_g1/TRINITY_DN9158_c3_g1/TRINITY_DN14953_c0_g3/TRINITY_DN709_c0_g1/TRINITY_DN7200_c0_g2/TRINITY_DN14663_c0_g1/TRINITY_DN16530_c0_g1/TRINITY_DN28241_c0_g1/TRINITY_DN52030_c0_g1/TRINITY_DN13354_c0_g1/TRINITY_DN49433_c0_g1	
GO:0003341	cilium movement	17/2626	36/33142	3.62×10^{-10}	4.26×10^{-8}	3.19×10^{-8}	TRINITY_DN9451_c0_g1/TRINITY_DN1979_c0_g1/TRINITY_DN3083_c0_g1/TRINITY_DN1979_c1_g1/TRINITY_DN9195_c0_g1/TRINITY_DN18134_c0_g1/TRINITY_DN9676_c0_g1/TRINITY_DN15113_c0_g1/TRINITY_DN5914_c0_g1/TRINITY_DN5968_c0_g1/TRINITY_DN8872_c0_g1/TRINITY_DN14798_c0_g1/TRINITY_DN3299_c0_g1/TRINITY_DN4533_c4_	17

insects MDPI

							g1/TRINITY_DN31689_c0_g1/TRINITY_DN5840_c0_g1/TRINITY_DN63431_c0_g1	
GO:0043204	perikaryon	25/2626	77/33142	5.29 × 10^{-10}	5.97 × 10^{-8}	4.47 × 10^{-8}	TRINITY_DN21399_c0_g2/TRINITY_DN8323_c0_g1/TRINITY_DN3446_c0_g1/TRINITY_DN7807_c0_g1/TRINITY_DN402_c1_g1/TRINITY_DN15486_c0_g2/TRINITY_DN13394_c0_g1/TRINITY_DN4811_c0_g1/TRINITY_DN2557_c0_g2/TRINITY_DN2786_c0_g1/TRINITY_DN8355_c0_g1/TRINITY_DN233_c0_g1/TRINITY_DN8012_c0_g1/TRINITY_DN1765_c2_g1/TRINITY_DN3816_c0_g1/TRINITY_DN10569_c0_g2/TRINITY_DN13011_c0_g1/TRINITY_DN9041_c0_g1/TRINITY_DN716_c0_g1/TRINITY_DN7200_c0_g2/TRINITY_DN9041_c0_g2/TRINITY_DN4000_c0_g2/TRINITY_DN4784_c1_g1/TRINITY_DN16754_c0_g1/TRINITY_DN18471_c0_g2	25
GO:0050804	modulation of chemical synaptic transmission	20/2626	51/33142	6.21 × 10^{-10}	6.74 × 10^{-8}	5.05 × 10^{-8}	TRINITY_DN21399_c0_g2/TRINITY_DN16006_c0_g1/TRINITY_DN30262_c0_g1/TRINITY_DN4329_c1_g1/TRINITY_DN9375_c0_g1/TRINITY_DN18321_c0_g1/TRINITY_DN8355_c0_g1/TRINITY_DN15308_c0_g2	20

							/TRINITY_DN1765_c2_g1/TRINITY_DN3816_c0_g1/TRINITY_DN9041_c0_g1/TRINITY_DN4029_c0_g1/TRINITY_DN15420_c0_g1/TRINITY_DN7687_c0_g1/TRINITY_DN5274_c0_g1/TRINITY_DN7200_c0_g2/TRINITY_DN7523_c0_g1/TRINITY_DN9041_c0_g2/TRINITY_DN49433_c0_g1/TRINITY_DN21399_c0_g1	
GO:0007268	chemical synaptic transmission	33/2626	127/33142	7.38×10^{-10}	7.72×10^{-8}	5.78×10^{-8}	TRINITY_DN21399_c0_g2/TRINITY_DN4032_c0_g1/TRINITY_DN2639_c0_g1/TRINITY_DN1748_c0_g1/TRINITY_DN2738_c0_g1/TRINITY_DN5470_c0_g1/TRINITY_DN11896_c0_g2/TRINITY_DN5859_c0_g1/TRINITY_DN4958_c0_g1/TRINITY_DN740_c0_g1/TRINITY_DN17565_c0_g1/TRINITY_DN19321_c0_g1/TRINITY_DN2598_c0_g2/TRINITY_DN11938_c0_g1/TRINITY_DN1765_c2_g1/TRINITY_DN1267_c0_g2/TRINITY_DN3816_c0_g1/TRINITY_DN84830_c0_g1/TRINITY_DN11846_c0_g1/TRINITY_DN13011_c0_g1/TRINITY_DN9041_c0_g1/TRINITY_DN2165_c3_g1/TRINITY_DN716_c0_g1/TRINITY_DN2938_c3_g1/TRIN	33

GO:0031090	organelle membrane	46/2626	220/33142	1.03 × 10^{-9}	1.04 × 10^{-7}	7.79 × 10^{-8}	ITY_DN15420_c0_g1/TRINITY_DN7200_c0_g2/TRINITY_DN11061_c0_g2/TRINITY_DN9041_c0_g2/TRINITY_DN7318_c0_g1/TRINITY_DN4000_c0_g2/TRINITY_DN29720_c0_g2/TRINITY_DN21399_c0_g1/TRINITY_DN8692_c0_g1 TRINITY_DN788_c0_g2/TRINITY_DN12459_c0_g1/TRINITY_DN510_c0_g1/TRINITY_DN14155_c0_g1/TRINITY_DN8337_c0_g3/TRINITY_DN16979_c0_g1/TRINITY_DN14638_c0_g1/TRINITY_DN2034_c0_g1/TRINITY_DN9929_c0_g1/TRINITY_DN80619_c0_g2/TRINITY_DN16674_c1_g3/TRINITY_DN16674_c1_g2/TRINITY_DN9323_c0_g2/TRINITY_DN23485_c0_g1/TRINITY_DN6729_c0_g2/TRINITY_DN6729_c0_g3/TRINITY_DN3648_c0_g6/TRINITY_DN8718_c0_g1/TRINITY_DN4475_c0_g2/TRINITY_DN10011_c0_g1/TRINITY_DN28084_c0_g1/TRINITY_DN6010_c3_g1/TRINITY_DN7771_c0_g2/TRINITY_DN10533_c0_g1/TRINITY_DN10583_c0_g1/TRINITY_DN996_c0_g1/TRINITY_DN5213_c0_g1/TRINITY_DN31	46

insects

MDPI

GO:0042734	presynaptic membrane	19/2626	48/33142	1.38 × 10^{-9}	1.34 × 10^{-7}	1.00 × 10^{-7}	28_c0_g2/TRINITY_DN8567_c0_g1/TRINITY_DN25933_c0_g2/TRINITY_DN8705_c0_g1/TRINITY_DN13226_c0_g1/TRINITY_DN21490_c0_g1/TRINITY_DN10422_c0_g1/TRINITY_DN6985_c0_g1/TRINITY_DN3753_c0_g1/TRINITY_DN9897_c0_g1/TRINITY_DN5625_c1_g1/TRINITY_DN13697_c0_g1/TRINITY_DN4037_c0_g1/TRINITY_DN23688_c0_g1/TRINITY_DN33814_c0_g1/TRINITY_DN8845_c0_g3/TRINITY_DN8029_c0_g2/TRINITY_DN7224_c1_g3/TRINITY_DN25846_c0_g2TRINITY_DN21399_c0_g2/TRINITY_DN18454_c0_g1/TRINITY_DN9375_c0_g1/TRINITY_DN8355_c0_g1/TRINITY_DN2161_c0_g1/TRINITY_DN1482_c0_g1/TRINITY_DN11938_c0_g1/TRINITY_DN1765_c2_g1/TRINITY_DN1420_c0_g1/TRINITY_DN3816_c0_g1/TRINITY_DN4620_c0_g1/TRINITY_DN9041_c0_g1/TRINITY_DN1482_c0_g2/TRINITY_DN7200_c0_g2/TRINITY_DN9041_c0_g2/TRINITY_DN12650_c0_g1/TRINITY_DN10344_c0_g1/TRINITY	19

							_DN49433_c0_g1/TRINITY_DN21399_c0_g1	
GO:0043005	neuron projection	39/2626	173/33142	2.00 × 10^{-9}	1.88 × 10^{-7}	1.41 × 10^{-7}	TRINITY_DN4032_c0_g1/TRINITY_DN1487_c0_g2/TRINITY_DN18454_c0_g1/TRINITY_DN954_c0_g1/TRINITY_DN1421_c0_g1/TRINITY_DN2557_c0_g2/TRINITY_DN5859_c0_g1/TRINITY_DN27259_c0_g1/TRINITY_DN31967_c0_g1/TRINITY_DN233_c0_g1/TRINITY_DN4958_c0_g1/TRINITY_DN15030_c0_g2/TRINITY_DN30182_c0_g1/TRINITY_DN11938_c0_g1/TRINITY_DN12463_c0_g1/TRINITY_DN130_c0_g1/TRINITY_DN84830_c0_g1/TRINITY_DN13011_c0_g1/TRINITY_DN93_c0_g1/TRINITY_DN2120_c1_g1/TRINITY_DN716_c0_g1/TRINITY_DN58254_c0_g1/TRINITY_DN19691_c1_g1/TRINITY_DN2938_c3_g1/TRINITY_DN273_c1_g1/TRINITY_DN11061_c0_g2/TRINITY_DN12650_c0_g1/TRINITY_DN14663_c0_g1/TRINITY_DN39318_c0_g1/TRINITY_DN428_c0_g1/TRINITY_DN28241_c0_g1/TRINITY_DN1186_c0_g1/TRINITY_DN4000_c0_g2/TRINITY_DN2961_c0_g1/TRINITY_DN29	39

							720_c0_g2/TRINITY_DN16754_c0_g1/TRINITY_DN49433_c0_g1/TRINITY_DN14134_c0_g1/TRINITY_DN8692_c0_g1	
GO:0036159	inner dynein arm assembly	10/2626	13/33142	2.20 × 10^{-9}	2.00 × 10^{-7}	1.49 × 10^{-7}	TRINITY_DN6657_c0_g1/TRINITY_DN19725_c0_g1/TRINITY_DN1979_c0_g2/TRINITY_DN1979_c0_g1/TRINITY_DN20881_c0_g2/TRINITY_DN3083_c0_g1/TRINITY_DN15113_c0_g1/TRINITY_DN5914_c0_g1/TRINITY_DN8872_c0_g1/TRINITY_DN3299_c0_g1	10
GO:0015651	quaternary ammonium group transmembrane transporter activity	11/2626	16/33142	2.28 × 10^{-9}	2.00 × 10^{-7}	1.49 × 10^{-7}	TRINITY_DN2006_c0_g1/TRINITY_DN2770_c0_g1/TRINITY_DN5871_c1_g1/TRINITY_DN1478_c1_g3/TRINITY_DN14468_c0_g1/TRINITY_DN10811_c0_g1/TRINITY_DN10798_c0_g1/TRINITY_DN11056_c1_g1/TRINITY_DN11061_c0_g2/TRINITY_DN24632_c0_g1/TRINITY_DN216_c0_g3	11
GO:0007605	sensory perception of sound	25/2626	82/33142	2.33 × 10^{-9}	2.00 × 10^{-7}	1.49 × 10^{-7}	TRINITY_DN7437_c0_g1/TRINITY_DN9358_c0_g1/TRINITY_DN72971_c0_g1/TRINITY_DN16444_c1_g1/TRINITY_DN5613_c1_g1/TRINITY_DN1557_c0_g1/TRINITY_DN6174_c0_g1/TRINITY_DN47_c0_g1/TRINITY_DN388_c0_g1/TRINITY_DN3296_c0_g1/TRINITY_DN	25

insects

							4826_c1_g1/TRINITY_DN16407_c1_g1/TRINITY_DN85_c0_g1/TRINITY_DN7759_c0_g1/TRINITY_DN5459_c0_g1/TRINITY_DN1267_c0_g2/TRINITY_DN25715_c0_g1/TRINITY_DN23750_c0_g1/TRINITY_DN2746_c0_g1/TRINITY_DN22905_c0_g1/TRINITY_DN338_c0_g1/TRINITY_DN26007_c0_g2/TRINITY_DN2094_c0_g1/TRINITY_DN2347_c1_g1/TRINITY_DN43641_c0_g1	
GO:0005856	cytoskeleton	48/2626	242/33142	2.85×10^{-9}	2.36×10^{-7}	1.77×10^{-7}	TRINITY_DN9277_c0_g1/TRINITY_DN9358_c0_g1/TRINITY_DN3120_c0_g1/TRINITY_DN57787_c0_g1/TRINITY_DN2729_c0_g1/TRINITY_DN235_c0_g1/TRINITY_DN47_c0_g1/TRINITY_DN15748_c0_g1/TRINITY_DN14031_c0_g1/TRINITY_DN9720_c0_g1/TRINITY_DN69323_c0_g1/TRINITY_DN15574_c0_g1/TRINITY_DN7939_c1_g1/TRINITY_DN6222_c0_g1/TRINITY_DN16032_c0_g1/TRINITY_DN36928_c0_g1/TRINITY_DN7759_c0_g1/TRINITY_DN17565_c0_g1/TRINITY_DN1633_c0_g1/TRINITY_DN1775_c0_g3/TRINITY_DN1420_c0_g1/TRINITY_DN6081_c1_g	48

							2/TRINITY_DN377_c0_g1/TRINITY_DN22758_c0_g1/TRINITY_DN11027_c0_g1/TRINITY_DN11154_c0_g1/TRINITY_DN3665_c0_g1/TRINITY_DN19224_c0_g1/TRINITY_DN9935_c1_g1/TRINITY_DN1532_c0_g1/TRINITY_DN4325_c0_g1/TRINITY_DN8675_c0_g2/TRINITY_DN9279_c0_g1/TRINITY_DN30302_c0_g1/TRINITY_DN10587_c0_g1/TRINITY_DN3890_c0_g1/TRINITY_DN39318_c0_g1/TRINITY_DN6219_c0_g1/TRINITY_DN2347_c1_g1/TRINITY_DN7420_c0_g1/TRINITY_DN10176_c0_g1/TRINITY_DN15794_c0_g1/TRINITY_DN10111_c0_g1/TRINITY_DN6505_c0_g1/TRINITY_DN43066_c0_g1/TRINITY_DN26727_c0_g1/TRINITY_DN269_c0_g1/TRINITY_DN4487_c3_g2	
GO:0006811	ion transport	55/2626	299/33142	3.65×10^{-9}	2.94×10^{-7}	2.20×10^{-7}	TRINITY_DN7814_c0_g2/TRINITY_DN6597_c0_g1/TRINITY_DN755_c2_g1/TRINITY_DN4992_c0_g1/TRINITY_DN2078_c0_g1/TRINITY_DN1748_c0_g1/TRINITY_DN3179_c0_g2/TRINITY_DN5378_c1_g1/TRINITY_DN2279_c0_g1/TRINITY_DN8167_c0_g1/TRINITY_D	55

N10654_c0_g1/TRINITY_DN1
7609_c0_g1/TRINITY_DN4385
_c0_g1/TRINITY_DN3654_c0_
g1/TRINITY_DN24459_c0_g1/
TRINITY_DN10707_c0_g1/TRI
NITY_DN2307_c0_g1/TRINIT
Y_DN6323_c1_g1/TRINITY_D
N2924_c1_g1/TRINITY_DN16
407_c1_g1/TRINITY_DN14468
_c0_g1/TRINITY_DN4958_c0_
g1/TRINITY_DN1316_c0_g1/T
RINITY_DN7659_c0_g1/TRIN
ITY_DN6079_c0_g1/TRINITY_
DN4477_c0_g1/TRINITY_DN3
180_c0_g1/TRINITY_DN16593
_c0_g1/TRINITY_DN19635_c0
_g1/TRINITY_DN10811_c0_g1
/TRINITY_DN7213_c0_g1/TRI
NITY_DN13011_c0_g1/TRINI
TY_DN7054_c0_g1/TRINITY_
DN6655_c0_g1/TRINITY_DN2
0528_c0_g1/TRINITY_DN6745
_c0_g1/TRINITY_DN5678_c0_
g1/TRINITY_DN14953_c0_g3/
TRINITY_DN2938_c3_g1/TRI
NITY_DN6690_c1_g1/TRINIT
Y_DN17398_c0_g2/TRINITY_
DN8644_c0_g1/TRINITY_DN1
1056_c1_g1/TRINITY_DN3030
_c0_g1/TRINITY_DN4757_c0_
g1/TRINITY_DN2003_c0_g1/T
RINITY_DN4264_c1_g1/TRIN

insects MDPI

							ITY_DN35025_c0_g1/TRINITY_DN4522_c0_g1/TRINITY_DN4000_c0_g2/TRINITY_DN4784_c1_g1/TRINITY_DN4674_c0_g1/TRINITY_DN10346_c1_g1/TRINITY_DN940_c0_g1/TRINITY_DN18471_c0_g2	
GO:0042073	intraciliary transport	12/2626	20/33142	4.12×10^{-9}	3.23×10^{-7}	2.42×10^{-7}	TRINITY_DN11509_c0_g1/TRINITY_DN6327_c1_g1/TRINITY_DN7196_c0_g1/TRINITY_DN5006_c0_g2/TRINITY_DN4043_c0_g1/TRINITY_DN9930_c0_g2/TRINITY_DN11340_c0_g1/TRINITY_DN13406_c0_g1/TRINITY_DN5875_c0_g1/TRINITY_DN2153_c0_g1/TRINITY_DN11027_c0_g1/TRINITY_DN16662_c0_g1	12
GO:0006030	chitin metabolic process	20/2626	56/33142	4.27×10^{-9}	3.26×10^{-7}	2.44×10^{-7}	TRINITY_DN5758_c0_g1/TRINITY_DN5801_c2_g2/TRINITY_DN6856_c0_g1/TRINITY_DN5537_c1_g1/TRINITY_DN8247_c0_g2/TRINITY_DN388_c0_g1/TRINITY_DN18094_c0_g1/TRINITY_DN12807_c0_g1/TRINITY_DN5448_c0_g1/TRINITY_DN16164_c0_g1/TRINITY_DN5168_c0_g1/TRINITY_DN3155_c0_g1/TRINITY_DN8589_c0_g1/TRINITY_DN1720_c0_g1/TRINITY_DN4912_c0_g1/TRINITY_DN5801_c2_g1/TRINIT	20

							Y_DN3148_c0_g1/TRINITY_DN12119_c0_g1/TRINITY_DN13068_c0_g1/TRINITY_DN5674_c2_g1	
GO:0005764	lysosome	42/2626	201/33142	5.50 × 10^{-9}	4.09 × 10^{-7}	3.06 × 10^{-7}	TRINITY_DN3991_c0_g1/TRINITY_DN7437_c0_g1/TRINITY_DN5056_c0_g1/TRINITY_DN72971_c0_g1/TRINITY_DN8493_c0_g1/TRINITY_DN1487_c0_g2/TRINITY_DN14379_c0_g1/TRINITY_DN9425_c0_g1/TRINITY_DN43642_c0_g1/TRINITY_DN16579_c1_g1/TRINITY_DN4687_c0_g1/TRINITY_DN2648_c0_g1/TRINITY_DN7743_c0_g1/TRINITY_DN6698_c0_g1/TRINITY_DN5278_c0_g1/TRINITY_DN200_c0_g2/TRINITY_DN8257_c0_g1/TRINITY_DN4826_c1_g1/TRINITY_DN16407_c1_g1/TRINITY_DN4477_c0_g1/TRINITY_DN19401_c0_g1/TRINITY_DN1252_c0_g1/TRINITY_DN15112_c0_g1/TRINITY_DN23951_c1_g1/TRINITY_DN19148_c0_g1/TRINITY_DN2682_c0_g1/TRINITY_DN8278_c2_g1/TRINITY_DN6654_c0_g1/TRINITY_DN7045_c1_g1/TRINITY_DN656_c0_g1/TRINITY_DN1532_c0_g1/TRINITY_DN15289_c0_g1/TRINI	42

							TY_DN3115_c0_g3/TRINITY_DN5009_c0_g2/TRINITY_DN55372_c0_g1/TRINITY_DN1598_c1_g1/TRINITY_DN9937_c0_g1/TRINITY_DN21292_c0_g1/TRINITY_DN43641_c0_g1/TRINITY_DN4674_c0_g1/TRINITY_DN111160_c0_g1/TRINITY_DN4895_c1_g1	
GO:0032983	kainate selective glutamate receptor complex	9/2626	11/33142	5.77×10^{-9}	4.18×10^{-7}	3.13×10^{-7}	TRINITY_DN21399_c0_g2/TRINITY_DN8355_c0_g1/TRINITY_DN1765_c2_g1/TRINITY_DN3816_c0_g1/TRINITY_DN9041_c0_g1/TRINITY_DN15420_c0_g1/TRINITY_DN7200_c0_g2/TRINITY_DN9041_c0_g2/TRINITY_DN21399_c0_g1	9
GO:0030286	dynein complex	25/2626	86/33142	6.95×10^{-9}	4.90×10^{-7}	3.67×10^{-7}	TRINITY_DN9451_c0_g1/TRINITY_DN5849_c0_g2/TRINITY_DN1979_c0_g2/TRINITY_DN13313_c0_g1/TRINITY_DN1979_c0_g1/TRINITY_DN4543_c0_g2/TRINITY_DN233_c0_g1/TRINITY_DN3083_c0_g1/TRINITY_DN1979_c1_g1/TRINITY_DN4208_c0_g1/TRINITY_DN18134_c0_g1/TRINITY_DN1909_c0_g1/TRINITY_DN3767_c0_g1/TRINITY_DN10356_c0_g1/TRINITY_DN2372_c0_g1/TRINITY_DN5968_c0_g1/TRINITY_DN436_c2_g1/TRINITY_	25

							DN14798_c0_g1/TRINITY_DN35591_c0_g1/TRINITY_DN31689_c0_g1/TRINITY_DN6282_c0_g1/TRINITY_DN9647_c0_g1/TRINITY_DN29956_c0_g1/TRINITY_DN5578_c0_g1/TRINITY_DN39491_c0_g1	
GO:0016323	basolateral plasma membrane	30/2626	119/33142	9.24×10^{-9}	6.37×10^{-7}	4.77×10^{-7}	TRINITY_DN1748_c0_g1/TRINITY_DN473_c0_g1/TRINITY_DN5886_c0_g1/TRINITY_DN2770_c0_g1/TRINITY_DN4503_c0_g3/TRINITY_DN406_c0_g3/TRINITY_DN5871_c1_g1/TRINITY_DN5049_c0_g1/TRINITY_DN10707_c0_g1/TRINITY_DN2307_c0_g1/TRINITY_DN28373_c0_g1/TRINITY_DN2999_c0_g1/TRINITY_DN7737_c0_g1/TRINITY_DN16407_c1_g1/TRINITY_DN32456_c0_g2/TRINITY_DN1267_c0_g2/TRINITY_DN1614_c0_g1/TRINITY_DN25715_c0_g1/TRINITY_DN406_c0_g2/TRINITY_DN10811_c0_g1/TRINITY_DN8284_c0_g1/TRINITY_DN6745_c0_g1/TRINITY_DN11886_c0_g1/TRINITY_DN8644_c0_g1/TRINITY_DN8569_c0_g1/TRINITY_DN23609_c0_g1/TRINITY_DN24632_c0_g1/TRINITY_DN4700_c0	30

							_g1/TRINITY_DN42565_c0_g1/TRINITY_DN10346_c1_g1	
GO:0005858	axonemal dynein complex	13/2626	25/33142	9.87×10^{-9}	6.64×10^{-7}	4.97×10^{-7}	TRINITY_DN9451_c0_g1/TRINITY_DN1979_c0_g1/TRINITY_DN4543_c0_g2/TRINITY_DN3083_c0_g1/TRINITY_DN18134_c0_g1/TRINITY_DN5968_c0_g1/TRINITY_DN14798_c0_g1/TRINITY_DN10133_c0_g1/TRINITY_DN16784_c0_g1/TRINITY_DN31689_c0_g1/TRINITY_DN9647_c0_g1/TRINITY_DN28131_c0_g1/TRINITY_DN3427_c0_g1	13
GO:0007155	cell adhesion	37/2626	169/33142	1.18×10^{-8}	7.73×10^{-7}	5.79×10^{-7}	TRINITY_DN12244_c0_g1/TRINITY_DN398_c0_g1/TRINITY_DN7148_c1_g1/TRINITY_DN9277_c0_g1/TRINITY_DN83267_c0_g1/TRINITY_DN2841_c0_g1/TRINITY_DN3740_c1_g1/TRINITY_DN22536_c0_g1/TRINITY_DN6174_c0_g1/TRINITY_DN59294_c0_g1/TRINITY_DN3487_c0_g1/TRINITY_DN11100_c0_g1/TRINITY_DN388_c0_g1/TRINITY_DN1266_c0_g1/TRINITY_DN34715_c0_g1/TRINITY_DN56882_c0_g1/TRINITY_DN4210_c0_g1/TRINITY_DN2786_c0_g1/TRINITY_DN1902_c0_g1/TRINITY_DN10018_c0_g1/TRINITY_DN4620_	37

							c0_g1/TRINITY_DN24147_c0_g1/TRINITY_DN2292_c2_g1/TRINITY_DN346_c0_g1/TRINITY_DN93_c0_g1/TRINITY_DN13362_c0_g1/TRINITY_DN144_c0_g1/TRINITY_DN12127_c0_g1/TRINITY_DN4321_c3_g1/TRINITY_DN21548_c0_g1/TRINITY_DN13862_c0_g2/TRINITY_DN44691_c0_g1/TRINITY_DN12958_c0_g1/TRINITY_DN9850_c0_g1/TRINITY_DN7420_c0_g1/TRINITY_DN2168_c3_g1/TRINITY_DN2285_c0_g1	
GO:0036064	ciliary basal body	20/2626	59/33142	1.20×10^{-8}	7.73×10^{-7}	5.79×10^{-7}	TRINITY_DN11509_c0_g1/TRINITY_DN6327_c1_g1/TRINITY_DN7740_c0_g1/TRINITY_DN36172_c0_g1/TRINITY_DN21912_c0_g1/TRINITY_DN4043_c0_g1/TRINITY_DN7556_c0_g1/TRINITY_DN12886_c0_g1/TRINITY_DN4988_c2_g1/TRINITY_DN5875_c0_g1/TRINITY_DN306_c1_g2/TRINITY_DN2153_c0_g1/TRINITY_DN5912_c0_g1/TRINITY_DN15411_c0_g1/TRINITY_DN2746_c0_g1/TRINITY_DN9168_c0_g2/TRINITY_DN10366_c0_g1/TRINITY_DN59259_c0_g1/TRINITY	20

							_DN16662_c0_g1/TRINITY_DN8113_c0_g2	
GO:0098978	glutamatergic synapse	29/2626	114/33142	1.30 × 10^{-8}	8.17 × 10^{-7}	6.11 × 10^{-7}	TRINITY_DN21399_c0_g2/TRINITY_DN2738_c0_g1/TRINITY_DN11100_c0_g1/TRINITY_DN6108_c0_g1/TRINITY_DN7277_c1_g1/TRINITY_DN31967_c0_g1/TRINITY_DN8355_c0_g1/TRINITY_DN740_c0_g1/TRINITY_DN1775_c0_g3/TRINITY_DN2598_c0_g2/TRINITY_DN11938_c0_g1/TRINITY_DN1765_c2_g1/TRINITY_DN1420_c0_g1/TRINITY_DN3816_c0_g1/TRINITY_DN9935_c1_g1/TRINITY_DN9041_c0_g1/TRINITY_DN10575_c2_g1/TRINITY_DN6910_c0_g1/TRINITY_DN15420_c0_g1/TRINITY_DN7200_c0_g2/TRINITY_DN11061_c0_g2/TRINITY_DN9041_c0_g2/TRINITY_DN12650_c0_g1/TRINITY_DN2094_c0_g1/TRINITY_DN1908_c0_g1/TRINITY_DN6505_c0_g1/TRINITY_DN49433_c0_g1/TRINITY_DN4487_c3_g2/TRINITY_DN14134_c0_g1	29
GO:0007283	spermatogenesis	38/2626	177/33142	1.33 × 10^{-8}	8.17 × 10^{-7}	6.11 × 10^{-7}	TRINITY_DN3991_c0_g1/TRINITY_DN2841_c0_g1/TRINITY_DN402_c1_g1/TRINITY_DN6515_c0_g1/TRINITY_DN12	38

							450_c0_g2/TRINITY_DN4544_c0_g1/TRINITY_DN7756_c0_g1/TRINITY_DN7743_c0_g1/TRINITY_DN7530_c0_g1/TRINITY_DN12628_c0_g2/TRINITY_DN21912_c0_g1/TRINITY_DN12628_c0_g1/TRINITY_DN3007_c0_g1/TRINITY_DN6323_c1_g1/TRINITY_DN2779_c1_g2/TRINITY_DN9930_c0_g2/TRINITY_DN3983_c0_g1/TRINITY_DN806_c0_g2/TRINITY_DN8796_c0_g1/TRINITY_DN7744_c0_g1/TRINITY_DN170_c1_g1/TRINITY_DN10783_c0_g1/TRINITY_DN7566_c0_g2/TRINITY_DN2682_c0_g1/TRINITY_DN1178_c2_g1/TRINITY_DN3665_c0_g1/TRINITY_DN2779_c1_g3/TRINITY_DN898_c0_g1/TRINITY_DN3032_c1_g2/TRINITY_DN2869_c2_g1/TRINITY_DN18013_c0_g1/TRINITY_DN7067_c0_g1/TRINITY_DN806_c0_g1/TRINITY_DN30782_c0_g2/TRINITY_DN1071_c0_g1/TRINITY_DN26727_c0_g1/TRINITY_DN14134_c0_g1/TRINITY_DN21101_c0_g1	
GO:0007018	microtubule-based movement	30/2626	121/33142	1.40×10^{-8}	8.39×10^{-7}	6.28×10^{-7}	TRINITY_DN11509_c0_g1/TRINITY_DN19161_c0_g1/TRINITY_DN9451_c0_g1/TRINITY_	30

							DN1979_c0_g2/TRINITY_DN1265_c0_g1/TRINITY_DN5811_c0_g1/TRINITY_DN552_c0_g1/TRINITY_DN1979_c0_g1/TRINITY_DN4543_c0_g2/TRINITY_DN3083_c0_g1/TRINITY_DN1979_c1_g1/TRINITY_DN18134_c0_g1/TRINITY_DN1909_c0_g1/TRINITY_DN3767_c0_g1/TRINITY_DN10356_c0_g1/TRINITY_DN18791_c0_g1/TRINITY_DN5968_c0_g1/TRINITY_DN436_c2_g1/TRINITY_DN12133_c0_g1/TRINITY_DN14798_c0_g1/TRINITY_DN35591_c0_g1/TRINITY_DN31689_c0_g1/TRINITY_DN6282_c0_g1/TRINITY_DN9647_c0_g1/TRINITY_DN5922_c0_g1/TRINITY_DN29956_c0_g1/TRINITY_DN5578_c0_g1/TRINITY_DN13780_c0_g1/TRINITY_DN39491_c0_g1/TRINITY_DN15080_c0_g1	
GO:0015695	organic cation transport	11/2626	18/33142	1.43×10^{-8}	8.39×10^{-7}	6.28×10^{-7}	TRINITY_DN2006_c0_g1/TRINITY_DN5871_c1_g1/TRINITY_DN19520_c0_g1/TRINITY_DN1478_c1_g3/TRINITY_DN14468_c0_g1/TRINITY_DN10811_c0_g1/TRINITY_DN10798_c0_g1/TRINITY_DN11056_c1_g1/TRINITY_DN11061_c0_g2/T	11

							RINITY_DN24632_c0_g1/TRINITY_DN8729_c1_g1	
GO:0005814	centriole	16/2626	39/33142	1.49 × 10^{-8}	8.47 × 10^{-7}	6.34 × 10^{-7}	TRINITY_DN9943_c0_g1/TRINITY_DN2841_c0_g1/TRINITY_DN13157_c0_g1/TRINITY_DN11936_c0_g1/TRINITY_DN15863_c0_g1/TRINITY_DN11896_c0_g2/TRINITY_DN3071_c0_g1/TRINITY_DN21912_c0_g1/TRINITY_DN7556_c0_g1/TRINITY_DN11340_c0_g1/TRINITY_DN3983_c0_g1/TRINITY_DN4988_c2_g1/TRINITY_DN306_c1_g2/TRINITY_DN5912_c0_g1/TRINITY_DN9168_c0_g2/TRINITY_DN10366_c0_g1	16
GO:0008061	chitin binding	25/2626	89/33142	1.50 × 10^{-8}	8.47 × 10^{-7}	6.34 × 10^{-7}	TRINITY_DN5758_c0_g1/TRINITY_DN5801_c2_g2/TRINITY_DN8264_c0_g1/TRINITY_DN6856_c0_g1/TRINITY_DN5537_c1_g1/TRINITY_DN8247_c0_g2/TRINITY_DN388_c0_g1/TRINITY_DN18094_c0_g1/TRINITY_DN4134_c0_g1/TRINITY_DN12807_c0_g1/TRINITY_DN5448_c0_g1/TRINITY_DN16164_c0_g1/TRINITY_DN5168_c0_g1/TRINITY_DN28998_c0_g1/TRINITY_DN3155_c0_g1/TRINITY_DN8589_c0_g1/TRINITY_DN19148_c0_g1/TRINITY_DN1720_c0_g1/TRINITY_	25

insects

MDPI

							DN4912_c0_g1/TRINITY_DN5801_c2_g1/TRINITY_DN3148_c0_g1/TRINITY_DN12119_c0_g1/TRINITY_DN27704_c0_g1/TRINITY_DN13068_c0_g1/TRINITY_DN5674_c2_g1	
GO:0052650	NADP-retinol dehydrogenase activity	14/2626	30/33142	1.60×10^{-8}	8.85×10^{-7}	6.63×10^{-7}	TRINITY_DN146_c0_g1/TRINITY_DN3778_c2_g1/TRINITY_DN19075_c0_g1/TRINITY_DN21901_c0_g1/TRINITY_DN10027_c0_g1/TRINITY_DN51872_c0_g1/TRINITY_DN25301_c0_g1/TRINITY_DN31870_c0_g1/TRINITY_DN12540_c0_g1/TRINITY_DN96301_c0_g1/TRINITY_DN9845_c0_g2/TRINITY_DN20346_c0_g1/TRINITY_DN49166_c0_g1/TRINITY_DN33814_c0_g1	14
GO:0035249	synaptic transmission， glutamatergic	13/2626	26/33142	1.83×10^{-8}	9.94×10^{-7}	7.44×10^{-7}	TRINITY_DN21399_c0_g2/TRINITY_DN16006_c0_g1/TRINITY_DN27259_c0_g1/TRINITY_DN8355_c0_g1/TRINITY_DN11938_c0_g1/TRINITY_DN1765_c2_g1/TRINITY_DN3816_c0_g1/TRINITY_DN9041_c0_g1/TRINITY_DN7200_c0_g2/TRINITY_DN9041_c0_g2/TRINITY_DN4674_c0_g1/TRINITY_DN49433_c0_g1/TRINITY_DN21399_c0_g1	13

GO:0004806	triglyceride lipase activity	19/2626	55/33142	1.95 × 10^{-8}	1.04 × 10^{-6}	7.79 × 10^{-7}	TRINITY_DN5872_c0_g1/TRINITY_DN6455_c0_g1/TRINITY_DN6700_c0_g1/TRINITY_DN5164_c0_g3/TRINITY_DN1568_c0_g1/TRINITY_DN2675_c0_g1/TRINITY_DN5164_c0_g2/TRINITY_DN9375_c0_g1/TRINITY_DN23263_c0_g1/TRINITY_DN4211_c1_g3/TRINITY_DN39313_c0_g1/TRINITY_DN6482_c0_g1/TRINITY_DN18499_c0_g1/TRINITY_DN21191_c0_g1/TRINITY_DN4537_c0_g1/TRINITY_DN26376_c0_g1/TRINITY_DN2780_c2_g1/TRINITY_DN5274_c0_g1/TRINITY_DN3989_c1_g1	19
GO:0045177	apical part of cell	24/2626	84/33142	2.01 × 10^{-8}	1.05 × 10^{-6}	7.87 × 10^{-7}	TRINITY_DN7384_c0_g1/TRINITY_DN4783_c0_g1/TRINITY_DN6174_c0_g1/TRINITY_DN11631_c0_g3/TRINITY_DN4514_c0_g1/TRINITY_DN42262_c0_g1/TRINITY_DN14877_c1_g1/TRINITY_DN3305_c0_g1/TRINITY_DN6348_c0_g1/TRINITY_DN1614_c0_g1/TRINITY_DN11583_c0_g1/TRINITY_DN25715_c0_g1/TRINITY_DN6810_c0_g1/TRINITY_DN3665_c0_g1/TRINITY_DN24147_c0_g1/TRINITY_DN10145_c1_g1/TRINITY_DN6745_c0_g1/TRI	24

insects MDPI

GO:0007411	axon guidance	40/2626	195/33142	2.17×10^{-8}	1.11×10^{-6}	8.33×10^{-7}	NITY_DN1118_c0_g1/TRINITY_DN273_c1_g1/TRINITY_DN2321_c0_g1/TRINITY_DN12958_c0_g1/TRINITY_DN1941_c0_g1/TRINITY_DN26727_c0_g1/TRINITY_DN18007_c0_g1TRINITY_DN180_c2_g1/TRINITY_DN2244_c1_g1/TRINITY_DN22690_c0_g1/TRINITY_DN11100_c0_g1/TRINITY_DN8297_c0_g1/TRINITY_DN4097_c0_g1/TRINITY_DN154_c0_g1/TRINITY_DN2133_c0_g1/TRINITY_DN2868_c0_g2/TRINITY_DN4169_c0_g2/TRINITY_DN4169_c0_g1/TRINITY_DN6076_c0_g1/TRINITY_DN2531_c0_g1/TRINITY_DN479_c0_g2/TRINITY_DN5781_c0_g1/TRINITY_DN2424_c0_g1/TRINITY_DN8217_c1_g1/TRINITY_DN5091_c0_g1/TRINITY_DN43399_c0_g1/TRINITY_DN7898_c0_g1/TRINITY_DN35269_c0_g1/TRINITY_DN419_c0_g2/TRINITY_DN24893_c0_g1/TRINITY_DN27045_c0_g1/TRINITY_DN2475_c0_g1/TRINITY_DN12452_c0_g1/TRINITY_DN4325_c0_g1/TRINITY_DN10575_c2_g1/TRINITY_DN9158_c3_g1/TRINITY_DN3333_c0_g1/TRIN	40

							ITY_DN446_c1_g1/TRINITY_DN709_c0_g1/TRINITY_DN3241_c0_g1/TRINITY_DN44691_c0_g1/TRINITY_DN14663_c0_g1/TRINITY_DN428_c0_g1/TRINITY_DN2168_c3_g1/TRINITY_DN2961_c0_g1/TRINITY_DN3810_c0_g1/TRINITY_DN13354_c0_g1	
GO:0030425	dendrite	43/2626	219/33142	2.53×10^{-8}	1.28×10^{-6}	9.56×10^{-7}	TRINITY_DN7437_c0_g1/TRINITY_DN21399_c0_g2/TRINITY_DN8323_c0_g1/TRINITY_DN3446_c0_g1/TRINITY_DN3245_c0_g1/TRINITY_DN1487_c0_g2/TRINITY_DN402_c1_g1/TRINITY_DN30087_c1_g2/TRINITY_DN1265_c0_g1/TRINITY_DN4329_c1_g1/TRINITY_DN13394_c0_g1/TRINITY_DN200_c0_g2/TRINITY_DN17663_c0_g1/TRINITY_DN2786_c0_g1/TRINITY_DN18321_c0_g1/TRINITY_DN8355_c0_g1/TRINITY_DN233_c0_g1/TRINITY_DN2415_c2_g1/TRINITY_DN8012_c0_g1/TRINITY_DN1765_c2_g1/TRINITY_DN3816_c0_g1/TRINITY_DN3665_c0_g1/TRINITY_DN9175_c0_g1/TRINITY_DN13011_c0_g1/TRINITY_DN8599_c1_g1/TRINITY_DN2475_c0_g1/TRINITY_DN2746_	43

insects MDPI

							c0_g1/TRINITY_DN19691_c1_g1/TRINITY_DN26935_c0_g1/TRINITY_DN15420_c0_g1/TRINITY_DN7687_c0_g1/TRINITY_DN7200_c0_g2/TRINITY_DN3238_c0_g1/TRINITY_DN11061_c0_g2/TRINITY_DN12650_c0_g1/TRINITY_DN14663_c0_g1/TRINITY_DN16530_c0_g1/TRINITY_DN428_c0_g1/TRINITY_DN52030_c0_g1/TRINITY_DN4000_c0_g2/TRINITY_DN7084_c0_g1/TRINITY_DN4784_c1_g1/TRINITY_DN26727_c0_g1	
GO:0007601	visual perception	31/2626	132/33142	3.18×10^{-8}	1.58×10^{-6}	1.18×10^{-6}	TRINITY_DN8493_c0_g1/TRINITY_DN1487_c0_g2/TRINITY_DN19075_c0_g1/TRINITY_DN2489_c0_g1/TRINITY_DN15240_c0_g1/TRINITY_DN7582_c0_g1/TRINITY_DN7048_c0_g1/TRINITY_DN183_c0_g1/TRINITY_DN5239_c1_g2/TRINITY_DN47_c0_g1/TRINITY_DN1120_c0_g2/TRINITY_DN5871_c1_g1/TRINITY_DN5198_c0_g1/TRINITY_DN20958_c0_g1/TRINITY_DN2492_c0_g1/TRINITY_DN2924_c1_g1/TRINITY_DN41433_c0_g1/TRINITY_DN1229_c2_g1/TRINITY_DN31870_c0_g1/TRINITY_DN2095	31

							8_c0_g2/TRINITY_DN5459_c0_g1/TRINITY_DN6805_c0_g1/TRINITY_DN33474_c0_g1/TRINITY_DN20346_c0_g1/TRINITY_DN25570_c0_g1/TRINITY_DN5121_c0_g1/TRINITY_DN2094_c0_g1/TRINITY_DN4264_c1_g1/TRINITY_DN61_c0_g1/TRINITY_DN4784_c1_g1/TRINITY_DN16662_c0_g1	
GO:0031526	brush border membrane	16/2626	41/33142	3.50×10^{-8}	1.66×10^{-6}	1.24×10^{-6}	TRINITY_DN3991_c0_g1/TRINITY_DN7437_c0_g1/TRINITY_DN4514_c0_g1/TRINITY_DN14877_c1_g1/TRINITY_DN5495_c1_g2/TRINITY_DN14381_c0_g1/TRINITY_DN9333_c0_g1/TRINITY_DN2682_c0_g1/TRINITY_DN7880_c0_g1/TRINITY_DN6705_c0_g2/TRINITY_DN22905_c0_g1/TRINITY_DN11886_c0_g1/TRINITY_DN9521_c0_g1/TRINITY_DN4384_c0_g1/TRINITY_DN216_c0_g3/TRINITY_DN10346_c1_g1	16
GO:0042438	melanin biosynthetic process	12/2626	23/33142	3.53×10^{-8}	1.66×10^{-6}	1.24×10^{-6}	TRINITY_DN2677_c0_g1/TRINITY_DN8260_c0_g2/TRINITY_DN9643_c0_g1/TRINITY_DN16500_c1_g1/TRINITY_DN3123_c0_g1/TRINITY_DN15335_c0_g1/TRINITY_DN855_c0_g1/TRINITY_DN1818_c2_g1/TRINITY_DN9994_c0_g1/TRINIT	12

							Y_DN12402_c0_g1/TRINITY_DN4947_c0_g1/TRINITY_DN14093_c0_g1	
GO:0090557	establishment of endothelial intestinal barrier	12/2626	23/33142	3.53×10^{-8}	1.66×10^{-6}	1.24×10^{-6}	TRINITY_DN19001_c0_g1/TRINITY_DN1614_c0_g1/TRINITY_DN23921_c0_g1/TRINITY_DN482_c1_g1/TRINITY_DN29856_c0_g1/TRINITY_DN1549_c0_g1/TRINITY_DN12127_c0_g1/TRINITY_DN40189_c0_g1/TRINITY_DN3178_c0_g1/TRINITY_DN484_c0_g1/TRINITY_DN244_c3_g1/TRINITY_DN61025_c0_g1	12
GO:0015020	glucuronosyltransferase activity	15/2626	37/33142	5.11×10^{-8}	2.36×10^{-6}	1.77×10^{-6}	TRINITY_DN32009_c0_g1/TRINITY_DN16979_c0_g1/TRINITY_DN11954_c0_g1/TRINITY_DN638_c0_g2/TRINITY_DN23485_c0_g1/TRINITY_DN5087_c0_g1/TRINITY_DN10011_c0_g1/TRINITY_DN4160_c0_g1/TRINITY_DN10583_c0_g1/TRINITY_DN974_c0_g1/TRINITY_DN15324_c1_g1/TRINITY_DN13235_c0_g1/TRINITY_DN13226_c0_g1/TRINITY_DN1200_c2_g1/TRINITY_DN8845_c0_g3	15
GO:0006629	lipid metabolic process	41/2626	209/33142	5.43×10^{-8}	2.47×10^{-6}	1.85×10^{-6}	TRINITY_DN7437_c0_g1/TRINITY_DN6455_c0_g1/TRINITY_DN77269_c0_g1/TRINITY_DN5164_c0_g3/TRINITY_DN8	41

909_c0_g1/TRINITY_DN11372
_c0_g1/TRINITY_DN11631_c0
_g3/TRINITY_DN3479_c0_g1/
TRINITY_DN21814_c0_g2/TRI
NITY_DN8236_c0_g1/TRINIT
Y_DN11204_c0_g1/TRINITY_
DN6049_c1_g2/TRINITY_DN1
659_c1_g2/TRINITY_DN6767_
c0_g1/TRINITY_DN17663_c0_
g1/TRINITY_DN23263_c0_g1/
TRINITY_DN4826_c1_g1/TRI
NITY_DN3305_c0_g1/TRINIT
Y_DN4485_c0_g1/TRINITY_D
N59840_c0_g1/TRINITY_DN2
8084_c0_g1/TRINITY_DN6435
_c0_g3/TRINITY_DN9779_c0_
g1/TRINITY_DN6810_c0_g1/T
RINITY_DN5693_c0_g2/TRIN
ITY_DN656_c0_g1/TRINITY_
DN5833_c0_g1/TRINITY_DN9
780_c0_g1/TRINITY_DN40503
_c0_g1/TRINITY_DN4537_c0_
g1/TRINITY_DN596_c8_g1/TR
INITY_DN15536_c0_g1/TRINI
TY_DN5274_c0_g1/TRINITY_
DN10316_c0_g1/TRINITY_DN
4037_c0_g1/TRINITY_DN3989
_c1_g1/TRINITY_DN12338_c0
_g1/TRINITY_DN5555_c0_g2/
TRINITY_DN43641_c0_g1/TRI
NITY_DN12549_c0_g1/TRINI
TY_DN18007_c0_g1

GO:0031012	extracellular matrix	22/2626	76/33142	5.97 × 10^{-8}	2.67 × 10^{-6}	2.00 × 10^{-6}	TRINITY_DN398_c0_g1/TRINITY_DN2870_c0_g1/TRINITY_DN6305_c1_g1/TRINITY_DN950_c4_g1/TRINITY_DN3487_c0_g1/TRINITY_DN42941_c0_g1/TRINITY_DN388_c0_g1/TRINITY_DN1266_c0_g1/TRINITY_DN15399_c0_g1/TRINITY_DN1120_c0_g2/TRINITY_DN8217_c0_g1/TRINITY_DN4210_c0_g1/TRINITY_DN479_c0_g2/TRINITY_DN740_c0_g1/TRINITY_DN2868_c0_g1/TRINITY_DN7367_c0_g1/TRINITY_DN4620_c0_g1/TRINITY_DN4437_c0_g1/TRINITY_DN403_c1_g1/TRINITY_DN5908_c0_g1/TRINITY_DN27704_c0_g1/TRINITY_DN16986_c0_g2	22
GO:0014069	postsynaptic density	27/2626	108/33142	6.04 × 10^{-8}	2.67 × 10^{-6}	2.00 × 10^{-6}	TRINITY_DN21399_c0_g2/TRINITY_DN4329_c1_g1/TRINITY_DN6108_c0_g1/TRINITY_DN2786_c0_g1/TRINITY_DN15308_c0_g2/TRINITY_DN1775_c0_g3/TRINITY_DN1765_c2_g1/TRINITY_DN1420_c0_g1/TRINITY_DN3816_c0_g1/TRINITY_DN3722_c0_g1/TRINITY_DN10569_c0_g2/TRINITY_DN19224_c0_g1/TRINITY_DN9935_c1_g1/TRINITY_DN9041_c0_g1/TRINITY_DN2869_c2_g1/T	27

							RINITY_DN15420_c0_g1/TRINITY_DN7687_c0_g1/TRINITY_DN7200_c0_g2/TRINITY_DN3238_c0_g1/TRINITY_DN7523_c0_g1/TRINITY_DN9041_c0_g2/TRINITY_DN2094_c0_g1/TRINITY_DN428_c0_g1/TRINITY_DN7084_c0_g1/TRINITY_DN2961_c0_g1/TRINITY_DN6505_c0_g1/TRINITY_DN49433_c0_g1	
GO:0015277	kainate selective glutamate receptor activity	9/2626	13/33142	6.47×10^{-8}	2.76×10^{-6}	2.06×10^{-6}	TRINITY_DN21399_c0_g2/TRINITY_DN8355_c0_g1/TRINITY_DN1765_c2_g1/TRINITY_DN3816_c0_g1/TRINITY_DN9041_c0_g1/TRINITY_DN15420_c0_g1/TRINITY_DN7200_c0_g2/TRINITY_DN9041_c0_g2/TRINITY_DN21399_c0_g1	9
GO:0051967	negative regulation of synaptic transmission， glutamatergic	9/2626	13/33142	6.47×10^{-8}	2.76×10^{-6}	2.06×10^{-6}	TRINITY_DN21399_c0_g2/TRINITY_DN8355_c0_g1/TRINITY_DN22381_c0_g1/TRINITY_DN1765_c2_g1/TRINITY_DN3816_c0_g1/TRINITY_DN31435_c0_g1/TRINITY_DN9041_c0_g1/TRINITY_DN7200_c0_g2/TRINITY_DN9041_c0_g2	9
GO:0010842	retina layer formation	12/2626	24/33142	6.54×10^{-8}	2.76×10^{-6}	2.06×10^{-6}	TRINITY_DN3778_c2_g1/TRINITY_DN9219_c0_g1/TRINITY_DN51872_c0_g1/TRINITY_DN9375_c0_g1/TRINITY_DN12540_c0_g1/TRINITY_DN9630	12

							1_c0_g1/TRINITY_DN4474_c0_g1/TRINITY_DN9845_c0_g2/TRINITY_DN8217_c1_g1/TRINITY_DN15468_c0_g1/TRINITY_DN20346_c0_g1/TRINITY_DN49166_c0_g1	
GO:0042383	sarcolemma	19/2626	59/33142	7.14 × 10^{-8}	2.97 × 10^{-6}	2.22 × 10^{-6}	TRINITY_DN5216_c1_g1/TRINITY_DN2738_c0_g1/TRINITY_DN15486_c0_g2/TRINITY_DN7743_c0_g1/TRINITY_DN200_c0_g2/TRINITY_DN16407_c1_g1/TRINITY_DN740_c0_g1/TRINITY_DN3180_c0_g1/TRINITY_DN2598_c0_g2/TRINITY_DN11154_c0_g1/TRINITY_DN93_c0_g1/TRINITY_DN6245_c0_g1/TRINITY_DN30302_c0_g1/TRINITY_DN5506_c0_g3/TRINITY_DN55372_c0_g1/TRINITY_DN10587_c0_g1/TRINITY_DN12958_c0_g1/TRINITY_DN6945_c0_g2/TRINITY_DN18471_c0_g2	19
GO:0005765	lysosomal membrane	36/2626	174/33142	8.56 × 10^{-8}	3.50 × 10^{-6}	2.62 × 10^{-6}	TRINITY_DN7814_c0_g2/TRINITY_DN7437_c0_g1/TRINITY_DN8323_c0_g1/TRINITY_DN3446_c0_g1/TRINITY_DN72971_c0_g1/TRINITY_DN16936_c0_g1/TRINITY_DN8493_c0_g1/TRINITY_DN2078_c0_g1/TRINITY_DN43642_c0_g1/TRINITY_DN47_c0_g1/TRINITY_	36

							DN13394_c0_g1/TRINITY_DN200_c0_g2/TRINITY_DN8257_c0_g1/TRINITY_DN8433_c1_g2/TRINITY_DN27259_c0_g1/TRINITY_DN4826_c1_g1/TRINITY_DN1482_c0_g1/TRINITY_DN7858_c0_g1/TRINITY_DN9333_c0_g1/TRINITY_DN4477_c0_g1/TRINITY_DN8012_c0_g1/TRINITY_DN9845_c0_g2/TRINITY_DN13943_c0_g1/TRINITY_DN4437_c0_g1/TRINITY_DN7045_c1_g1/TRINITY_DN9994_c0_g1/TRINITY_DN8433_c1_g1/TRINITY_DN4307_c0_g1/TRINITY_DN6603_c0_g1/TRINITY_DN4554_c0_g1/TRINITY_DN18131_c0_g1/TRINITY_DN1482_c0_g2/TRINITY_DN3354_c0_g1/TRINITY_DN55372_c0_g1/TRINITY_DN273_c1_g1/TRINITY_DN4674_c0_g1	
GO:0045211	postsynaptic membrane	29/2626	124/33142	9.63×10^{-8}	3.88×10^{-6}	2.91×10^{-6}	TRINITY_DN21399_c0_g2/TRINITY_DN16006_c0_g1/TRINITY_DN9375_c0_g1/TRINITY_DN8355_c0_g1/TRINITY_DN2161_c0_g1/TRINITY_DN4958_c0_g1/TRINITY_DN1316_c0_g1/TRINITY_DN15308_c0_g2/TRINITY_DN1765_c2_g1/TRINITY_DN3816_c0_g1/TRINITY_DN19635_c0_g1/TRINITY_DN	29

							84830_c0_g1/TRINITY_DN13011_c0_g1/TRINITY_DN7054_c0_g1/TRINITY_DN9935_c1_g1/TRINITY_DN9041_c0_g1/TRINITY_DN6910_c0_g1/TRINITY_DN2869_c2_g1/TRINITY_DN2938_c3_g1/TRINITY_DN15420_c0_g1/TRINITY_DN7200_c0_g2/TRINITY_DN7523_c0_g1/TRINITY_DN9041_c0_g2/TRINITY_DN12650_c0_g1/TRINITY_DN428_c0_g1/TRINITY_DN4000_c0_g2/TRINITY_DN6505_c0_g1/TRINITY_DN49433_c0_g1/TRINITY_DN21399_c0_g1	
GO:0071353	cellular response to interleukin-4	12/2626	25/33142	1.17×10^{-7}	4.60×10^{-6}	3.44×10^{-6}	TRINITY_DN19001_c0_g1/TRINITY_DN23921_c0_g1/TRINITY_DN482_c1_g1/TRINITY_DN29856_c0_g1/TRINITY_DN1549_c0_g1/TRINITY_DN40503_c0_g1/TRINITY_DN4137_c2_g1/TRINITY_DN40189_c0_g1/TRINITY_DN484_c0_g1/TRINITY_DN244_c3_g1/TRINITY_DN5788_c1_g1/TRINITY_DN61025_c0_g1	12
GO:0032496	response to lipopolysaccharide	15/2626	39/33142	1.18×10^{-7}	4.60×10^{-6}	3.44×10^{-6}	TRINITY_DN3991_c0_g1/TRINITY_DN7384_c0_g1/TRINITY_DN1557_c0_g1/TRINITY_DN5216_c1_g1/TRINITY_DN42262_c0_g1/TRINITY_DN3007_	15

							c0_g1/TRINITY_DN2779_c1_g2/TRINITY_DN5951_c0_g4/TRINITY_DN13981_c0_g1/TRINITY_DN2682_c0_g1/TRINITY_DN974_c0_g1/TRINITY_DN2779_c1_g3/TRINITY_DN1118_c0_g1/TRINITY_DN12958_c0_g1/TRINITY_DN6589_c0_g1	
GO:0005216	ion channel activity	18/2626	55/33142	1.19×10^{-7}	4.60×10^{-6}	3.44×10^{-6}	TRINITY_DN6597_c0_g1/TRINITY_DN1748_c0_g1/TRINITY_DN8167_c0_g1/TRINITY_DN10654_c0_g1/TRINITY_DN6323_c1_g1/TRINITY_DN2924_c1_g1/TRINITY_DN16407_c1_g1/TRINITY_DN3180_c0_g1/TRINITY_DN16593_c0_g1/TRINITY_DN14953_c0_g3/TRINITY_DN17398_c0_g2/TRINITY_DN30782_c0_g2/TRINITY_DN2003_c0_g1/TRINITY_DN4264_c1_g1/TRINITY_DN35025_c0_g1/TRINITY_DN4522_c0_g1/TRINITY_DN7318_c0_g1/TRINITY_DN4784_c1_g1	18
GO:0001662	behavioral fear response	11/2626	21/33142	1.26×10^{-7}	4.82×10^{-6}	3.61×10^{-6}	TRINITY_DN21399_c0_g2/TRINITY_DN5239_c1_g2/TRINITY_DN2327_c0_g1/TRINITY_DN22381_c0_g1/TRINITY_DN1765_c2_g1/TRINITY_DN3816_c0_g1/TRINITY_DN31435_c0_g1/TRINITY_DN9041_c0_g1/TRINITY_DN7200_c0_g2/TRIN	11

							ITY_DN9041_c0_g2/TRINITY_DN1071_c0_g1	
GO:0015697	quaternary ammonium group transport	9/2626	14/33142	1.68 × 10^{-7}	6.26 × 10^{-6}	4.68 × 10^{-6}	TRINITY_DN2006_c0_g1/TRINITY_DN2770_c0_g1/TRINITY_DN1478_c1_g3/TRINITY_DN14468_c0_g1/TRINITY_DN10811_c0_g1/TRINITY_DN10798_c0_g1/TRINITY_DN11056_c1_g1/TRINITY_DN11061_c0_g2/TRINITY_DN216_c0_g3	9
GO:0060294	cilium movement involved in cell motility	9/2626	14/33142	1.68 × 10^{-7}	6.26 × 10^{-6}	4.68 × 10^{-6}	TRINITY_DN6657_c0_g1/TRINITY_DN19725_c0_g1/TRINITY_DN11686_c1_g1/TRINITY_DN7740_c0_g1/TRINITY_DN1979_c0_g2/TRINITY_DN6610_c0_g1/TRINITY_DN2725_c0_g1/TRINITY_DN59259_c0_g1/TRINITY_DN63431_c0_g1	9
GO:0048468	cell development	8/2626	11/33142	2.04 × 10^{-7}	7.41 × 10^{-6}	5.55 × 10^{-6}	TRINITY_DN9767_c0_g3/TRINITY_DN29267_c0_g2/TRINITY_DN37845_c0_g1/TRINITY_DN23921_c0_g1/TRINITY_DN1549_c0_g1/TRINITY_DN484_c0_g1/TRINITY_DN244_c3_g1/TRINITY_DN61025_c0_g1	8
GO:0043025	neuronal cell body	43/2626	235/33142	2.05 × 10^{-7}	7.41 × 10^{-6}	5.55 × 10^{-6}	TRINITY_DN21399_c0_g2/TRINITY_DN8323_c0_g1/TRINITY_DN3446_c0_g1/TRINITY_DN3245_c0_g1/TRINITY_DN1487_c0_g2/TRINITY_DN205_c0_g1/TRINITY_DN1557_c0_g1/TRINITY_DN16186_c0_g1/TRI	43

NITY_DN30087_c1_g2/TRINI
TY_DN13394_c0_g1/TRINITY
_DN200_c0_g2/TRINITY_DN1
7663_c0_g1/TRINITY_DN5781
_c0_g1/TRINITY_DN15030_c0
_g2/TRINITY_DN7131_c0_g1/
TRINITY_DN8012_c0_g1/TRI
NITY_DN3816_c0_g1/TRINIT
Y_DN800_c0_g2/TRINITY_D
N4620_c0_g1/TRINITY_DN36
65_c0_g1/TRINITY_DN9175_c
0_g1/TRINITY_DN8599_c1_g1
/TRINITY_DN9935_c1_g1/TRI
NITY_DN93_c0_g1/TRINITY_
DN2746_c0_g1/TRINITY_DN2
165_c3_g1/TRINITY_DN58254
_c0_g1/TRINITY_DN9158_c3_
g1/TRINITY_DN14953_c0_g3/
TRINITY_DN709_c0_g1/TRIN
ITY_DN15420_c0_g1/TRINITY
_DN7200_c0_g2/TRINITY_DN
11061_c0_g2/TRINITY_DN126
50_c0_g1/TRINITY_DN44537_
c0_g1/TRINITY_DN16530_c0_
g1/TRINITY_DN1186_c0_g1/T
RINITY_DN35025_c0_g1/TRI
NITY_DN1908_c0_g1/TRINIT
Y_DN2961_c0_g1/TRINITY_D
N6505_c0_g1/TRINITY_DN26
727_c0_g1/TRINITY_DN16754
_c0_g1

GO:0062023	collagen-containing extracellular matrix	22/2626	81/33142	2.07×10^{-7}	7.41×10^{-6}	5.55×10^{-6}	TRINITY_DN12244_c0_g1/TRINITY_DN5310_c0_g2/TRINITY_DN72971_c0_g1/TRINITY_DN6305_c1_g1/TRINITY_DN5613_c1_g1/TRINITY_DN2738_c0_g1/TRINITY_DN1533_c0_g2/TRINITY_DN1120_c0_g2/TRINITY_DN8217_c0_g1/TRINITY_DN636_c0_g1/TRINITY_DN4826_c1_g1/TRINITY_DN740_c0_g1/TRINITY_DN1902_c0_g1/TRINITY_DN2598_c0_g2/TRINITY_DN2868_c0_g1/TRINITY_DN35269_c0_g1/TRINITY_DN7326_c0_g1/TRINITY_DN9853_c0_g1/TRINITY_DN56782_c0_g1/TRINITY_DN2281_c0_g1/TRINITY_DN5908_c0_g1/TRINITY_DN4326_c0_g1	22
GO:0007399	nervous system development	35/2626	173/33142	2.28×10^{-7}	7.98×10^{-6}	5.97×10^{-6}	TRINITY_DN12244_c0_g1/TRINITY_DN3245_c0_g1/TRINITY_DN1487_c0_g2/TRINITY_DN3120_c0_g1/TRINITY_DN1695_c0_g1/TRINITY_DN17581_c0_g1/TRINITY_DN52390_c0_g1/TRINITY_DN4097_c0_g1/TRINITY_DN5176_c0_g1/TRINITY_DN1313_c0_g2/TRINITY_DN5680_c0_g1/TRINITY_DN2531_c0_g1/TRINITY_DN5951_c0_g4/TRINITY_DN3284_c0_g1/TRINITY_DN306_c1_g2/TRI	35

							NITY_DN8607_c0_g1/TRINITY_DN8681_c0_g1/TRINITY_DN130_c0_g1/TRINITY_DN23750_c0_g1/TRINITY_DN5276_c1_g1/TRINITY_DN10575_c2_g1/TRINITY_DN716_c0_g1/TRINITY_DN5362_c0_g1/TRINITY_DN3333_c0_g1/TRINITY_DN7006_c0_g1/TRINITY_DN3636_c0_g3/TRINITY_DN3636_c0_g4/TRINITY_DN7687_c0_g1/TRINITY_DN435_c2_g1/TRINITY_DN9041_c0_g2/TRINITY_DN1436_c1_g1/TRINITY_DN61_c0_g1/TRINITY_DN8449_c0_g1/TRINITY_DN29720_c0_g2/TRINITY_DN21399_c0_g1	
GO:0005330	dopamine:sodium symporter activity	10/2626	18/33142	2.32×10^{-7}	7.98×10^{-6}	5.97×10^{-6}	TRINITY_DN2006_c0_g1/TRINITY_DN18454_c0_g1/TRINITY_DN5871_c1_g1/TRINITY_DN1478_c1_g3/TRINITY_DN14468_c0_g1/TRINITY_DN10811_c0_g1/TRINITY_DN10798_c0_g1/TRINITY_DN11056_c1_g1/TRINITY_DN11061_c0_g2/TRINITY_DN24632_c0_g1	10
GO:0007193	adenylate cyclase-inhibiting G protein-coupled receptor signaling pathway	10/2626	18/33142	2.32×10^{-7}	7.98×10^{-6}	5.97×10^{-6}	TRINITY_DN72971_c0_g1/TRINITY_DN20653_c0_g1/TRINITY_DN4826_c1_g1/TRINITY_DN22381_c0_g1/TRINITY_DN31435_c0_g1/TRINITY_DN6839_c2_g1/TRINITY_DN3032_c1	10

							_g2/TRINITY_DN12650_c0_g1/TRINITY_DN43641_c0_g1/TRINITY_DN10344_c0_g1	
GO:0015101	organic cation transmembrane transporter activity	11/2626	22/33142	2.35×10^{-7}	7.98×10^{-6}	5.97×10^{-6}	TRINITY_DN2006_c0_g1/TRINITY_DN5871_c1_g1/TRINITY_DN19520_c0_g1/TRINITY_DN1478_c1_g3/TRINITY_DN14468_c0_g1/TRINITY_DN10811_c0_g1/TRINITY_DN10798_c0_g1/TRINITY_DN11056_c1_g1/TRINITY_DN11061_c0_g2/TRINITY_DN24632_c0_g1/TRINITY_DN8729_c1_g1	11
GO:0010951	negative regulation of endopeptidase activity	12/2626	27/33142	3.36×10^{-7}	1.13×10^{-5}	8.45×10^{-6}	TRINITY_DN5613_c1_g1/TRINITY_DN6082_c1_g2/TRINITY_DN16500_c1_g1/TRINITY_DN4861_c0_g1/TRINITY_DN3123_c0_g1/TRINITY_DN4603_c1_g1/TRINITY_DN1510_c0_g1/TRINITY_DN10648_c0_g1/TRINITY_DN3717_c0_g1/TRINITY_DN7326_c0_g1/TRINITY_DN56782_c0_g1/TRINITY_DN14093_c0_g1	12
GO:0042594	response to starvation	21/2626	77/33142	3.61×10^{-7}	1.20×10^{-5}	8.98×10^{-6}	TRINITY_DN7953_c1_g1/TRINITY_DN1015_c0_g2/TRINITY_DN39639_c3_g1/TRINITY_DN30262_c0_g1/TRINITY_DN7051_c0_g1/TRINITY_DN2378_c0_g1/TRINITY_DN5861_c0_g1/TRINITY_DN8848_c0_g1/TRINITY_DN3614_c1_g1/TRIN	21

							ITY_DN974_c0_g1/TRINITY_DN1486_c0_g1/TRINITY_DN12852_c0_g1/TRINITY_DN346_c0_g1/TRINITY_DN4859_c0_g1/TRINITY_DN9642_c1_g2/TRINITY_DN3032_c1_g2/TRINITY_DN9521_c0_g2/TRINITY_DN15536_c0_g1/TRINITY_DN43262_c0_g1/TRINITY_DN27661_c0_g1/TRINITY_DN864_c0_g3	
GO:0007156	homophilic cell adhesion via plasma membrane adhesion molecules	23/2626	90/33142	3.68×10^{-7}	1.21×10^{-5}	9.04×10^{-6}	TRINITY_DN5310_c0_g2/TRINITY_DN7785_c0_g1/TRINITY_DN6331_c0_g1/TRINITY_DN2244_c1_g1/TRINITY_DN6174_c0_g1/TRINITY_DN1990_c0_g1/TRINITY_DN9219_c0_g1/TRINITY_DN1120_c0_g2/TRINITY_DN2868_c0_g2/TRINITY_DN6076_c0_g1/TRINITY_DN6348_c0_g1/TRINITY_DN8217_c1_g1/TRINITY_DN43399_c0_g1/TRINITY_DN7898_c0_g1/TRINITY_DN27045_c0_g1/TRINITY_DN2475_c0_g1/TRINITY_DN12452_c0_g1/TRINITY_DN9158_c3_g1/TRINITY_DN446_c1_g1/TRINITY_DN709_c0_g1/TRINITY_DN2321_c0_g1/TRINITY_DN1941_c0_g1/TRINITY_DN13354_c0_g1	23

insects MDPI

GO:0008066	glutamate receptor activity	10/2626	19/33142	4.54 × 10^{-7}	1.46 × 10^{-5}	1.09 × 10^{-5}	TRINITY_DN21399_c0_g2/TRINITY_DN16006_c0_g1/TRINITY_DN8355_c0_g1/TRINITY_DN1765_c2_g1/TRINITY_DN3816_c0_g1/TRINITY_DN9041_c0_g1/TRINITY_DN7200_c0_g2/TRINITY_DN9041_c0_g2/TRINITY_DN49433_c0_g1/TRINITY_DN21399_c0_g1	10
GO:0030224	monocyte differentiation	10/2626	19/33142	4.54 × 10^{-7}	1.46 × 10^{-5}	1.09 × 10^{-5}	TRINITY_DN19001_c0_g1/TRINITY_DN23921_c0_g1/TRINITY_DN482_c1_g1/TRINITY_DN29856_c0_g1/TRINITY_DN1549_c0_g1/TRINITY_DN40189_c0_g1/TRINITY_DN484_c0_g1/TRINITY_DN10344_c0_g1/TRINITY_DN244_c3_g1/TRINITY_DN61025_c0_g1	10
GO:0005543	phospholipid binding	17/2626	54/33142	4.99 × 10^{-7}	1.58 × 10^{-5}	1.18 × 10^{-5}	TRINITY_DN72971_c0_g1/TRINITY_DN834_c1_g1/TRINITY_DN7048_c0_g1/TRINITY_DN15486_c0_g2/TRINITY_DN23953_c0_g1/TRINITY_DN4826_c1_g1/TRINITY_DN8977_c0_g1/TRINITY_DN9755_c0_g1/TRINITY_DN11938_c0_g1/TRINITY_DN656_c0_g1/TRINITY_DN9853_c0_g1/TRINITY_DN4859_c0_g1/TRINITY_DN10575_c2_g1/TRINITY_DN3890_c0_g1/TRINITY_DN43641_c0_g1/T	17

							RINITY_DN6505_c0_g1/TRINITY_DN17100_c0_g1	
GO:0007215	glutamate receptor signaling pathway	8/2626	12/33142	5.69×10^{-7}	1.77×10^{-5}	1.32×10^{-5}	TRINITY_DN21399_c0_g2/TRINITY_DN8355_c0_g1/TRINITY_DN1765_c2_g1/TRINITY_DN9041_c0_g1/TRINITY_DN55372_c0_g1/TRINITY_DN7200_c0_g2/TRINITY_DN9041_c0_g2/TRINITY_DN21399_c0_g1	8
GO:0035082	axoneme assembly	8/2626	12/33142	5.69×10^{-7}	1.77×10^{-5}	1.32×10^{-5}	TRINITY_DN7740_c0_g1/TRINITY_DN5006_c0_g2/TRINITY_DN7530_c0_g1/TRINITY_DN2153_c0_g1/TRINITY_DN15544_c0_g1/TRINITY_DN4533_c4_g1/TRINITY_DN59259_c0_g1/TRINITY_DN63431_c0_g1	8
GO:0014070	response to organic cyclic compound	13/2626	33/33142	5.95×10^{-7}	1.82×10^{-5}	1.37×10^{-5}	TRINITY_DN32009_c0_g1/TRINITY_DN11954_c0_g1/TRINITY_DN1568_c0_g1/TRINITY_DN406_c0_g3/TRINITY_DN7743_c0_g1/TRINITY_DN5049_c0_g1/TRINITY_DN2999_c0_g1/TRINITY_DN3713_c1_g1/TRINITY_DN6037_c0_g1/TRINITY_DN406_c0_g2/TRINITY_DN15324_c1_g1/TRINITY_DN6985_c0_g1/TRINITY_DN42565_c0_g1	13
GO:0007275	multicellular organism development	58/2626	373/33142	6.14×10^{-7}	1.86×10^{-5}	1.40×10^{-5}	TRINITY_DN2677_c0_g1/TRINITY_DN2639_c0_g1/TRINITY_DN9943_c0_g1/TRINITY_DN2841_c0_g1/TRINITY_DN59	58

02_c1_g1/TRINITY_DN11509_
c0_g1/TRINITY_DN8299_c0_g
1/TRINITY_DN9643_c0_g1/TR
INITY_DN2276_c0_g1/TRINIT
Y_DN15863_c0_g1/TRINITY_
DN5878_c0_g1/TRINITY_DN2
648_c0_g1/TRINITY_DN5239_
c1_g2/TRINITY_DN7756_c0_g
1/TRINITY_DN5049_c0_g1/TR
INITY_DN2778_c0_g3/TRINIT
Y_DN3090_c1_g1/TRINITY_D
N17487_c0_g1/TRINITY_DN2
1912_c0_g1/TRINITY_DN4811
_c0_g1/TRINITY_DN2999_c0_
g1/TRINITY_DN35198_c0_g1/
TRINITY_DN6323_c1_g1/TRI
NITY_DN6222_c0_g1/TRINIT
Y_DN10609_c0_g1/TRINITY_
DN16331_c0_g1/TRINITY_DN
5327_c0_g1/TRINITY_DN9233
_c0_g1/TRINITY_DN8796_c0_
g1/TRINITY_DN85_c0_g1/TRI
NITY_DN7744_c0_g1/TRINIT
Y_DN12886_c0_g1/TRINITY_
DN49948_c0_g1/TRINITY_DN
5875_c0_g1/TRINITY_DN2615
_c0_g2/TRINITY_DN4173_c0_
g1/TRINITY_DN3180_c0_g1/T
RINITY_DN9676_c0_g1/TRIN
ITY_DN18049_c0_g1/TRINITY
_DN1998_c0_g1/TRINITY_DN
3058_c0_g1/TRINITY_DN7512

							_c0_g1/TRINITY_DN39858_c0_g1/TRINITY_DN12402_c0_g1/TRINITY_DN25570_c0_g1/TRINITY_DN4005_c0_g1/TRINITY_DN77356_c0_g1/TRINITY_DN12133_c0_g1/TRINITY_DN34504_c0_g1/TRINITY_DN33662_c0_g1/TRINITY_DN51821_c0_g1/TRINITY_DN4963_c0_g1/TRINITY_DN3459_c0_g1/TRINITY_DN6230_c4_g1/TRINITY_DN3241_c0_g1/TRINITY_DN30782_c0_g2/TRINITY_DN9055_c0_g2/TRINITY_DN674_c2_g1	
GO:0015771	trehalose transport	19/2626	67/33142	6.67×10^{-7}	2.00×10^{-5}	1.50×10^{-5}	TRINITY_DN15648_c3_g1/TRINITY_DN950_c2_g2/TRINITY_DN24938_c0_g1/TRINITY_DN15759_c0_g1/TRINITY_DN27949_c0_g1/TRINITY_DN33728_c0_g1/TRINITY_DN2995_c1_g1/TRINITY_DN8156_c0_g1/TRINITY_DN39203_c0_g2/TRINITY_DN27227_c0_g1/TRINITY_DN59776_c0_g1/TRINITY_DN20880_c0_g1/TRINITY_DN27273_c0_g1/TRINITY_DN6365_c0_g1/TRINITY_DN4204_c3_g1/TRINITY_DN34_c4_g1/TRINITY_DN6567_c0_g1/TRINITY_DN13085_c0_g1/TRINITY_DN11476_c0_g1	19

insects MDPI

GO:0042632	cholesterol homeostasis	15/2626	44/33142	7.36 × 10^{-7}	2.19 × 10^{-5}	1.64 × 10^{-5}	TRINITY_DN1568_c0_g1/TRINITY_DN5364_c0_g1/TRINITY_DN7048_c0_g1/TRINITY_DN5086_c0_g1/TRINITY_DN5278_c0_g1/TRINITY_DN10757_c0_g1/TRINITY_DN7045_c1_g1/TRINITY_DN9063_c0_g1/TRINITY_DN40503_c0_g1/TRINITY_DN26376_c0_g1/TRINITY_DN489_c1_g1/TRINITY_DN4137_c2_g1/TRINITY_DN333_c0_g1/TRINITY_DN38748_c0_g1/TRINITY_DN8292_c0_g1	15
GO:0032839	dendrite cytoplasm	9/2626	16/33142	8.31 × 10^{-7}	2.42 × 10^{-5}	1.81 × 10^{-5}	TRINITY_DN21399_c0_g2/TRINITY_DN8355_c0_g1/TRINITY_DN1765_c2_g1/TRINITY_DN3816_c0_g1/TRINITY_DN9041_c0_g1/TRINITY_DN7200_c0_g2/TRINITY_DN9041_c0_g2/TRINITY_DN9730_c0_g1/TRINITY_DN6505_c0_g1	9
GO:0008283	cell population proliferation	32/2626	159/33142	8.32 × 10^{-7}	2.42 × 10^{-5}	1.81 × 10^{-5}	TRINITY_DN3991_c0_g1/TRINITY_DN7437_c0_g1/TRINITY_DN21789_c0_g1/TRINITY_DN219_c3_g1/TRINITY_DN1990_c0_g1/TRINITY_DN5878_c0_g1/TRINITY_DN1266_c0_g1/TRINITY_DN2428_c0_g1/TRINITY_DN3296_c0_g1/TRINITY_DN19324_c0_g1/TRINITY_DN8903_c0_g1/TRINITY_DN2557_c0_g2/TRINITY_DN85_c0	32

insects MDPI

							_g1/TRINITY_DN1541_c0_g1/TRINITY_DN1902_c0_g1/TRINITY_DN2682_c0_g1/TRINITY_DN13467_c0_g1/TRINITY_DN1117_c1_g1/TRINITY_DN24147_c0_g1/TRINITY_DN8625_c0_g1/TRINITY_DN13564_c0_g1/TRINITY_DN9994_c0_g1/TRINITY_DN15457_c0_g1/TRINITY_DN11156_c0_g5/TRINITY_DN1699_c1_g1/TRINITY_DN4190_c0_g1/TRINITY_DN51821_c0_g1/TRINITY_DN26376_c0_g1/TRINITY_DN2281_c0_g1/TRINITY_DN645_c0_g1/TRINITY_DN4839_c0_g1/TRINITY_DN7633_c0_g1	
GO:0043170	macromolecule metabolic process	10/2626	20/33142	8.44×10^{-7}	2.43×10^{-5}	1.82×10^{-5}	TRINITY_DN28084_c0_g1/TRINITY_DN19001_c0_g1/TRINITY_DN23921_c0_g1/TRINITY_DN482_c1_g1/TRINITY_DN29856_c0_g1/TRINITY_DN1549_c0_g1/TRINITY_DN40189_c0_g1/TRINITY_DN484_c0_g1/TRINITY_DN244_c3_g1/TRINITY_DN61025_c0_g1	10
GO:0050896	response to stimulus	19/2626	68/33142	8.57×10^{-7}	2.44×10^{-5}	1.83×10^{-5}	TRINITY_DN6305_c1_g1/TRINITY_DN15240_c0_g1/TRINITY_DN7582_c0_g1/TRINITY_DN6134_c0_g1/TRINITY_DN7048_c0_g1/TRINITY_DN42941_c0_g1/TRINITY_DN1120_c0_	19

							g2/TRINITY_DN5198_c0_g1/TRINITY_DN2492_c0_g1/TRINITY_DN8217_c0_g1/TRINITY_DN2924_c1_g1/TRINITY_DN5459_c0_g1/TRINITY_DN2868_c0_g1/TRINITY_DN25570_c0_g1/TRINITY_DN5121_c0_g1/TRINITY_DN4264_c1_g1/TRINITY_DN61_c0_g1/TRINITY_DN4784_c1_g1/TRINITY_DN16662_c0_g1	
GO:0005319	lipid transporter activity	13/2626	34/33142	8.93×10^{-7}	2.50×10^{-5}	1.87×10^{-5}	TRINITY_DN11463_c0_g1/TRINITY_DN34083_c0_g1/TRINITY_DN60341_c0_g1/TRINITY_DN7425_c6_g1/TRINITY_DN12443_c0_g1/TRINITY_DN8493_c0_g1/TRINITY_DN7048_c0_g1/TRINITY_DN32249_c0_g1/TRINITY_DN5278_c0_g1/TRINITY_DN383_c0_g1/TRINITY_DN46257_c0_g1/TRINITY_DN7045_c1_g1/TRINITY_DN35596_c0_g1	13
GO:0007286	spermatid development	13/2626	34/33142	8.93×10^{-7}	2.50×10^{-5}	1.87×10^{-5}	TRINITY_DN9943_c0_g1/TRINITY_DN2841_c0_g1/TRINITY_DN23053_c0_g1/TRINITY_DN15863_c0_g1/TRINITY_DN4544_c0_g1/TRINITY_DN954_c0_g1/TRINITY_DN6380_c0_g1/TRINITY_DN9994_c0_g1/TRINITY_DN93_c0_g1/TRINITY_DN7067_c0_g1/TRINITY_DN	13

							9809_c0_g3/TRINITY_DN686_c0_g2/TRINITY_DN39_c1_g2	
GO:0016758	transferase activity， transferring hexosyl groups	20/2626	75/33142	1.01×10^{-6}	2.79×10^{-5}	2.09×10^{-5}	TRINITY_DN32009_c0_g1/TRINITY_DN16979_c0_g1/TRINITY_DN11954_c0_g1/TRINITY_DN638_c0_g2/TRINITY_DN50797_c0_g3/TRINITY_DN23485_c0_g1/TRINITY_DN15702_c0_g2/TRINITY_DN15487_c0_g1/TRINITY_DN5087_c0_g1/TRINITY_DN10011_c0_g1/TRINITY_DN4160_c0_g1/TRINITY_DN10583_c0_g1/TRINITY_DN974_c0_g1/TRINITY_DN15702_c0_g4/TRINITY_DN15324_c1_g1/TRINITY_DN13235_c0_g1/TRINITY_DN13226_c0_g1/TRINITY_DN1200_c2_g1/TRINITY_DN8845_c0_g3/TRINITY_DN27558_c0_g1	20
GO:0015574	trehalose transmembrane transporter activity	18/2626	63/33142	1.16×10^{-6}	3.17×10^{-5}	2.37×10^{-5}	TRINITY_DN15648_c3_g1/TRINITY_DN950_c2_g2/TRINITY_DN24938_c0_g1/TRINITY_DN15759_c0_g1/TRINITY_DN27949_c0_g1/TRINITY_DN33728_c0_g1/TRINITY_DN2995_c1_g1/TRINITY_DN39203_c0_g2/TRINITY_DN27227_c0_g1/TRINITY_DN59776_c0_g1/TRINITY_DN20880_c0_g1/TRINITY_DN27273_c0_g1/TRINITY_DN6365_c0_g1/TRINITY_DN42	18

							04_c3_g1/TRINITY_DN34_c4_g1/TRINITY_DN6567_c0_g1/TRINITY_DN13085_c0_g1/TRINITY_DN11476_c0_g1	
GO:0001822	kidney development	13/2626	35/33142	1.32 × 10^{-6}	3.58 × 10^{-5}	2.68 × 10^{-5}	TRINITY_DN3991_c0_g1/TRINITY_DN950_c4_g1/TRINITY_DN5216_c1_g1/TRINITY_DN6886_c3_g1/TRINITY_DN3762_c0_g1/TRINITY_DN13406_c0_g1/TRINITY_DN306_c1_g2/TRINITY_DN2682_c0_g1/TRINITY_DN6745_c0_g1/TRINITY_DN9168_c0_g2/TRINITY_DN2011_c0_g1/TRINITY_DN52030_c0_g1/TRINITY_DN4416_c0_g1	13
GO:0006583	melanin biosynthetic process from tyrosine	10/2626	21/33142	1.50 × 10^{-6}	3.99 × 10^{-5}	2.98 × 10^{-5}	TRINITY_DN14689_c0_g1/TRINITY_DN2778_c1_g1/TRINITY_DN1229_c0_g2/TRINITY_DN55476_c0_g1/TRINITY_DN18821_c0_g1/TRINITY_DN70025_c0_g1/TRINITY_DN1229_c0_g4/TRINITY_DN1229_c0_g3/TRINITY_DN855_c0_g1/TRINITY_DN4947_c0_g1	10
GO:0019731	antibacterial humoral response	10/2626	21/33142	1.50 × 10^{-6}	3.99 × 10^{-5}	2.98 × 10^{-5}	TRINITY_DN33530_c0_g1/TRINITY_DN15601_c0_g3/TRINITY_DN945_c0_g1/TRINITY_DN6944_c0_g1/TRINITY_DN3487_c1_g1/TRINITY_DN10831_c0_g1/TRINITY_DN972_c0_g1/TRINITY_DN15618_c0_g1/TR	10

							INITY_DN16383_c1_g1/TRINITY_DN15618_c0_g2	
GO:0006464	cellular protein modification process	16/2626	52/33142	1.52 × 10^{-6}	3.99 × 10^{-5}	2.99 × 10^{-5}	TRINITY_DN1936_c0_g1/TRINITY_DN879_c0_g2/TRINITY_DN6049_c1_g2/TRINITY_DN4759_c0_g1/TRINITY_DN3981_c0_g1/TRINITY_DN9948_c0_g1/TRINITY_DN2415_c2_g1/TRINITY_DN16168_c0_g1/TRINITY_DN1406_c0_g1/TRINITY_DN8866_c0_g1/TRINITY_DN15133_c0_g1/TRINITY_DN489_c2_g1/TRINITY_DN9716_c0_g1/TRINITY_DN15544_c0_g1/TRINITY_DN21292_c0_g1/TRINITY_DN7633_c0_g1	16
GO:0006915	apoptotic process	38/2626	211/33142	1.53 × 10^{-6}	3.99 × 10^{-5}	2.99 × 10^{-5}	TRINITY_DN755_c2_g1/TRINITY_DN3772_c0_g1/TRINITY_DN6327_c1_g1/TRINITY_DN3120_c0_g1/TRINITY_DN1557_c0_g1/TRINITY_DN2279_c0_g1/TRINITY_DN4385_c0_g1/TRINITY_DN5086_c0_g1/TRINITY_DN10707_c0_g1/TRINITY_DN17487_c0_g1/TRINITY_DN1169_c0_g1/TRINITY_DN6947_c0_g1/TRINITY_DN7712_c0_g1/TRINITY_DN36153_c0_g3/TRINITY_DN14468_c0_g1/TRINITY_DN7744_c0_g1/TRINITY_DN7659_c0_g1/TRINITY_DN10783_c0_g1/TRINITY_DN1	38

insects

MDPI

							797_c0_g1/TRINITY_DN5157_c0_g2/TRINITY_DN10811_c0_g1/TRINITY_DN10145_c1_g1/TRINITY_DN520_c0_g1/TRINITY_DN3321_c0_g1/TRINITY_DN32077_c0_g1/TRINITY_DN93_c0_g1/TRINITY_DN1699_c1_g1/TRINITY_DN20528_c0_g1/TRINITY_DN11886_c0_g1/TRINITY_DN9786_c0_g1/TRINITY_DN51821_c0_g1/TRINITY_DN4326_c0_g1/TRINITY_DN3238_c0_g1/TRINITY_DN3030_c0_g1/TRINITY_DN4137_c2_g1/TRINITY_DN6505_c0_g1/TRINITY_DN7059_c0_g1/TRINITY_DN5788_c1_g1	
GO:0042587	glycogen granule	9/2626	17/33142	1.64×10^{-6}	4.25×10^{-5}	3.18×10^{-5}	TRINITY_DN19001_c0_g1/TRINITY_DN23921_c0_g1/TRINITY_DN482_c1_g1/TRINITY_DN29856_c0_g1/TRINITY_DN1549_c0_g1/TRINITY_DN40189_c0_g1/TRINITY_DN484_c0_g1/TRINITY_DN244_c3_g1/TRINITY_DN61025_c0_g1	9
GO:0003777	microtubule motor activity	22/2626	91/33142	1.81×10^{-6}	4.64×10^{-5}	3.48×10^{-5}	TRINITY_DN19161_c0_g1/TRINITY_DN9451_c0_g1/TRINITY_DN1979_c0_g2/TRINITY_DN1265_c0_g1/TRINITY_DN5811_c0_g1/TRINITY_DN1979_c0_g1/TRINITY_DN4543_c0_g2/TRINITY_DN3083_c0_g1/TRI	22

insects
MDPI

							NITY_DN18134_c0_g1/TRINITY_DN1909_c0_g1/TRINITY_DN3767_c0_g1/TRINITY_DN10356_c0_g1/TRINITY_DN18791_c0_g1/TRINITY_DN5968_c0_g1/TRINITY_DN12133_c0_g1/TRINITY_DN35591_c0_g1/TRINITY_DN31689_c0_g1/TRINITY_DN6282_c0_g1/TRINITY_DN9647_c0_g1/TRINITY_DN5922_c0_g1/TRINITY_DN5578_c0_g1/TRINITY_DN13780_c0_g1	
GO:0031406	carboxylic acid binding	7/2626	10/33142	1.88×10^{-6}	4.75×10^{-5}	3.56×10^{-5}	TRINITY_DN15486_c0_g2/TRINITY_DN10137_c0_g1/TRINITY_DN18587_c0_g2/TRINITY_DN1610_c0_g1/TRINITY_DN15536_c0_g1/TRINITY_DN6300_c0_g1/TRINITY_DN4895_c1_g1	7
GO:0036156	inner dynein arm	7/2626	10/33142	1.88×10^{-6}	4.75×10^{-5}	3.56×10^{-5}	TRINITY_DN1979_c0_g2/TRINITY_DN1979_c0_g1/TRINITY_DN3083_c0_g1/TRINITY_DN1979_c1_g1/TRINITY_DN15113_c0_g1/TRINITY_DN10356_c0_g1/TRINITY_DN29956_c0_g1	7
GO:0005230	extracellular ligand-gated ion channel activity	11/2626	26/33142	1.91×10^{-6}	4.76×10^{-5}	3.57×10^{-5}	TRINITY_DN5378_c1_g1/TRINITY_DN3654_c0_g1/TRINITY_DN4958_c0_g1/TRINITY_DN1316_c0_g1/TRINITY_DN19635_c0_g1/TRINITY_DN13011	11

							_c0_g1/TRINITY_DN7054_c0_g1/TRINITY_DN6655_c0_g1/TRINITY_DN2938_c3_g1/TRINITY_DN4757_c0_g1/TRINITY_DN4000_c0_g2	
GO:0045121	membrane raft	20/2626	78/33142	1.97 × 10^{-6}	4.88 × 10^{-5}	3.65 × 10^{-5}	TRINITY_DN17778_c0_g1/TRINITY_DN1487_c0_g2/TRINITY_DN6931_c0_g1/TRINITY_DN18454_c0_g1/TRINITY_DN1557_c0_g1/TRINITY_DN7048_c0_g1/TRINITY_DN4514_c0_g1/TRINITY_DN200_c0_g2/TRINITY_DN16407_c1_g1/TRINITY_DN18051_c0_g1/TRINITY_DN17565_c0_g1/TRINITY_DN1852_c2_g1/TRINITY_DN6839_c2_g1/TRINITY_DN6910_c0_g1/TRINITY_DN4326_c0_g1/TRINITY_DN12650_c0_g1/TRINITY_DN7944_c0_g1/TRINITY_DN2285_c0_g1/TRINITY_DN14134_c0_g1/TRINITY_DN8692_c0_g1	20
GO:0045505	dynein intermediate chain binding	17/2626	59/33142	2.00 × 10^{-6}	4.90 × 10^{-5}	3.67 × 10^{-5}	TRINITY_DN9451_c0_g1/TRINITY_DN1979_c0_g2/TRINITY_DN552_c0_g1/TRINITY_DN1979_c0_g1/TRINITY_DN4543_c0_g2/TRINITY_DN3083_c0_g1/TRINITY_DN1979_c1_g1/TRINITY_DN4208_c0_g1/TRINITY_DN18134_c0_g1/TRINITY_DN10356_c0_g1/TRINITY	17

							_DN436_c2_g1/TRINITY_DN31689_c0_g1/TRINITY_DN6282_c0_g1/TRINITY_DN9647_c0_g1/TRINITY_DN29956_c0_g1/TRINITY_DN39491_c0_g1/TRINITY_DN15080_c0_g1	
GO:0006874	cellular calcium ion homeostasis	16/2626	53/33142	2.01×10^{-6}	4.90×10^{-5}	3.67×10^{-5}	TRINITY_DN21399_c0_g2/TRINITY_DN3388_c0_g1/TRINITY_DN17739_c0_g2/TRINITY_DN31967_c0_g1/TRINITY_DN7131_c0_g1/TRINITY_DN3180_c0_g1/TRINITY_DN1765_c2_g1/TRINITY_DN3816_c0_g1/TRINITY_DN9041_c0_g1/TRINITY_DN6603_c0_g1/TRINITY_DN982_c0_g1/TRINITY_DN7200_c0_g2/TRINITY_DN11061_c0_g2/TRINITY_DN9041_c0_g2/TRINITY_DN2197_c0_g1/TRINITY_DN18471_c0_g2	16
GO:0008340	determination of adult lifespan	24/2626	106/33142	2.21×10^{-6}	5.32×10^{-5}	3.98×10^{-5}	TRINITY_DN77781_c0_g1/TRINITY_DN10721_c0_g1/TRINITY_DN39639_c3_g1/TRINITY_DN7051_c0_g1/TRINITY_DN60483_c1_g1/TRINITY_DN5861_c0_g1/TRINITY_DN11422_c0_g1/TRINITY_DN3295_c0_g3/TRINITY_DN3065_c1_g1/TRINITY_DN1267_c0_g2/TRINITY_DN11704_c0_g1/TRINITY_DN1486_c0_g1/TRINITY_DN12852_c0_g1/TRINITY_DN346_	24

							c0_g1/TRINITY_DN16751_c0_g1/TRINITY_DN9642_c1_g2/TRINITY_DN953_c0_g1/TRINITY_DN6745_c0_g1/TRINITY_DN3595_c0_g1/TRINITY_DN4700_c0_g1/TRINITY_DN43262_c0_g1/TRINITY_DN33069_c0_g1/TRINITY_DN864_c0_g3/TRINITY_DN3295_c0_g1	
GO:0080030	methyl indole-3-acetate esterase activity	18/2626	66/33142	2.43×10^{-6}	5.81×10^{-5}	4.35×10^{-5}	TRINITY_DN9635_c0_g2/TRINITY_DN1429_c0_g1/TRINITY_DN9635_c0_g1/TRINITY_DN3521_c0_g1/TRINITY_DN13673_c0_g1/TRINITY_DN20279_c0_g1/TRINITY_DN13985_c0_g1/TRINITY_DN6946_c0_g1/TRINITY_DN39313_c0_g1/TRINITY_DN9545_c0_g1/TRINITY_DN638_c1_g1/TRINITY_DN59975_c0_g1/TRINITY_DN7783_c0_g2/TRINITY_DN2780_c2_g1/TRINITY_DN6194_c0_g1/TRINITY_DN5738_c0_g1/TRINITY_DN5274_c0_g1/TRINITY_DN7348_c1_g2	18
GO:0050885	neuromuscular process controlling balance	10/2626	22/33142	2.55×10^{-6}	6.01×10^{-5}	4.50×10^{-5}	TRINITY_DN72971_c0_g1/TRINITY_DN17778_c0_g1/TRINITY_DN20653_c0_g1/TRINITY_DN28373_c0_g1/TRINITY_DN9375_c0_g1/TRINITY_DN4826_c1_g1/TRINITY_DN25715_c0_g1/TRINITY_DN6839_c2_g	10

							1/TRINITY_DN9027_c0_g2/TRINITY_DN43641_c0_g1	
GO:0003779	actin binding	40/2626	232/33142	2.55 × 10^{-6}	6.01 × 10^{-5}	4.50 × 10^{-5}	TRINITY_DN3991_c0_g1/TRINITY_DN6440_c0_g1/TRINITY_DN6256_c0_g1/TRINITY_DN3245_c0_g1/TRINITY_DN3740_c1_g1/TRINITY_DN18454_c0_g1/TRINITY_DN47_c0_g1/TRINITY_DN954_c0_g1/TRINITY_DN6222_c0_g1/TRINITY_DN16954_c0_g1/TRINITY_DN2531_c0_g1/TRINITY_DN233_c0_g1/TRINITY_DN3732_c0_g1/TRINITY_DN7759_c0_g1/TRINITY_DN5519_c0_g1/TRINITY_DN5459_c0_g1/TRINITY_DN4441_c3_g1/TRINITY_DN2424_c0_g1/TRINITY_DN6081_c1_g2/TRINITY_DN2682_c0_g1/TRINITY_DN130_c0_g1/TRINITY_DN11154_c0_g1/TRINITY_DN8504_c0_g1/TRINITY_DN10575_c2_g1/TRINITY_DN5362_c0_g1/TRINITY_DN898_c0_g1/TRINITY_DN1174_c0_g1/TRINITY_DN2869_c2_g1/TRINITY_DN30302_c0_g1/TRINITY_DN3974_c0_g2/TRINITY_DN342_c0_g1/TRINITY_DN428_c0_g1/TRINITY_DN1186_c0_g1/TRINITY_DN2347_c1_g1/TRINITY_DN6505_c0_g1/TRI	40

							NITY_DN3437_c0_g1/TRINITY_DN5922_c0_g1/TRINITY_DN269_c0_g1/TRINITY_DN6541_c1_g2/TRINITY_DN4487_c3_g2	
GO:0002576	platelet degranulation	11/2626	27/33142	2.99 × 10^{-6}	6.83 × 10^{-5}	5.11 × 10^{-5}	TRINITY_DN72971_c0_g1/TRINITY_DN406_c0_g3/TRINITY_DN15748_c0_g1/TRINITY_DN5049_c0_g1/TRINITY_DN2999_c0_g1/TRINITY_DN4826_c1_g1/TRINITY_DN406_c0_g2/TRINITY_DN56782_c0_g1/TRINITY_DN7713_c1_g1/TRINITY_DN5675_c0_g1/TRINITY_DN42565_c0_g1	11
GO:0048786	presynaptic active zone	11/2626	27/33142	2.99 × 10^{-6}	6.83 × 10^{-5}	5.11 × 10^{-5}	TRINITY_DN1748_c0_g1/TRINITY_DN31967_c0_g1/TRINITY_DN11938_c0_g1/TRINITY_DN1420_c0_g1/TRINITY_DN4620_c0_g1/TRINITY_DN4029_c0_g1/TRINITY_DN5234_c0_g1/TRINITY_DN11061_c0_g2/TRINITY_DN8343_c0_g1/TRINITY_DN4674_c0_g1/TRINITY_DN49433_c0_g1	11
GO:0098595	perivitelline space	8/2626	14/33142	2.99 × 10^{-6}	6.83 × 10^{-5}	5.11 × 10^{-5}	TRINITY_DN6790_c0_g1/TRINITY_DN1350_c0_g2/TRINITY_DN3312_c0_g1/TRINITY_DN10159_c1_g1/TRINITY_DN6641_c0_g2/TRINITY_DN6641_c0_g1/TRINITY_DN3717_c0_g1/TRINITY_DN5802_c0_g1	8

GO:0016339	calcium-dependent cell-cell adhesion via plasma membrane cell adhesion molecules	12/2626	32/33142	3.00×10^{-6}	6.83×10^{-5}	5.11×10^{-5}	TRINITY_DN7785_c0_g1/TRINITY_DN6331_c0_g1/TRINITY_DN2244_c1_g1/TRINITY_DN6174_c0_g1/TRINITY_DN1990_c0_g1/TRINITY_DN6348_c0_g1/TRINITY_DN43399_c0_g1/TRINITY_DN27045_c0_g1/TRINITY_DN2475_c0_g1/TRINITY_DN446_c1_g1/TRINITY_DN7687_c0_g1/TRINITY_DN2321_c0_g1	12
GO:0030223	neutrophil differentiation	9/2626	18/33142	3.05×10^{-6}	6.89×10^{-5}	5.16×10^{-5}	TRINITY_DN19001_c0_g1/TRINITY_DN23921_c0_g1/TRINITY_DN482_c1_g1/TRINITY_DN29856_c0_g1/TRINITY_DN1549_c0_g1/TRINITY_DN40189_c0_g1/TRINITY_DN484_c0_g1/TRINITY_DN244_c3_g1/TRINITY_DN61025_c0_g1	9
GO:0008611	ether lipid biosynthetic process	14/2626	43/33142	3.22×10^{-6}	7.21×10^{-5}	5.40×10^{-5}	TRINITY_DN2997_c0_g1/TRINITY_DN8066_c0_g1/TRINITY_DN57502_c0_g1/TRINITY_DN19001_c0_g1/TRINITY_DN23921_c0_g1/TRINITY_DN482_c1_g1/TRINITY_DN29856_c0_g1/TRINITY_DN6810_c0_g1/TRINITY_DN1549_c0_g1/TRINITY_DN1450_c0_g3/TRINITY_DN40189_c0_g1/TRINITY_DN484_c0_g1/TRINITY_DN244_c3_g1/TRINITY_DN61025_c0_g1	14

insects MDPI

GO:0030165	PDZ domain binding	15/2626	49/33142	3.45 × 10^{-6}	7.67 × 10^{-5}	5.74 × 10^{-5}	TRINITY_DN21399_c0_g2/TRINITY_DN1765_c2_g1/TRINITY_DN1420_c0_g1/TRINITY_DN3816_c0_g1/TRINITY_DN9041_c0_g1/TRINITY_DN22905_c0_g1/TRINITY_DN11886_c0_g1/TRINITY_DN3032_c1_g2/TRINITY_DN30302_c0_g1/TRINITY_DN7687_c0_g1/TRINITY_DN7200_c0_g2/TRINITY_DN9041_c0_g2/TRINITY_DN216_c0_g3/TRINITY_DN1941_c0_g1/TRINITY_DN10346_c1_g1	15
GO:0009617	response to bacterium	28/2626	139/33142	3.86 × 10^{-6}	8.52 × 10^{-5}	6.38 × 10^{-5}	TRINITY_DN5310_c0_g2/TRINITY_DN6481_c0_g1/TRINITY_DN6342_c6_g1/TRINITY_DN14550_c0_g1/TRINITY_DN24518_c0_g1/TRINITY_DN1120_c0_g2/TRINITY_DN8330_c0_g1/TRINITY_DN15785_c0_g1/TRINITY_DN51904_c0_g1/TRINITY_DN1696_c0_g3/TRINITY_DN3916_c0_g1/TRINITY_DN4211_c1_g3/TRINITY_DN4477_c0_g1/TRINITY_DN28084_c0_g1/TRINITY_DN8330_c0_g2/TRINITY_DN7030_c0_g1/TRINITY_DN7326_c0_g1/TRINITY_DN18499_c0_g1/TRINITY_DN15289_c0_g1/TRINITY_DN10846_c0_g1/TRINITY_DN338_c0_g1/TRINITY_DN3238_c0_	28

							g1/TRINITY_DN13165_c0_g1/TRINITY_DN2058_c0_g5/TRINITY_DN637_c0_g1/TRINITY_DN11595_c0_g1/TRINITY_DN11595_c0_g2/TRINITY_DN4674_c0_g1	
GO:0008063	Toll signaling pathway	10/2626	23/33142	4.19×10^{-6}	9.16×10^{-5}	6.86×10^{-5}	TRINITY_DN22536_c0_g1/TRINITY_DN10890_c0_g1/TRINITY_DN15335_c0_g1/TRINITY_DN10159_c1_g1/TRINITY_DN14429_c0_g1/TRINITY_DN6641_c0_g2/TRINITY_DN6641_c0_g1/TRINITY_DN3717_c0_g1/TRINITY_DN2292_c2_g1/TRINITY_DN5802_c0_g1	10
GO:0007005	mitochondrion organization	19/2626	75/33142	4.28×10^{-6}	9.30×10^{-5}	6.97×10^{-5}	TRINITY_DN59294_c0_g1/TRINITY_DN17581_c0_g1/TRINITY_DN11896_c0_g2/TRINITY_DN542_c0_g2/TRINITY_DN36153_c0_g3/TRINITY_DN1201_c3_g1/TRINITY_DN10873_c0_g1/TRINITY_DN2327_c0_g1/TRINITY_DN21082_c0_g1/TRINITY_DN13138_c0_g1/TRINITY_DN9780_c0_g1/TRINITY_DN6745_c0_g1/TRINITY_DN15536_c0_g1/TRINITY_DN982_c0_g1/TRINITY_DN4978_c0_g1/TRINITY_DN1071_c0_g1/TRINITY_DN1436_c1_g1/TRINITY_DN16058_c0_g1/TRINITY_DN579_c0_g1	19

GO:0008194	UDP-glycosyltransferase activity	14/2626	44/33142	4.37 × 10^{-6}	9.43 × 10^{-5}	7.06 × 10^{-5}	TRINITY_DN32009_c0_g1/TRINITY_DN16979_c0_g1/TRINITY_DN11954_c0_g1/TRINITY_DN23485_c0_g1/TRINITY_DN15487_c0_g1/TRINITY_DN5087_c0_g1/TRINITY_DN10011_c0_g1/TRINITY_DN4160_c0_g1/TRINITY_DN10583_c0_g1/TRINITY_DN974_c0_g1/TRINITY_DN15324_c1_g1/TRINITY_DN13235_c0_g1/TRINITY_DN13226_c0_g1/TRINITY_DN1200_c2_g1	14
GO:0005201	extracellular matrix structural constituent	16/2626	56/33142	4.50 × 10^{-6}	9.62 × 10^{-5}	7.20 × 10^{-5}	TRINITY_DN12244_c0_g1/TRINITY_DN5310_c0_g2/TRINITY_DN2398_c2_g1/TRINITY_DN1949_c0_g1/TRINITY_DN6305_c1_g1/TRINITY_DN2738_c0_g1/TRINITY_DN1120_c0_g2/TRINITY_DN12194_c0_g1/TRINITY_DN8217_c0_g1/TRINITY_DN740_c0_g1/TRINITY_DN2598_c0_g2/TRINITY_DN18049_c0_g1/TRINITY_DN2868_c0_g1/TRINITY_DN8663_c0_g1/TRINITY_DN2281_c0_g1/TRINITY_DN5908_c0_g1	16
GO:0045752	positive regulation of Toll signaling pathway	11/2626	28/33142	4.57 × 10^{-6}	9.69 × 10^{-5}	7.26 × 10^{-5}	TRINITY_DN6113_c0_g1/TRINITY_DN15335_c0_g1/TRINITY_DN9354_c0_g1/TRINITY_DN10159_c1_g1/TRINITY_DN6957_c0_g1/TRINITY_DN1442	11

							9_c0_g1/TRINITY_DN6641_c0_g2/TRINITY_DN9798_c0_g1/TRINITY_DN6641_c0_g1/TRINITY_DN5802_c0_g1/TRINITY_DN61_c0_g1	
GO:0050892	intestinal absorption	7/2626	11/33142	4.82 × 10^{-6}	0.000101	7.55 × 10^{-5}	TRINITY_DN5364_c0_g1/TRINITY_DN4514_c0_g1/TRINITY_DN5086_c0_g1/TRINITY_DN14877_c1_g1/TRINITY_DN16407_c1_g1/TRINITY_DN11886_c0_g1/TRINITY_DN333_c0_g1	7
GO:0051615	histamine uptake	7/2626	11/33142	4.82 × 10^{-6}	0.000101	7.55 × 10^{-5}	TRINITY_DN2006_c0_g1/TRINITY_DN1478_c1_g3/TRINITY_DN14468_c0_g1/TRINITY_DN10798_c0_g1/TRINITY_DN11056_c1_g1/TRINITY_DN11061_c0_g2/TRINITY_DN6409_c3_g1	7
GO:0042470	melanosome	19/2626	76/33142	5.29 × 10^{-6}	0.000109	8.13 × 10^{-5}	TRINITY_DN47_c0_g1/TRINITY_DN7743_c0_g1/TRINITY_DN9564_c0_g1/TRINITY_DN200_c0_g2/TRINITY_DN8257_c0_g1/TRINITY_DN3507_c0_g1/TRINITY_DN17565_c0_g1/TRINITY_DN19001_c0_g1/TRINITY_DN23921_c0_g1/TRINITY_DN482_c1_g1/TRINITY_DN29856_c0_g1/TRINITY_DN7019_c0_g1/TRINITY_DN1549_c0_g1/TRINITY_DN55372_c0_g1/TRINITY_DN40189_c0_g1/TRI	19

insects

MDPI

							NITY_DN484_c0_g1/TRINITY_DN244_c3_g1/TRINITY_DN111160_c0_g1/TRINITY_DN61025_c0_g1	
GO:0007416	synapse assembly	13/2626	39/33142	5.36×10^{-6}	0.000109	8.13×10^{-5}	TRINITY_DN22536_c0_g1/TRINITY_DN9219_c0_g1/TRINITY_DN2738_c0_g1/TRINITY_DN9375_c0_g1/TRINITY_DN2557_c0_g2/TRINITY_DN8832_c1_g1/TRINITY_DN740_c0_g1//TRINITY_DN2598_c0_g2/TRINITY_DN8217_c1_g1/TRINITY_DN19224_c0_g1/TRINITY_DN2292_c2_g1/TRINITY_DN7687_c0_g1/TRINITY_DN428_c0_g1	13
GO:0007288	sperm axoneme assembly	9/2626	19/33142	5.39×10^{-6}	0.000109	8.13×10^{-5}	TRINITY_DN2752_c0_g1/TRINITY_DN1979_c0_g2/TRINITY_DN3077_c2_g1/TRINITY_DN36928_c0_g1/TRINITY_DN16168_c0_g1/TRINITY_DN6380_c0_g1/TRINITY_DN306_c1_g2/TRINITY_DN10366_c0_g1/TRINITY_DN15544_c0_g1	9
GO:0042462	eye photoreceptor cell development	9/2626	19/33142	5.39×10^{-6}	0.000109	8.13×10^{-5}	TRINITY_DN1893_c0_g1/TRINITY_DN3778_c2_g1/TRINITY_DN6327_c1_g1/TRINITY_DN51872_c0_g1/TRINITY_DN12540_c0_g1/TRINITY_DN96301_c0_g1/TRINITY_DN9845_c0_g2/TRINITY_DN20346_c0_g1/TRINITY_DN49166_c0_g1	9

GO:0097730	non-motile cilium	9/2626	19/33142	5.39 × 10⁻⁶ → 5.39×10^{-6}	0.000109	8.13×10^{-5}	TRINITY_DN2398_c2_g1/TRINITY_DN11509_c0_g1/TRINITY_DN1949_c0_g1/TRINITY_DN22381_c0_g1/TRINITY_DN306_c1_g2/TRINITY_DN31435_c0_g1/TRINITY_DN9168_c0_g2/TRINITY_DN3299_c0_g1/TRINITY_DN4784_c1_g1	9
GO:0008017	microtubule binding	32/2626	173/33142	5.54×10^{-6}	0.000111	8.31×10^{-5}	TRINITY_DN3245_c0_g1/TRINITY_DN19161_c0_g1/TRINITY_DN10881_c0_g1/TRINITY_DN7740_c0_g1/TRINITY_DN4223_c1_g1/TRINITY_DN12450_c0_g2/TRINITY_DN16186_c0_g1/TRINITY_DN16674_c1_g3/TRINITY_DN16674_c1_g2/TRINITY_DN1265_c0_g1/TRINITY_DN5811_c0_g1/TRINITY_DN6222_c0_g1/TRINITY_DN3762_c0_g1/TRINITY_DN2531_c0_g1/TRINITY_DN233_c0_g1/TRINITY_DN13406_c0_g1/TRINITY_DN3597_c0_g2/TRINITY_DN10076_c0_g1/TRINITY_DN4474_c0_g1/TRINITY_DN377_c0_g1/TRINITY_DN8504_c0_g1/TRINITY_DN11664_c0_g1/TRINITY_DN10569_c0_g2/TRINITY_DN18791_c0_g1/TRINITY_DN12133_c0_g1/TRINITY_DN2869_c2_g1/TRINITY_DN26007_c0_g2/TRINITY_DN	32

							3890_c0_g1/TRINITY_DN59259_c0_g1/TRINITY_DN3810_c0_g1/TRINITY_DN5922_c0_g1/TRINITY_DN13780_c0_g1	
GO:0016042	lipid catabolic process	32/2626	174/33142	6.28×10^{-6}	0.000125	9.35×10^{-5}	TRINITY_DN5872_c0_g1/TRINITY_DN1568_c0_g2/TRINITY_DN3772_c0_g1/TRINITY_DN6455_c0_g1/TRINITY_DN1487_c0_g2/TRINITY_DN6700_c0_g1/TRINITY_DN9425_c0_g1/TRINITY_DN77269_c0_g1/TRINITY_DN5164_c0_g3/TRINITY_DN4813_c0_g1/TRINITY_DN21814_c0_g2/TRINITY_DN5164_c0_g2/TRINITY_DN9375_c0_g1/TRINITY_DN23263_c0_g1/TRINITY_DN4485_c0_g1/TRINITY_DN4211_c1_g3/TRINITY_DN39313_c0_g1/TRINITY_DN6482_c0_g1/TRINITY_DN6654_c0_g1/TRINITY_DN18499_c0_g1/TRINITY_DN17391_c0_g1/TRINITY_DN5833_c0_g1/TRINITY_DN20484_c0_g3/TRINITY_DN6745_c0_g1/TRINITY_DN21191_c0_g1/TRINITY_DN4537_c0_g1/TRINITY_DN26376_c0_g1/TRINITY_DN2780_c2_g1/TRINITY_DN3989_c1_g1/TRINITY_DN29189_c0_g1/TRINITY_DN2413_c0_g1/TRINITY_DN10629_c0_g1	32

GO:0006814	sodium ion transport	27/2626	135/33142	6.56 × 10^{-6}	0.00013	9.70 × 10^{-5}	TRINITY_DN3268_c0_g1/TRINITY_DN16444_c1_g1/TRINITY_DN149_c1_g1/TRINITY_DN17739_c0_g2/TRINITY_DN10841_c0_g1/TRINITY_DN27259_c0_g1/TRINITY_DN7737_c0_g1/TRINITY_DN570_c0_g1/TRINITY_DN14068_c0_g2/TRINITY_DN32284_c0_g2/TRINITY_DN6304_c1_g1/TRINITY_DN14068_c0_g1/TRINITY_DN9994_c0_g1/TRINITY_DN8765_c1_g1/TRINITY_DN17412_c0_g1/TRINITY_DN10545_c1_g1/TRINITY_DN17398_c0_g2/TRINITY_DN8644_c0_g1/TRINITY_DN12721_c0_g1/TRINITY_DN14068_c0_g3/TRINITY_DN216_c0_g3/TRINITY_DN4700_c0_g1/TRINITY_DN2003_c0_g1/TRINITY_DN19234_c0_g1/TRINITY_DN20249_c0_g1/TRINITY_DN9004_c0_g1/TRINITY_DN46_c0_g1	27
GO:0008233	peptidase activity	30/2626	159/33142	7.12 × 10^{-6}	0.00014	0.000105	TRINITY_DN3991_c0_g1/TRINITY_DN21789_c0_g1/TRINITY_DN7371_c0_g1/TRINITY_DN3388_c0_g1/TRINITY_DN1830_c0_g2/TRINITY_DN459_c1_g1/TRINITY_DN2476_c2_g1/TRINITY_DN1557_c0_g1/TRINITY_DN13505_c0_g1/TRINI	30

insects

MDPI

							TY_DN2648_c0_g1/TRINITY_DN17581_c0_g1/TRINITY_DN9354_c0_g1/TRINITY_DN40529_c0_g1/TRINITY_DN3503_c0_g1/TRINITY_DN12260_c0_g1/TRINITY_DN19398_c0_g2/TRINITY_DN14570_c0_g1/TRINITY_DN1252_c0_g1/TRINITY_DN4173_c0_g1/TRINITY_DN18049_c0_g1/TRINITY_DN7714_c0_g1/TRINITY_DN60975_c0_g1/TRINITY_DN2682_c0_g1/TRINITY_DN30055_c0_g1/TRINITY_DN46894_c1_g1/TRINITY_DN12254_c0_g3/TRINITY_DN55372_c0_g1/TRINITY_DN1589_c5_g1/TRINITY_DN1436_c1_g1/TRINITY_DN7595_c1_g1	
GO:0003774	motor activity	20/2626	85/33142	8.16×10^{-6}	0.000159	0.000119	TRINITY_DN5849_c0_g2/TRINITY_DN47_c0_g1/TRINITY_DN13313_c0_g1/TRINITY_DN552_c0_g1/TRINITY_DN12886_c0_g1/TRINITY_DN7759_c0_g1/TRINITY_DN4208_c0_g1/TRINITY_DN5459_c0_g1/TRINITY_DN4441_c3_g1/TRINITY_DN15113_c0_g1/TRINITY_DN18791_c0_g1/TRINITY_DN2372_c0_g1/TRINITY_DN5968_c0_g1/TRINITY_DN436_c2_g1/TRINITY_DN14798_c0_g1/TRI	20

							NITY_DN342_c0_g1/TRINITY_DN2347_c1_g1/TRINITY_DN12005_c0_g1/TRINITY_DN39491_c0_g1/TRINITY_DN15080_c0_g1	
GO:0030879	mammary gland development	9/2626	20/33142	9.10×10^{-6}	0.000176	0.000132	TRINITY_DN19001_c0_g1/TRINITY_DN23921_c0_g1/TRINITY_DN482_c1_g1/TRINITY_DN29856_c0_g1/TRINITY_DN1549_c0_g1/TRINITY_DN40189_c0_g1/TRINITY_DN484_c0_g1/TRINITY_DN244_c3_g1/TRINITY_DN61025_c0_g1	9
GO:0004364	glutathione transferase activity	20/2626	86/33142	9.84×10^{-6}	0.000189	0.000141	TRINITY_DN6898_c0_g1/TRINITY_DN7384_c0_g1/TRINITY_DN2458_c0_g2/TRINITY_DN42262_c0_g1/TRINITY_DN6827_c0_g1/TRINITY_DN2458_c0_g1/TRINITY_DN3736_c0_g1/TRINITY_DN12544_c0_g1/TRINITY_DN7082_c0_g1/TRINITY_DN5646_c0_g1/TRINITY_DN10967_c0_g2/TRINITY_DN8346_c1_g1/TRINITY_DN13353_c0_g1/TRINITY_DN1578_c0_g1/TRINITY_DN1118_c0_g1/TRINITY_DN10219_c0_g2/TRINITY_DN14778_c0_g1/TRINITY_DN3942_c0_g1/TRINITY_DN43074_c0_g1/TRINITY_DN8671_c0_g1	20

insects MDPI

GO:0001654	eye development	11/2626	30/33142	1.00 × 10^{-5}	0.000189	0.000141	TRINITY_DN2476_c2_g1/TRINITY_DN2489_c0_g1/TRINITY_DN7582_c0_g1/TRINITY_DN6485_c0_g1/TRINITY_DN480_c0_g1/TRINITY_DN306_c1_g2/TRINITY_DN26279_c0_g1/TRINITY_DN11825_c0_g1/TRINITY_DN3241_c0_g1/TRINITY_DN8116_c0_g1/TRINITY_DN4381_c0_g1	11
GO:0042572	retinol metabolic process	11/2626	30/33142	1.00 × 10^{-5}	0.000189	0.000141	TRINITY_DN146_c0_g1/TRINITY_DN19075_c0_g1/TRINITY_DN21901_c0_g1/TRINITY_DN10027_c0_g1/TRINITY_DN25301_c0_g1/TRINITY_DN31870_c0_g1/TRINITY_DN2009_c0_g1/TRINITY_DN9845_c0_g2/TRINITY_DN20346_c0_g1/TRINITY_DN77404_c0_g1/TRINITY_DN56436_c0_g1	11
GO:0030672	synaptic vesicle membrane	15/2626	53/33142	1.01 × 10^{-5}	0.000189	0.000141	TRINITY_DN9170_c1_g1/TRINITY_DN4032_c0_g1/TRINITY_DN28373_c0_g1/TRINITY_DN5859_c0_g1/TRINITY_DN27259_c0_g1/TRINITY_DN31967_c0_g1/TRINITY_DN7737_c0_g1/TRINITY_DN4477_c0_g1/TRINITY_DN11938_c0_g1/TRINITY_DN25715_c0_g1/TRINITY_DN2165_c3_g1/TRINITY_DN1800_c0_g1/TRINITY_DN33662_c0_g1/TRINITY_DN1106	15

							1_c0_g2/TRINITY_DN4674_c0_g1	
GO:0045503	dynein light chain binding	15/2626	53/33142	1.01 × 10^{-5}	0.000189	0.000141	TRINITY_DN9451_c0_g1/TRINITY_DN1979_c0_g2/TRINITY_DN2557_c0_g2/TRINITY_DN1979_c0_g1/TRINITY_DN4543_c0_g2/TRINITY_DN3083_c0_g1/TRINITY_DN1979_c1_g1/TRINITY_DN18134_c0_g1/TRINITY_DN10356_c0_g1/TRINITY_DN5968_c0_g1/TRINITY_DN14798_c0_g1/TRINITY_DN31689_c0_g1/TRINITY_DN6282_c0_g1/TRINITY_DN9647_c0_g1/TRINITY_DN29956_c0_g1	15
GO:0031638	zymogen activation	10/2626	25/33142	1.03 × 10^{-5}	0.000192	0.000144	TRINITY_DN15335_c0_g1/TRINITY_DN3007_c0_g1/TRINITY_DN2779_c1_g2/TRINITY_DN15112_c0_g1/TRINITY_DN14429_c0_g1/TRINITY_DN6641_c0_g2/TRINITY_DN39494_c0_g1/TRINITY_DN6641_c0_g1/TRINITY_DN2779_c1_g3/TRINITY_DN5802_c0_g1	10
GO:0004503	monophenol monooxygenase activity	8/2626	16/33142	1.11 × 10^{-5}	0.000202	0.000151	TRINITY_DN14689_c0_g1/TRINITY_DN2778_c1_g1/TRINITY_DN1229_c0_g2/TRINITY_DN55476_c0_g1/TRINITY_DN18821_c0_g1/TRINITY_DN70025_c0_g1/TRINITY_DN1229_c0_g4/TRINITY_DN1229_c0_g3	8

GO:0032420	stereocilium	8/2626	16/33142	1.11×10^{-5}	0.000202	0.000151	TRINITY_DN9358_c0_g1/TRINITY_DN17778_c0_g1/TRINITY_DN6174_c0_g1/TRINITY_DN47_c0_g1/TRINITY_DN20653_c0_g1/TRINITY_DN7759_c0_g1/TRINITY_DN2746_c0_g1/TRINITY_DN6945_c0_g2	8
GO:0034375	high-density lipoprotein particle remodeling	8/2626	16/33142	1.11×10^{-5}	0.000202	0.000151	TRINITY_DN8493_c0_g1/TRINITY_DN1568_c0_g1/TRINITY_DN5364_c0_g1/TRINITY_DN5086_c0_g1/TRINITY_DN10757_c0_g1/TRINITY_DN26376_c0_g1/TRINITY_DN333_c0_g1/TRINITY_DN38748_c0_g1	8
GO:0007498	mesoderm development	13/2626	42/33142	1.34×10^{-5}	0.000243	0.000182	TRINITY_DN14383_c0_g1/TRINITY_DN3284_c0_g1/TRINITY_DN13981_c0_g1/TRINITY_DN85_c0_g1/TRINITY_DN5519_c0_g1/TRINITY_DN35269_c0_g1/TRINITY_DN2805_c7_g1/TRINITY_DN9843_c0_g1/TRINITY_DN144_c0_g1/TRINITY_DN39722_c0_g1/TRINITY_DN982_c0_g1/TRINITY_DN8181_c0_g1/TRINITY_DN342_c0_g1	13
GO:0043195	terminal bouton	14/2626	48/33142	1.36×10^{-5}	0.000244	0.000183	TRINITY_DN21399_c0_g2/TRINITY_DN9170_c1_g1/TRINITY_DN8355_c0_g1/TRINITY_DN29720_c0_g1/TRINITY_DN2424_c0_g1/TRINITY_DN11938_c0_g1/TRINITY_DN1765_c2_	14

insects

							g1/TRINITY_DN3816_c0_g1/TRINITY_DN9041_c0_g1/TRINITY_DN4029_c0_g1/TRINITY_DN10575_c2_g1/TRINITY_DN15420_c0_g1/TRINITY_DN7200_c0_g2/TRINITY_DN9041_c0_g2	
GO:0001750	photoreceptor outer segment	10/2626	26/33142	1.56×10^{-5}	0.000275	0.000206	TRINITY_DN146_c0_g1/TRINITY_DN21901_c0_g1/TRINITY_DN10027_c0_g1/TRINITY_DN7048_c0_g1/TRINITY_DN47_c0_g1/TRINITY_DN2924_c1_g1/TRINITY_DN25301_c0_g1/TRINITY_DN5459_c0_g1/TRINITY_DN9845_c0_g2/TRINITY_DN6945_c0_g2	10
GO:0015631	tubulin binding	10/2626	26/33142	1.56×10^{-5}	0.000275	0.000206	TRINITY_DN3245_c0_g1/TRINITY_DN13157_c0_g1/TRINITY_DN5956_c5_g1/TRINITY_DN2051_c0_g1/TRINITY_DN9930_c0_g2/TRINITY_DN2415_c2_g1/TRINITY_DN5912_c0_g1/TRINITY_DN3890_c0_g1/TRINITY_DN3810_c0_g1/TRINITY_DN579_c0_g1	10
GO:0043113	receptor clustering	10/2626	26/33142	1.56×10^{-5}	0.000275	0.000206	TRINITY_DN21399_c0_g2/TRINITY_DN2738_c0_g1/TRINITY_DN740_c0_g1/TRINITY_DN2598_c0_g2/TRINITY_DN1765_c2_g1/TRINITY_DN3816_c0_g1/TRINITY_DN9041_c0_g1/TRINITY_DN7200_c0_g2/TRI	10

							NITY_DN9041_c0_g2/TRINITY_DN7084_c0_g1	
GO:0043197	dendritic spine	15/2626	55/33142	1.65 × 10^{-5}	0.000288	0.000216	TRINITY_DN4329_c1_g1/TRINITY_DN1775_c0_g3/TRINITY_DN1765_c2_g1/TRINITY_DN10569_c0_g2/TRINITY_DN9935_c1_g1/TRINITY_DN10575_c2_g1/TRINITY_DN2869_c2_g1/TRINITY_DN15420_c0_g1/TRINITY_DN7687_c0_g1/TRINITY_DN12650_c0_g1/TRINITY_DN428_c0_g1/TRINITY_DN1908_c0_g1/TRINITY_DN6505_c0_g1/TRINITY_DN49433_c0_g1/TRINITY_DN18471_c0_g2	15
GO:0051959	dynein light intermediate chain binding	15/2626	55/33142	1.65 × 10^{-5}	0.000288	0.000216	TRINITY_DN9451_c0_g1/TRINITY_DN1979_c0_g2/TRINITY_DN1979_c0_g1/TRINITY_DN4543_c0_g2/TRINITY_DN3083_c0_g1/TRINITY_DN1979_c1_g1/TRINITY_DN4208_c0_g1/TRINITY_DN18134_c0_g1/TRINITY_DN10356_c0_g1/TRINITY_DN436_c2_g1/TRINITY_DN31689_c0_g1/TRINITY_DN6282_c0_g1/TRINITY_DN9647_c0_g1/TRINITY_DN29956_c0_g1/TRINITY_DN39491_c0_g1	15
GO:0045944	positive regulation of transcription by RNA polymerase II	61/2626	444/33142	1.86 × 10^{-5}	0.000322	0.000241	TRINITY_DN2578_c0_g1/TRINITY_DN219_c3_g1/TRINITY_DN11739_c0_g1/TRINITY_D	61

N22536_c0_g1/TRINITY_DN1
0890_c0_g1/TRINITY_DN439_
c0_g1/TRINITY_DN3120_c0_g
1/TRINITY_DN2738_c0_g1/TR
INITY_DN5878_c0_g1/TRINIT
Y_DN9571_c0_g2/TRINITY_D
N2476_c1_g1/TRINITY_DN27
78_c0_g3/TRINITY_DN2843_c
0_g2/TRINITY_DN21131_c0_g
2/TRINITY_DN8903_c0_g1/TR
INITY_DN19269_c0_g1/TRINI
TY_DN22837_c0_g1/TRINITY
_DN2898_c0_g1/TRINITY_DN
11693_c0_g2/TRINITY_DN143
83_c0_g1/TRINITY_DN85_c0_
g1/TRINITY_DN170_c1_g1/TR
INITY_DN3713_c1_g1/TRINIT
Y_DN740_c0_g1/TRINITY_D
N1541_c0_g1/TRINITY_DN45
371_c0_g1/TRINITY_DN2598_
c0_g2/TRINITY_DN5296_c0_g
1/TRINITY_DN18436_c0_g1/T
RINITY_DN27747_c0_g1/TRI
NITY_DN9245_c0_g1/TRINIT
Y_DN3722_c0_g1/TRINITY_D
N12413_c0_g1/TRINITY_DN1
111_c0_g1/TRINITY_DN23750
_c0_g1/TRINITY_DN2292_c2_
g1/TRINITY_DN56782_c0_g1/
TRINITY_DN1699_c1_g1/TRI
NITY_DN29974_c0_g1/TRINI
TY_DN40503_c0_g1/TRINITY

						_DN34504_c0_g1/TRINITY_DN10400_c0_g1/TRINITY_DN3636_c0_g3/TRINITY_DN1174_c0_g1/TRINITY_DN3636_c0_g4/TRINITY_DN338_c0_g1/TRINITY_DN4326_c0_g1/TRINITY_DN12512_c0_g1/TRINITY_DN15930_c0_g1/TRINITY_DN3241_c0_g1/TRINITY_DN5118_c0_g2/TRINITY_DN4137_c2_g1/TRINITY_DN16221_c0_g1/TRINITY_DN9800_c1_g1/TRINITY_DN9485_c0_g1/TRINITY_DN8449_c0_g1/TRINITY_DN4784_c1_g1/TRINITY_DN6589_c0_g1/TRINITY_DN7059_c0_g1/TRINITY_DN674_c2_g1/TRINITY_DN8692_c0_g1	
GO:0043691	reverse cholesterol transport	8/2626	17/33142	1.95×10^{-5}	0.000333	0.000249	TRINITY_DN8493_c0_g1/TRINITY_DN5364_c0_g1/TRINITY_DN7048_c0_g1/TRINITY_DN5086_c0_g1/TRINITY_DN10757_c0_g1/TRINITY_DN26376_c0_g1/TRINITY_DN333_c0_g1/TRINITY_DN38748_c0_g1	8
GO:0046328	regulation of JNK cascade	8/2626	17/33142	1.95×10^{-5}	0.000333	0.000249	TRINITY_DN21399_c0_g2/TRINITY_DN1765_c2_g1/TRINITY_DN3816_c0_g1/TRINITY_DN12463_c0_g1/TRINITY_DN9041_c0_g1/TRINITY_DN3333_c0_g1/TRINITY_DN7200_c0_g2/TRINITY_DN9041_c0_g2	8

GO:0018095	protein polyglutamylation	7/2626	13/33142	2.18 × 10^{-5}	0.00037	0.000277	TRINITY_DN7196_c0_g1/TRINITY_DN2415_c2_g1/TRINITY_DN16168_c0_g1/TRINITY_DN489_c2_g1/TRINITY_DN9716_c0_g1/TRINITY_DN7469_c2_g1/TRINITY_DN15544_c0_g1	7
GO:0007368	determination of left/right symmetry	14/2626	50/33142	2.27 × 10^{-5}	0.000384	0.000287	TRINITY_DN6327_c1_g1/TRINITY_DN15240_c0_g1/TRINITY_DN7740_c0_g1/TRINITY_DN9451_c0_g1/TRINITY_DN19269_c0_g1/TRINITY_DN85_c0_g1/TRINITY_DN18134_c0_g1/TRINITY_DN9170_c1_g2/TRINITY_DN306_c1_g2/TRINITY_DN5968_c0_g1/TRINITY_DN3299_c0_g1/TRINITY_DN342_c0_g1/TRINITY_DN59259_c0_g1/TRINITY_DN6541_c1_g2	14
GO:0051290	protein heterotetramerization	12/2626	38/33142	2.30 × 10^{-5}	0.000384	0.000288	TRINITY_DN6597_c0_g1/TRINITY_DN2738_c0_g1/TRINITY_DN8167_c0_g1/TRINITY_DN8805_c1_g1/TRINITY_DN740_c0_g1/TRINITY_DN2598_c0_g2/TRINITY_DN2654_c0_g1/TRINITY_DN17694_c0_g1/TRINITY_DN14132_c0_g1/TRINITY_DN7687_c0_g1/TRINITY_DN4522_c0_g1/TRINITY_DN4784_c1_g1	12
GO:0005044	scavenger receptor activity	10/2626	27/33142	2.30 × 10^{-5}	0.000384	0.000288	TRINITY_DN6550_c0_g1/TRINITY_DN10126_c0_g1/TRINI	10

							TY_DN3487_c0_g1/TRINITY_DN5086_c0_g1/TRINITY_DN309_c0_g1/TRINITY_DN8587_c0_g1/TRINITY_DN28478_c0_g1/TRINITY_DN6641_c0_g2/TRINITY_DN6030_c0_g1/TRINITY_DN3178_c0_g1	
GO:1904315	transmitter-gated ion channel activity involved in regulation of postsynaptic membrane potential	9/2626	22/33142	2.33×10^{-5}	0.000387	0.00029	TRINITY_DN21399_c0_g2/TRINITY_DN16006_c0_g1/TRINITY_DN8355_c0_g1/TRINITY_DN1765_c2_g1/TRINITY_DN3816_c0_g1/TRINITY_DN9041_c0_g1/TRINITY_DN7200_c0_g2/TRINITY_DN9041_c0_g2/TRINITY_DN21399_c0_g1	9
GO:0001889	liver development	13/2626	44/33142	2.35×10^{-5}	0.000389	0.000291	TRINITY_DN32009_c0_g1/TRINITY_DN7426_c0_g1/TRINITY_DN11954_c0_g1/TRINITY_DN1568_c0_g1/TRINITY_DN306_c1_g2/TRINITY_DN13138_c0_g1/TRINITY_DN974_c0_g1/TRINITY_DN16751_c0_g1/TRINITY_DN4859_c0_g1/TRINITY_DN15324_c1_g1/TRINITY_DN40503_c0_g1/TRINITY_DN15536_c0_g1/TRINITY_DN4137_c2_g1	13
GO:0043525	positive regulation of neuron apoptotic process	11/2626	33/33142	2.84×10^{-5}	0.000466	0.000349	TRINITY_DN21399_c0_g2/TRINITY_DN1557_c0_g1/TRINITY_DN2738_c0_g1/TRINITY_DN740_c0_g1/TRINITY_DN2598_c0_g2/TRINITY_DN1797_c0	11

							_g1/TRINITY_DN1765_c2_g1/TRINITY_DN3816_c0_g1/TRINITY_DN9041_c0_g1/TRINITY_DN7200_c0_g2/TRINITY_DN9041_c0_g2	
GO:0005125	cytokine activity	14/2626	51/33142	2.90×10^{-5}	0.000468	0.00035	TRINITY_DN6342_c6_g1/TRINITY_DN14550_c0_g1/TRINITY_DN24518_c0_g1/TRINITY_DN9767_c0_g3/TRINITY_DN8330_c0_g1/TRINITY_DN15785_c0_g1/TRINITY_DN51904_c0_g1/TRINITY_DN37845_c0_g1/TRINITY_DN8330_c0_g2/TRINITY_DN7030_c0_g1/TRINITY_DN8681_c0_g1/TRINITY_DN13165_c0_g1/TRINITY_DN11595_c0_g1/TRINITY_DN11595_c0_g2	14
GO:0050839	cell adhesion molecule binding	14/2626	51/33142	2.90×10^{-5}	0.000468	0.00035	TRINITY_DN5310_c0_g2/TRINITY_DN2244_c1_g1/TRINITY_DN1266_c0_g1/TRINITY_DN1120_c0_g2/TRINITY_DN9375_c0_g1/TRINITY_DN15308_c0_g2/TRINITY_DN17565_c0_g1/TRINITY_DN43399_c0_g1/TRINITY_DN27045_c0_g1/TRINITY_DN12127_c0_g1/TRINITY_DN446_c1_g1/TRINITY_DN7687_c0_g1/TRINITY_DN7523_c0_g1/TRINITY_DN12958_c0_g1	14

GO:0070402	NADPH binding	14/2626	51/33142	2.90 × 10^{-5}	0.000468	0.00035	TRINITY_DN10137_c0_g1/TRINITY_DN58623_c2_g1/TRINITY_DN19001_c0_g1/TRINITY_DN1610_c0_g1/TRINITY_DN23921_c0_g1/TRINITY_DN482_c1_g1/TRINITY_DN29856_c0_g1/TRINITY_DN1549_c0_g1/TRINITY_DN40189_c0_g1/TRINITY_DN484_c0_g1/TRINITY_DN6300_c0_g1/TRINITY_DN244_c3_g1/TRINITY_DN8169_c0_g2/TRINITY_DN61025_c0_g1	14
GO:0004017	adenylate kinase activity	8/2626	18/33142	3.26 × 10^{-5}	0.000523	0.000392	TRINITY_DN7530_c0_g1/TRINITY_DN15793_c0_g2/TRINITY_DN34424_c0_g1/TRINITY_DN10718_c0_g1/TRINITY_DN2881_c0_g1/TRINITY_DN5649_c0_g1/TRINITY_DN38340_c0_g1/TRINITY_DN2881_c0_g2	8
GO:0008144	drug binding	15/2626	58/33142	3.30 × 10^{-5}	0.000526	0.000393	TRINITY_DN3991_c0_g1/TRINITY_DN7437_c0_g1/TRINITY_DN6767_c0_g1/TRINITY_DN9333_c0_g1/TRINITY_DN19001_c0_g1/TRINITY_DN2682_c0_g1/TRINITY_DN23921_c0_g1/TRINITY_DN482_c1_g1/TRINITY_DN29856_c0_g1/TRINITY_DN1549_c0_g1/TRINITY_DN40189_c0_g1/TRINITY_DN484_c0_g1/TRINITY_DN10344	15

							_c0_g1/TRINITY_DN244_c3_g 1/TRINITY_DN61025_c0_g1	
GO:0047117	enoyl-[acyl-carrier-protein] reductase (NADPH，A-specific) activity	10/2626	28/33142	3.32 × 10^{-5}	0.000527	0.000395	TRINITY_DN606_c3_g1/TRINITY_DN19001_c0_g1/TRINITY_DN23921_c0_g1/TRINITY_DN482_c1_g1/TRINITY_DN29856_c0_g1/TRINITY_DN1549_c0_g1/TRINITY_DN40189_c0_g1/TRINITY_DN484_c0_g1/TRINITY_DN244_c3_g1/TRINITY_DN61025_c0_g1	10
GO:0051607	defense response to virus	22/2626	108/33142	3.37 × 10^{-5}	0.000532	0.000399	TRINITY_DN6342_c6_g1/TRINITY_DN402_c1_g1/TRINITY_DN5956_c5_g1/TRINITY_DN2051_c0_g1/TRINITY_DN14550_c0_g1/TRINITY_DN5216_c1_g1/TRINITY_DN24518_c0_g1/TRINITY_DN8330_c0_g1/TRINITY_DN15785_c0_g1/TRINITY_DN4768_c0_g1/TRINITY_DN51904_c0_g1/TRINITY_DN479_c0_g2/TRINITY_DN8330_c0_g2/TRINITY_DN6677_c0_g1/TRINITY_DN7030_c0_g1/TRINITY_DN5912_c0_g1/TRINITY_DN68176_c0_g1/TRINITY_DN4326_c0_g1/TRINITY_DN3238_c0_g1/TRINITY_DN13165_c0_g1/TRINITY_DN11595_c0_g1/TRINITY_DN11595_c0_g2	22
GO:0007189	adenylate cyclase-activating G protein-coupled receptor signaling pathway	11/2626	34/33142	3.90 × 10^{-5}	0.000593	0.000444	TRINITY_DN17778_c0_g1/TRINITY_DN14379_c0_g1/TRINI	11

insects MDPI

							TY_DN7048_c0_g1/TRINITY_DN20653_c0_g1/TRINITY_DN20653_c0_g4/TRINITY_DN6839_c2_g1/TRINITY_DN58254_c0_g1/TRINITY_DN58043_c0_g1/TRINITY_DN20653_c0_g2/TRINITY_DN2285_c0_g1/TRINITY_DN8692_c0_g1	
GO:0018833	DDT-dehydrochlorinase activity	6/2626	10/33142	3.91×10^{-5}	0.000593	0.000444	TRINITY_DN2458_c0_g2/TRINITY_DN2458_c0_g1/TRINITY_DN12544_c0_g1/TRINITY_DN10967_c0_g2/TRINITY_DN43074_c0_g1/TRINITY_DN8671_c0_g1	6
GO:0048169	regulation of long-term neuronal synaptic plasticity	6/2626	10/33142	3.91×10^{-5}	0.000593	0.000444	TRINITY_DN21399_c0_g2/TRINITY_DN1765_c2_g1/TRINITY_DN3816_c0_g1/TRINITY_DN9041_c0_g1/TRINITY_DN7200_c0_g2/TRINITY_DN9041_c0_g2	6
GO:0050806	positive regulation of synaptic transmission	6/2626	10/33142	3.91×10^{-5}	0.000593	0.000444	TRINITY_DN21399_c0_g2/TRINITY_DN1765_c2_g1/TRINITY_DN3816_c0_g1/TRINITY_DN9041_c0_g1/TRINITY_DN7200_c0_g2/TRINITY_DN9041_c0_g2	6
GO:0060122	inner ear receptor cell stereocilium organization	6/2626	10/33142	3.91×10^{-5}	0.000593	0.000444	TRINITY_DN9358_c0_g1/TRINITY_DN6174_c0_g1/TRINITY_DN47_c0_g1/TRINITY_DN306_c1_g2/TRINITY_DN2746_c0_g1/TRINITY_DN9168_c0_g2	6

GO:0097225	sperm midpiece	6/2626	10/33142	3.91 × 10^{-5}	0.000593	0.000444	TRINITY_DN3991_c0_g1/TRINITY_DN954_c0_g1/TRINITY_DN36153_c0_g3/TRINITY_DN2682_c0_g1/TRINITY_DN22905_c0_g1/TRINITY_DN11886_c0_g1	6
GO:1904322	cellular response to forskolin	6/2626	10/33142	3.91 × 10^{-5}	0.000593	0.000444	TRINITY_DN17778_c0_g1/TRINITY_DN20653_c0_g1/TRINITY_DN3713_c1_g1/TRINITY_DN20653_c0_g4/TRINITY_DN6839_c2_g1/TRINITY_DN20653_c0_g2	6
GO:0030317	flagellated sperm motility	13/2626	46/33142	3.97 × 10^{-5}	0.000596	0.000446	TRINITY_DN6657_c0_g1/TRINITY_DN19725_c0_g1/TRINITY_DN11686_c1_g1/TRINITY_DN7740_c0_g1/TRINITY_DN9451_c0_g1/TRINITY_DN1979_c0_g2/TRINITY_DN6610_c0_g1/TRINITY_DN6323_c1_g1/TRINITY_DN7737_c0_g1/TRINITY_DN18134_c0_g1/TRINITY_DN2725_c0_g1/TRINITY_DN30782_c0_g2/TRINITY_DN59259_c0_g1	13
GO:0046426	negative regulation of receptor signaling pathway via JAK-STAT	13/2626	46/33142	3.97 × 10^{-5}	0.000596	0.000446	TRINITY_DN6342_c6_g1/TRINITY_DN14550_c0_g1/TRINITY_DN24518_c0_g1/TRINITY_DN2476_c1_g1/TRINITY_DN8330_c0_g1/TRINITY_DN15785_c0_g1/TRINITY_DN51904_c0_g1/TRINITY_DN8330_c0_g2/TRINITY_DN7030_c0_g1/TRI	13

							NITY_DN13165_c0_g1/TRINITY_DN11595_c0_g1/TRINITY_DN11595_c0_g2/TRINITY_DN16327_c0_g2	
GO:0019228	neuronal action potential	7/2626	14/33142	4.05×10^{-5}	0.000596	0.000446	TRINITY_DN21399_c0_g2/TRINITY_DN1765_c2_g1/TRINITY_DN3816_c0_g1/TRINITY_DN9041_c0_g1/TRINITY_DN17398_c0_g2/TRINITY_DN7200_c0_g2/TRINITY_DN9041_c0_g2	7
GO:0019367	fatty acid elongation， saturated fatty acid	7/2626	14/33142	4.05×10^{-5}	0.000596	0.000446	TRINITY_DN5032_c1_g1/TRINITY_DN1854_c0_g2/TRINITY_DN7107_c0_g1/TRINITY_DN12176_c0_g1/TRINITY_DN34926_c1_g1/TRINITY_DN10258_c0_g1/TRINITY_DN13730_c0_g1	7
GO:0034626	fatty acid elongation， polyunsaturated fatty acid	7/2626	14/33142	4.05×10^{-5}	0.000596	0.000446	TRINITY_DN5032_c1_g1/TRINITY_DN1854_c0_g2/TRINITY_DN7107_c0_g1/TRINITY_DN12176_c0_g1/TRINITY_DN34926_c1_g1/TRINITY_DN10258_c0_g1/TRINITY_DN13730_c0_g1	7
GO:0060080	inhibitory postsynaptic potential	7/2626	14/33142	4.05×10^{-5}	0.000596	0.000446	TRINITY_DN21399_c0_g2/TRINITY_DN1765_c2_g1/TRINITY_DN3816_c0_g1/TRINITY_DN9041_c0_g1/TRINITY_DN15930_c0_g1/TRINITY_DN7200_c0_g2/TRINITY_DN9041_c0_g2	7

GO:0015630	microtubule cytoskeleton	16/2626	66/33142	4.32×10^{-5}	0.000632	0.000473	TRINITY_DN16600_c0_g1/TRINITY_DN3245_c0_g1/TRINITY_DN7740_c0_g1/TRINITY_DN4544_c0_g1/TRINITY_DN4759_c0_g1/TRINITY_DN15133_c0_g1/TRINITY_DN3665_c0_g1/TRINITY_DN10569_c0_g2/TRINITY_DN1699_c1_g1/TRINITY_DN12133_c0_g1/TRINITY_DN2869_c2_g1/TRINITY_DN17984_c0_g1/TRINITY_DN22883_c0_g1/TRINITY_DN26007_c0_g2/TRINITY_DN59259_c0_g1/TRINITY_DN5922_c0_g1	16
GO:0004313	[acyl-carrier-protein] S-acetyltransferase activity	10/2626	29/33142	4.71×10^{-5}	0.000682	0.000511	TRINITY_DN606_c3_g1/TRINITY_DN19001_c0_g1/TRINITY_DN23921_c0_g1/TRINITY_DN482_c1_g1/TRINITY_DN29856_c0_g1/TRINITY_DN1549_c0_g1/TRINITY_DN40189_c0_g1/TRINITY_DN484_c0_g1/TRINITY_DN244_c3_g1/TRINITY_DN61025_c0_g1	10
GO:0006084	acetyl-CoA metabolic process	10/2626	29/33142	4.71×10^{-5}	0.000682	0.000511	TRINITY_DN9281_c0_g1/TRINITY_DN19001_c0_g1/TRINITY_DN23921_c0_g1/TRINITY_DN482_c1_g1/TRINITY_DN29856_c0_g1/TRINITY_DN1549_c0_g1/TRINITY_DN40189_c0_g1/TRINITY_DN484_c0_g1/TRINITY_DN244_c3_g1/TRINITY_DN61025_c0_g1	10

GO:0009897	external side of plasma membrane	15/2626	60/33142	5.06 × 10^{-5}	0.00073	0.000546	TRINITY_DN3991_c0_g1/TRINITY_DN7437_c0_g1/TRINITY_DN22536_c0_g1/TRINITY_DN7048_c0_g1/TRINITY_DN7743_c0_g1/TRINITY_DN200_c0_g2/TRINITY_DN479_c0_g2/TRINITY_DN1765_c2_g1/TRINITY_DN2682_c0_g1/TRINITY_DN10757_c0_g1/TRINITY_DN2292_c2_g1/TRINITY_DN5968_c0_g1/TRINITY_DN7687_c0_g1/TRINITY_DN12958_c0_g1/TRINITY_DN38748_c0_g1	15
GO:0098686	hippocampal mossy fiber to CA3 synapse	8/2626	19/33142	5.24 × 10^{-5}	0.000751	0.000562	TRINITY_DN21399_c0_g2/TRINITY_DN11938_c0_g1/TRINITY_DN1765_c2_g1/TRINITY_DN3816_c0_g1/TRINITY_DN9041_c0_g1/TRINITY_DN7200_c0_g2/TRINITY_DN273_c1_g1/TRINITY_DN9041_c0_g2	8
GO:0009950	dorsal/ventral axis specification	11/2626	35/33142	5.28 × 10^{-5}	0.000751	0.000562	TRINITY_DN22536_c0_g1/TRINITY_DN10890_c0_g1/TRINITY_DN7582_c0_g1/TRINITY_DN15335_c0_g1/TRINITY_DN10159_c1_g1/TRINITY_DN6957_c0_g1/TRINITY_DN14429_c0_g1/TRINITY_DN6641_c0_g2/TRINITY_DN6641_c0_g1/TRINITY_DN2292_c2_g1/TRINITY_DN5802_c0_g1	11
GO:0033344	cholesterol efflux	9/2626	24/33142	5.29 × 10^{-5}	0.000751	0.000562	TRINITY_DN8493_c0_g1/TRINITY_DN5364_c0_g1/TRINIT	9

							Y_DN7048_c0_g1/TRINITY_DN5086_c0_g1/TRINITY_DN10757_c0_g1/TRINITY_DN7045_c1_g1/TRINITY_DN333_c0_g1/TRINITY_DN38748_c0_g1/TRINITY_DN8292_c0_g1	
GO:0043065	positive regulation of apoptotic process	20/2626	96/33142	5.40 × 10^{-5}	0.000763	0.000571	TRINITY_DN3991_c0_g1/TRINITY_DN7582_c0_g1/TRINITY_DN1990_c0_g1/TRINITY_DN4544_c0_g1/TRINITY_DN17487_c0_g1/TRINITY_DN6485_c0_g1/TRINITY_DN5951_c0_g4/TRINITY_DN7131_c0_g1/TRINITY_DN1797_c0_g1/TRINITY_DN11938_c0_g1/TRINITY_DN2682_c0_g1/TRINITY_DN3665_c0_g1/TRINITY_DN10145_c1_g1/TRINITY_DN5120_c0_g1/TRINITY_DN4326_c0_g1/TRINITY_DN3238_c0_g1/TRINITY_DN23722_c0_g1/TRINITY_DN6505_c0_g1/TRINITY_DN26727_c0_g1/TRINITY_DN7471_c0_g1	20
GO:0006749	glutathione metabolic process	15/2626	61/33142	6.23 × 10^{-5}	0.000875	0.000655	TRINITY_DN7384_c0_g1/TRINITY_DN2458_c0_g2/TRINITY_DN42262_c0_g1/TRINITY_DN2458_c0_g1/TRINITY_DN3007_c0_g1/TRINITY_DN2779_c1_g2/TRINITY_DN12544_c0_g1/TRINITY_DN5646_c0_g1/TRINITY_DN10967_c0_g2/TRI	15

							NITY_DN2779_c1_g3/TRINITY_DN13353_c0_g1/TRINITY_DN1118_c0_g1/TRINITY_DN3942_c0_g1/TRINITY_DN43074_c0_g1/TRINITY_DN8671_c0_g1	
GO:0004320	oleoyl-[acyl-carrier-protein] hydrolase activity	10/2626	30/33142	6.57×10^{-5}	0.000918	0.000687	TRINITY_DN606_c3_g1/TRINITY_DN19001_c0_g1/TRINITY_DN23921_c0_g1/TRINITY_DN482_c1_g1/TRINITY_DN29856_c0_g1/TRINITY_DN1549_c0_g1/TRINITY_DN40189_c0_g1/TRINITY_DN484_c0_g1/TRINITY_DN244_c3_g1/TRINITY_DN61025_c0_g1	10
GO:0009922	fatty acid elongase activity	7/2626	15/33142	7.08×10^{-5}	0.000979	0.000733	TRINITY_DN5032_c1_g1/TRINITY_DN1854_c0_g2/TRINITY_DN7107_c0_g1/TRINITY_DN12176_c0_g1/TRINITY_DN34926_c1_g1/TRINITY_DN10258_c0_g1/TRINITY_DN13730_c0_g1	7
GO:0097542	ciliary tip	7/2626	15/33142	7.08×10^{-5}	0.000979	0.000733	TRINITY_DN5006_c0_g2/TRINITY_DN19269_c0_g1/TRINITY_DN11340_c0_g1/TRINITY_DN13406_c0_g1/TRINITY_DN5875_c0_g1/TRINITY_DN306_c1_g2/TRINITY_DN9168_c0_g2	7
GO:0102131	3-oxo-glutaryl-[acp] methyl ester reductase activity	15/2626	62/33142	7.62×10^{-5}	0.001038	0.000777	TRINITY_DN606_c3_g1/TRINITY_DN4958_c0_g1/TRINITY_DN6834_c2_g3/TRINITY_DN1	15

							9001_c0_g1/TRINITY_DN9765_c0_g1/TRINITY_DN23785_c0_g1/TRINITY_DN23921_c0_g1/TRINITY_DN482_c1_g1/TRINITY_DN29856_c0_g1/TRINITY_DN1549_c0_g1/TRINITY_DN26032_c0_g1/TRINITY_DN40189_c0_g1/TRINITY_DN484_c0_g1/TRINITY_DN244_c3_g1/TRINITY_DN61025_c0_g1	
GO:0102132	3-oxo-pimeloyl-[acp] methyl ester reductase activity	15/2626	62/33142	7.62×10^{-5}	0.001038	0.000777	TRINITY_DN606_c3_g1/TRINITY_DN4958_c0_g1/TRINITY_DN6834_c2_g3/TRINITY_DN19001_c0_g1/TRINITY_DN9765_c0_g1/TRINITY_DN23785_c0_g1/TRINITY_DN23921_c0_g1/TRINITY_DN482_c1_g1/TRINITY_DN29856_c0_g1/TRINITY_DN1549_c0_g1/TRINITY_DN26032_c0_g1/TRINITY_DN40189_c0_g1/TRINITY_DN484_c0_g1/TRINITY_DN244_c3_g1/TRINITY_DN61025_c0_g1	15
GO:0019216	regulation of lipid metabolic process	9/2626	25/33142	7.69×10^{-5}	0.001038	0.000777	TRINITY_DN72971_c0_g1/TRINITY_DN7048_c0_g1/TRINITY_DN4826_c1_g1/TRINITY_DN3732_c0_g1/TRINITY_DN974_c0_g1/TRINITY_DN16751_c0_g1/TRINITY_DN12338_c0_g1/TRINITY_DN43641_c0_g1/TRINITY_DN3026_c1_g1	9

GO:0032587	ruffle membrane	9/2626	25/33142	7.69×10^{-5}	0.001038	0.000777	TRINITY_DN9277_c0_g1/TRINITY_DN4329_c1_g1/TRINITY_DN7045_c1_g1/TRINITY_DN93_c0_g1/TRINITY_DN5362_c0_g1/TRINITY_DN2869_c2_g1/TRINITY_DN30302_c0_g1/TRINITY_DN13875_c0_g1/TRINITY_DN6505_c0_g1	9
GO:0051402	neuron apoptotic process	9/2626	25/33142	7.69×10^{-5}	0.001038	0.000777	TRINITY_DN21399_c0_g2/TRINITY_DN1557_c0_g1/TRINITY_DN1765_c2_g1/TRINITY_DN3816_c0_g1/TRINITY_DN9041_c0_g1/TRINITY_DN55372_c0_g1/TRINITY_DN7200_c0_g2/TRINITY_DN3238_c0_g1/TRINITY_DN9041_c0_g2	9
GO:0032098	regulation of appetite	6/2626	11/33142	8.02×10^{-5}	0.001072	0.000803	TRINITY_DN2006_c0_g1/TRINITY_DN1478_c1_g3/TRINITY_DN14468_c0_g1/TRINITY_DN10798_c0_g1/TRINITY_DN11056_c1_g1/TRINITY_DN11061_c0_g2	6
GO:0061178	regulation of insulin secretion involved in cellular response to glucose stimulus	6/2626	11/33142	8.02×10^{-5}	0.001072	0.000803	TRINITY_DN17778_c0_g1/TRINITY_DN20653_c0_g1/TRINITY_DN7737_c0_g1/TRINITY_DN20653_c0_g4/TRINITY_DN6839_c2_g1/TRINITY_DN20653_c0_g2	6
GO:0001669	acrosomal vesicle	8/2626	20/33142	8.13×10^{-5}	0.001077	0.000806	TRINITY_DN9943_c0_g1/TRINITY_DN2841_c0_g1/TRINITY_DN3375_c0_g1/TRINITY_DN15863_c0_g1/TRINITY_DN5	8

							629_c0_g1/TRINITY_DN5859_c0_g1/TRINITY_DN19398_c0_g2/TRINITY_DN22607_c0_g1	
GO:0042574	retinal metabolic process	8/2626	20/33142	8.13×10^{-5}	0.001077	0.000806	TRINITY_DN3778_c2_g1/TRINITY_DN19075_c0_g1/TRINITY_DN51872_c0_g1/TRINITY_DN12540_c0_g1/TRINITY_DN96301_c0_g1/TRINITY_DN9845_c0_g2/TRINITY_DN20346_c0_g1/TRINITY_DN49166_c0_g1	8
GO:0008201	heparin binding	13/2626	49/33142	8.19×10^{-5}	0.00108	0.000809	TRINITY_DN6651_c0_g1/TRINITY_DN1568_c0_g2/TRINITY_DN1568_c0_g1/TRINITY_DN6886_c3_g1/TRINITY_DN4813_c0_g1/TRINITY_DN11100_c0_g1/TRINITY_DN7955_c1_g1/TRINITY_DN8681_c0_g1/TRINITY_DN17391_c0_g1/TRINITY_DN26376_c0_g1/TRINITY_DN5908_c0_g1/TRINITY_DN5274_c0_g1/TRINITY_DN2285_c0_g1	13
GO:0030154	cell differentiation	39/2626	260/33142	8.83×10^{-5}	0.001145	0.000858	TRINITY_DN12244_c0_g1/TRINITY_DN3120_c0_g1/TRINITY_DN1695_c0_g1/TRINITY_DN5878_c0_g1/TRINITY_DN2648_c0_g1/TRINITY_DN4544_c0_g1/TRINITY_DN11204_c0_g1/TRINITY_DN17487_c0_g1/TRINITY_DN12504_c0_g1/TRINITY_DN6323_c1_g1/TRINIT	39

							Y_DN36153_c0_g3/TRINITY_DN14383_c0_g1/TRINITY_DN8796_c0_g1/TRINITY_DN7744_c0_g1/TRINITY_DN170_c1_g1/TRINITY_DN2117_c0_g2/TRINITY_DN10783_c0_g1/TRINITY_DN4173_c0_g1/TRINITY_DN3363_c0_g1/TRINITY_DN3352_c0_g2/TRINITY_DN12891_c0_g1/TRINITY_DN4979_c0_g1/TRINITY_DN306_c4_g1/TRINITY_DN1699_c1_g1/TRINITY_DN8043_c0_g1/TRINITY_DN5276_c1_g1/TRINITY_DN144_c0_g1/TRINITY_DN34504_c0_g1/TRINITY_DN2281_c0_g1/TRINITY_DN4000_c0_g1/TRINITY_DN15930_c0_g1/TRINITY_DN23609_c0_g1/TRINITY_DN30782_c0_g2/TRINITY_DN15445_c0_g1/TRINITY_DN4137_c2_g1/TRINITY_DN22707_c1_g1/TRINITY_DN12889_c0_g1/TRINITY_DN6505_c0_g1/TRINITY_DN11152_c0_g1	
GO:0003333	amino acid transmembrane transport	12/2626	43/33142	8.96×10^{-5}	0.001145	0.000858	TRINITY_DN8323_c0_g1/TRINITY_DN3446_c0_g1/TRINITY_DN6519_c0_g1/TRINITY_DN43642_c0_g1/TRINITY_DN4139_c0_g1/TRINITY_DN13394_c0_g1/TRINITY_DN13889_c0_g1/TRINITY_DN8012_c0_g1/	12

							TRINITY_DN6705_c0_g2/TRINITY_DN4307_c0_g1/TRINITY_DN3638_c0_g2/TRINITY_DN12101_c0_g2	
GO:0008344	adult locomotory behavior	12/2626	43/33142	8.96×10^{-5}	0.001145	0.000858	TRINITY_DN7807_c0_g1/TRINITY_DN1487_c0_g2/TRINITY_DN1748_c0_g1/TRINITY_DN388_c0_g1/TRINITY_DN6076_c0_g1/TRINITY_DN9333_c0_g1/TRINITY_DN1267_c0_g2/TRINITY_DN9532_c0_g1/TRINITY_DN5521_c0_g1/TRINITY_DN8599_c1_g1/TRINITY_DN6745_c0_g1/TRINITY_DN26007_c0_g2	12
GO:0007517	muscle organ development	17/2626	77/33142	8.99×10^{-5}	0.001145	0.000858	TRINITY_DN4644_c0_g1/TRINITY_DN85_c0_g1/TRINITY_DN3732_c0_g1/TRINITY_DN5519_c0_g1/TRINITY_DN10569_c0_g2/TRINITY_DN8662_c1_g1/TRINITY_DN9843_c0_g1/TRINITY_DN40503_c0_g1/TRINITY_DN144_c0_g1/TRINITY_DN5362_c0_g1/TRINITY_DN2281_c0_g1/TRINITY_DN3103_c0_g2/TRINITY_DN14168_c0_g2/TRINITY_DN15445_c0_g1/TRINITY_DN4137_c2_g1/TRINITY_DN3103_c2_g1/TRINITY_DN14850_c0_g1	17
GO:0005902	microvillus	10/2626	31/33142	9.01×10^{-5}	0.001145	0.000858	TRINITY_DN9358_c0_g1/TRINITY_DN1568_c0_g1/TRINIT	10

							Y_DN47_c0_g1/TRINITY_DN5495_c1_g2/TRINITY_DN14381_c0_g1/TRINITY_DN6745_c0_g1/TRINITY_DN22905_c0_g1/TRINITY_DN22607_c0_g1/TRINITY_DN12958_c0_g1/TRINITY_DN2347_c1_g1	
GO:0016295	myristoyl-[acyl-carrier-protein] hydrolase activity	10/2626	31/33142	9.01×10^{-5}	0.001145	0.000858	TRINITY_DN606_c3_g1/TRINITY_DN19001_c0_g1/TRINITY_DN23921_c0_g1/TRINITY_DN482_c1_g1/TRINITY_DN29856_c0_g1/TRINITY_DN1549_c0_g1/TRINITY_DN40189_c0_g1/TRINITY_DN484_c0_g1/TRINITY_DN244_c3_g1/TRINITY_DN61025_c0_g1	10
GO:0016296	palmitoyl-[acyl-carrier-protein] hydrolase activity	10/2626	31/33142	9.01×10^{-5}	0.001145	0.000858	TRINITY_DN606_c3_g1/TRINITY_DN19001_c0_g1/TRINITY_DN23921_c0_g1/TRINITY_DN482_c1_g1/TRINITY_DN29856_c0_g1/TRINITY_DN1549_c0_g1/TRINITY_DN40189_c0_g1/TRINITY_DN484_c0_g1/TRINITY_DN244_c3_g1/TRINITY_DN61025_c0_g1	10
GO:0048814	regulation of dendrite morphogenesis	10/2626	31/33142	9.01×10^{-5}	0.001145	0.000858	TRINITY_DN5239_c1_g2/TRINITY_DN6076_c0_g1/TRINITY_DN6677_c0_g1/TRINITY_DN1178_c2_g1/TRINITY_DN10145_c1_g1/TRINITY_DN9935_c1_g1/TRINITY_DN2475_c0_g1/TRINITY_DN9158_c3_g1/TR	10

							INITY_DN2869_c2_g1/TRINITY_DN709_c0_g1	
GO:0004316	3-oxoacyl-[acyl-carrier-protein] reductase (NADPH) activity	15/2626	63/33142	9.27×10^{-5}	0.001169	0.000875	TRINITY_DN606_c3_g1/TRINITY_DN4958_c0_g1/TRINITY_DN6834_c2_g3/TRINITY_DN19001_c0_g1/TRINITY_DN9765_c0_g1/TRINITY_DN23785_c0_g1/TRINITY_DN23921_c0_g1/TRINITY_DN482_c1_g1/TRINITY_DN29856_c0_g1/TRINITY_DN1549_c0_g1/TRINITY_DN26032_c0_g1/TRINITY_DN40189_c0_g1/TRINITY_DN484_c0_g1/TRINITY_DN244_c3_g1/TRINITY_DN61025_c0_g1	15
GO:0007584	response to nutrient	15/2626	63/33142	9.27×10^{-5}	0.001169	0.000875	TRINITY_DN5056_c0_g1/TRINITY_DN7048_c0_g1/TRINITY_DN2629_c0_g1/TRINITY_DN5495_c1_g2/TRINITY_DN14381_c0_g1/TRINITY_DN9333_c0_g1/TRINITY_DN974_c0_g1/TRINITY_DN8278_c2_g1/TRINITY_DN6638_c2_g1/TRINITY_DN4859_c0_g1/TRINITY_DN26376_c0_g1/TRINITY_DN15536_c0_g1/TRINITY_DN6985_c0_g1/TRINITY_DN12958_c0_g1/TRINITY_DN5788_c1_g1	15
GO:0007420	brain development	23/2626	124/33142	0.000105	0.001311	0.000982	TRINITY_DN1487_c0_g2/TRINITY_DN4783_c0_g1/TRINITY_DN7740_c0_g1/TRINITY_DN9219_c0_g1/TRINITY_DN45	23

							44_c0_g1/TRINITY_DN5239_c1_g2/TRINITY_DN7530_c0_g1/TRINITY_DN17663_c0_g1/TRINITY_DN5781_c0_g1/TRINITY_DN480_c0_g1/TRINITY_DN9676_c0_g1/TRINITY_DN1797_c0_g1/TRINITY_DN306_c1_g2/TRINITY_DN2095_c0_g1/TRINITY_DN23750_c0_g1/TRINITY_DN4859_c0_g1/TRINITY_DN637_c0_g1/TRINITY_DN59259_c0_g1/TRINITY_DN7318_c0_g1/TRINITY_DN13354_c0_g1/TRINITY_DN2285_c0_g1/TRINITY_DN8116_c0_g1/TRINITY_DN4381_c0_g1	
GO:0000981	DNA-binding transcription factor activity, RNA polymerase II-specific	37/2626	244/33142	0.000105	0.001313	0.000983	TRINITY_DN1893_c0_g1/TRINITY_DN219_c3_g1/TRINITY_DN10890_c0_g1/TRINITY_DN1695_c0_g1/TRINITY_DN5878_c0_g1/TRINITY_DN8236_c0_g1/TRINITY_DN22837_c0_g1/TRINITY_DN4789_c0_g1/TRINITY_DN14383_c0_g1/TRINITY_DN13176_c0_g1/TRINITY_DN3284_c0_g1/TRINITY_DN85_c0_g1/TRINITY_DN170_c1_g1/TRINITY_DN3713_c1_g1/TRINITY_DN49948_c0_g1/TRINITY_DN1541_c0_g1/TRINITY_DN5296_c0_g1/TRINITY_DN3352_c0_g2/TRINITY_DN12	37

insects MDPI

							891_c0_g1/TRINITY_DN21038_c0_g2/TRINITY_DN2095_c0_g1/TRINITY_DN3722_c0_g1/TRINITY_DN13176_c0_g2/TRINITY_DN306_c4_g1/TRINITY_DN40503_c0_g1/TRINITY_DN25570_c0_g1/TRINITY_DN10400_c0_g1/TRINITY_DN15930_c0_g1/TRINITY_DN4137_c2_g1/TRINITY_DN22707_c1_g1/TRINITY_DN3609_c1_g1/TRINITY_DN9485_c0_g1/TRINITY_DN3093_c0_g1/TRINITY_DN6589_c0_g1/TRINITY_DN7059_c0_g1/TRINITY_DN674_c2_g1/TRINITY_DN14850_c0_g1	
GO:0016485	protein processing	16/2626	71/33142	0.000111	0.001378	0.001032	TRINITY_DN256_c0_g1/TRINITY_DN3133_c1_g2/TRINITY_DN26373_c0_g1/TRINITY_DN1557_c0_g1/TRINITY_DN3503_c0_g1/TRINITY_DN12260_c0_g1/TRINITY_DN31763_c0_g1/TRINITY_DN6108_c0_g1/TRINITY_DN1797_c0_g1/TRINITY_DN6641_c0_g1/TRINITY_DN1085_c0_g1/TRINITY_DN9853_c0_g1/TRINITY_DN46894_c1_g1/TRINITY_DN9924_c0_g1/TRINITY_DN55372_c0_g1/TRINITY_DN2961_c0_g1	16
GO:0000122	negative regulation of transcription by RNA polymerase II	44/2626	309/33142	0.000112	0.001392	0.001042	TRINITY_DN14358_c0_g1/TRINITY_DN1893_c0_g1/TRINIT	44

Y_DN219_c3_g1/TRINITY_D
N11739_c0_g1/TRINITY_DN1
0890_c0_g1/TRINITY_DN439_
c0_g1/TRINITY_DN5239_c1_g
2/TRINITY_DN8236_c0_g1/TR
INITY_DN21131_c0_g2/TRINI
TY_DN22837_c0_g1/TRINITY
_DN4789_c0_g1/TRINITY_DN
12504_c0_g1/TRINITY_DN361
53_c0_g3/TRINITY_DN11693_
c0_g2/TRINITY_DN578_c1_g1
/TRINITY_DN4644_c0_g1/TRI
NITY_DN14383_c0_g1/TRINI
TY_DN13176_c0_g1/TRINITY
_DN3284_c0_g1/TRINITY_DN
29267_c0_g2/TRINITY_DN373
2_c0_g1/TRINITY_DN3713_c1
_g1/TRINITY_DN45371_c0_g1
/TRINITY_DN5296_c0_g1/TRI
NITY_DN12891_c0_g1/TRINI
TY_DN9245_c0_g1/TRINITY_
DN2095_c0_g1/TRINITY_DN3
722_c0_g1/TRINITY_DN12413
_c0_g1/TRINITY_DN2431_c0_
g1/TRINITY_DN13176_c0_g2/
TRINITY_DN1111_c0_g1/TRI
NITY_DN29974_c0_g1/TRINI
TY_DN40503_c0_g1/TRINITY
_DN10400_c0_g1/TRINITY_D
N51821_c0_g1/TRINITY_DN1
2512_c0_g1/TRINITY_DN489_
c1_g1/TRINITY_DN16221_c0_

insects MDPI

							g1/TRINITY_DN3609_c1_g1/TRINITY_DN10344_c0_g1/TRINITY_DN3093_c0_g1/TRINITY_DN6589_c0_g1/TRINITY_DN17100_c0_g1	
GO:0000943	retrotransposon nucleocapsid	20/2626	101/33142	0.000114	0.001404	0.001052	TRINITY_DN6070_c0_g1/TRINITY_DN20392_c0_g1/TRINITY_DN2636_c0_g2/TRINITY_DN1830_c0_g2/TRINITY_DN20442_c0_g1/TRINITY_DN23043_c0_g3/TRINITY_DN1326_c1_g1/TRINITY_DN8531_c0_g1/TRINITY_DN1252_c0_g1/TRINITY_DN60975_c0_g1/TRINITY_DN30295_c0_g1/TRINITY_DN3766_c0_g1/TRINITY_DN30055_c0_g1/TRINITY_DN57331_c0_g1/TRINITY_DN16018_c0_g1/TRINITY_DN12254_c0_g3/TRINITY_DN1618_c0_g1/TRINITY_DN1589_c5_g1/TRINITY_DN9422_c0_g1/TRINITY_DN11438_c0_g1	20
GO:0042552	myelination	7/2626	16/33142	0.000117	0.001438	0.001077	TRINITY_DN72971_c0_g1/TRINITY_DN15487_c0_g1/TRINITY_DN4826_c1_g1/TRINITY_DN15468_c0_g1/TRINITY_DN5431_c0_g1/TRINITY_DN43641_c0_g1/TRINITY_DN18471_c0_g2	7
GO:0004312	fatty acid synthase activity	10/2626	32/33142	0.000122	0.001449	0.001085	TRINITY_DN606_c3_g1/TRINITY_DN19001_c0_g1/TRINITY	10

							_DN23921_c0_g1/TRINITY_DN482_c1_g1/TRINITY_DN29856_c0_g1/TRINITY_DN1549_c0_g1/TRINITY_DN40189_c0_g1/TRINITY_DN484_c0_g1/TRINITY_DN244_c3_g1/TRINITY_DN61025_c0_g1	
GO:0008146	sulfotransferase activity	10/2626	32/33142	0.000122	0.001449	0.001085	TRINITY_DN44907_c0_g1/TRINITY_DN806_c0_g2/TRINITY_DN12529_c0_g1/TRINITY_DN6957_c0_g1/TRINITY_DN512_c1_g1/TRINITY_DN7730_c0_g4/TRINITY_DN806_c0_g1/TRINITY_DN1017_c0_g1/TRINITY_DN7903_c0_g1/TRINITY_DN3213_c4_g1	10
GO:0008659	(3R)-hydroxymyristoyl-[acyl-carrier-protein] dehydratase activity	10/2626	32/33142	0.000122	0.001449	0.001085	TRINITY_DN606_c3_g1/TRINITY_DN19001_c0_g1/TRINITY_DN23921_c0_g1/TRINITY_DN482_c1_g1/TRINITY_DN29856_c0_g1/TRINITY_DN1549_c0_g1/TRINITY_DN40189_c0_g1/TRINITY_DN484_c0_g1/TRINITY_DN244_c3_g1/TRINITY_DN61025_c0_g1	10
GO:0047451	3-hydroxyoctanoyl-[acyl-carrier-protein] dehydratase activity	10/2626	32/33142	0.000122	0.001449	0.001085	TRINITY_DN606_c3_g1/TRINITY_DN19001_c0_g1/TRINITY_DN23921_c0_g1/TRINITY_DN482_c1_g1/TRINITY_DN29856_c0_g1/TRINITY_DN1549_c0_g1/TRINITY_DN40189_c0_g1/TRINITY_DN484_c0_g1/TRI	10

							NITY_DN244_c3_g1/TRINITY_DN61025_c0_g1	
GO:0009953	dorsal/ventral pattern formation	11/2626	38/33142	0.000122	0.001449	0.001085	TRINITY_DN10890_c0_g1/TRINITY_DN11340_c0_g1/TRINITY_DN10159_c1_g1/TRINITY_DN4173_c0_g1/TRINITY_DN6641_c0_g2/TRINITY_DN306_c1_g2/TRINITY_DN6641_c0_g1/TRINITY_DN2095_c0_g1/TRINITY_DN3717_c0_g1/TRINITY_DN5802_c0_g1/TRINITY_DN13354_c0_g1	11
GO:0001917	photoreceptor inner segment	8/2626	21/33142	0.000122	0.001449	0.001085	TRINITY_DN19075_c0_g1/TRINITY_DN6174_c0_g1/TRINITY_DN47_c0_g1/TRINITY_DN31870_c0_g1/TRINITY_DN5459_c0_g1/TRINITY_DN20346_c0_g1/TRINITY_DN2746_c0_g1/TRINITY_DN6945_c0_g2	8
GO:0010243	response to organonitrogen compound	8/2626	21/33142	0.000122	0.001449	0.001085	TRINITY_DN7384_c0_g1/TRINITY_DN406_c0_g3/TRINITY_DN42262_c0_g1/TRINITY_DN5049_c0_g1/TRINITY_DN2999_c0_g1/TRINITY_DN406_c0_g2/TRINITY_DN1118_c0_g1/TRINITY_DN42565_c0_g1	8
GO:0048172	regulation of short-term neuronal synaptic plasticity	8/2626	21/33142	0.000122	0.001449	0.001085	TRINITY_DN21399_c0_g2/TRINITY_DN11938_c0_g1/TRINITY_DN1765_c2_g1/TRINITY_DN3816_c0_g1/TRINITY_DN11154_c0_g1/TRINITY_DN9041	8

insects MDPI

							_c0_g1/TRINITY_DN7200_c0_g2/TRINITY_DN9041_c0_g2	
GO:0007166	cell surface receptor signaling pathway	16/2626	72/33142	0.000132	0.00156	0.001168	TRINITY_DN14379_c0_g1/TRINITY_DN39639_c3_g1/TRINITY_DN938_c2_g1/TRINITY_DN7051_c0_g1/TRINITY_DN30087_c1_g2/TRINITY_DN8257_c0_g1/TRINITY_DN1451_c0_g2/TRINITY_DN10783_c0_g1/TRINITY_DN4391_c0_g1/TRINITY_DN1486_c0_g1/TRINITY_DN1451_c0_g1/TRINITY_DN9642_c1_g2/TRINITY_DN58254_c0_g1/TRINITY_DN58043_c0_g1/TRINITY_DN2285_c0_g1/TRINITY_DN864_c0_g3	16
GO:0001525	angiogenesis	15/2626	65/33142	0.000135	0.001593	0.001193	TRINITY_DN14379_c0_g1/TRINITY_DN3120_c0_g1/TRINITY_DN11100_c0_g1/TRINITY_DN1266_c0_g1/TRINITY_DN2557_c0_g2/TRINITY_DN85_c0_g1/TRINITY_DN1902_c0_g1/TRINITY_DN56782_c0_g1/TRINITY_DN9780_c0_g1/TRINITY_DN93_c0_g1/TRINITY_DN40503_c0_g1/TRINITY_DN2281_c0_g1/TRINITY_DN4137_c2_g1/TRINITY_DN14663_c0_g1/TRINITY_DN2285_c0_g1	15
GO:0030017	sarcomere	12/2626	45/33142	0.000145	0.001689	0.001265	TRINITY_DN725_c5_g1/TRINITY_DN1691_c0_g2/TRINITY_DN1908_c3_g1/TRINITY_DN9	12

insects MDPI

							219_c0_g1/TRINITY_DN36153_c0_g3/TRINITY_DN5519_c0_g1/TRINITY_DN5521_c0_g1/TRINITY_DN1174_c0_g1/TRINITY_DN2869_c2_g1/TRINITY_DN17422_c0_g2/TRINITY_DN2168_c3_g1/TRINITY_DN8235_c1_g1	
GO:0043524	negative regulation of neuron apoptotic process	12/2626	45/33142	0.000145	0.001689	0.001265	TRINITY_DN21399_c0_g2/TRINITY_DN3245_c0_g1/TRINITY_DN1487_c0_g2/TRINITY_DN1765_c2_g1/TRINITY_DN3816_c0_g1/TRINITY_DN9041_c0_g1/TRINITY_DN21152_c0_g1/TRINITY_DN7200_c0_g2/TRINITY_DN9041_c0_g2/TRINITY_DN12889_c0_g1/TRINITY_DN301_c4_g1/TRINITY_DN1908_c0_g1	12
GO:0042493	response to drug	27/2626	160/33142	0.000148	0.001702	0.001274	TRINITY_DN7384_c0_g1/TRINITY_DN5886_c0_g1/TRINITY_DN1568_c0_g1/TRINITY_DN7048_c0_g1/TRINITY_DN406_c0_g3/TRINITY_DN4514_c0_g1/TRINITY_DN42262_c0_g1/TRINITY_DN5049_c0_g1/TRINITY_DN23485_c0_g1/TRINITY_DN2999_c0_g1/TRINITY_DN17663_c0_g1/TRINITY_DN10011_c0_g1/TRINITY_DN1852_c2_g1/TRINITY_DN406_c0_g2/TRINITY_DN4859_c0_g1/T	27

insects MDPI

							RINITY_DN33241_c0_g1/TRINITY_DN40503_c0_g1/TRINITY_DN471_c1_g1/TRINITY_DN1118_c0_g1/TRINITY_DN13226_c0_g1/TRINITY_DN6985_c0_g1/TRINITY_DN16317_c0_g1/TRINITY_DN7318_c0_g1/TRINITY_DN42565_c0_g1/TRINITY_DN6589_c0_g1/TRINITY_DN10346_c1_g1/TRINITY_DN5788_c1_g1	
GO:0030414	peptidase inhibitor activity	6/2626	12/33142	0.00015	0.001702	0.001274	TRINITY_DN12244_c0_g1/TRINITY_DN3923_c0_g1/TRINITY_DN2018_c1_g1/TRINITY_DN13407_c0_g1/TRINITY_DN4266_c0_g3/TRINITY_DN3554_c0_g1	6
GO:0034625	fatty acid elongation, monounsaturated fatty acid	6/2626	12/33142	0.00015	0.001702	0.001274	TRINITY_DN5032_c1_g1/TRINITY_DN1854_c0_g2/TRINITY_DN7107_c0_g1/TRINITY_DN12176_c0_g1/TRINITY_DN34926_c1_g1/TRINITY_DN13730_c0_g1	6
GO:0035904	aorta development	6/2626	12/33142	0.00015	0.001702	0.001274	TRINITY_DN7437_c0_g1/TRINITY_DN1893_c0_g1/TRINITY_DN2448_c0_g1/TRINITY_DN9110_c0_g1/TRINITY_DN9158_c3_g1/TRINITY_DN709_c0_g1	6
GO:0044458	motile cilium assembly	6/2626	12/33142	0.00015	0.001702	0.001274	TRINITY_DN6657_c0_g1/TRINITY_DN6327_c1_g1/TRINITY_DN17079_c0_g1/TRINITY_	6

							DN20881_c0_g2/TRINITY_DN5914_c0_g1/TRINITY_DN63431_c0_g1	
GO:0071320	cellular response to cAMP	6/2626	12/33142	0.00015	0.001702	0.001274	TRINITY_DN16407_c1_g1/TRINITY_DN3713_c1_g1/TRINITY_DN11886_c0_g1/TRINITY_DN10344_c0_g1/TRINITY_DN7471_c0_g1/TRINITY_DN18471_c0_g2	6
GO:0030285	integral component of synaptic vesicle membrane	9/2626	27/33142	0.000152	0.001729	0.001294	TRINITY_DN4032_c0_g1/TRINITY_DN200_c0_g2/TRINITY_DN5859_c0_g1/TRINITY_DN27259_c0_g1/TRINITY_DN19635_c0_g1/TRINITY_DN7054_c0_g1/TRINITY_DN273_c1_g1/TRINITY_DN11061_c0_g2/TRINITY_DN4674_c0_g1	9
GO:0006869	lipid transport	22/2626	119/33142	0.000154	0.001735	0.001299	TRINITY_DN11463_c0_g1/TRINITY_DN72971_c0_g1/TRINITY_DN2516_c0_g1/TRINITY_DN12443_c0_g1/TRINITY_DN8493_c0_g1/TRINITY_DN17444_c0_g1/TRINITY_DN7641_c0_g1/TRINITY_DN7048_c0_g1/TRINITY_DN17444_c0_g2/TRINITY_DN5086_c0_g1/TRINITY_DN383_c0_g1/TRINITY_DN6767_c0_g1/TRINITY_DN17663_c0_g1/TRINITY_DN4826_c1_g1/TRINITY_DN2697_c1_g1/TRINITY_DN1998_c0_g1/TRINITY_DN7045_c1_g1/TRINIT	22

							Y_DN10060_c0_g1/TRINITY_DN39858_c0_g1/TRINITY_DN26496_c0_g1/TRINITY_DN4009_c0_g2/TRINITY_DN16662_c0_g1	
GO:0031430	M band	10/2626	33/33142	0.000162	0.001826	0.001367	TRINITY_DN725_c5_g1/TRINITY_DN1691_c0_g2/TRINITY_DN1908_c3_g1/TRINITY_DN13981_c0_g1/TRINITY_DN3732_c0_g1/TRINITY_DN5521_c0_g1/TRINITY_DN10569_c0_g2/TRINITY_DN471_c1_g1/TRINITY_DN39722_c0_g1/TRINITY_DN8235_c1_g1	10
GO:0033993	response to lipid	8/2626	22/33142	0.000179	0.002001	0.001498	TRINITY_DN1015_c0_g2/TRINITY_DN2378_c0_g1/TRINITY_DN1568_c0_g1/TRINITY_DN2675_c0_g1/TRINITY_DN8848_c0_g1/TRINITY_DN10757_c0_g1/TRINITY_DN42475_c0_g1/TRINITY_DN38748_c0_g1	8
GO:0008569	ATP-dependent microtubule motor activity, minus-end-directed	12/2626	46/33142	0.000182	0.00203	0.00152	TRINITY_DN9451_c0_g1/TRINITY_DN1979_c0_g2/TRINITY_DN1979_c0_g1/TRINITY_DN4543_c0_g2/TRINITY_DN3083_c0_g1/TRINITY_DN1979_c1_g1/TRINITY_DN18134_c0_g1/TRINITY_DN10356_c0_g1/TRINITY_DN31689_c0_g1/TRINITY_DN6282_c0_g1/TRINITY_DN9647_c0_g1/TRINITY_DN29956_c0_g1	12

insects MDPI

GO:0030855	epithelial cell differentiation	7/2626	17/33142	0.000186	0.002055	0.001538	TRINITY_DN5878_c0_g1/TRINITY_DN7743_c0_g1/TRINITY_DN8257_c0_g1/TRINITY_DN85_c0_g1/TRINITY_DN755_c1_g1/TRINITY_DN7476_c0_g1/TRINITY_DN111160_c0_g1	7
GO:0035338	long-chain fatty-acyl-CoA biosynthetic process	7/2626	17/33142	0.000186	0.002055	0.001538	TRINITY_DN5032_c1_g1/TRINITY_DN1854_c0_g2/TRINITY_DN2835_c0_g1/TRINITY_DN7107_c0_g1/TRINITY_DN12176_c0_g1/TRINITY_DN10258_c0_g1/TRINITY_DN13730_c0_g1	7
GO:0007224	smoothened signaling pathway	11/2626	40/33142	0.000202	0.002229	0.001669	TRINITY_DN6327_c1_g1/TRINITY_DN3375_c0_g1/TRINITY_DN5006_c0_g2/TRINITY_DN21912_c0_g1/TRINITY_DN4002_c0_g3/TRINITY_DN11340_c0_g1/TRINITY_DN306_c1_g2/TRINITY_DN2153_c0_g1/TRINITY_DN13467_c0_g1/TRINITY_DN15988_c0_g2/TRINITY_DN4002_c0_g1	11
GO:0000977	RNA polymerase II regulatory region sequence-specific DNA binding	23/2626	130/33142	0.000217	0.002389	0.001788	TRINITY_DN2578_c0_g1/TRINITY_DN1893_c0_g1/TRINITY_DN10890_c0_g1/TRINITY_DN4336_c0_g2/TRINITY_DN8903_c0_g1/TRINITY_DN12504_c0_g1/TRINITY_DN14383_c0_g1/TRINITY_DN13176_c0_g1/TRINITY_DN3284_c0_g1/TRINITY_DN29267_c0_g2/TRINI	23

insects MDPI

							TY_DN49948_c0_g1/TRINITY_DN21038_c0_g2/TRINITY_DN2095_c0_g1/TRINITY_DN12413_c0_g1/TRINITY_DN13176_c0_g2/TRINITY_DN1111_c0_g1/TRINITY_DN40503_c0_g1/TRINITY_DN12512_c0_g1/TRINITY_DN4137_c2_g1/TRINITY_DN16221_c0_g1/TRINITY_DN9485_c0_g1/TRINITY_DN3093_c0_g1/TRINITY_DN674_c2_g1	
GO:0002020	protease binding	12/2626	47/33142	0.000227	0.002481	0.001858	TRINITY_DN72971_c0_g1/TRINITY_DN1691_c0_g2/TRINITY_DN5613_c1_g1/TRINITY_DN1557_c0_g1/TRINITY_DN1908_c3_g1/TRINITY_DN4826_c1_g1/TRINITY_DN56782_c0_g1/TRINITY_DN40503_c0_g1/TRINITY_DN4641_c0_g1/TRINITY_DN5031_c0_g1/TRINITY_DN43641_c0_g1/TRINITY_DN2961_c0_g1	12
GO:0005604	basement membrane	13/2626	54/33142	0.000238	0.002569	0.001923	TRINITY_DN5310_c0_g2/TRINITY_DN6305_c1_g1/TRINITY_DN6886_c3_g1/TRINITY_DN2738_c0_g1/TRINITY_DN1120_c0_g2/TRINITY_DN7712_c0_g1/TRINITY_DN8217_c0_g1/TRINITY_DN740_c0_g1/TRINITY_DN2598_c0_g2/TRINITY_DN18049_c0_g1/TRINITY_	13

							DN2868_c0_g1/TRINITY_DN35269_c0_g1/TRINITY_DN2281_c0_g1	
GO:0043087	regulation of GTPase activity	13/2626	54/33142	0.000238	0.002569	0.001923	TRINITY_DN2489_c0_g1/TRINITY_DN22690_c0_g1/TRINITY_DN2738_c0_g1/TRINITY_DN8297_c0_g1/TRINITY_DN740_c0_g1/TRINITY_DN4391_c0_g1/TRINITY_DN2087_c0_g1/TRINITY_DN2598_c0_g2/TRINITY_DN9935_c1_g1/TRINITY_DN24893_c0_g1/TRINITY_DN10956_c0_g1/TRINITY_DN2078_c0_g2/TRINITY_DN14663_c0_g1	13
GO:0070330	aromatase activity	13/2626	54/33142	0.000238	0.002569	0.001923	TRINITY_DN788_c0_g2/TRINITY_DN9929_c0_g1/TRINITY_DN80619_c0_g2/TRINITY_DN6729_c0_g2/TRINITY_DN6729_c0_g3/TRINITY_DN3648_c0_g6/TRINITY_DN6010_c3_g1/TRINITY_DN996_c0_g1/TRINITY_DN5213_c0_g1/TRINITY_DN25933_c0_g2/TRINITY_DN3753_c0_g1/TRINITY_DN5625_c1_g1/TRINITY_DN23688_c0_g1	13
GO:0005911	cell-cell junction	14/2626	61/33142	0.000238	0.002569	0.001923	TRINITY_DN2244_c1_g1/TRINITY_DN36172_c0_g1/TRINITY_DN2786_c0_g1/TRINITY_DN31967_c0_g1/TRINITY_DN1614_c0_g1/TRINITY_DN1014	14

							5_c1_g1/TRINITY_DN43399_c0_g1/TRINITY_DN27045_c0_g1/TRINITY_DN2475_c0_g1/TRINITY_DN12127_c0_g1/TRINITY_DN446_c1_g1/TRINITY_DN11061_c0_g2/TRINITY_DN9850_c0_g1/TRINITY_DN7084_c0_g1	
GO:0061098	positive regulation of protein tyrosine kinase activity	6/2626	13/33142	0.000259	0.002781	0.002082	TRINITY_DN3991_c0_g1/TRINITY_DN2738_c0_g1/TRINITY_DN740_c0_g1/TRINITY_DN2598_c0_g2/TRINITY_DN2682_c0_g1/TRINITY_DN7471_c0_g1	6
GO:0031624	ubiquitin conjugating enzyme binding	9/2626	29/33142	0.000282	0.003014	0.002256	TRINITY_DN21399_c0_g2/TRINITY_DN8061_c0_g1/TRINITY_DN755_c1_g1/TRINITY_DN1765_c2_g1/TRINITY_DN3816_c0_g1/TRINITY_DN9041_c0_g1/TRINITY_DN7200_c0_g2/TRINITY_DN9041_c0_g2/TRINITY_DN11447_c0_g1	9
GO:0006171	cAMP biosynthetic process	7/2626	18/33142	0.000283	0.003014	0.002257	TRINITY_DN17778_c0_g1/TRINITY_DN20653_c0_g1/TRINITY_DN20653_c0_g4/TRINITY_DN3665_c0_g1/TRINITY_DN6839_c2_g1/TRINITY_DN20653_c0_g2/TRINITY_DN26727_c0_g1	7
GO:0007568	aging	14/2626	62/33142	0.000286	0.003032	0.00227	TRINITY_DN77781_c0_g1/TRINITY_DN60483_c1_g1/TRINITY_DN3007_c0_g1/TRINITY_	14

							DN17663_c0_g1/TRINITY_DN2779_c1_g2/TRINITY_DN19321_c0_g1/TRINITY_DN22381_c0_g1/TRINITY_DN1797_c0_g1/TRINITY_DN31435_c0_g1/TRINITY_DN9175_c0_g1/TRINITY_DN2779_c1_g3/TRINITY_DN33241_c0_g1/TRINITY_DN6985_c0_g1/TRINITY_DN12958_c0_g1	
GO:0030659	cytoplasmic vesicle membrane	16/2626	77/33142	0.000301	0.003179	0.00238	TRINITY_DN473_c0_g1/TRINITY_DN3899_c0_g1/TRINITY_DN9564_c0_g1/TRINITY_DN6222_c0_g1/TRINITY_DN3507_c0_g1/TRINITY_DN16407_c1_g1/TRINITY_DN1482_c0_g1/TRINITY_DN3180_c0_g1/TRINITY_DN8504_c0_g1/TRINITY_DN11886_c0_g1/TRINITY_DN7113_c0_g1/TRINITY_DN1482_c0_g2/TRINITY_DN55372_c0_g1/TRINITY_DN3255_c0_g2/TRINITY_DN11447_c0_g1/TRINITY_DN5075_c0_g1	16
GO:0046982	protein heterodimerization activity	42/2626	304/33142	0.000306	0.003219	0.00241	TRINITY_DN17778_c0_g1/TRINITY_DN11558_c1_g1/TRINITY_DN16186_c0_g1/TRINITY_DN8167_c0_g1/TRINITY_DN8805_c1_g1/TRINITY_DN20653_c0_g1/TRINITY_DN4789_c0_g1/TRINITY_DN1229_c2_g1/TRINITY_DN57132_c0_g2/TRI	42

							NITY_DN3713_c1_g1/TRINITY_DN17565_c0_g1/TRINITY_DN49948_c0_g1/TRINITY_DN13614_c1_g1/TRINITY_DN1473_c2_g1/TRINITY_DN974_c0_g1/TRINITY_DN13726_c0_g1/TRINITY_DN9684_c1_g1/TRINITY_DN4620_c0_g1/TRINITY_DN10757_c0_g1/TRINITY_DN3722_c0_g1/TRINITY_DN84830_c0_g1/TRINITY_DN3743_c0_g1/TRINITY_DN23750_c0_g1/TRINITY_DN2654_c0_g1/TRINITY_DN17694_c0_g1/TRINITY_DN2746_c0_g1/TRINITY_DN2165_c3_g1/TRINITY_DN29974_c0_g1/TRINITY_DN40503_c0_g1/TRINITY_DN6839_c2_g1/TRINITY_DN13727_c0_g1/TRINITY_DN14132_c0_g1/TRINITY_DN3238_c0_g1/TRINITY_DN4137_c2_g1/TRINITY_DN12650_c0_g1/TRINITY_DN9107_c0_g1/TRINITY_DN829_c0_g3/TRINITY_DN13252_c0_g1/TRINITY_DN9485_c0_g1/TRINITY_DN6505_c0_g1/TRINITY_DN6589_c0_g1/TRINITY_DN38748_c0_g1	
GO:0007507	heart development	23/2626	133/33142	0.000307	0.003219	0.00241	TRINITY_DN14379_c0_g1/TRINITY_DN1691_c0_g2/TRINITY_DN22536_c0_g1/TRINITY_	23

GO:0030246	carbohydrate binding	28/2626	176/33142	0.000311	0.003252	0.002435	DN1557_c0_g1/TRINITY_DN1908_c3_g1/TRINITY_DN5216_c1_g1/TRINITY_DN9451_c0_g1/TRINITY_DN19269_c0_g1/TRINITY_DN14383_c0_g1/TRINITY_DN3284_c0_g1/TRINITY_DN85_c0_g1/TRINITY_DN18134_c0_g1/TRINITY_DN306_c1_g2/TRINITY_DN35269_c0_g1/TRINITY_DN13564_c0_g1/TRINITY_DN2292_c2_g1/TRINITY_DN9158_c3_g1/TRINITY_DN339_c0_g2/TRINITY_DN11825_c0_g1/TRINITY_DN709_c0_g1/TRINITY_DN489_c1_g1/TRINITY_DN3299_c0_g1/TRINITY_DN4381_c0_g1 TRINITY_DN737_c0_g1/TRINITY_DN7148_c1_g1/TRINITY_DN83267_c0_g1/TRINITY_DN1686_c0_g1/TRINITY_DN1533_c0_g2/TRINITY_DN388_c0_g1/TRINITY_DN34715_c0_g1/TRINITY_DN56882_c0_g1/TRINITY_DN12607_c0_g1/TRINITY_DN683_c0_g1/TRINITY_DN4210_c0_g1/TRINITY_DN2117_c0_g2/TRINITY_DN9492_c0_g1/TRINITY_DN13138_c0_g1/TRINITY_DN9492_c0_g2/TRINITY_DN56756_c0_g1/TRINITY_DN5869_c0_g2/TRINITY_	28

							DN13662_c0_g1/TRINITY_DN8043_c0_g1/TRINITY_DN26993_c0_g1/TRINITY_DN6892_c0_g1/TRINITY_DN25356_c0_g1/TRINITY_DN51228_c1_g1/TRINITY_DN5431_c0_g1/TRINITY_DN1598_c1_g1/TRINITY_DN9937_c0_g1/TRINITY_DN6757_c0_g1/TRINITY_DN3178_c0_g1	
GO:0004190	aspartic-type endopeptidase activity	37/2626	258/33142	0.000328	0.003413	0.002555	TRINITY_DN3270_c0_g1/TRINITY_DN6070_c0_g1/TRINITY_DN20392_c0_g1/TRINITY_DN2636_c0_g2/TRINITY_DN1830_c0_g2/TRINITY_DN20442_c0_g1/TRINITY_DN5348_c0_g2/TRINITY_DN1557_c0_g1/TRINITY_DN23043_c0_g3/TRINITY_DN1090_c1_g2/TRINITY_DN1326_c1_g1/TRINITY_DN8531_c0_g1/TRINITY_DN2943_c1_g1/TRINITY_DN9542_c0_g1/TRINITY_DN12254_c0_g1/TRINITY_DN1252_c0_g1/TRINITY_DN24885_c0_g1/TRINITY_DN60975_c0_g1/TRINITY_DN154919_c0_g1/TRINITY_DN1085_c0_g1/TRINITY_DN1874_c0_g1/TRINITY_DN30295_c0_g1/TRINITY_DN9090_c0_g2/TRINITY_DN3766_c0_g1/TRINITY_DN9861_c0_g1/TRIN	37

							ITY_DN30055_c0_g1/TRINITY_DN5009_c0_g2/TRINITY_DN57331_c0_g1/TRINITY_DN16018_c0_g1/TRINITY_DN12254_c0_g3/TRINITY_DN1618_c0_g1/TRINITY_DN1589_c5_g1/TRINITY_DN13245_c0_g1/TRINITY_DN3084_c0_g1/TRINITY_DN38702_c0_g1/TRINITY_DN9422_c0_g1/TRINITY_DN11438_c0_g1	
GO:0034765	regulation of ion transmembrane transport	14/2626	63/33142	0.000341	0.003537	0.002649	TRINITY_DN1748_c0_g1/TRINITY_DN5216_c1_g1/TRINITY_DN10654_c0_g1/TRINITY_DN12107_c0_g1/TRINITY_DN16407_c1_g1/TRINITY_DN15120_c0_g1/TRINITY_DN16593_c0_g1/TRINITY_DN13726_c0_g1/TRINITY_DN14953_c0_g3/TRINITY_DN9551_c0_g3/TRINITY_DN17398_c0_g2/TRINITY_DN4779_c2_g1/TRINITY_DN2003_c0_g1/TRINITY_DN16754_c0_g1	14
GO:0008354	germ cell migration	11/2626	43/33142	0.000403	0.004169	0.003122	TRINITY_DN3284_c0_g1/TRINITY_DN1216_c2_g1/TRINITY_DN23353_c2_g1/TRINITY_DN5370_c0_g1/TRINITY_DN520_c0_g1/TRINITY_DN346_c0_g1/TRINITY_DN339_c0_g2/TRINITY_DN3459_c0_g1/TRINITY_DN3241_c0_g1/TRINITY_	11

							DN23609_c0_g1/TRINITY_DN12338_c0_g1	
GO:0004016	adenylate cyclase activity	7/2626	19/33142	0.000417	0.00427	0.003197	TRINITY_DN17778_c0_g1/TRINITY_DN20653_c0_g1/TRINITY_DN20653_c0_g4/TRINITY_DN3665_c0_g1/TRINITY_DN6839_c2_g1/TRINITY_DN20653_c0_g2/TRINITY_DN26727_c0_g1	7
GO:0015114	phosphate ion transmembrane transporter activity	7/2626	19/33142	0.000417	0.00427	0.003197	TRINITY_DN3268_c0_g1/TRINITY_DN149_c1_g1/TRINITY_DN14068_c0_g2/TRINITY_DN14068_c0_g1/TRINITY_DN14068_c0_g3/TRINITY_DN9004_c0_g1/TRINITY_DN46_c0_g1	7
GO:0031100	animal organ regeneration	7/2626	19/33142	0.000417	0.00427	0.003197	TRINITY_DN3991_c0_g1/TRINITY_DN32009_c0_g1/TRINITY_DN11954_c0_g1/TRINITY_DN2682_c0_g1/TRINITY_DN974_c0_g1/TRINITY_DN9853_c0_g1/TRINITY_DN15324_c1_g1	7
GO:0001227	DNA-binding transcription repressor activity, RNA polymerase II-specific	12/2626	50/33142	0.000422	0.004295	0.003216	TRINITY_DN1893_c0_g1/TRINITY_DN5239_c1_g2/TRINITY_DN8167_c0_g1/TRINITY_DN2843_c0_g2/TRINITY_DN13176_c0_g1/TRINITY_DN29267_c0_g2/TRINITY_DN49948_c0_g1/TRINITY_DN2095_c0_g1/TRINITY_DN3722_c0_g1/TRINITY_DN13176_c0_g2/TRINI	12

							TY_DN5956_c0_g2/TRINITY_DN17100_c0_g1	
GO:0007509	mesoderm migration involved in gastrulation	6/2626	14/33142	0.000423	0.004295	0.003216	TRINITY_DN39639_c3_g1/TRINITY_DN806_c0_g2/TRINITY_DN9642_c1_g2/TRINITY_DN339_c0_g2/TRINITY_DN806_c0_g1/TRINITY_DN864_c0_g3	6
GO:0015074	DNA integration	50/2626	387/33142	0.000442	0.004475	0.00335	TRINITY_DN3270_c0_g1/TRINITY_DN6070_c0_g1/TRINITY_DN20392_c0_g1/TRINITY_DN2636_c0_g2/TRINITY_DN1830_c0_g2/TRINITY_DN20442_c0_g1/TRINITY_DN459_c1_g1/TRINITY_DN5348_c0_g2/TRINITY_DN5356_c0_g1/TRINITY_DN1817_c1_g1/TRINITY_DN23043_c0_g3/TRINITY_DN1738_c0_g1/TRINITY_DN12282_c1_g1/TRINITY_DN1090_c1_g2/TRINITY_DN26151_c0_g2/TRINITY_DN1326_c1_g1/TRINITY_DN8531_c0_g1/TRINITY_DN21282_c0_g1/TRINITY_DN9542_c0_g1/TRINITY_DN12254_c0_g1/TRINITY_DN6054_c0_g1/TRINITY_DN1252_c0_g1/TRINITY_DN60975_c0_g1/TRINITY_DN154919_c0_g1/TRINITY_DN1874_c0_g1/TRINITY_DN30295_c0_g1/TRINITY_DN16389_c0_g1/TRINITY_DN9090_c0_g2/TRINITY_DN66	50

							443_c0_g1/TRINITY_DN3766_c0_g1/TRINITY_DN9861_c0_g1/TRINITY_DN30055_c0_g1/TRINITY_DN4190_c0_g1/TRINITY_DN716_c0_g1/TRINITY_DN23887_c0_g1/TRINITY_DN3333_c0_g1/TRINITY_DN57331_c0_g1/TRINITY_DN16018_c0_g1/TRINITY_DN12254_c0_g3/TRINITY_DN1618_c0_g1/TRINITY_DN1589_c5_g1/TRINITY_DN13245_c0_g1/TRINITY_DN13637_c0_g1/TRINITY_DN3084_c0_g1/TRINITY_DN38702_c0_g1/TRINITY_DN9422_c0_g1/TRINITY_DN88_c0_g2/TRINITY_DN11438_c0_g1/TRINITY_DN7448_c0_g1/TRINITY_DN31780_c0_g2	
GO:0007586	digestion	10/2626	37/33142	0.000456	0.00458	0.003429	TRINITY_DN2384_c0_g1/TRINITY_DN11818_c0_g1/TRINITY_DN10126_c0_g1/TRINITY_DN2648_c0_g1/TRINITY_DN21814_c0_g2/TRINITY_DN309_c0_g1/TRINITY_DN4485_c0_g1/TRINITY_DN1773_c1_g2/TRINITY_DN5833_c0_g1/TRINITY_DN32069_c0_g1	10
GO:0008202	steroid metabolic process	10/2626	37/33142	0.000456	0.00458	0.003429	TRINITY_DN33627_c0_g1/TRINITY_DN2751_c1_g2/TRINITY_DN2751_c1_g3/TRINITY_DN2751_c1_g1/TRINITY_DN54	10

							58_c0_g1/TRINITY_DN8091_c0_g1/TRINITY_DN10873_c0_g1/TRINITY_DN10583_c0_g1/TRINITY_DN9063_c0_g1/TRINITY_DN7730_c0_g4	
GO:0030018	Z disc	16/2626	80/33142	0.000473	0.004723	0.003536	TRINITY_DN1691_c0_g2/TRINITY_DN8299_c0_g1/TRINITY_DN1908_c3_g1/TRINITY_DN13981_c0_g1/TRINITY_DN2021_c1_g1/TRINITY_DN3732_c0_g1/TRINITY_DN5519_c0_g1/TRINITY_DN3180_c0_g1/TRINITY_DN4441_c3_g1/TRINITY_DN5521_c0_g1/TRINITY_DN10569_c0_g2/TRINITY_DN716_c0_g1/TRINITY_DN39722_c0_g1/TRINITY_DN15445_c0_g1/TRINITY_DN9055_c0_g2/TRINITY_DN8235_c1_g1	16
GO:0004519	endonuclease activity	46/2626	349/33142	0.000474	0.004723	0.003536	TRINITY_DN3270_c0_g1/TRINITY_DN6070_c0_g1/TRINITY_DN20392_c0_g1/TRINITY_DN2636_c0_g2/TRINITY_DN20442_c0_g1/TRINITY_DN38264_c0_g1/TRINITY_DN459_c1_g1/TRINITY_DN5348_c0_g2/TRINITY_DN3284_c2_g1/TRINITY_DN23043_c0_g3/TRINITY_DN9571_c0_g2/TRINITY_DN1090_c1_g2/TRINITY_DN1340_c0_g1/TRINITY_DN1326_c1_g1/TRINITY_DN8531_c0_g1/T	46

insects MDPI

							RINITY_DN5794_c0_g1/TRINITY_DN2943_c1_g1/TRINITY_DN14935_c1_g1/TRINITY_DN9542_c0_g1/TRINITY_DN12254_c0_g1/TRINITY_DN302_c0_g3/TRINITY_DN27747_c0_g1/TRINITY_DN154919_c0_g1/TRINITY_DN1874_c0_g1/TRINITY_DN30295_c0_g1/TRINITY_DN9090_c0_g2/TRINITY_DN3766_c0_g1/TRINITY_DN9861_c0_g1/TRINITY_DN5389_c0_g1/TRINITY_DN2240_c0_g1/TRINITY_DN30055_c0_g1/TRINITY_DN4190_c0_g1/TRINITY_DN10695_c0_g1/TRINITY_DN716_c0_g1/TRINITY_DN533_c4_g1/TRINITY_DN57331_c0_g1/TRINITY_DN30956_c0_g1/TRINITY_DN16018_c0_g1/TRINITY_DN1618_c0_g1/TRINITY_DN141_c0_g1/TRINITY_DN6581_c0_g1/TRINITY_DN9541_c0_g2/TRINITY_DN3084_c0_g1/TRINITY_DN38702_c0_g1/TRINITY_DN9422_c0_g1/TRINITY_DN11438_c0_g1	
GO:0008284	positive regulation of cell population proliferation	23/2626	137/33142	0.000475	0.004723	0.003536	TRINITY_DN5239_c1_g2/TRINITY_DN9375_c0_g1/TRINITY_DN6108_c0_g1/TRINITY_DN7712_c0_g1/TRINITY_DN85_c0_g1/TRINITY_DN6348_c0_	23

							g1/TRINITY_DN17565_c0_g1/TRINITY_DN2327_c0_g1/TRINITY_DN15112_c0_g1/TRINITY_DN2352_c0_g2/TRINITY_DN6093_c0_g2/TRINITY_DN93_c0_g1/TRINITY_DN10426_c0_g1/TRINITY_DN40503_c0_g1/TRINITY_DN339_c0_g2/TRINITY_DN645_c0_g1/TRINITY_DN6985_c0_g1/TRINITY_DN10063_c0_g1/TRINITY_DN2321_c0_g1/TRINITY_DN1071_c0_g1/TRINITY_DN12889_c0_g1/TRINITY_DN23722_c0_g1/TRINITY_DN4416_c0_g1	
GO:0007204	positive regulation of cytosolic calcium ion concentration	8/2626	25/33142	0.000487	0.004794	0.003589	TRINITY_DN17778_c0_g1/TRINITY_DN20653_c0_g1/TRINITY_DN22381_c0_g1/TRINITY_DN20653_c0_g4/TRINITY_DN31435_c0_g1/TRINITY_DN6839_c2_g1/TRINITY_DN6945_c0_g2/TRINITY_DN20653_c0_g2	8
GO:0044297	cell body	8/2626	25/33142	0.000487	0.004794	0.003589	TRINITY_DN954_c0_g1/TRINITY_DN18321_c0_g1/TRINITY_DN233_c0_g1/TRINITY_DN10760_c0_g1/TRINITY_DN5121_c0_g1/TRINITY_DN39318_c0_g1/TRINITY_DN2961_c0_g1/TRINITY_DN8692_c0_g1	8
GO:0097110	scaffold protein binding	8/2626	25/33142	0.000487	0.004794	0.003589	TRINITY_DN17778_c0_g1/TRINITY_DN20653_c0_g1/TRINI	8

							TY_DN16407_c1_g1/TRINITY_DN15308_c0_g2/TRINITY_DN6839_c2_g1/TRINITY_DN7687_c0_g1/TRINITY_DN7523_c0_g1/TRINITY_DN428_c0_g1	
GO:0008328	ionotropic glutamate receptor complex	5/2626	10/33142	0.000558	0.005363	0.004015	TRINITY_DN21399_c0_g2/TRINITY_DN9333_c0_g1/TRINITY_DN3816_c0_g1/TRINITY_DN15420_c0_g1/TRINITY_DN7200_c0_g2	5
GO:0030240	skeletal muscle thin filament assembly	5/2626	10/33142	0.000558	0.005363	0.004015	TRINITY_DN1691_c0_g2/TRINITY_DN1908_c3_g1/TRINITY_DN2424_c0_g1/TRINITY_DN4325_c0_g1/TRINITY_DN982_c0_g1	5
GO:0032310	prostaglandin secretion	5/2626	10/33142	0.000558	0.005363	0.004015	TRINITY_DN406_c0_g3/TRINITY_DN5049_c0_g1/TRINITY_DN2999_c0_g1/TRINITY_DN406_c0_g2/TRINITY_DN42565_c0_g1	5
GO:0034097	response to cytokine	5/2626	10/33142	0.000558	0.005363	0.004015	TRINITY_DN10890_c0_g1/TRINITY_DN4861_c0_g1/TRINITY_DN7743_c0_g1/TRINITY_DN7326_c0_g1/TRINITY_DN6589_c0_g1	5
GO:0090102	cochlea development	5/2626	10/33142	0.000558	0.005363	0.004015	TRINITY_DN72971_c0_g1/TRINITY_DN4826_c1_g1/TRINITY_DN306_c1_g2/TRINITY_DN43641_c0_g1/TRINITY_DN7318_c0_g1	5
GO:0090175	regulation of establishment of planar polarity	5/2626	10/33142	0.000558	0.005363	0.004015	TRINITY_DN2244_c1_g1/TRINITY_DN43399_c0_g1/TRINI	5

							TY_DN27045_c0_g1/TRINITY_DN446_c1_g1/TRINITY_DN10063_c0_g1	
GO:2000379	positive regulation of reactive oxygen species metabolic process	5/2626	10/33142	0.000558	0.005363	0.004015	TRINITY_DN3120_c0_g1/TRINITY_DN922_c0_g1/TRINITY_DN10426_c0_g1/TRINITY_DN56840_c0_g1/TRINITY_DN7471_c0_g1	5
GO:0070593	dendrite self-avoidance	10/2626	38/33142	0.000575	0.005504	0.004121	TRINITY_DN2244_c1_g1/TRINITY_DN2868_c0_g2/TRINITY_DN6076_c0_g1/TRINITY_DN8217_c1_g1/TRINITY_DN43399_c0_g1/TRINITY_DN7898_c0_g1/TRINITY_DN27045_c0_g1/TRINITY_DN12452_c0_g1/TRINITY_DN446_c1_g1/TRINITY_DN13354_c0_g1	10
GO:0006909	phagocytosis	7/2626	20/33142	0.000598	0.005669	0.004245	TRINITY_DN47_c0_g1/TRINITY_DN542_c0_g2/TRINITY_DN15399_c0_g1/TRINITY_DN5086_c0_g1/TRINITY_DN13394_c0_g1/TRINITY_DN7045_c1_g1/TRINITY_DN16986_c0_g2	7
GO:0042761	very long-chain fatty acid biosynthetic process	7/2626	20/33142	0.000598	0.005669	0.004245	TRINITY_DN5032_c1_g1/TRINITY_DN1854_c0_g2/TRINITY_DN7107_c0_g1/TRINITY_DN12176_c0_g1/TRINITY_DN34926_c1_g1/TRINITY_DN10258_c0_g1/TRINITY_DN13730_c0_g1	7
GO:0080025	phosphatidylinositol-3, 5-bisphosphate binding	7/2626	20/33142	0.000598	0.005669	0.004245	TRINITY_DN19161_c0_g1/TRINITY_DN12001_c0_g1/TRINI	7

							TY_DN21883_c0_g1/TRINITY_DN13153_c0_g1/TRINITY_DN1812_c0_g1/TRINITY_DN75_c2_g1/TRINITY_DN6505_c0_g1	
GO:0005770	late endosome	15/2626	74/33142	0.000606	0.005717	0.00428	TRINITY_DN72971_c0_g1/TRINITY_DN8493_c0_g1/TRINITY_DN43642_c0_g1/TRINITY_DN11355_c0_g3/TRINITY_DN1568_c0_g1/TRINITY_DN2629_c0_g1/TRINITY_DN200_c0_g2/TRINITY_DN36153_c0_g3/TRINITY_DN4826_c1_g1/TRINITY_DN16407_c1_g1/TRINITY_DN1532_c0_g1/TRINITY_DN901_c0_g1/TRINITY_DN43641_c0_g1/TRINITY_DN7084_c0_g1/TRINITY_DN901_c0_g2	15
GO:0098794	postsynapse	12/2626	52/33142	0.000618	0.00582	0.004358	TRINITY_DN21399_c0_g2/TRINITY_DN1765_c2_g1/TRINITY_DN3816_c0_g1/TRINITY_DN9935_c1_g1/TRINITY_DN7687_c0_g1/TRINITY_DN15930_c0_g1/TRINITY_DN7200_c0_g2/TRINITY_DN6505_c0_g1/TRINITY_DN4674_c0_g1/TRINITY_DN4487_c3_g2/TRINITY_DN14134_c0_g1/TRINITY_DN18471_c0_g2	12
GO:0035176	social behavior	8/2626	26/33142	0.000655	0.005932	0.004442	TRINITY_DN5239_c1_g2/TRINITY_DN9375_c0_g1/TRINITY_DN85_c0_g1/TRINITY_DN	8

							15308_c0_g2/TRINITY_DN2095_c0_g1/TRINITY_DN15930_c0_g1/TRINITY_DN7523_c0_g1/TRINITY_DN428_c0_g1	
GO:0051384	response to glucocorticoid	8/2626	26/33142	0.000655	0.005932	0.004442	TRINITY_DN1557_c0_g1/TRINITY_DN1568_c0_g1/TRINITY_DN4514_c0_g1/TRINITY_DN2675_c0_g1/TRINITY_DN1852_c2_g1/TRINITY_DN6037_c0_g1/TRINITY_DN6985_c0_g1/TRINITY_DN4895_c1_g1	8
GO:0102336	3-oxo-arachidoyl-CoA synthase activity	8/2626	26/33142	0.000655	0.005932	0.004442	TRINITY_DN5032_c1_g1/TRINITY_DN1854_c0_g2/TRINITY_DN2199_c2_g2/TRINITY_DN7107_c0_g1/TRINITY_DN12176_c0_g1/TRINITY_DN34926_c1_g1/TRINITY_DN10258_c0_g1/TRINITY_DN13730_c0_g1	8
GO:0102337	3-oxo-cerotoyl-CoA synthase activity	8/2626	26/33142	0.000655	0.005932	0.004442	TRINITY_DN5032_c1_g1/TRINITY_DN1854_c0_g2/TRINITY_DN2199_c2_g2/TRINITY_DN7107_c0_g1/TRINITY_DN12176_c0_g1/TRINITY_DN34926_c1_g1/TRINITY_DN10258_c0_g1/TRINITY_DN13730_c0_g1	8
GO:0102338	3-oxo-lignoceronyl-CoA synthase activity	8/2626	26/33142	0.000655	0.005932	0.004442	TRINITY_DN5032_c1_g1/TRINITY_DN1854_c0_g2/TRINITY_DN2199_c2_g2/TRINITY_DN7107_c0_g1/TRINITY_DN12176_c0_g1/TRINITY_DN34926_c1_g1/TRINITY_DN10258_c0_g1/TRINITY_DN13730_c0_g1	8

GO:0102756	very-long-chain 3-ketoacyl-CoA synthase activity	8/2626	26/33142	0.000655	0.005932	0.004442	TRINITY_DN5032_c1_g1/TRINITY_DN1854_c0_g2/TRINITY_DN2199_c2_g2/TRINITY_DN7107_c0_g1/TRINITY_DN12176_c0_g1/TRINITY_DN34926_c1_g1/TRINITY_DN10258_c0_g1/TRINITY_DN13730_c0_g1	8
GO:1905515	non-motile cilium assembly	8/2626	26/33142	0.000655	0.005932	0.004442	TRINITY_DN11509_c0_g1/TRINITY_DN6327_c1_g1/TRINITY_DN19269_c0_g1/TRINITY_DN4002_c0_g3/TRINITY_DN11340_c0_g1/TRINITY_DN306_c1_g2/TRINITY_DN9168_c0_g2/TRINITY_DN4002_c0_g1	8
GO:0030301	cholesterol transport	6/2626	15/33142	0.000658	0.005932	0.004442	TRINITY_DN1568_c0_g1/TRINITY_DN7048_c0_g1/TRINITY_DN5086_c0_g1/TRINITY_DN5278_c0_g1/TRINITY_DN10757_c0_g1/TRINITY_DN38748_c0_g1	6
GO:0031674	I band	6/2626	15/33142	0.000658	0.005932	0.004442	TRINITY_DN725_c5_g1/TRINITY_DN1691_c0_g2/TRINITY_DN1908_c3_g1/TRINITY_DN13981_c0_g1/TRINITY_DN5519_c0_g1/TRINITY_DN471_c1_g1	6
GO:0042472	inner ear morphogenesis	6/2626	15/33142	0.000658	0.005932	0.004442	TRINITY_DN9358_c0_g1/TRINITY_DN6174_c0_g1/TRINITY_DN47_c0_g1/TRINITY_DN7712_c0_g1/TRINITY_DN85_c0_g1/TRINITY_DN7759_c0_g1	6

GO:0043014	alpha-tubulin binding	6/2626	15/33142	0.000658	0.005932	0.004442	TRINITY_DN18454_c0_g1/TRINITY_DN954_c0_g1/TRINITY_DN233_c0_g1/TRINITY_DN7131_c0_g1/TRINITY_DN2372_c0_g1/TRINITY_DN953_c0_g1	6
GO:0051968	positive regulation of synaptic transmission, glutamatergic	6/2626	15/33142	0.000658	0.005932	0.004442	TRINITY_DN9375_c0_g1/TRINITY_DN2557_c0_g2/TRINITY_DN9333_c0_g1/TRINITY_DN15420_c0_g1/TRINITY_DN7687_c0_g1/TRINITY_DN428_c0_g1	6
GO:1902017	regulation of cilium assembly	6/2626	15/33142	0.000658	0.005932	0.004442	TRINITY_DN7371_c0_g1/TRINITY_DN36172_c0_g1/TRINITY_DN12886_c0_g1/TRINITY_DN306_c1_g2/TRINITY_DN9168_c0_g2/TRINITY_DN2869_c2_g1	6
GO:0045880	positive regulation of smoothened signaling pathway	10/2626	39/33142	0.000719	0.006465	0.004841	TRINITY_DN7740_c0_g1/TRINITY_DN35198_c0_g1/TRINITY_DN18321_c0_g1/TRINITY_DN722_c1_g1/TRINITY_DN4002_c0_g3/TRINITY_DN5483_c0_g1/TRINITY_DN9633_c0_g1/TRINITY_DN6230_c4_g1/TRINITY_DN4002_c0_g1/TRINITY_DN59259_c0_g1	10
GO:0005549	odorant binding	12/2626	53/33142	0.000743	0.006634	0.004967	TRINITY_DN83616_c0_g1/TRINITY_DN34526_c0_g1/TRINITY_DN10273_c0_g1/TRINITY_DN10142_c0_g3/TRINITY_DN11012_c0_g1/TRINITY_DN26479_c0_g1/TRINITY_DN9973	12

							_c0_g1/TRINITY_DN27088_c0_g3/TRINITY_DN64506_c0_g1/TRINITY_DN13749_c0_g1/TRINITY_DN24670_c0_g1/TRINITY_DN16300_c0_g1	
GO:0007626	locomotory behavior	12/2626	53/33142	0.000743	0.006634	0.004967	TRINITY_DN17778_c0_g1/TRINITY_DN6174_c0_g1/TRINITY_DN20653_c0_g1/TRINITY_DN1580_c0_g1/TRINITY_DN7759_c0_g1/TRINITY_DN22381_c0_g1/TRINITY_DN1267_c0_g2/TRINITY_DN8217_c1_g1/TRINITY_DN31435_c0_g1/TRINITY_DN8346_c1_g1/TRINITY_DN18131_c0_g1/TRINITY_DN6839_c2_g1	12
GO:0005261	cation channel activity	7/2626	21/33142	0.000837	0.00745	0.005578	TRINITY_DN6597_c0_g1/TRINITY_DN1570_c0_g1/TRINITY_DN8167_c0_g1/TRINITY_DN10654_c0_g1/TRINITY_DN28241_c0_g1/TRINITY_DN35025_c0_g1/TRINITY_DN4522_c0_g1	7
GO:0016616	oxidoreductase activity, acting on the CH-OH group of donors, NAD or NADP as acceptor	22/2626	134/33142	0.000849	0.007537	0.005643	TRINITY_DN2997_c0_g1/TRINITY_DN8066_c0_g1/TRINITY_DN19075_c0_g1/TRINITY_DN11631_c0_g3/TRINITY_DN1929_c1_g1/TRINITY_DN84_c2_g1/TRINITY_DN7625_c0_g1/TRINITY_DN1636_c0_g1/TRINITY_DN10137_c0_g1/TRINITY_DN3305_c0_g1/TRINITY_	22

							DN8561_c0_g2/TRINITY_DN2398_c0_g2/TRINITY_DN17415_c0_g1/TRINITY_DN1610_c0_g1/TRINITY_DN6810_c0_g1/TRINITY_DN7264_c0_g1/TRINITY_DN11473_c0_g1/TRINITY_DN3559_c0_g1/TRINITY_DN37668_c0_g1/TRINITY_DN7072_c1_g2/TRINITY_DN692_c2_g1/TRINITY_DN6300_c0_g1	
GO:0008285	negative regulation of cell population proliferation	20/2626	117/33142	0.000854	0.007555	0.005657	TRINITY_DN219_c3_g1/TRINITY_DN1990_c0_g1/TRINITY_DN6886_c3_g1/TRINITY_DN5781_c0_g1/TRINITY_DN12891_c0_g1/TRINITY_DN1178_c2_g1/TRINITY_DN10145_c1_g1/TRINITY_DN11156_c0_g5/TRINITY_DN93_c0_g1/TRINITY_DN22905_c0_g1/TRINITY_DN9158_c3_g1/TRINITY_DN4000_c0_g1/TRINITY_DN709_c0_g1/TRINITY_DN2011_c0_g1/TRINITY_DN12650_c0_g1/TRINITY_DN12338_c0_g1/TRINITY_DN7084_c0_g1/TRINITY_DN3545_c0_g1/TRINITY_DN2285_c0_g1/TRINITY_DN7471_c0_g1	20
GO:0004602	glutathione peroxidase activity	8/2626	27/33142	0.000867	0.007625	0.005709	TRINITY_DN7384_c0_g1/TRINITY_DN4836_c0_g1/TRINITY_DN7756_c0_g1/TRINITY_DN42262_c0_g1/TRINITY_DN1	8

							3964_c0_g1/TRINITY_DN1118_c0_g1/TRINITY_DN13697_c0_g1/TRINITY_DN8671_c0_g1	
GO:0035002	liquid clearance, open tracheal system	8/2626	27/33142	0.000867	0.007625	0.005709	TRINITY_DN11631_c0_g3/TRINITY_DN10841_c0_g1/TRINITY_DN3305_c0_g1/TRINITY_DN6810_c0_g1/TRINITY_DN10545_c1_g1/TRINITY_DN19234_c0_g1/TRINITY_DN3178_c0_g1/TRINITY_DN18007_c0_g1	8
GO:0016298	lipase activity	12/2626	54/33142	0.000887	0.007774	0.005821	TRINITY_DN6455_c0_g1/TRINITY_DN77269_c0_g1/TRINITY_DN5164_c0_g3/TRINITY_DN1568_c0_g1/TRINITY_DN21814_c0_g2/TRINITY_DN2675_c0_g1/TRINITY_DN23263_c0_g1/TRINITY_DN4485_c0_g1/TRINITY_DN4211_c1_g3/TRINITY_DN5833_c0_g1/TRINITY_DN4537_c0_g1/TRINITY_DN3989_c1_g1	12
GO:0005743	mitochondrial inner membrane	51/2626	410/33142	0.000931	0.008136	0.006092	TRINITY_DN6661_c0_g1/TRINITY_DN4992_c0_g1/TRINITY_DN3778_c2_g1/TRINITY_DN7384_c0_g1/TRINITY_DN59294_c0_g1/TRINITY_DN15486_c0_g2/TRINITY_DN17581_c0_g1/TRINITY_DN51872_c0_g1/TRINITY_DN52390_c0_g1/TRINITY_DN3655_c4_g1/TRINITY_DN5690_c0_g1/TRINITY_DN42262_c0_g1/TRINITY_DN1	51

4803_c0_g1/TRINITY_DN1283
5_c0_g1/TRINITY_DN4818_c0
_g1/TRINITY_DN7737_c0_g1/
TRINITY_DN3983_c0_g1/TRI
NITY_DN365_c5_g1/TRINITY
_DN10159_c1_g1/TRINITY_D
N7813_c0_g1/TRINITY_DN51
99_c0_g1/TRINITY_DN2899_c
0_g1/TRINITY_DN10873_c0_g
1/TRINITY_DN12540_c0_g1/T
RINITY_DN8476_c0_g2/TRIN
ITY_DN96301_c0_g1/TRINITY
_DN21082_c0_g1/TRINITY_D
N9845_c0_g2/TRINITY_DN38
01_c0_g1/TRINITY_DN6638_c
2_g1/TRINITY_DN7958_c0_g1
/TRINITY_DN20844_c0_g1/TR
INITY_DN4859_c0_g1/TRINIT
Y_DN20346_c0_g1/TRINITY_
DN49166_c0_g1/TRINITY_DN
1118_c0_g1/TRINITY_DN1188
6_c0_g1/TRINITY_DN15536_c
0_g1/TRINITY_DN31840_c0_g
1/TRINITY_DN6690_c1_g1/TR
INITY_DN7252_c0_g2/TRINIT
Y_DN16317_c0_g1/TRINITY_
DN6254_c0_g1/TRINITY_DN4
978_c0_g1/TRINITY_DN6225_
c0_g1/TRINITY_DN1436_c1_g
1/TRINITY_DN8291_c0_g1/TR
INITY_DN7875_c0_g1/TRINIT
Y_DN10344_c0_g1/TRINITY_

insects MDPI

							DN7264_c0_g3/TRINITY_DN940_c0_g1	
GO:0017016	Ras GTPase binding	5/2626	11/33142	0.000957	0.008187	0.00613	TRINITY_DN22690_c0_g1/TRINITY_DN8297_c0_g1/TRINITY_DN3024_c0_g1/TRINITY_DN24893_c0_g1/TRINITY_DN12127_c0_g1	5
GO:0034383	low-density lipoprotein particle clearance	5/2626	11/33142	0.000957	0.008187	0.00613	TRINITY_DN1568_c0_g1/TRINITY_DN5364_c0_g1/TRINITY_DN5086_c0_g1/TRINITY_DN333_c0_g1/TRINITY_DN8292_c0_g1	5
GO:0035006	melanization defense response	5/2626	11/33142	0.000957	0.008187	0.00613	TRINITY_DN10890_c0_g1/TRINITY_DN12136_c0_g2/TRINITY_DN10159_c1_g1/TRINITY_DN3717_c0_g1/TRINITY_DN22425_c0_g1	5
GO:0035725	sodium ion transmembrane transport	5/2626	11/33142	0.000957	0.008187	0.00613	TRINITY_DN10654_c0_g1/TRINITY_DN17398_c0_g2/TRINITY_DN8644_c0_g1/TRINITY_DN2003_c0_g1/TRINITY_DN18471_c0_g2	5
GO:0035735	intraciliary transport involved in cilium assembly	5/2626	11/33142	0.000957	0.008187	0.00613	TRINITY_DN19269_c0_g1/TRINITY_DN9930_c0_g2/TRINITY_DN11340_c0_g1/TRINITY_DN12886_c0_g1/TRINITY_DN9168_c0_g2	5
GO:0045504	dynein heavy chain binding	5/2626	11/33142	0.000957	0.008187	0.00613	TRINITY_DN12886_c0_g1/TRINITY_DN15113_c0_g1/TRINITY_DN2372_c0_g1/TRINITY_DN5968_c0_g1/TRINITY_DN14798_c0_g1	5

GO:0050729	positive regulation of inflammatory response	5/2626	11/33142	0.000957	0.008187	0.00613	TRINITY_DN3991_c0_g1/TRINITY_DN6931_c0_g1/TRINITY_DN2682_c0_g1/TRINITY_DN56782_c0_g1/TRINITY_DN10344_c0_g1	5
GO:0005796	Golgi lumen	6/2626	16/33142	0.000982	0.008301	0.006215	TRINITY_DN2738_c0_g1/TRINITY_DN12529_c0_g1/TRINITY_DN740_c0_g1/TRINITY_DN1902_c0_g1/TRINITY_DN2598_c0_g2/TRINITY_DN9853_c0_g1	6
GO:0007369	gastrulation	6/2626	16/33142	0.000982	0.008301	0.006215	TRINITY_DN15240_c0_g1/TRINITY_DN39639_c3_g1/TRINITY_DN15411_c0_g1/TRINITY_DN9642_c1_g2/TRINITY_DN645_c0_g1/TRINITY_DN864_c0_g3	6
GO:0071260	cellular response to mechanical stimulus	6/2626	16/33142	0.000982	0.008301	0.006215	TRINITY_DN8167_c0_g1/TRINITY_DN7743_c0_g1/TRINITY_DN1797_c0_g1/TRINITY_DN3238_c0_g1/TRINITY_DN10344_c0_g1/TRINITY_DN4895_c1_g1	6
GO:0097546	ciliary base	6/2626	16/33142	0.000982	0.008301	0.006215	TRINITY_DN5006_c0_g2/TRINITY_DN11340_c0_g1/TRINITY_DN13406_c0_g1/TRINITY_DN5875_c0_g1/TRINITY_DN306_c1_g2/TRINITY_DN9168_c0_g2	6
GO:0030308	negative regulation of cell growth	9/2626	34/33142	0.001028	0.008613	0.006449	TRINITY_DN14358_c0_g1/TRINITY_DN1487_c0_g2/TRINITY_DN4329_c1_g1/TRINITY_D	9

							N1178_c2_g1/TRINITY_DN2095_c0_g1/TRINITY_DN10569_c0_g2/TRINITY_DN1653_c0_g1/TRINITY_DN22883_c0_g1/TRINITY_DN16713_c0_g1	
GO:0030336	negative regulation of cell migration	9/2626	34/33142	0.001028	0.008613	0.006449	TRINITY_DN1313_c0_g2/TRINITY_DN10760_c0_g1/TRINITY_DN56782_c0_g1/TRINITY_DN22905_c0_g1/TRINITY_DN9158_c3_g1/TRINITY_DN12127_c0_g1/TRINITY_DN51821_c0_g1/TRINITY_DN709_c0_g1/TRINITY_DN7084_c0_g1	9
GO:0071222	cellular response to lipopolysaccharide	9/2626	34/33142	0.001028	0.008613	0.006449	TRINITY_DN3120_c0_g1/TRINITY_DN7048_c0_g1/TRINITY_DN8167_c0_g1/TRINITY_DN56782_c0_g1/TRINITY_DN40503_c0_g1/TRINITY_DN4326_c0_g1/TRINITY_DN4137_c2_g1/TRINITY_DN5788_c1_g1/TRINITY_DN14134_c0_g1	9
GO:0007218	neuropeptide signaling pathway	12/2626	55/33142	0.001053	0.008773	0.006569	TRINITY_DN5910_c0_g1/TRINITY_DN36816_c0_g1/TRINITY_DN14157_c0_g1/TRINITY_DN7577_c0_g1/TRINITY_DN965_c0_g1/TRINITY_DN13011_c0_g1/TRINITY_DN5678_c0_g1/TRINITY_DN58254_c0_g1/TRINITY_DN3032_c1_g2/TRINITY_DN33745_c0_g1/TRINITY_DN15502_c0_g1/TRINITY_DN4000_c0_g2	12

GO:0030027	lamellipodium	12/2626	55/33142	0.001053	0.008773	0.006569	TRINITY_DN3327_c0_g1/TRINITY_DN4329_c1_g1/TRINITY_DN10760_c0_g1/TRINITY_DN3363_c0_g1/TRINITY_DN93_c0_g1/TRINITY_DN5362_c0_g1/TRINITY_DN273_c1_g1/TRINITY_DN2094_c0_g1/TRINITY_DN39318_c0_g1/TRINITY_DN1908_c0_g1/TRINITY_DN13875_c0_g1/TRINITY_DN4487_c3_g2	12
GO:0051015	actin filament binding	20/2626	119/33142	0.001061	0.008813	0.006599	TRINITY_DN6440_c0_g1/TRINITY_DN725_c5_g1/TRINITY_DN9277_c0_g1/TRINITY_DN9358_c0_g1/TRINITY_DN3740_c1_g1/TRINITY_DN1691_c0_g2/TRINITY_DN1908_c3_g1/TRINITY_DN47_c0_g1/TRINITY_DN4329_c1_g1/TRINITY_DN4441_c3_g1/TRINITY_DN5521_c0_g1/TRINITY_DN130_c0_g1/TRINITY_DN9935_c1_g1/TRINITY_DN5362_c0_g1/TRINITY_DN12127_c0_g1/TRINITY_DN2869_c2_g1/TRINITY_DN1186_c0_g1/TRINITY_DN2347_c1_g1/TRINITY_DN7420_c0_g1/TRINITY_DN6505_c0_g1	20
GO:0031625	ubiquitin protein ligase binding	28/2626	190/33142	0.001074	0.008892	0.006658	TRINITY_DN1936_c0_g1/TRINITY_DN21399_c0_g2/TRINITY_DN6834_c3_g1/TRINITY_	28

							DN8213_c0_g1/TRINITY_DN16674_c1_g3/TRINITY_DN16674_c1_g2/TRINITY_DN954_c0_g1/TRINITY_DN20852_c0_g1/TRINITY_DN35198_c0_g1/TRINITY_DN36153_c0_g3/TRINITY_DN578_c1_g1/TRINITY_DN4644_c0_g1/TRINITY_DN6485_c0_g1/TRINITY_DN3983_c0_g1/TRINITY_DN233_c0_g1/TRINITY_DN7813_c0_g1/TRINITY_DN755_c1_g1/TRINITY_DN1765_c2_g1/TRINITY_DN3816_c0_g1/TRINITY_DN9935_c1_g1/TRINITY_DN9041_c0_g1/TRINITY_DN11156_c0_g5/TRINITY_DN2331_c0_g1/TRINITY_DN40503_c0_g1/TRINITY_DN3262_c0_g1/TRINITY_DN7200_c0_g2/TRINITY_DN9041_c0_g2/TRINITY_DN8343_c0_g1	
GO:0004314	[acyl-carrier-protein] S-malonyltransferase activity	10/2626	41/33142	0.001096	0.00897	0.006716	TRINITY_DN606_c3_g1/TRINITY_DN19001_c0_g1/TRINITY_DN23921_c0_g1/TRINITY_DN482_c1_g1/TRINITY_DN29856_c0_g1/TRINITY_DN1549_c0_g1/TRINITY_DN40189_c0_g1/TRINITY_DN484_c0_g1/TRINITY_DN244_c3_g1/TRINITY_DN61025_c0_g1	10

insects MDPI

GO:0007596	blood coagulation	10/2626	41/33142	0.001096	0.00897	0.006716	TRINITY_DN2738_c0_g1/TRINITY_DN5848_c0_g1/TRINITY_DN11048_c0_g1/TRINITY_DN23953_c0_g1/TRINITY_DN9755_c0_g1/TRINITY_DN7955_c1_g1/TRINITY_DN39494_c0_g1/TRINITY_DN2734_c0_g1/TRINITY_DN9853_c0_g1/TRINITY_DN9123_c0_g1	10
GO:0007602	phototransduction	10/2626	41/33142	0.001096	0.00897	0.006716	TRINITY_DN1748_c0_g1/TRINITY_DN5198_c0_g1/TRINITY_DN20958_c0_g1/TRINITY_DN41433_c0_g1/TRINITY_DN1229_c2_g1/TRINITY_DN20958_c0_g2/TRINITY_DN6805_c0_g1/TRINITY_DN33474_c0_g1/TRINITY_DN2094_c0_g1/TRINITY_DN4784_c1_g1	10
GO:0090630	activation of GTPase activity	10/2626	41/33142	0.001096	0.00897	0.006716	TRINITY_DN4223_c1_g1/TRINITY_DN2087_c0_g1/TRINITY_DN3614_c1_g1/TRINITY_DN9935_c1_g1/TRINITY_DN18317_c0_g1/TRINITY_DN10575_c2_g1/TRINITY_DN5362_c0_g1/TRINITY_DN8911_c1_g2/TRINITY_DN13875_c0_g1/TRINITY_DN4816_c0_g2	10
GO:0001523	retinoid metabolic process	8/2626	28/33142	0.00113	0.009117	0.006826	TRINITY_DN7437_c0_g1/TRINITY_DN8493_c0_g1/TRINITY_DN19075_c0_g1/TRINITY_DN2492_c0_g1/TRINITY_DN2009_c0_g1/TRINITY_DN5121_	8

insects MDPI

							c0_g1/TRINITY_DN77404_c0_g1/TRINITY_DN56436_c0_g1	
GO:0004620	phospholipase activity	8/2626	28/33142	0.00113	0.009117	0.006826	TRINITY_DN1568_c0_g2/TRINITY_DN4813_c0_g1/TRINITY_DN2848_c1_g1/TRINITY_DN2675_c0_g1/TRINITY_DN4211_c1_g3/TRINITY_DN18499_c0_g1/TRINITY_DN17391_c0_g1/TRINITY_DN26376_c0_g1	8
GO:0019534	toxin transmembrane transporter activity	8/2626	28/33142	0.00113	0.009117	0.006826	TRINITY_DN2006_c0_g1/TRINITY_DN1478_c1_g3/TRINITY_DN14468_c0_g1/TRINITY_DN10757_c0_g1/TRINITY_DN10798_c0_g1/TRINITY_DN11056_c1_g1/TRINITY_DN11061_c0_g2/TRINITY_DN38748_c0_g1	8
GO:0032355	response to estradiol	8/2626	28/33142	0.00113	0.009117	0.006826	TRINITY_DN1557_c0_g1/TRINITY_DN3007_c0_g1/TRINITY_DN2779_c1_g2/TRINITY_DN10569_c0_g2/TRINITY_DN2779_c1_g3/TRINITY_DN9853_c0_g1/TRINITY_DN4859_c0_g1/TRINITY_DN14134_c0_g1	8
GO:0080019	fatty-acyl-CoA reductase (alcohol-forming) activity	8/2626	28/33142	0.00113	0.009117	0.006826	TRINITY_DN2997_c0_g1/TRINITY_DN8066_c0_g1/TRINITY_DN57502_c0_g1/TRINITY_DN11631_c0_g3/TRINITY_DN3305_c0_g1/TRINITY_DN6810_c0_g1/TRINITY_DN1450_c0_g3/TRINITY_DN18007_c0_g1	8

GO:0008364	pupal chitin-based cuticle development	7/2626	22/33142	0.001144	0.009198	0.006887	TRINITY_DN7191_c0_g1/TRINITY_DN18146_c0_g1/TRINITY_DN9801_c0_g1/TRINITY_DN7191_c0_g2/TRINITY_DN34213_c0_g1/TRINITY_DN28886_c0_g1/TRINITY_DN16873_c0_g1	7
GO:0048749	compound eye development	15/2626	79/33142	0.001231	0.00987	0.00739	TRINITY_DN3245_c0_g1/TRINITY_DN17739_c0_g2/TRINITY_DN12504_c0_g1/TRINITY_DN18321_c0_g1/TRINITY_DN2241_c0_g2/TRINITY_DN15411_c0_g1/TRINITY_DN9245_c0_g1/TRINITY_DN5091_c0_g1/TRINITY_DN4517_c0_g2/TRINITY_DN3636_c0_g3/TRINITY_DN3636_c0_g4/TRINITY_DN1941_c0_g1/TRINITY_DN61_c0_g1/TRINITY_DN3093_c0_g1/TRINITY_DN17100_c0_g1	15
GO:0042391	regulation of membrane potential	12/2626	56/33142	0.001245	0.009932	0.007437	TRINITY_DN21399_c0_g2/TRINITY_DN30262_c0_g1/TRINITY_DN8355_c0_g1/TRINITY_DN16407_c1_g1/TRINITY_DN4958_c0_g1/TRINITY_DN16593_c0_g1/TRINITY_DN3816_c0_g1/TRINITY_DN13726_c0_g1/TRINITY_DN13011_c0_g1/TRINITY_DN2938_c3_g1/TRINITY_DN7200_c0_g2/TRINITY_DN4000_c0_g2	12

GO:0071805	potassium ion transmembrane transport	12/2626	56/33142	0.001245	0.009932	0.007437	TRINITY_DN10654_c0_g1/TRINITY_DN6323_c1_g1/TRINITY_DN12107_c0_g1/TRINITY_DN16407_c1_g1/TRINITY_DN15030_c0_g2/TRINITY_DN16593_c0_g1/TRINITY_DN13726_c0_g1/TRINITY_DN14953_c0_g3/TRINITY_DN14377_c0_g1/TRINITY_DN30782_c0_g2/TRINITY_DN4021_c0_g2/TRINITY_DN7318_c0_g1	12
GO:0003007	heart morphogenesis	9/2626	35/33142	0.001288	0.01024	0.007667	TRINITY_DN77781_c0_g1/TRINITY_DN1691_c0_g2/TRINITY_DN1908_c3_g1/TRINITY_DN60483_c1_g1/TRINITY_DN85_c0_g1/TRINITY_DN13564_c0_g1/TRINITY_DN16751_c0_g1/TRINITY_DN3636_c0_g3/TRINITY_DN3636_c0_g4	9
GO:0005938	cell cortex	24/2626	156/33142	0.001298	0.010293	0.007707	TRINITY_DN5310_c0_g2/TRINITY_DN6305_c1_g1/TRINITY_DN47_c0_g1/TRINITY_DN1120_c0_g2/TRINITY_DN5049_c0_g1/TRINITY_DN8217_c0_g1/TRINITY_DN6222_c0_g1/TRINITY_DN16954_c0_g1/TRINITY_DN3024_c0_g1/TRINITY_DN3400_c0_g1/TRINITY_DN2868_c0_g1/TRINITY_DN6081_c1_g2/TRINITY_DN377_c0_g1/TRINITY_DN3058_c0_g1/TRINITY_DN11154_c0_g1/TRI	24

							NITY_DN8504_c0_g1/TRINITY_DN31678_c0_g1/TRINITY_DN898_c0_g1/TRINITY_DN2869_c2_g1/TRINITY_DN3459_c0_g1/TRINITY_DN8181_c0_g1/TRINITY_DN342_c0_g1/TRINITY_DN2347_c1_g1/TRINITY_DN6505_c0_g1	
GO:0004181	metallocarboxypeptidase activity	10/2626	42/33142	0.001338	0.010574	0.007917	TRINITY_DN5956_c5_g1/TRINITY_DN2051_c0_g1/TRINITY_DN1919_c0_g1/TRINITY_DN5912_c0_g1/TRINITY_DN1773_c1_g2/TRINITY_DN4270_c0_g2/TRINITY_DN15913_c0_g1/TRINITY_DN4541_c0_g1/TRINITY_DN11117_c1_g1/TRINITY_DN579_c0_g1	10
GO:0043547	positive regulation of GTPase activity	13/2626	64/33142	0.001341	0.010574	0.007917	TRINITY_DN11355_c0_g3/TRINITY_DN22690_c0_g1/TRINITY_DN2738_c0_g1/TRINITY_DN8297_c0_g1/TRINITY_DN740_c0_g1/TRINITY_DN2598_c0_g2/TRINITY_DN24893_c0_g1/TRINITY_DN6093_c0_g2/TRINITY_DN12127_c0_g1/TRINITY_DN6910_c0_g1/TRINITY_DN9123_c0_g1/TRINITY_DN2078_c0_g2/TRINITY_DN4816_c0_g2	13
GO:0048471	perinuclear region of cytoplasm	42/2626	327/33142	0.001354	0.010641	0.007967	TRINITY_DN658_c0_g1/TRINITY_DN8299_c0_g1/TRINITY_DN3327_c0_g1/TRINITY_DN7	42

insects

048_c0_g1/TRINITY_DN7743_
c0_g1/TRINITY_DN2476_c1_g
1/TRINITY_DN338_c0_g2/TRI
NITY_DN5278_c0_g1/TRINIT
Y_DN200_c0_g2/TRINITY_D
N5173_c0_g1/TRINITY_DN54
44_c0_g1/TRINITY_DN17663_
c0_g1/TRINITY_DN6222_c0_g
1/TRINITY_DN578_c1_g1/TRI
NITY_DN4644_c0_g1/TRINIT
Y_DN233_c0_g1/TRINITY_D
N755_c1_g1/TRINITY_DN200
88_c0_g1/TRINITY_DN271_c0
_g1/TRINITY_DN3665_c0_g1/
TRINITY_DN9175_c0_g1/TRI
NITY_DN93_c0_g1/TRINITY_
DN5276_c1_g1/TRINITY_DN2
165_c3_g1/TRINITY_DN22905
_c0_g1/TRINITY_DN898_c0_g
1/TRINITY_DN2869_c2_g1/TR
INITY_DN26935_c0_g1/TRINI
TY_DN4326_c0_g1/TRINITY_
DN273_c1_g1/TRINITY_DN66
43_c0_g1/TRINITY_DN15445_
c0_g1/TRINITY_DN25627_c0_
g1/TRINITY_DN9055_c0_g2/T
RINITY_DN7084_c0_g1/TRIN
ITY_DN10344_c0_g1/TRINITY
_DN6505_c0_g1/TRINITY_DN
26727_c0_g1/TRINITY_DN111
160_c0_g1/TRINITY_DN7471_

							c0_g1/TRINITY_DN4416_c0_g1/TRINITY_DN18471_c0_g2	
GO:0010629	negative regulation of gene expression	17/2626	96/33142	0.001357	0.010641	0.007967	TRINITY_DN3991_c0_g1/TRINITY_DN2578_c0_g1/TRINITY_DN32305_c0_g1/TRINITY_DN1893_c0_g1/TRINITY_DN4783_c0_g1/TRINITY_DN10890_c0_g1/TRINITY_DN3120_c0_g1/TRINITY_DN1990_c0_g1/TRINITY_DN1695_c0_g1/TRINITY_DN5049_c0_g1/TRINITY_DN5781_c0_g1/TRINITY_DN2682_c0_g1/TRINITY_DN9532_c0_g1/TRINITY_DN10569_c0_g2/TRINITY_DN9158_c3_g1/TRINITY_DN709_c0_g1/TRINITY_DN56840_c0_g1	17
GO:0032391	photoreceptor connecting cilium	6/2626	17/33142	0.001417	0.011077	0.008294	TRINITY_DN6327_c1_g1/TRINITY_DN47_c0_g1/TRINITY_DN3762_c0_g1/TRINITY_DN11340_c0_g1/TRINITY_DN306_c1_g2/TRINITY_DN2746_c0_g1	6
GO:0016705	oxidoreductase activity, acting on paired donors, with incorporation or reduction of molecular oxygen	32/2626	231/33142	0.001428	0.011136	0.008338	TRINITY_DN788_c0_g2/TRINITY_DN12459_c0_g1/TRINITY_DN510_c0_g1/TRINITY_DN14155_c0_g1/TRINITY_DN8337_c0_g3/TRINITY_DN14638_c0_g1/TRINITY_DN2034_c0_g1/TRINITY_DN9929_c0_g1/TRINITY_DN80619_c0_g2/TRINITY_DN11949_c0_g2/TRINITY	32

GO:0016055	Wnt signaling pathway	20/2626	122/33142	0.001453	0.011211	0.008394	_DN10323_c0_g1/TRINITY_DN9323_c0_g2/TRINITY_DN15512_c0_g1/TRINITY_DN6729_c0_g2/TRINITY_DN6729_c0_g3/TRINITY_DN3648_c0_g6/TRINITY_DN8718_c0_g1/TRINITY_DN6010_c3_g1/TRINITY_DN7771_c0_g2/TRINITY_DN996_c0_g1/TRINITY_DN5213_c0_g1/TRINITY_DN25933_c0_g2/TRINITY_DN33241_c0_g1/TRINITY_DN10120_c0_g1/TRINITY_DN8705_c0_g1/TRINITY_DN21490_c0_g1/TRINITY_DN10422_c0_g1/TRINITY_DN3753_c0_g1/TRINITY_DN5625_c1_g1/TRINITY_DN8029_c0_g2/TRINITY_DN7224_c1_g3/TRINITY_DN25846_c0_g2 TRINITY_DN34083_c0_g1/TRINITY_DN60341_c0_g1/TRINITY_DN7425_c6_g1/TRINITY_DN32249_c0_g1/TRINITY_DN383_c0_g1/TRINITY_DN2786_c0_g1/TRINITY_DN46257_c0_g1/TRINITY_DN806_c0_g2/TRINITY_DN9233_c0_g1/TRINITY_DN75_c2_g2/TRINITY_DN1406_c0_g1/TRINITY_DN5157_c0_g2/TRINITY_DN22905_c0_g1/TRINITY_DN77356_c0_g1/TRINITY_DN2869_c2_g1/TRI	20

							NITY_DN6230_c4_g1/TRINITY_DN806_c0_g1/TRINITY_DN35596_c0_g1/TRINITY_DN6541_c1_g2/TRINITY_DN1084_c0_g1	
GO:0007613	memory	8/2626	29/33142	0.001454	0.011211	0.008394	TRINITY_DN9170_c1_g1/TRINITY_DN6515_c0_g1/TRINITY_DN2095_c0_g1/TRINITY_DN7045_c1_g1/TRINITY_DN428_c0_g1/TRINITY_DN2961_c0_g1/TRINITY_DN6409_c3_g1/TRINITY_DN18471_c0_g2	8
GO:0036158	outer dynein arm assembly	8/2626	29/33142	0.001454	0.011211	0.008394	TRINITY_DN6657_c0_g1/TRINITY_DN9451_c0_g1/TRINITY_DN20881_c0_g2/TRINITY_DN18134_c0_g1/TRINITY_DN5914_c0_g1/TRINITY_DN2372_c0_g1/TRINITY_DN5968_c0_g1/TRINITY_DN3299_c0_g1	8
GO:0046693	sperm storage	8/2626	29/33142	0.001454	0.011211	0.008394	TRINITY_DN7191_c0_g1/TRINITY_DN18146_c0_g1/TRINITY_DN9801_c0_g1/TRINITY_DN7191_c0_g2/TRINITY_DN638_c1_g1/TRINITY_DN34213_c0_g1/TRINITY_DN28886_c0_g1/TRINITY_DN16873_c0_g1	8
GO:0005272	sodium channel activity	7/2626	23/33142	0.001532	0.011306	0.008465	TRINITY_DN10654_c0_g1/TRINITY_DN10841_c0_g1/TRINITY_DN17412_c0_g1/TRINITY_DN10545_c1_g1/TRINITY_DN12721_c0_g1/TRINITY_DN2	7

							003_c0_g1/TRINITY_DN19234_c0_g1		
GO:0050680	negative regulation of epithelial cell proliferation	7/2626	23/33142	0.001532	0.011306	0.008465	TRINITY_DN6327_c1_g1/TRINITY_DN19269_c0_g1/TRINITY_DN4002_c0_g3/TRINITY_DN11340_c0_g1/TRINITY_DN306_c1_g2/TRINITY_DN5362_c0_g1/TRINITY_DN4002_c0_g1	7
GO:0031088	platelet dense granule membrane	5/2626	12/33142	0.001534	0.011306	0.008465	TRINITY_DN406_c0_g3/TRINITY_DN5049_c0_g1/TRINITY_DN2999_c0_g1/TRINITY_DN406_c0_g2/TRINITY_DN42565_c0_g1	5
GO:0035973	aggrephagy	5/2626	12/33142	0.001534	0.011306	0.008465	TRINITY_DN16674_c1_g3/TRINITY_DN16674_c1_g2/TRINITY_DN4811_c0_g1/TRINITY_DN36153_c0_g3/TRINITY_DN1201_c3_g1	5
GO:0040018	positive regulation of multicellular organism growth	5/2626	12/33142	0.001534	0.011306	0.008465	TRINITY_DN19520_c0_g1/TRINITY_DN669_c0_g1/TRINITY_DN338_c0_g1/TRINITY_DN8729_c1_g1/TRINITY_DN14134_c0_g1	5
GO:0045433	male courtship behavior, veined wing generated song production	5/2626	12/33142	0.001534	0.011306	0.008465	TRINITY_DN1748_c0_g1/TRINITY_DN32284_c0_g2/TRINITY_DN23750_c0_g1/TRINITY_DN2003_c0_g1/TRINITY_DN20249_c0_g1	5
GO:0045861	negative regulation of proteolysis	5/2626	12/33142	0.001534	0.011306	0.008465	TRINITY_DN16500_c1_g1/TRINITY_DN3123_c0_g1/TRINITY_DN233_c0_g1/TRINITY_D	5

							N3717_c0_g1/TRINITY_DN14093_c0_g1	
GO:0048286	lung alveolus development	5/2626	12/33142	0.001534	0.011306	0.008465	TRINITY_DN3991_c0_g1/TRINITY_DN7048_c0_g1/TRINITY_DN2682_c0_g1/TRINITY_DN13138_c0_g1/TRINITY_DN2095_c0_g1	5
GO:0060076	excitatory synapse	5/2626	12/33142	0.001534	0.011306	0.008465	TRINITY_DN9375_c0_g1/TRINITY_DN27259_c0_g1/TRINITY_DN1765_c2_g1/TRINITY_DN7687_c0_g1/TRINITY_DN4674_c0_g1	5
GO:0102485	dATP phosphohydrolase activity	5/2626	12/33142	0.001534	0.011306	0.008465	TRINITY_DN24800_c0_g1/TRINITY_DN434_c0_g1/TRINITY_DN595_c0_g3/TRINITY_DN9430_c1_g1/TRINITY_DN153517_c0_g1	5
GO:0102486	dCTP phosphohydrolase activity	5/2626	12/33142	0.001534	0.011306	0.008465	TRINITY_DN24800_c0_g1/TRINITY_DN434_c0_g1/TRINITY_DN595_c0_g3/TRINITY_DN9430_c1_g1/TRINITY_DN153517_c0_g1	5
GO:0102487	dUTP phosphohydrolase activity	5/2626	12/33142	0.001534	0.011306	0.008465	TRINITY_DN24800_c0_g1/TRINITY_DN434_c0_g1/TRINITY_DN595_c0_g3/TRINITY_DN9430_c1_g1/TRINITY_DN153517_c0_g1	5
GO:0102488	dTTP phosphohydrolase activity	5/2626	12/33142	0.001534	0.011306	0.008465	TRINITY_DN24800_c0_g1/TRINITY_DN434_c0_g1/TRINITY_DN595_c0_g3/TRINITY_DN9430_c1_g1/TRINITY_DN153517_c0_g1	5

GO:0102489	GTP phosphohydrolase activity	5/2626	12/33142	0.001534	0.011306	0.008465	TRINITY_DN24800_c0_g1/TRINITY_DN434_c0_g1/TRINITY_DN595_c0_g3/TRINITY_DN9430_c1_g1/TRINITY_DN153517_c0_g1	5
GO:0102490	8-oxo-dGTP phosphohydrolase activity	5/2626	12/33142	0.001534	0.011306	0.008465	TRINITY_DN24800_c0_g1/TRINITY_DN434_c0_g1/TRINITY_DN595_c0_g3/TRINITY_DN9430_c1_g1/TRINITY_DN153517_c0_g1	5
GO:0102491	dGTP phosphohydrolase activity	5/2626	12/33142	0.001534	0.011306	0.008465	TRINITY_DN24800_c0_g1/TRINITY_DN434_c0_g1/TRINITY_DN595_c0_g3/TRINITY_DN9430_c1_g1/TRINITY_DN153517_c0_g1	5
GO:1902667	regulation of axon guidance	5/2626	12/33142	0.001534	0.011306	0.008465	TRINITY_DN16600_c0_g1/TRINITY_DN2738_c0_g1/TRINITY_DN740_c0_g1/TRINITY_DN2598_c0_g2/TRINITY_DN9082_c0_g1	5
GO:0030030	cell projection organization	9/2626	36/33142	0.001598	0.011746	0.008794	TRINITY_DN47_c0_g1/TRINITY_DN7530_c0_g1/TRINITY_DN38928_c0_g2/TRINITY_DN1909_c0_g1/TRINITY_DN3767_c0_g1/TRINITY_DN2331_c0_g1/TRINITY_DN35591_c0_g1/TRINITY_DN9647_c0_g1/TRINITY_DN5578_c0_g1	9
GO:0004970	ionotropic glutamate receptor activity	10/2626	43/33142	0.001621	0.011856	0.008877	TRINITY_DN21399_c0_g2/TRINITY_DN16006_c0_g1/TRINITY_DN77056_c0_g1/TRINITY_DN8355_c0_g1/TRINITY_DN	10

							1765_c2_g1/TRINITY_DN3816_c0_g1/TRINITY_DN9041_c0_g1/TRINITY_DN15420_c0_g1/TRINITY_DN7200_c0_g2/TRINITY_DN9041_c0_g2	
GO:0051056	regulation of small GTPase mediated signal transduction	10/2626	43/33142	0.001621	0.011856	0.008877	TRINITY_DN22690_c0_g1/TRINITY_DN8297_c0_g1/TRINITY_DN873_c0_g1/TRINITY_DN13983_c0_g1/TRINITY_DN9935_c1_g1/TRINITY_DN24893_c0_g1/TRINITY_DN6093_c0_g2/TRINITY_DN5120_c0_g1/TRINITY_DN6674_c0_g1/TRINITY_DN10575_c2_g1	10
GO:0051260	protein homooligomerization	19/2626	115/33142	0.001717	0.012527	0.009379	TRINITY_DN7426_c0_g1/TRINITY_DN4544_c0_g1/TRINITY_DN5871_c1_g1/TRINITY_DN1709_c0_g1/TRINITY_DN2557_c0_g2/TRINITY_DN2161_c0_g1/TRINITY_DN10760_c0_g1/TRINITY_DN1035_c0_g3/TRINITY_DN10811_c0_g1/TRINITY_DN10569_c0_g2/TRINITY_DN1517_c0_g1/TRINITY_DN14953_c0_g3/TRINITY_DN4641_c0_g1/TRINITY_DN7687_c0_g1/TRINITY_DN24632_c0_g1/TRINITY_DN3103_c0_g2/TRINITY_DN3103_c2_g1/TRINITY_DN4000_c0_g2/TRINITY_DN2961_c0_g1	19

GO:0007010	cytoskeleton organization	13/2626	66/33142	0.0018	0.013063	0.009781	TRINITY_DN6657_c0_g1/TRINITY_DN4400_c1_g1/TRINITY_DN309_c0_g1/TRINITY_DN15487_c0_g1/TRINITY_DN2241_c0_g2/TRINITY_DN130_c0_g1/TRINITY_DN9626_c0_g2/TRINITY_DN19224_c0_g1/TRINITY_DN10575_c2_g1/TRINITY_DN2281_c0_g1/TRINITY_DN10111_c0_g1/TRINITY_DN6106_c1_g1/TRINITY_DN4487_c3_g2	13
GO:0045471	response to ethanol	13/2626	66/33142	0.0018	0.013063	0.009781	TRINITY_DN77781_c0_g1/TRINITY_DN1843_c0_g2/TRINITY_DN15486_c0_g2/TRINITY_DN60483_c1_g1/TRINITY_DN1879_c0_g1/TRINITY_DN7743_c0_g1/TRINITY_DN11422_c0_g1/TRINITY_DN3916_c0_g1/TRINITY_DN10783_c0_g1/TRINITY_DN4859_c0_g1/TRINITY_DN31730_c0_g2/TRINITY_DN12958_c0_g1/TRINITY_DN33069_c0_g1	13
GO:0035336	long-chain fatty-acyl-CoA metabolic process	8/2626	30/33142	0.001846	0.013328	0.009979	TRINITY_DN2997_c0_g1/TRINITY_DN8066_c0_g1/TRINITY_DN57502_c0_g1/TRINITY_DN11631_c0_g3/TRINITY_DN3305_c0_g1/TRINITY_DN6810_c0_g1/TRINITY_DN1450_c0_g3/TRINITY_DN18007_c0_g1	8

GO:0050830	defense response to Gram-positive bacterium	8/2626	30/33142	0.001846	0.013328	0.009979	TRINITY_DN6113_c0_g1/TRINITY_DN22536_c0_g1/TRINITY_DN9354_c0_g1/TRINITY_DN1338_c0_g1/TRINITY_DN28084_c0_g1/TRINITY_DN9798_c0_g1/TRINITY_DN2292_c2_g1/TRINITY_DN10426_c0_g1	8
GO:0030176	integral component of endoplasmic reticulum membrane	17/2626	99/33142	0.001918	0.013811	0.010341	TRINITY_DN5032_c1_g1/TRINITY_DN1854_c0_g2/TRINITY_DN180_c2_g1/TRINITY_DN2199_c2_g2/TRINITY_DN7107_c0_g1/TRINITY_DN28084_c0_g1/TRINITY_DN9175_c0_g1/TRINITY_DN16751_c0_g1/TRINITY_DN12176_c0_g1/TRINITY_DN40503_c0_g1/TRINITY_DN34926_c1_g1/TRINITY_DN9521_c0_g2/TRINITY_DN10258_c0_g1/TRINITY_DN13730_c0_g1/TRINITY_DN4137_c2_g1/TRINITY_DN4009_c0_g2/TRINITY_DN689_c0_g1	17
GO:0031410	cytoplasmic vesicle	20/2626	125/33142	0.001962	0.014077	0.01054	TRINITY_DN32305_c0_g1/TRINITY_DN16936_c0_g1/TRINITY_DN4687_c0_g1/TRINITY_DN6886_c3_g1/TRINITY_DN16674_c1_g3/TRINITY_DN5198_c0_g1/TRINITY_DN16674_c1_g2/TRINITY_DN1265_c0_g1/TRINITY_DN19269_c0_g1/TRINITY_DN8433_c1_g2/TRINITY_DN7858_c0_g1/TRINITY_D	20

							N19401_c0_g1/TRINITY_DN1252_c0_g1/TRINITY_DN1614_c0_g1/TRINITY_DN271_c0_g1/TRINITY_DN7045_c1_g1/TRINITY_DN8433_c1_g1/TRINITY_DN8911_c1_g2/TRINITY_DN6409_c3_g1/TRINITY_DN4816_c0_g2	
GO:0004714	transmembrane receptor protein tyrosine kinase activity	9/2626	37/33142	0.001965	0.014077	0.01054	TRINITY_DN2276_c0_g1/TRINITY_DN10609_c0_g1/TRINITY_DN6348_c0_g1/TRINITY_DN8625_c0_g1/TRINITY_DN339_c0_g2/TRINITY_DN9924_c0_g1/TRINITY_DN2321_c0_g1/TRINITY_DN4839_c0_g1/TRINITY_DN14663_c0_g1	9
GO:0043434	response to peptide hormone	6/2626	18/33142	0.001983	0.014103	0.010559	TRINITY_DN4861_c0_g1/TRINITY_DN1568_c0_g1/TRINITY_DN7743_c0_g1/TRINITY_DN2675_c0_g1/TRINITY_DN7326_c0_g1/TRINITY_DN33241_c0_g1	6
GO:0046928	regulation of neurotransmitter secretion	6/2626	18/33142	0.001983	0.014103	0.010559	TRINITY_DN1748_c0_g1/TRINITY_DN5470_c0_g1/TRINITY_DN627_c0_g1/TRINITY_DN11154_c0_g1/TRINITY_DN4029_c0_g1/TRINITY_DN33662_c0_g1	6
GO:0070507	regulation of microtubule cytoskeleton organization	6/2626	18/33142	0.001983	0.014103	0.010559	TRINITY_DN2738_c0_g1/TRINITY_DN13406_c0_g1/TRINITY_DN740_c0_g1/TRINITY_DN2598_c0_g2/TRINITY_DN14	6

							663_c0_g1/TRINITY_DN5922_c0_g1	
GO:0030426	growth cone	12/2626	59/33142	0.002002	0.014199	0.010631	TRINITY_DN205_c0_g1/TRINITY_DN3327_c0_g1/TRINITY_DN16186_c0_g1/TRINITY_DN1265_c0_g1/TRINITY_DN10760_c0_g1/TRINITY_DN1420_c0_g1/TRINITY_DN8217_c1_g1/TRINITY_DN3665_c0_g1/TRINITY_DN9175_c0_g1/TRINITY_DN93_c0_g1/TRINITY_DN2746_c0_g1/TRINITY_DN26727_c0_g1	12
GO:0001540	amyloid-beta binding	7/2626	24/33142	0.002017	0.014199	0.010631	TRINITY_DN5364_c0_g1/TRINITY_DN5086_c0_g1/TRINITY_DN10873_c0_g1/TRINITY_DN1765_c2_g1/TRINITY_DN10569_c0_g2/TRINITY_DN7687_c0_g1/TRINITY_DN333_c0_g1	7
GO:0005680	anaphase-promoting complex	7/2626	24/33142	0.002017	0.014199	0.010631	TRINITY_DN5463_c0_g2/TRINITY_DN2352_c0_g2/TRINITY_DN13467_c0_g1/TRINITY_DN1117_c1_g1/TRINITY_DN50069_c0_g1/TRINITY_DN7439_c0_g1/TRINITY_DN4455_c0_g1	7
GO:0048661	positive regulation of smooth muscle cell proliferation	7/2626	24/33142	0.002017	0.014199	0.010631	TRINITY_DN658_c0_g1/TRINITY_DN14379_c0_g1/TRINITY_DN406_c0_g3/TRINITY_DN5049_c0_g1/TRINITY_DN2999_	7

							c0_g1/TRINITY_DN406_c0_g2/TRINITY_DN42565_c0_g1	
GO:0015171	amino acid transmembrane transporter activity	11/2626	52/33142	0.002188	0.015367	0.011506	TRINITY_DN8323_c0_g1/TRINITY_DN3446_c0_g1/TRINITY_DN6519_c0_g1/TRINITY_DN43642_c0_g1/TRINITY_DN4139_c0_g1/TRINITY_DN13394_c0_g1/TRINITY_DN8012_c0_g1/TRINITY_DN4307_c0_g1/TRINITY_DN4384_c0_g1/TRINITY_DN3638_c0_g2/TRINITY_DN12101_c0_g2	11
GO:0015662	ATPase activity, coupled to transmembrane movement of ions, phosphorylative mechanism	5/2626	13/33142	0.002331	0.016143	0.012087	TRINITY_DN406_c0_g3/TRINITY_DN5049_c0_g1/TRINITY_DN2999_c0_g1/TRINITY_DN406_c0_g2/TRINITY_DN42565_c0_g1	5
GO:0019233	sensory perception of pain	5/2626	13/33142	0.002331	0.016143	0.012087	TRINITY_DN3991_c0_g1/TRINITY_DN9375_c0_g1/TRINITY_DN2682_c0_g1/TRINITY_DN52030_c0_g1/TRINITY_DN49433_c0_g1	5
GO:0043225	ATPase-coupled anion transmembrane transporter activity	5/2626	13/33142	0.002331	0.016143	0.012087	TRINITY_DN406_c0_g3/TRINITY_DN5049_c0_g1/TRINITY_DN2999_c0_g1/TRINITY_DN406_c0_g2/TRINITY_DN42565_c0_g1	5
GO:0097484	dendrite extension	5/2626	13/33142	0.002331	0.016143	0.012087	TRINITY_DN8323_c0_g1/TRINITY_DN3446_c0_g1/TRINITY_DN13394_c0_g1/TRINITY_DN8012_c0_g1/TRINITY_DN1178_c2_g1	5

GO:0007528	neuromuscular junction development	10/2626	45/33142	0.002333	0.016143	0.012087	TRINITY_DN21789_c0_g1/TRINITY_DN3245_c0_g1/TRINITY_DN2738_c0_g1/TRINITY_DN5470_c0_g1/TRINITY_DN8832_c1_g1/TRINITY_DN740_c0_g1/TRINITY_DN2598_c0_g2/TRINITY_DN11938_c0_g1/TRINITY_DN3103_c0_g2/TRINITY_DN3103_c2_g1	10
GO:0010976	positive regulation of neuron projection development	10/2626	45/33142	0.002333	0.016143	0.012087	TRINITY_DN5056_c0_g1/TRINITY_DN3327_c0_g1/TRINITY_DN10760_c0_g1/TRINITY_DN800_c0_g2/TRINITY_DN130_c0_g1/TRINITY_DN8278_c2_g1/TRINITY_DN3722_c0_g1/TRINITY_DN7084_c0_g1/TRINITY_DN12228_c1_g1/TRINITY_DN8692_c0_g1	10
GO:0045494	photoreceptor cell maintenance	9/2626	38/33142	0.002396	0.016537	0.012382	TRINITY_DN8493_c0_g1/TRINITY_DN6327_c1_g1/TRINITY_DN6174_c0_g1/TRINITY_DN7048_c0_g1/TRINITY_DN4043_c0_g1/TRINITY_DN1541_c0_g1/TRINITY_DN5795_c3_g1/TRINITY_DN6945_c0_g2/TRINITY_DN2094_c0_g1	9
GO:0048813	dendrite morphogenesis	18/2626	110/33142	0.002497	0.017194	0.012874	TRINITY_DN2578_c0_g1/TRINITY_DN8323_c0_g1/TRINITY_DN3446_c0_g1/TRINITY_DN3245_c0_g1/TRINITY_DN2244_c1_g1/TRINITY_DN9767_c0_g3/TRINITY_DN2133_c0_g1	18

							/TRINITY_DN13394_c0_g1/TRINITY_DN8903_c0_g1/TRINITY_DN37845_c0_g1/TRINITY_DN8012_c0_g1/TRINITY_DN2424_c0_g1/TRINITY_DN8217_c1_g1/TRINITY_DN43399_c0_g1/TRINITY_DN27045_c0_g1/TRINITY_DN446_c1_g1/TRINITY_DN3545_c0_g1/TRINITY_DN2756_c0_g1	
GO:0005546	phosphatidylinositol-4, 5-bisphosphate binding	11/2626	53/33142	0.002567	0.017631	0.013201	TRINITY_DN2629_c0_g1/TRINITY_DN2531_c0_g1/TRINITY_DN16407_c1_g1/TRINITY_DN8977_c0_g1/TRINITY_DN17565_c0_g1/TRINITY_DN1775_c0_g3/TRINITY_DN2165_c3_g1/TRINITY_DN5362_c0_g1/TRINITY_DN342_c0_g1/TRINITY_DN3890_c0_g1/TRINITY_DN6505_c0_g1	11
GO:0003707	steroid hormone receptor activity	7/2626	25/33142	0.002612	0.017896	0.013399	TRINITY_DN3217_c0_g1/TRINITY_DN1883_c0_g1/TRINITY_DN5239_c1_g2/TRINITY_DN2445_c0_g1/TRINITY_DN1541_c0_g1/TRINITY_DN12413_c0_g1/TRINITY_DN669_c0_g1	7
GO:0017124	SH3 domain binding	12/2626	61/33142	0.002689	0.018188	0.013618	TRINITY_DN7437_c0_g1/TRINITY_DN5629_c0_g1/TRINITY_DN6108_c0_g1/TRINITY_DN10760_c0_g1/TRINITY_DN9843_c0_g1/TRINITY_DN4326_c0_g1/TRINITY_DN2078_c0_g	12

							2/TRINITY_DN14709_c0_g1/TRINITY_DN428_c0_g1/TRINITY_DN9107_c0_g1/TRINITY_DN6505_c0_g1/TRINITY_DN12228_c1_g1	
GO:0030198	extracellular matrix organization	12/2626	61/33142	0.002689	0.018188	0.013618	TRINITY_DN950_c4_g1/TRINITY_DN5239_c1_g2/TRINITY_DN1266_c0_g1/TRINITY_DN15399_c0_g1/TRINITY_DN2476_c1_g1/TRINITY_DN18049_c0_g1/TRINITY_DN56782_c0_g1/TRINITY_DN2281_c0_g1/TRINITY_DN5908_c0_g1/TRINITY_DN13862_c0_g2/TRINITY_DN12958_c0_g1/TRINITY_DN16986_c0_g2	12
GO:0008021	synaptic vesicle	14/2626	77/33142	0.002697	0.018188	0.013618	TRINITY_DN4032_c0_g1/TRINITY_DN1487_c0_g2/TRINITY_DN4544_c0_g1/TRINITY_DN200_c0_g2/TRINITY_DN5859_c0_g1/TRINITY_DN1420_c0_g1/TRINITY_DN35269_c0_g1/TRINITY_DN1532_c0_g1/TRINITY_DN55372_c0_g1/TRINITY_DN273_c1_g1/TRINITY_DN11061_c0_g2/TRINITY_DN12650_c0_g1/TRINITY_DN901_c0_g1/TRINITY_DN52030_c0_g1	14
GO:0007413	axonal fasciculation	6/2626	19/33142	0.002706	0.018188	0.013618	TRINITY_DN1557_c0_g1/TRINITY_DN4169_c0_g2/TRINITY_DN4169_c0_g1/TRINITY_D	6

							N2475_c0_g1/TRINITY_DN13362_c0_g1/TRINITY_DN14663_c0_g1	
GO:0009888	tissue development	6/2626	19/33142	0.002706	0.018188	0.013618	TRINITY_DN1990_c0_g1/TRINITY_DN23921_c0_g1/TRINITY_DN1549_c0_g1/TRINITY_DN484_c0_g1/TRINITY_DN244_c3_g1/TRINITY_DN61025_c0_g1	6
GO:0042811	pheromone biosynthetic process	6/2626	19/33142	0.002706	0.018188	0.013618	TRINITY_DN638_c1_g1/TRINITY_DN28084_c0_g1/TRINITY_DN6435_c0_g3/TRINITY_DN9779_c0_g1/TRINITY_DN5693_c0_g2/TRINITY_DN12549_c0_g1	6
GO:0045664	regulation of neuron differentiation	6/2626	19/33142	0.002706	0.018188	0.013618	TRINITY_DN5296_c0_g1/TRINITY_DN8625_c0_g1/TRINITY_DN7687_c0_g1/TRINITY_DN4839_c0_g1/TRINITY_DN12889_c0_g1/TRINITY_DN4784_c1_g1	6
GO:0061512	protein localization to cilium	6/2626	19/33142	0.002706	0.018188	0.013618	TRINITY_DN15240_c0_g1/TRINITY_DN21912_c0_g1/TRINITY_DN2153_c0_g1/TRINITY_DN4554_c0_g1/TRINITY_DN7277_c2_g1/TRINITY_DN12092_c0_g3	6
GO:0005776	autophagosome	10/2626	46/33142	0.002773	0.018549	0.013888	TRINITY_DN16674_c1_g3/TRINITY_DN16674_c1_g2/TRINITY_DN4811_c0_g1/TRINITY_DN36153_c0_g3/TRINITY_DN1201_c3_g1/TRINITY_DN3614	10

							_c1_g1/TRINITY_DN75_c2_g1/TRINITY_DN8911_c1_g2/TRINITY_DN901_c0_g1/TRINITY_DN901_c0_g2	
GO:0042995	cell projection	10/2626	46/33142	0.002773	0.018549	0.013888	TRINITY_DN3245_c0_g1/TRINITY_DN4544_c0_g1/TRINITY_DN235_c0_g1/TRINITY_DN15574_c0_g1/TRINITY_DN2557_c0_g2/TRINITY_DN1614_c0_g1/TRINITY_DN130_c0_g1/TRINITY_DN2372_c0_g1/TRINITY_DN8181_c0_g1/TRINITY_DN7084_c0_g1	10
GO:0016477	cell migration	16/2626	94/33142	0.002815	0.018784	0.014064	TRINITY_DN21789_c0_g1/TRINITY_DN4329_c1_g1/TRINITY_DN8257_c0_g1/TRINITY_DN32456_c0_g2/TRINITY_DN10076_c0_g1/TRINITY_DN4441_c3_g1/TRINITY_DN2241_c0_g2/TRINITY_DN2331_c0_g1/TRINITY_DN144_c0_g1/TRINITY_DN77356_c0_g1/TRINITY_DN339_c0_g2/TRINITY_DN2281_c0_g1/TRINITY_DN23609_c0_g1/TRINITY_DN14663_c0_g1/TRINITY_DN1186_c0_g1/TRINITY_DN2285_c0_g1	16
GO:0001649	osteoblast differentiation	8/2626	32/33142	0.002878	0.019095	0.014297	TRINITY_DN4002_c0_g3/TRINITY_DN3732_c0_g1/TRINITY_DN9845_c0_g2/TRINITY_DN9780_c0_g1/TRINITY_DN3753_c0_g1/TRINITY_DN4002_c	8

							0_g1/TRINITY_DN12650_c0_g1/TRINITY_DN40189_c0_g1	
GO:0035159	regulation of tube length, open tracheal system	8/2626	32/33142	0.002878	0.019095	0.014297	TRINITY_DN16444_c1_g1/TRINITY_DN2244_c1_g1/TRINITY_DN1990_c0_g1/TRINITY_DN1267_c0_g2/TRINITY_DN43399_c0_g1/TRINITY_DN27045_c0_g1/TRINITY_DN446_c1_g1/TRINITY_DN3178_c0_g1	8
GO:0035556	intracellular signal transduction	30/2626	222/33142	0.002882	0.019095	0.014297	TRINITY_DN9943_c0_g1/TRINITY_DN2841_c0_g1/TRINITY_DN2489_c0_g1/TRINITY_DN20439_c0_g1/TRINITY_DN15863_c0_g1/TRINITY_DN3725_c3_g1/TRINITY_DN20653_c0_g1/TRINITY_DN291_c0_g1/TRINITY_DN10783_c0_g1/TRINITY_DN5875_c0_g1/TRINITY_DN755_c1_g1/TRINITY_DN1902_c0_g1/TRINITY_DN6136_c0_g1/TRINITY_DN11938_c0_g1/TRINITY_DN51589_c0_g2/TRINITY_DN13614_c1_g1/TRINITY_DN3665_c0_g1/TRINITY_DN5267_c0_g1/TRINITY_DN9843_c0_g1/TRINITY_DN5234_c0_g1/TRINITY_DN6839_c2_g1/TRINITY_DN6910_c0_g1/TRINITY_DN5506_c0_g3/TRINITY_DN26007_c0_g2/TRINITY_DN4529_c4_g1/TRINITY_DN2011_c0_g1/TRINITY_D	30

insects MDPI

							N22945_c0_g1/TRINITY_DN2094_c0_g1/TRINITY_DN26727_c0_g1/TRINITY_DN8692_c0_g1	
GO:0005262	calcium channel activity	9/2626	39/33142	0.002899	0.019166	0.01435	TRINITY_DN6597_c0_g1/TRINITY_DN8167_c0_g1/TRINITY_DN17739_c0_g2/TRINITY_DN3180_c0_g1/TRINITY_DN11156_c0_g5/TRINITY_DN18131_c0_g1/TRINITY_DN35025_c0_g1/TRINITY_DN4522_c0_g1/TRINITY_DN10344_c0_g1	9
GO:0098793	presynapse	15/2626	87/33142	0.003307	0.021815	0.016334	TRINITY_DN2841_c0_g1/TRINITY_DN834_c1_g1/TRINITY_DN5470_c0_g1/TRINITY_DN5049_c0_g1/TRINITY_DN15308_c0_g2/TRINITY_DN1633_c0_g1/TRINITY_DN40503_c0_g1/TRINITY_DN6910_c0_g1/TRINITY_DN7687_c0_g1/TRINITY_DN5274_c0_g1/TRINITY_DN7523_c0_g1/TRINITY_DN3890_c0_g1/TRINITY_DN12650_c0_g1/TRINITY_DN6409_c3_g1/TRINITY_DN6505_c0_g1	15
GO:0008138	protein tyrosine/serine/threonine phosphatase activity	7/2626	26/33142	0.003333	0.021853	0.016362	TRINITY_DN3655_c4_g1/TRINITY_DN6775_c0_g2/TRINITY_DN3296_c0_g1/TRINITY_DN51821_c0_g1/TRINITY_DN645_c0_g1/TRINITY_DN443_c0_g1/TRINITY_DN6775_c0_g1	7

GO:0047372	acylglycerol lipase activity	7/2626	26/33142	0.003333	0.021853	0.016362	TRINITY_DN1568_c0_g1/TRINITY_DN2675_c0_g1/TRINITY_DN4211_c1_g3/TRINITY_DN18499_c0_g1/TRINITY_DN15468_c0_g1/TRINITY_DN26376_c0_g1/TRINITY_DN5274_c0_g1	7
GO:0005326	neurotransmitter transporter activity	5/2626	14/33142	0.003391	0.021853	0.016362	TRINITY_DN183_c0_g1/TRINITY_DN5871_c1_g1/TRINITY_DN27259_c0_g1/TRINITY_DN4029_c0_g1/TRINITY_DN4674_c0_g1	5
GO:0006836	neurotransmitter transport	5/2626	14/33142	0.003391	0.021853	0.016362	TRINITY_DN4032_c0_g1/TRINITY_DN18454_c0_g1/TRINITY_DN5859_c0_g1/TRINITY_DN19635_c0_g1/TRINITY_DN4029_c0_g1	5
GO:0006937	regulation of muscle contraction	5/2626	14/33142	0.003391	0.021853	0.016362	TRINITY_DN16891_c0_g1/TRINITY_DN3032_c1_g2/TRINITY_DN982_c0_g1/TRINITY_DN39828_c0_g1/TRINITY_DN7318_c0_g1	5
GO:0009566	fertilization	5/2626	14/33142	0.003391	0.021853	0.016362	TRINITY_DN23053_c0_g1/TRINITY_DN18484_c0_g1/TRINITY_DN15863_c0_g1/TRINITY_DN5680_c0_g1/TRINITY_DN14429_c0_g1	5
GO:0031941	filamentous actin	5/2626	14/33142	0.003391	0.021853	0.016362	TRINITY_DN10760_c0_g1/TRINITY_DN5459_c0_g1/TRINITY_DN4441_c3_g1/TRINITY_DN471_c1_g1/TRINITY_DN51821_c0_g1	5

GO:0032100	positive regulation of appetite	5/2626	14/33142	0.003391	0.021853	0.016362	TRINITY_DN606_c3_g1/TRINITY_DN19001_c0_g1/TRINITY_DN1549_c0_g1/TRINITY_DN484_c0_g1/TRINITY_DN14134_c0_g1	5
GO:0050482	arachidonic acid secretion	5/2626	14/33142	0.003391	0.021853	0.016362	TRINITY_DN3991_c0_g1/TRINITY_DN2682_c0_g1/TRINITY_DN26504_c0_g2/TRINITY_DN29189_c0_g1/TRINITY_DN2413_c0_g1	5
GO:0060976	coronary vasculature development	5/2626	14/33142	0.003391	0.021853	0.016362	TRINITY_DN1893_c0_g1/TRINITY_DN2448_c0_g1/TRINITY_DN9110_c0_g1/TRINITY_DN9158_c3_g1/TRINITY_DN709_c0_g1	5
GO:0006103	2-oxoglutarate metabolic process	9/2626	40/33142	0.003482	0.022293	0.016692	TRINITY_DN15486_c0_g2/TRINITY_DN18587_c0_g2/TRINITY_DN5262_c1_g1/TRINITY_DN6037_c0_g1/TRINITY_DN7264_c0_g1/TRINITY_DN5233_c1_g1/TRINITY_DN5675_c0_g1/TRINITY_DN7264_c0_g3/TRINITY_DN4895_c1_g1	9
GO:0051124	synaptic growth at neuromuscular junction	9/2626	40/33142	0.003482	0.022293	0.016692	TRINITY_DN9170_c1_g1/TRINITY_DN10783_c0_g1/TRINITY_DN1267_c0_g2/TRINITY_DN4620_c0_g1/TRINITY_DN4029_c0_g1/TRINITY_DN3103_c0_g2/TRINITY_DN428_c0_g1/TRINITY_DN3103_c2_g1/TRINITY_DN6589_c0_g1	9

insects MDPI

GO:0042060	wound healing	11/2626	55/33142	0.003483	0.022293	0.016692	TRINITY_DN1557_c0_g1/TRINITY_DN14835_c0_g1/TRINITY_DN5680_c0_g1/TRINITY_DN6348_c0_g1/TRINITY_DN9245_c0_g1/TRINITY_DN3148_c0_g1/TRINITY_DN2869_c2_g1/TRINITY_DN2321_c0_g1/TRINITY_DN13862_c0_g2/TRINITY_DN1186_c0_g1/TRINITY_DN6589_c0_g1	11
GO:0001738	morphogenesis of a polarized epithelium	6/2626	20/33142	0.003609	0.022845	0.017105	TRINITY_DN2244_c1_g1/TRINITY_DN13406_c0_g1/TRINITY_DN43399_c0_g1/TRINITY_DN27045_c0_g1/TRINITY_DN2281_c0_g1/TRINITY_DN446_c1_g1	6
GO:0006565	L-serine catabolic process	6/2626	20/33142	0.003609	0.022845	0.017105	TRINITY_DN1440_c0_g2/TRINITY_DN10554_c0_g2/TRINITY_DN10867_c0_g1/TRINITY_DN291_c1_g1/TRINITY_DN3638_c0_g1/TRINITY_DN17288_c0_g1	6
GO:0007464	R3/R4 cell fate commitment	6/2626	20/33142	0.003609	0.022845	0.017105	TRINITY_DN2244_c1_g1/TRINITY_DN2133_c0_g1/TRINITY_DN43399_c0_g1/TRINITY_DN27045_c0_g1/TRINITY_DN446_c1_g1/TRINITY_DN6589_c0_g1	6
GO:0016082	synaptic vesicle priming	6/2626	20/33142	0.003609	0.022845	0.017105	TRINITY_DN31967_c0_g1/TRINITY_DN11938_c0_g1/TRINITY_DN1420_c0_g1/TRINITY_DN25715_c0_g1/TRINITY_DN	6

							1532_c0_g1/TRINITY_DN11061_c0_g2	
GO:0097729	9+2 motile cilium	6/2626	20/33142	0.003609	0.022845	0.017105	TRINITY_DN7740_c0_g1/TRINITY_DN9451_c0_g1/TRINITY_DN18134_c0_g1/TRINITY_DN9647_c0_g1/TRINITY_DN59259_c0_g1/TRINITY_DN63431_c0_g1	6
GO:0031902	late endosome membrane	18/2626	114/33142	0.003712	0.023445	0.017554	TRINITY_DN8323_c0_g1/TRINITY_DN3446_c0_g1/TRINITY_DN8493_c0_g1/TRINITY_DN4783_c0_g1/TRINITY_DN43642_c0_g1/TRINITY_DN834_c1_g1/TRINITY_DN13394_c0_g1/TRINITY_DN5278_c0_g1/TRINITY_DN8257_c0_g1/TRINITY_DN8012_c0_g1/TRINITY_DN6603_c0_g1/TRINITY_DN18131_c0_g1/TRINITY_DN5454_c0_g1/TRINITY_DN2085_c0_g2/TRINITY_DN273_c1_g1/TRINITY_DN2006_c1_g2/TRINITY_DN901_c0_g1/TRINITY_DN901_c0_g2	18
GO:0006816	calcium ion transport	10/2626	48/33142	0.00385	0.024259	0.018163	TRINITY_DN6597_c0_g1/TRINITY_DN1748_c0_g1/TRINITY_DN14379_c0_g1/TRINITY_DN6174_c0_g1/TRINITY_DN8167_c0_g1/TRINITY_DN17739_c0_g2/TRINITY_DN15120_c0_g1/TRINITY_DN3180_c0_g1/	10

GO:0006914	autophagy	16/2626	97/33142	0.003887	0.024438	0.018298	TRINITY_DN35025_c0_g1/TRINITY_DN4522_c0_g1 TRINITY_DN2578_c0_g1/TRINITY_DN32305_c0_g1/TRINITY_DN5056_c0_g1/TRINITY_DN1748_c0_g1/TRINITY_DN7743_c0_g1/TRINITY_DN1169_c0_g1/TRINITY_DN36153_c0_g3/TRINITY_DN755_c1_g1/TRINITY_DN1178_c2_g1/TRINITY_DN3614_c1_g1/TRINITY_DN8278_c2_g1/TRINITY_DN4517_c0_g2/TRINITY_DN40503_c0_g1/TRINITY_DN8911_c1_g2/TRINITY_DN2611_c1_g1/TRINITY_DN1119_c0_g1	16
GO:0003796	lysozyme activity	9/2626	41/33142	0.004153	0.025937	0.01942	TRINITY_DN70231_c0_g1/TRINITY_DN11824_c0_g1/TRINITY_DN36027_c0_g1/TRINITY_DN66315_c3_g1/TRINITY_DN45601_c0_g1/TRINITY_DN2305_c2_g1/TRINITY_DN2479_c0_g1/TRINITY_DN66315_c1_g1/TRINITY_DN2058_c7_g1	9
GO:0004550	nucleoside diphosphate kinase activity	9/2626	41/33142	0.004153	0.025937	0.01942	TRINITY_DN7530_c0_g1/TRINITY_DN16032_c0_g1/TRINITY_DN2009_c0_g1/TRINITY_DN9292_c0_g1/TRINITY_DN2881_c0_g1/TRINITY_DN22883_c0_g1/TRINITY_DN2881_c0_g2/TRINITY_DN11858_c0_g1/TRINITY_DN7067_c0_g1	9

GO:0006536	glutamate metabolic process	9/2626	41/33142	0.004153	0.025937	0.01942	TRINITY_DN9506_c0_g2/TRINITY_DN15486_c0_g2/TRINITY_DN9506_c0_g1/TRINITY_DN3007_c0_g1/TRINITY_DN2557_c0_g2/TRINITY_DN2779_c1_g2/TRINITY_DN6037_c0_g1/TRINITY_DN2779_c1_g3/TRINITY_DN4895_c1_g1	9
GO:0010977	negative regulation of neuron projection development	7/2626	27/33142	0.004195	0.026145	0.019576	TRINITY_DN16186_c0_g1/TRINITY_DN20653_c0_g1/TRINITY_DN10760_c0_g1/TRINITY_DN3722_c0_g1/TRINITY_DN93_c0_g1/TRINITY_DN2951_c0_g1/TRINITY_DN8692_c0_g1	7
GO:0007417	central nervous system development	16/2626	98/33142	0.004311	0.026633	0.019941	TRINITY_DN5056_c0_g1/TRINITY_DN22183_c0_g2/TRINITY_DN4210_c0_g1/TRINITY_DN15554_c0_g1/TRINITY_DN800_c0_g2/TRINITY_DN8278_c2_g1/TRINITY_DN33241_c0_g1/TRINITY_DN339_c0_g2/TRINITY_DN30302_c0_g1/TRINITY_DN23609_c0_g1/TRINITY_DN9041_c0_g2/TRINITY_DN14168_c0_g2/TRINITY_DN7258_c0_g1/TRINITY_DN13354_c0_g1/TRINITY_DN689_c0_g1/TRINITY_DN21399_c0_g1	16
GO:0035149	lumen formation, open tracheal system	8/2626	34/33142	0.004311	0.026633	0.019941	TRINITY_DN439_c0_g1/TRINITY_DN11631_c0_g3/TRINITY_DN6790_c0_g1/TRINITY_DN1350_c0_g2/TRINITY_DN3312	8

insects

MDPI

							_c0_g1/TRINITY_DN3305_c0_g1/TRINITY_DN6810_c0_g1/TRINITY_DN18007_c0_g1	
GO:0051897	positive regulation of protein kinase B signaling	8/2626	34/33142	0.004311	0.026633	0.019941	TRINITY_DN23953_c0_g1/TRINITY_DN10783_c0_g1/TRINITY_DN9755_c0_g1/TRINITY_DN9853_c0_g1/TRINITY_DN1908_c0_g1/TRINITY_DN6505_c0_g1/TRINITY_DN7471_c0_g1/TRINITY_DN4784_c2_g1	8
GO:0055088	lipid homeostasis	8/2626	34/33142	0.004311	0.026633	0.019941	TRINITY_DN8909_c0_g1/TRINITY_DN11372_c0_g1/TRINITY_DN7048_c0_g1/TRINITY_DN4514_c0_g1/TRINITY_DN30087_c1_g2/TRINITY_DN11204_c0_g1/TRINITY_DN9281_c0_g1/TRINITY_DN1852_c2_g1	8
GO:0031594	neuromuscular junction	14/2626	81/33142	0.004352	0.026827	0.020087	TRINITY_DN1748_c0_g1/TRINITY_DN10890_c0_g1/TRINITY_DN5049_c0_g1/TRINITY_DN31967_c0_g1/TRINITY_DN2531_c0_g1/TRINITY_DN8977_c0_g1/TRINITY_DN10783_c0_g1/TRINITY_DN11938_c0_g1/TRINITY_DN4620_c0_g1/TRINITY_DN4029_c0_g1/TRINITY_DN2281_c0_g1/TRINITY_DN11061_c0_g2/TRINITY_DN6945_c0_g2/TRINITY_DN18471_c0_g2	14
GO:0004315	3-oxoacyl-[acyl-carrier-protein] synthase activity	10/2626	49/33142	0.0045	0.027555	0.020632	TRINITY_DN606_c3_g1/TRINITY_DN19001_c0_g1/TRINITY	10

							_DN23921_c0_g1/TRINITY_DN482_c1_g1/TRINITY_DN29856_c0_g1/TRINITY_DN1549_c0_g1/TRINITY_DN40189_c0_g1/TRINITY_DN484_c0_g1/TRINITY_DN244_c3_g1/TRINITY_DN61025_c0_g1	
GO:0042593	glucose homeostasis	10/2626	49/33142	0.0045	0.027555	0.020632	TRINITY_DN8485_c0_g1/TRINITY_DN15343_c0_g1/TRINITY_DN13981_c0_g1/TRINITY_DN1178_c2_g1/TRINITY_DN6365_c0_g1/TRINITY_DN9063_c0_g1/TRINITY_DN40503_c0_g1/TRINITY_DN39722_c0_g1/TRINITY_DN5908_c0_g1/TRINITY_DN7705_c0_g1	10
GO:0051259	protein complex oligomerization	10/2626	49/33142	0.0045	0.027555	0.020632	TRINITY_DN3120_c0_g1/TRINITY_DN1568_c0_g1/TRINITY_DN10137_c0_g1/TRINITY_DN1610_c0_g1/TRINITY_DN4849_c0_g1/TRINITY_DN9175_c0_g1/TRINITY_DN13011_c0_g1/TRINITY_DN3354_c0_g1/TRINITY_DN22945_c0_g1/TRINITY_DN6300_c0_g1	10
GO:0001947	heart looping	6/2626	21/33142	0.004718	0.028512	0.021348	TRINITY_DN6327_c1_g1/TRINITY_DN5006_c0_g2/TRINITY_DN11340_c0_g1/TRINITY_DN5914_c0_g1/TRINITY_DN15411_c0_g1/TRINITY_DN4751_c0_g2	6

GO:0006919	activation of cysteine-type endopeptidase activity involved in apoptotic process	6/2626	21/33142	0.004718	0.028512	0.021348	TRINITY_DN6327_c1_g1/TRINITY_DN1169_c0_g1/TRINITY_DN9158_c3_g1/TRINITY_DN709_c0_g1/TRINITY_DN56840_c0_g1/TRINITY_DN6505_c0_g1	6
GO:0016079	synaptic vesicle exocytosis	6/2626	21/33142	0.004718	0.028512	0.021348	TRINITY_DN9170_c1_g1/TRINITY_DN28373_c0_g1/TRINITY_DN25715_c0_g1/TRINITY_DN19224_c0_g1/TRINITY_DN4029_c0_g1/TRINITY_DN2165_c3_g1	6
GO:0099055	integral component of postsynaptic membrane	6/2626	21/33142	0.004718	0.028512	0.021348	TRINITY_DN11100_c0_g1/TRINITY_DN9375_c0_g1/TRINITY_DN7687_c0_g1/TRINITY_DN12650_c0_g1/TRINITY_DN49433_c0_g1/TRINITY_DN18471_c0_g2	6
GO:0001935	endothelial cell proliferation	5/2626	15/33142	0.004757	0.028512	0.021348	TRINITY_DN5364_c0_g1/TRINITY_DN5086_c0_g1/TRINITY_DN40503_c0_g1/TRINITY_DN4137_c2_g1/TRINITY_DN333_c0_g1	5
GO:0005246	calcium channel regulator activity	5/2626	15/33142	0.004757	0.028512	0.021348	TRINITY_DN15120_c0_g1/TRINITY_DN22381_c0_g1/TRINITY_DN31435_c0_g1/TRINITY_DN32337_c0_g1/TRINITY_DN49433_c0_g1	5
GO:0034612	response to tumor necrosis factor	5/2626	15/33142	0.004757	0.028512	0.021348	TRINITY_DN1557_c0_g1/TRINITY_DN3007_c0_g1/TRINITY_DN6108_c0_g1/TRINITY_D	5

							N2779_c1_g2/TRINITY_DN2779_c1_g3	
GO:0042730	fibrinolysis	5/2626	15/33142	0.004757	0.028512	0.021348	TRINITY_DN21131_c0_g2/TRINITY_DN11048_c0_g1/TRINITY_DN45371_c0_g1/TRINITY_DN2734_c0_g1/TRINITY_DN56782_c0_g1	5
GO:0060071	Wnt signaling pathway, planar cell polarity pathway	5/2626	15/33142	0.004757	0.028512	0.021348	TRINITY_DN2244_c1_g1/TRINITY_DN1990_c0_g1/TRINITY_DN43399_c0_g1/TRINITY_DN27045_c0_g1/TRINITY_DN446_c1_g1	5
GO:0070328	triglyceride homeostasis	5/2626	15/33142	0.004757	0.028512	0.021348	TRINITY_DN1568_c0_g1/TRINITY_DN5364_c0_g1/TRINITY_DN5086_c0_g1/TRINITY_DN333_c0_g1/TRINITY_DN15502_c0_g1	5
GO:0007169	transmembrane receptor protein tyrosine kinase signaling pathway	9/2626	42/33142	0.00492	0.029305	0.021942	TRINITY_DN6348_c0_g1/TRINITY_DN1902_c0_g1/TRINITY_DN14619_c0_g1/TRINITY_DN8625_c0_g1/TRINITY_DN339_c0_g2/TRINITY_DN9924_c0_g1/TRINITY_DN2321_c0_g1/TRINITY_DN4839_c0_g1/TRINITY_DN14663_c0_g1	9
GO:0030307	positive regulation of cell growth	9/2626	42/33142	0.00492	0.029305	0.021942	TRINITY_DN6108_c0_g1/TRINITY_DN19520_c0_g1/TRINITY_DN17565_c0_g1/TRINITY_DN2327_c0_g1/TRINITY_DN10967_c0_g2/TRINITY_DN32376_c1_g1/TRINITY_DN93_c0_	9

insects MDPI

GO:0070588	calcium ion transmembrane transport	9/2626	42/33142	0.00492	0.029305	0.021942	g1/TRINITY_DN8729_c1_g1/TRINITY_DN1071_c0_g1 TRINITY_DN1748_c0_g1/TRINITY_DN10654_c0_g1/TRINITY_DN17739_c0_g2/TRINITY_DN15120_c0_g1/TRINITY_DN3180_c0_g1/TRINITY_DN18131_c0_g1/TRINITY_DN35025_c0_g1/TRINITY_DN2197_c0_g1/TRINITY_DN18471_c0_g2	9
GO:0042626	ATPase activity, coupled to transmembrane movement of substances	23/2626	163/33142	0.004936	0.029335	0.021964	TRINITY_DN8493_c0_g1/TRINITY_DN7048_c0_g1/TRINITY_DN406_c0_g3/TRINITY_DN4514_c0_g1/TRINITY_DN5690_c0_g1/TRINITY_DN5049_c0_g1/TRINITY_DN2999_c0_g1/TRINITY_DN1216_c2_g1/TRINITY_DN23353_c2_g1/TRINITY_DN5370_c0_g1/TRINITY_DN1852_c2_g1/TRINITY_DN406_c0_g2/TRINITY_DN10757_c0_g1/TRINITY_DN7045_c1_g1/TRINITY_DN7512_c0_g1/TRINITY_DN4554_c0_g1/TRINITY_DN19729_c0_g3/TRINITY_DN5012_c0_g1/TRINITY_DN1746_c0_g1/TRINITY_DN6225_c0_g1/TRINITY_DN6409_c3_g1/TRINITY_DN42565_c0_g1/TRINITY_DN38748_c0_g1	23
GO:0006812	cation transport	15/2626	91/33142	0.005117	0.030349	0.022723	TRINITY_DN6597_c0_g1/TRINITY_DN16444_c1_g1/TRINI	15

insects

MDPI

GO:0005759	mitochondrial matrix	39/2626	321/33142	0.005154	0.030503	0.022838	TY_DN8167_c0_g1/TRINITY_DN6323_c1_g1/TRINITY_DN2924_c1_g1/TRINITY_DN7737_c0_g1/TRINITY_DN1267_c0_g2/TRINITY_DN10811_c0_g1/TRINITY_DN7054_c0_g1/TRINITY_DN4220_c0_g1/TRINITY_DN15420_c0_g1/TRINITY_DN35025_c0_g1/TRINITY_DN4522_c0_g1/TRINITY_DN4784_c1_g1/TRINITY_DN56423_c0_g2TRINITY_DN13575_c0_g1/TRINITY_DN9506_c0_g2/TRINITY_DN15486_c0_g2/TRINITY_DN8805_c1_g1/TRINITY_DN10162_c0_g1/TRINITY_DN23924_c0_g1/TRINITY_DN6049_c1_g2/TRINITY_DN1808_c0_g2/TRINITY_DN9506_c0_g1/TRINITY_DN7625_c0_g1/TRINITY_DN8254_c0_g1/TRINITY_DN41254_c0_g1/TRINITY_DN13785_c0_g1/TRINITY_DN365_c5_g1/TRINITY_DN1580_c0_g1/TRINITY_DN8848_c0_g1/TRINITY_DN29993_c0_g1/TRINITY_DN5502_c0_g1/TRINITY_DN6436_c2_g2/TRINITY_DN271_c0_g1/TRINITY_DN1727_c2_g1/TRINITY_DN1808_c0_g3/TRINITY_DN2654_c0_g1/TRINITY_DN17694_c0_g1/TRINI	39

							TY_DN9780_c0_g1/TRINITY_DN4859_c0_g1/TRINITY_DN21888_c1_g1/TRINITY_DN1517_c0_g1/TRINITY_DN11473_c0_g1/TRINITY_DN608_c0_g1/TRINITY_DN3559_c0_g1/TRINITY_DN15536_c0_g1/TRINITY_DN9703_c0_g1/TRINITY_DN80403_c0_g1/TRINITY_DN10720_c0_g1/TRINITY_DN12441_c0_g1/TRINITY_DN23860_c0_g1/TRINITY_DN10344_c0_g1/TRINITY_DN20231_c0_g1	
GO:0044255	cellular lipid metabolic process	8/2626	35/33142	0.005207	0.030748	0.023022	TRINITY_DN6455_c0_g1/TRINITY_DN77269_c0_g1/TRINITY_DN21814_c0_g2/TRINITY_DN23263_c0_g1/TRINITY_DN4485_c0_g1/TRINITY_DN2168_c4_g1/TRINITY_DN5833_c0_g1/TRINITY_DN16751_c0_g1	8
GO:0005901	caveola	7/2626	28/33142	0.005217	0.030748	0.023022	TRINITY_DN5364_c0_g1/TRINITY_DN5086_c0_g1/TRINITY_DN200_c0_g2/TRINITY_DN233_c0_g1/TRINITY_DN19100_c0_g1/TRINITY_DN333_c0_g1/TRINITY_DN5922_c0_g1	7
GO:0042981	regulation of apoptotic process	12/2626	66/33142	0.005268	0.030982	0.023197	TRINITY_DN6327_c1_g1/TRINITY_DN9767_c0_g3/TRINITY_DN2476_c1_g1/TRINITY_DN6947_c0_g1/TRINITY_DN18321_c0_g1/TRINITY_DN37845	12

							_c0_g1/TRINITY_DN1797_c0_g1/TRINITY_DN8625_c0_g1/TRINITY_DN1699_c1_g1/TRINITY_DN6643_c0_g1/TRINITY_DN4839_c0_g1/TRINITY_DN6505_c0_g1	
GO:0001894	tissue homeostasis	4/2626	10/33142	0.005595	0.032105	0.024038	TRINITY_DN658_c0_g1/TRINITY_DN9281_c0_g1/TRINITY_DN1267_c0_g2/TRINITY_DN1178_c2_g1	4
GO:0003091	renal water homeostasis	4/2626	10/33142	0.005595	0.032105	0.024038	TRINITY_DN658_c0_g1/TRINITY_DN17778_c0_g1/TRINITY_DN20653_c0_g1/TRINITY_DN6839_c2_g1	4
GO:0003990	acetylcholinesterase activity	4/2626	10/33142	0.005595	0.032105	0.024038	TRINITY_DN13673_c0_g1/TRINITY_DN9375_c0_g1/TRINITY_DN2207_c0_g1/TRINITY_DN1133_c1_g2	4
GO:0004114	3', 5'-cyclic-nucleotide phosphodiesterase activity	4/2626	10/33142	0.005595	0.032105	0.024038	TRINITY_DN5339_c0_g1/TRINITY_DN4899_c1_g1/TRINITY_DN5951_c0_g4/TRINITY_DN10344_c0_g1	4
GO:0004613	phosphoenolpyruvate carboxykinase (GTP) activity	4/2626	10/33142	0.005595	0.032105	0.024038	TRINITY_DN1015_c0_g2/TRINITY_DN2378_c0_g1/TRINITY_DN1015_c0_g3/TRINITY_DN8848_c0_g1	4
GO:0032780	negative regulation of ATPase activity	4/2626	10/33142	0.005595	0.032105	0.024038	TRINITY_DN7684_c0_g1/TRINITY_DN30262_c0_g1/TRINITY_DN11189_c0_g1/TRINITY_DN3574_c0_g1	4
GO:0040040	thermosensory behavior	4/2626	10/33142	0.005595	0.032105	0.024038	TRINITY_DN161_c0_g1/TRINITY_DN7807_c0_g1/TRINITY_	4

							DN18321_c0_g1/TRINITY_DN4784_c1_g1	
GO:0043325	phosphatidylinositol-3, 4-bisphosphate binding	4/2626	10/33142	0.005595	0.032105	0.024038	TRINITY_DN19161_c0_g1/TRINITY_DN2629_c0_g1/TRINITY_DN8977_c0_g1/TRINITY_DN6505_c0_g1	4
GO:0048060	negative gravitaxis	4/2626	10/33142	0.005595	0.032105	0.024038	TRINITY_DN6597_c0_g1/TRINITY_DN8167_c0_g1/TRINITY_DN11596_c0_g1/TRINITY_DN4522_c0_g1	4
GO:0048067	cuticle pigmentation	4/2626	10/33142	0.005595	0.032105	0.024038	TRINITY_DN8260_c0_g2/TRINITY_DN7807_c0_g1/TRINITY_DN855_c0_g1/TRINITY_DN4947_c0_g1	4
GO:0048839	inner ear development	4/2626	10/33142	0.005595	0.032105	0.024038	TRINITY_DN6174_c0_g1/TRINITY_DN47_c0_g1/TRINITY_DN16407_c1_g1/TRINITY_DN1541_c0_g1	4
GO:0050905	neuromuscular process	4/2626	10/33142	0.005595	0.032105	0.024038	TRINITY_DN16600_c0_g1/TRINITY_DN1267_c0_g2/TRINITY_DN16751_c0_g1/TRINITY_DN579_c0_g1	4
GO:0004180	carboxypeptidase activity	9/2626	43/33142	0.005794	0.033177	0.024841	TRINITY_DN3991_c0_g1/TRINITY_DN16579_c1_g1/TRINITY_DN2682_c0_g1/TRINITY_DN3991_c0_g2/TRINITY_DN1773_c1_g2/TRINITY_DN9651_c0_g1/TRINITY_DN13564_c0_g1/TRINITY_DN4541_c0_g1/TRINITY_DN11117_c1_g1	9
GO:0006954	inflammatory response	10/2626	51/33142	0.006057	0.034484	0.025819	TRINITY_DN6931_c0_g1/TRINITY_DN22536_c0_g1/TRINI	10

							TY_DN10890_c0_g1/TRINITY_DN3120_c0_g1/TRINITY_DN755_c1_g1/TRINITY_DN271_c0_g1/TRINITY_DN2292_c2_g1/TRINITY_DN489_c1_g1/TRINITY_DN5431_c0_g1/TRINITY_DN5031_c0_g1	
GO:0009166	nucleotide catabolic process	6/2626	22/33142	0.006059	0.034484	0.025819	TRINITY_DN24800_c0_g1/TRINITY_DN9314_c0_g1/TRINITY_DN434_c0_g1/TRINITY_DN595_c0_g3/TRINITY_DN9430_c1_g1/TRINITY_DN2810_c0_g2	6
GO:0042043	neurexin family protein binding	6/2626	22/33142	0.006059	0.034484	0.025819	TRINITY_DN9375_c0_g1/TRINITY_DN15308_c0_g2/TRINITY_DN17565_c0_g1/TRINITY_DN7687_c0_g1/TRINITY_DN5274_c0_g1/TRINITY_DN7523_c0_g1	6
GO:0019730	antimicrobial humoral response	7/2626	29/33142	0.006415	0.0359	0.026879	TRINITY_DN2476_c1_g1/TRINITY_DN2133_c0_g1/TRINITY_DN5173_c0_g1/TRINITY_DN1338_c0_g1/TRINITY_DN3284_c0_g1/TRINITY_DN68176_c0_g1/TRINITY_DN25627_c0_g1	7
GO:0045165	cell fate commitment	7/2626	29/33142	0.006415	0.0359	0.026879	TRINITY_DN1893_c0_g1/TRINITY_DN1557_c0_g1/TRINITY_DN5239_c1_g2/TRINITY_DN2778_c0_g3/TRINITY_DN419_c0_g2/TRINITY_DN23750_c	7

							0_g1/TRINITY_DN77356_c0_g1	
GO:0051491	positive regulation of filopodium assembly	7/2626	29/33142	0.006415	0.0359	0.026879	TRINITY_DN2489_c0_g1/TRINITY_DN2738_c0_g1/TRINITY_DN10760_c0_g1/TRINITY_DN740_c0_g1/TRINITY_DN5459_c0_g1/TRINITY_DN2598_c0_g2/TRINITY_DN7687_c0_g1	7
GO:0071333	cellular response to glucose stimulus	7/2626	29/33142	0.006415	0.0359	0.026879	TRINITY_DN1015_c0_g2/TRINITY_DN2378_c0_g1/TRINITY_DN8848_c0_g1/TRINITY_DN11938_c0_g1/TRINITY_DN40503_c0_g1/TRINITY_DN4641_c0_g1/TRINITY_DN7471_c0_g1	7
GO:0003148	outflow tract septum morphogenesis	5/2626	16/33142	0.006473	0.0359	0.026879	TRINITY_DN7437_c0_g1/TRINITY_DN11100_c0_g1/TRINITY_DN85_c0_g1/TRINITY_DN9158_c3_g1/TRINITY_DN709_c0_g1	5
GO:0005313	L-glutamate transmembrane transporter activity	5/2626	16/33142	0.006473	0.0359	0.026879	TRINITY_DN1709_c0_g1/TRINITY_DN27259_c0_g1/TRINITY_DN5199_c0_g1/TRINITY_DN6690_c1_g1/TRINITY_DN4674_c0_g1	5
GO:0007622	rhythmic behavior	5/2626	16/33142	0.006473	0.0359	0.026879	TRINITY_DN7953_c1_g1/TRINITY_DN154_c0_g1/TRINITY_DN18321_c0_g1/TRINITY_DN29974_c0_g1/TRINITY_DN27661_c0_g1	5
GO:0015718	monocarboxylic acid transport	5/2626	16/33142	0.006473	0.0359	0.026879	TRINITY_DN8258_c0_g1/TRINITY_DN4523_c0_g1/TRINIT	5

							Y_DN1659_c1_g2/TRINITY_DN8515_c2_g1/TRINITY_DN1138_c0_g1	
GO:0016404	15-hydroxyprostaglandin dehydrogenase (NAD+) activity	5/2626	16/33142	0.006473	0.0359	0.026879	TRINITY_DN406_c0_g3/TRINITY_DN5049_c0_g1/TRINITY_DN2999_c0_g1/TRINITY_DN406_c0_g2/TRINITY_DN42565_c0_g1	5
GO:0030100	regulation of endocytosis	5/2626	16/33142	0.006473	0.0359	0.026879	TRINITY_DN1633_c0_g1/TRINITY_DN10575_c2_g1/TRINITY_DN6505_c0_g1/TRINITY_DN17100_c0_g1/TRINITY_DN8692_c0_g1	5
GO:0030553	cGMP binding	5/2626	16/33142	0.006473	0.0359	0.026879	TRINITY_DN2924_c1_g1/TRINITY_DN5951_c0_g4/TRINITY_DN4264_c1_g1/TRINITY_DN4784_c1_g1/TRINITY_DN10344_c0_g1	5
GO:0031463	Cul3-RING ubiquitin ligase complex	5/2626	16/33142	0.006473	0.0359	0.026879	TRINITY_DN16186_c0_g1/TRINITY_DN11676_c0_g1/TRINITY_DN13467_c0_g1/TRINITY_DN50069_c0_g1/TRINITY_DN2331_c0_g1	5
GO:0043052	thermotaxis	5/2626	16/33142	0.006473	0.0359	0.026879	TRINITY_DN161_c0_g1/TRINITY_DN7807_c0_g1/TRINITY_DN6805_c0_g1/TRINITY_DN2094_c0_g1/TRINITY_DN4784_c1_g1	5
GO:0004523	RNA-DNA hybrid ribonuclease activity	16/2626	103/33142	0.007037	0.03895	0.029163	TRINITY_DN1830_c0_g2/TRINITY_DN180_c0_g2/TRINITY_DN1070_c0_g3/TRINITY_DN103_c0_g1/TRINITY_DN6397_	16

							c2_g1/TRINITY_DN27616_c1_g1/TRINITY_DN23001_c0_g3/TRINITY_DN1252_c0_g1/TRINITY_DN24885_c0_g1/TRINITY_DN60975_c0_g1/TRINITY_DN7464_c1_g1/TRINITY_DN30055_c0_g1/TRINITY_DN392_c2_g1/TRINITY_DN12254_c0_g3/TRINITY_DN1589_c5_g1/TRINITY_DN13245_c0_g1	
GO:0010025	wax biosynthetic process	8/2626	37/33142	0.007418	0.040819	0.030563	TRINITY_DN2997_c0_g1/TRINITY_DN8066_c0_g1/TRINITY_DN57502_c0_g1/TRINITY_DN11631_c0_g3/TRINITY_DN3305_c0_g1/TRINITY_DN6810_c0_g1/TRINITY_DN1450_c0_g3/TRINITY_DN18007_c0_g1	8
GO:0035206	regulation of hemocyte proliferation	8/2626	37/33142	0.007418	0.040819	0.030563	TRINITY_DN2639_c0_g1/TRINITY_DN77781_c0_g1/TRINITY_DN20439_c0_g1/TRINITY_DN10890_c0_g1/TRINITY_DN60483_c1_g1/TRINITY_DN5519_c0_g1/TRINITY_DN3180_c0_g1/TRINITY_DN77356_c0_g1	8
GO:0060548	negative regulation of cell death	8/2626	37/33142	0.007418	0.040819	0.030563	TRINITY_DN11750_c0_g1/TRINITY_DN4514_c0_g1/TRINITY_DN7743_c0_g1/TRINITY_DN2629_c0_g1/TRINITY_DN14877_c1_g1/TRINITY_DN954_c0_g1/TRINITY_DN6189_c1_g2/TRINITY_DN3238_c0_g1	8

GO:0010628	positive regulation of gene expression	21/2626	150/33142	0.007652	0.041968	0.031423	TRINITY_DN1893_c0_g1/TRINITY_DN1691_c0_g2/TRINITY_DN4783_c0_g1/TRINITY_DN439_c0_g1/TRINITY_DN3120_c0_g1/TRINITY_DN402_c1_g1/TRINITY_DN1908_c3_g1/TRINITY_DN8903_c0_g1/TRINITY_DN18321_c0_g1/TRINITY_DN9281_c0_g1/TRINITY_DN15112_c0_g1/TRINITY_DN3722_c0_g1/TRINITY_DN2746_c0_g1/TRINITY_DN19100_c0_g1/TRINITY_DN9158_c3_g1/TRINITY_DN12127_c0_g1/TRINITY_DN709_c0_g1/TRINITY_DN4326_c0_g1/TRINITY_DN4481_c0_g1/TRINITY_DN4784_c1_g1/TRINITY_DN10344_c0_g1	21
GO:0016358	dendrite development	6/2626	23/33142	0.007656	0.041968	0.031423	TRINITY_DN3245_c0_g1/TRINITY_DN2244_c1_g1/TRINITY_DN43399_c0_g1/TRINITY_DN27045_c0_g1/TRINITY_DN446_c1_g1/TRINITY_DN1186_c0_g1	6
GO:0004725	protein tyrosine phosphatase activity	11/2626	61/33142	0.007868	0.042964	0.032169	TRINITY_DN3655_c4_g1/TRINITY_DN6775_c0_g2/TRINITY_DN3296_c0_g1/TRINITY_DN3417_c0_g1/TRINITY_DN51821_c0_g1/TRINITY_DN645_c0_g1/TRINITY_DN443_c0_g1/TRINITY_DN6775_c0_g1/TRI	11

							NITY_DN39318_c0_g1/TRINITY_DN6219_c0_g1/TRINITY_DN10111_c0_g1	
GO:0016209	antioxidant activity	11/2626	61/33142	0.007868	0.042964	0.032169	TRINITY_DN77781_c0_g1/TRINITY_DN9425_c0_g1/TRINITY_DN4836_c0_g1/TRINITY_DN60483_c1_g1/TRINITY_DN7756_c0_g1/TRINITY_DN84_c2_g1/TRINITY_DN271_c0_g1/TRINITY_DN13964_c0_g1/TRINITY_DN346_c0_g1/TRINITY_DN997_c0_g1/TRINITY_DN13697_c0_g1	11
GO:0007370	ventral furrow formation	4/2626	11/33142	0.008246	0.043755	0.032761	TRINITY_DN10159_c1_g1/TRINITY_DN6641_c0_g2/TRINITY_DN6641_c0_g1/TRINITY_DN5802_c0_g1	4
GO:0007588	excretion	4/2626	11/33142	0.008246	0.043755	0.032761	TRINITY_DN10137_c0_g1/TRINITY_DN12107_c0_g1/TRINITY_DN1610_c0_g1/TRINITY_DN6300_c0_g1	4
GO:0008241	peptidyl-dipeptidase activity	4/2626	11/33142	0.008246	0.043755	0.032761	TRINITY_DN3991_c0_g1/TRINITY_DN2682_c0_g1/TRINITY_DN3991_c0_g2/TRINITY_DN13564_c0_g1	4
GO:0016486	peptide hormone processing	4/2626	11/33142	0.008246	0.043755	0.032761	TRINITY_DN256_c0_g1/TRINITY_DN26373_c0_g1/TRINITY_DN31763_c0_g1/TRINITY_DN33745_c0_g1	4
GO:0030695	GTPase regulator activity	4/2626	11/33142	0.008246	0.043755	0.032761	TRINITY_DN11325_c0_g1/TRINITY_DN38512_c0_g1/TRINI	4

							TY_DN8297_c0_g1/TRINITY_DN38306_c0_g1	
GO:0032536	regulation of cell projection size	4/2626	11/33142	0.008246	0.043755	0.032761	TRINITY_DN8323_c0_g1/TRINITY_DN3446_c0_g1/TRINITY_DN13394_c0_g1/TRINITY_DN8012_c0_g1	4
GO:0033700	phospholipid efflux	4/2626	11/33142	0.008246	0.043755	0.032761	TRINITY_DN7048_c0_g1/TRINITY_DN10757_c0_g1/TRINITY_DN7045_c1_g1/TRINITY_DN38748_c0_g1	4
GO:0036019	endolysosome	4/2626	11/33142	0.008246	0.043755	0.032761	TRINITY_DN8323_c0_g1/TRINITY_DN3446_c0_g1/TRINITY_DN13394_c0_g1/TRINITY_DN8012_c0_g1	4
GO:0043083	synaptic cleft	4/2626	11/33142	0.008246	0.043755	0.032761	TRINITY_DN2738_c0_g1/TRINITY_DN740_c0_g1/TRINITY_DN2598_c0_g2/TRINITY_DN2281_c0_g1	4
GO:0048542	lymph gland development	4/2626	11/33142	0.008246	0.043755	0.032761	TRINITY_DN3284_c0_g1/TRINITY_DN19401_c0_g1/TRINITY_DN339_c0_g2/TRINITY_DN14168_c0_g2	4
GO:0050062	long-chain-fatty-acyl-CoA reductase activity	4/2626	11/33142	0.008246	0.043755	0.032761	TRINITY_DN11631_c0_g3/TRINITY_DN3305_c0_g1/TRINITY_DN6810_c0_g1/TRINITY_DN18007_c0_g1	4
GO:0051898	negative regulation of protein kinase B signaling	4/2626	11/33142	0.008246	0.043755	0.032761	TRINITY_DN93_c0_g1/TRINITY_DN22905_c0_g1/TRINITY_DN56840_c0_g1/TRINITY_DN7084_c0_g1	4
GO:0060179	male mating behavior	4/2626	11/33142	0.008246	0.043755	0.032761	TRINITY_DN2677_c0_g1/TRINITY_DN9643_c0_g1/TRINIT	4

							Y_DN5339_c0_g1/TRINITY_DN12402_c0_g1	
GO:0060395	SMAD protein signal transduction	4/2626	11/33142	0.008246	0.043755	0.032761	TRINITY_DN1695_c0_g1/TRINITY_DN9767_c0_g3/TRINITY_DN37845_c0_g1/TRINITY_DN7084_c0_g1	4
GO:0070840	dynein complex binding	4/2626	11/33142	0.008246	0.043755	0.032761	TRINITY_DN16186_c0_g1/TRINITY_DN233_c0_g1/TRINITY_DN1633_c0_g1/TRINITY_DN5914_c0_g1	4
GO:0003941	L-serine ammonia-lyase activity	5/2626	17/33142	0.00858	0.044936	0.033645	TRINITY_DN10554_c0_g2/TRINITY_DN10867_c0_g1/TRINITY_DN291_c1_g1/TRINITY_DN3638_c0_g1/TRINITY_DN17288_c0_g1	5
GO:0007041	lysosomal transport	5/2626	17/33142	0.00858	0.044936	0.033645	TRINITY_DN72971_c0_g1/TRINITY_DN5278_c0_g1/TRINITY_DN4826_c1_g1/TRINITY_DN3354_c0_g1/TRINITY_DN43641_c0_g1	5
GO:0007472	wing disc morphogenesis	5/2626	17/33142	0.00858	0.044936	0.033645	TRINITY_DN39639_c3_g1/TRINITY_DN7582_c0_g1/TRINITY_DN9642_c1_g2/TRINITY_DN864_c0_g3/TRINITY_DN17100_c0_g1	5
GO:0008559	xenobiotic transmembrane transporting ATPase activity	5/2626	17/33142	0.00858	0.044936	0.033645	TRINITY_DN4514_c0_g1/TRINITY_DN14877_c1_g1/TRINITY_DN1216_c2_g1/TRINITY_DN23353_c2_g1/TRINITY_DN5370_c0_g1	5
GO:0017105	acyl-CoA delta11-desaturase activity	5/2626	17/33142	0.00858	0.044936	0.033645	TRINITY_DN28084_c0_g1/TRINITY_DN6435_c0_g3/TRINIT	5

insects MDPI

							Y_DN9779_c0_g1/TRINITY_DN5693_c0_g2/TRINITY_DN12549_c0_g1	
GO:0032367	intracellular cholesterol transport	5/2626	17/33142	0.00858	0.044936	0.033645	TRINITY_DN6638_c3_g1/TRINITY_DN7048_c0_g1/TRINITY_DN5278_c0_g1/TRINITY_DN10757_c0_g1/TRINITY_DN38748_c0_g1	5
GO:0071345	cellular response to cytokine stimulus	5/2626	17/33142	0.00858	0.044936	0.033645	TRINITY_DN10760_c0_g1/TRINITY_DN4326_c0_g1/TRINITY_DN39318_c0_g1/TRINITY_DN6219_c0_g1/TRINITY_DN52030_c0_g1	5
GO:0008049	male courtship behavior	8/2626	38/33142	0.008757	0.045779	0.034277	TRINITY_DN9170_c1_g1/TRINITY_DN7953_c1_g1/TRINITY_DN7807_c0_g1/TRINITY_DN419_c0_g2/TRINITY_DN11596_c0_g1/TRINITY_DN16530_c0_g1/TRINITY_DN6409_c3_g1/TRINITY_DN27661_c0_g1	8
GO:0005811	lipid droplet	9/2626	46/33142	0.00914	0.047694	0.03571	TRINITY_DN2516_c0_g1/TRINITY_DN16186_c0_g1/TRINITY_DN2477_c0_g1/TRINITY_DN16937_c0_g2/TRINITY_DN11204_c0_g1/TRINITY_DN226_c0_g3/TRINITY_DN5675_c0_g1/TRINITY_DN33814_c0_g1/TRINITY_DN13766_c0_g1	9
GO:0043066	negative regulation of apoptotic process	23/2626	172/33142	0.009388	0.048721	0.036479	TRINITY_DN658_c0_g1/TRINITY_DN1487_c0_g2/TRINITY_DN1557_c0_g1/TRINITY_DN5239_c1_g2/TRINITY_DN2476_	23

							c1_g1/TRINITY_DN3732_c0_g1/TRINITY_DN5483_c0_g1/TRINITY_DN10783_c0_g1/TRINITY_DN4441_c3_g1/TRINITY_DN15112_c0_g1/TRINITY_DN1797_c0_g1/TRINITY_DN6136_c0_g1/TRINITY_DN12891_c0_g1/TRINITY_DN271_c0_g1/TRINITY_DN10569_c0_g2/TRINITY_DN93_c0_g1/TRINITY_DN29974_c0_g1/TRINITY_DN40503_c0_g1/TRINITY_DN3238_c0_g1/TRINITY_DN7903_c0_g1/TRINITY_DN4137_c2_g1/TRINITY_DN6505_c0_g1/TRINITY_DN689_c0_g1	
GO:0007050	cell cycle arrest	7/2626	31/33142	0.009406	0.048721	0.036479	TRINITY_DN43642_c0_g1/TRINITY_DN1344_c0_g1/TRINITY_DN4329_c1_g1/TRINITY_DN1178_c2_g1/TRINITY_DN19338_c0_g1/TRINITY_DN11156_c0_g5/TRINITY_DN4000_c0_g1	7
GO:0030148	sphingolipid biosynthetic process	7/2626	31/33142	0.009406	0.048721	0.036479	TRINITY_DN5032_c1_g1/TRINITY_DN1854_c0_g2/TRINITY_DN7107_c0_g1/TRINITY_DN12176_c0_g1/TRINITY_DN34926_c1_g1/TRINITY_DN13730_c0_g1/TRINITY_DN12338_c0_g1	7
GO:1990830	cellular response to leukemia inhibitory factor	7/2626	31/33142	0.009406	0.048721	0.036479	TRINITY_DN11750_c0_g1/TRINITY_DN11100_c0_g1/TRINI	7

insects MDPI

							TY_DN2651_c0_g1/TRINITY_DN1440_c0_g2/TRINITY_DN20844_c0_g1/TRINITY_DN922_c0_g1/TRINITY_DN40503_c0_g1	
GO:0007229	integrin-mediated signaling pathway	6/2626	24/33142	0.009536	0.049214	0.036849	TRINITY_DN9277_c0_g1/TRINITY_DN4223_c1_g1/TRINITY_DN6886_c3_g1/TRINITY_DN2331_c0_g1/TRINITY_DN2281_c0_g1/TRINITY_DN13862_c0_g2	6
GO:0016849	phosphorus-oxygen lyase activity	6/2626	24/33142	0.009536	0.049214	0.036849	TRINITY_DN20653_c0_g1/TRINITY_DN13614_c1_g1/TRINITY_DN3665_c0_g1/TRINITY_DN5234_c0_g1/TRINITY_DN6839_c2_g1/TRINITY_DN26727_c0_g1	6

insects MDPI

Table S5. Enriched GO terms in both groups.

GO Terms	Discription	*p* Value (C-vs-L)	*p* Value (FE-vs-FL)
**MF(molecular function)**			
GO:0042302	structural constituent of cuticle	8.55828×10^{-7}	3.39188×10^{-29}
GO:0004252	serine-type endopeptidase activity	5.34017×10^{-16}	2.49093×10^{-17}
GO:0008061	chitin binding	0.000550851	1.49938×10^{-8}
GO:0004806	triglyceride lipase activity	0.000792967	1.95232×10^{-8}
GO:0015574	trehalose transmembrane transporter activity	0.000380213	1.15591×10^{-6}
GO:0003777	microtubule motor activity	0.000241447	1.8088×10^{-6}
GO:0005230	extracellular ligand-gated ion channel activity	2.22376×10^{-5}	1.90692×10^{-6}
GO:0070330	aromatase activity	0.000142353	0.000237917
GO:0030246	carbohydrate binding	0.000192478	0.000311039
GO:0102336	3-oxo-arachidoyl-CoA synthase activity	7.2823×10^{-5}	0.000655346
GO:0102337	3-oxo-cerotoyl-CoA synthase activity	7.2823×10^{-5}	0.000655346
GO:0102338	3-oxo-lignoceronyl-CoA synthase activity	7.2823×10^{-5}	0.000655346
GO:0102756	very-long-chain 3-ketoacyl-CoA synthase activity	7.2823×10^{-5}	0.000655346
GO:0005549	odorant binding	1.07119×10^{-12}	0.00074255
GO:0016616	oxidoreductase activity, acting on the CH-OH group of donors, NAD or NADP as acceptor	1.30622×10^{-5}	0.000848985
GO:0080019	fatty-acyl-CoA reductase (alcohol-forming) activity	2.0474×10^{-6}	0.001130314
GO:0016705	oxidoreductase activity, acting on paired donors, with incorporation or reduction of molecular oxygen	1.34491×10^{-6}	0.001428051
GO:0050062	long-chain-fatty-acyl-CoA reductase activity	0.00052776	0.008245781
BP (biological process)			
GO:0060285	cilium-dependent cell motility	1.58×10^{-7}	9.13×10^{-12}
GO:0003351	epithelial cilium movement	6.52×10^{-9}	2.77×10^{-11}
GO:0036159	inner dynein arm assembly	7.39×10^{-8}	2.20×10^{-9}
GO:0006811	ion transport	1.19×10^{-6}	3.65×10^{-9}
GO:0042073	intraciliary transport	9.84×10^{-8}	4.12×10^{-9}
GO:0006030	chitin metabolic process	1.61×10^{-5}	4.27×10^{-9}
GO:0006629	lipid metabolic process	9.42×10^{-5}	5.43×10^{-8}
GO:0060294	cilium movement involved in cell motility	3.39×10^{-7}	1.68×10^{-7}
GO:0015771	trehalose transport	0.000586373	6.67×10^{-7}
GO:0007286	spermatid development	0.000499646	8.93×10^{-7}
GO:0006583	melanin biosynthetic process from tyrosine	4.38×10^{-5}	1.50×10^{-6}
GO:0008340	determination of adult lifespan	0.000306786	2.21×10^{-6}
GO:0008611	ether lipid biosynthetic process	0.000428428	3.22×10^{-6}
GO:0007288	sperm axoneme assembly	2.82×10^{-9}	5.39×10^{-6}
GO:0016042	lipid catabolic process	0.000169164	6.28×10^{-6}
GO:0018095	protein polyglutamylation	0.000151719	2.18×10^{-5}
GO:0007224	smoothened signaling pathway	2.10×10^{-5}	0.000202125
GO:0007586	digestion	1.56×10^{-5}	0.000455913
GO:0035002	liquid clearance, open tracheal system	0.001133494	0.000867076
GO:0036158	outer dynein arm assembly	0.00129575	0.001453549
GO:0035336	long-chain fatty-acyl-CoA metabolic process	2.05×10^{-6}	0.001845944
GO:0042811	pheromone biosynthetic process	0.00052776	0.002706005
GO:0006565	L-serine catabolic process	0.000818339	0.003609235
GO:0010025	wax biosynthetic process	3.09×10^{-6}	0.00741777
CC (cellular component)			
GO:0031514	motile cilium	7.02×10^{-17}	3.51×10^{-28}
GO:0005929	cilium	6.92×10^{-6}	5.67×10^{-27}

GO:0005930	axoneme	4.75×10^{-8}	1.16×10^{-15}
GO:0030992	intraciliary transport particle B	1.63×10^{-7}	3.83×10^{-14}
GO:0036126	sperm flagellum	5.26×10^{-12}	2.60×10^{-12}
GO:0031090	organelle membrane	9.55×10^{-7}	1.03×10^{-9}
GO:0005764	lysosome	0.000376332	5.50×10^{-9}
GO:0030286	dynein complex	0.000322986	6.95×10^{-9}
GO:0005858	axonemal dynein complex	2.57×10^{-6}	9.87×10^{-9}
GO:0036156	inner dynein arm	1.06×10^{-8}	1.88×10^{-6}
GO:0001669	acrosomal vesicle	0.001270588	8.13×10^{-5}
GO:0097546	ciliary base	0.00085926	0.000982076

insects

Table S6. Candidate juvenile hormone (JH) and moulting hormone (ecdysone) expression analysis of C-vs-L group.

Row.names	baseMean	log2FoldChange	lfcSE	stat	pvalue	padj	C1	C2	C3	L1	L2	L3
Dib	69.07495	−2.80984	0.430371	−6.52888	6.63×10^{-11}	1.46×10^{-9}	9.201501	23.34062	20.05101	126.69	106.7711	128.3955
FDP	67.25664	−3.57481	0.563691	−6.3418	2.27×10^{-10}	2.50×10^{-9}	14.4595	12.56802	4.296645	48.33261	146.8102	177.0729
MevPPD	27678.14	1.402388	0.26762	5.240227	1.60×10^{-7}	1.18×10^{-6}	37847.09	49200.23	33440.79	13749.9	12265.32	19565.49
FPPP	362.5937	2.373825	0.79085	3.001611	0.002686	0.014771	634.9036	700.2185	488.3853	257.7739	37.13776	57.14304
JHDK	669.5392	2.86628	1.053584	2.720503	0.006518	0.02868	1561.626	64.63556	1906.278	382.267	25.53221	76.89618
HMGR	145.9369	−1.4261	0.564404	−2.52673	0.011513	0.042214	56.52351	66.43099	114.5772	91.53904	363.834	182.7166
JHE	3.307633	5.752463	2.386864	2.41005	0.01595	0.05013	14.4595	5.386296	0	0	0	0
FPPS	2292.896	−1.29303	0.578638	−2.23461	0.025443	0.069967	1873.163	833.0805	1280.4	2072.444	3801.978	3896.308
MevK	1094.432	1.397501	0.733558	1.905099	0.056767	0.113534	667.7661	3429.275	663.1155	622.4655	568.0916	615.8749
JHAMT	249.2475	−1.66986	0.865514	−1.92933	0.05369	0.113534	214.2635	48.47667	94.52619	50.52955	880.2809	207.4081
phm	285.2763	1.161711	0.589188	1.971715	0.048642	0.113534	274.7305	482.9712	425.3679	217.4968	189.7507	121.3408
CYP18A1	881.5521	1.120999	0.772861	1.450453	0.146932	0.269376	1318.444	1382.483	922.3465	1200.992	329.5976	135.4502
MevPK	40.74006	−0.53775	0.387036	−1.38941	0.164708	0.278736	23.661	39.49951	37.23759	39.54487	64.99107	39.5063
SAD	69.33357	0.574397	0.480183	1.196203	0.231617	0.34206	44.69301	150.8163	54.42417	43.20643	81.23884	41.6227
SHD	528.4596	−1.05432	0.88442	−1.1921	0.233222	0.34206	89.38601	141.8391	799.176	363.2269	578.5366	1198.593
AACT	2532.337	0.592295	0.619279	0.956427	0.338856	0.414158	3315.169	2231.722	3587.699	3623.481	961.5197	1474.431
IPPI	908.1342	0.568516	0.578912	0.982042	0.326079	0.414158	921.4646	1515.345	817.7948	711.0753	672.5416	810.5845
spook	393.9459	−0.8606	0.846078	−1.01717	0.309075	0.414158	39.435	423.722	376.6725	246.0569	676.0232	601.7655
Nvd	237.1985	0.719022	0.797314	0.901806	0.36716	0.425132	160.369	633.7875	91.66176	44.67105	247.1982	245.5034
FDH	3122.018	−0.14089	0.57313	−0.24583	0.805817	0.886399	3893.549	1504.572	3510.359	4001.355	1870.234	3952.041
HMGS	305.9354	−0.06489	0.58732	−0.11049	0.912025	0.950645	210.32	380.6316	306.494	270.9556	308.1273	359.084
Epoxidase	3.894594	−0.0866	1.399122	−0.0619	0.950645	0.950645	6.572501	1.795432	2.86443	5.126186	3.481665	3.527348
JHEH	2216.754	1.116502	1.033617	1.080189	NA	NA	4193.255	852.8303	4056.033	3950.825	101.5486	146.0322

insects

Table S7. Candidate juvenile hormone (JH) and moulting hormone (ecdysone) expression analysis of E-vs-F group.

Row.names	baseMean	log2FoldChange	lfcSE	stat	pvalue	padj	E1	E2	E3	F1	F2	F3
FDP	119.6213	5.075544	0.931899	5.446454	5.14×10^{-8}	1.18×10^{-6}	5.556407	12.40516	2.630001	138.4645	462.0522	96.61978
HMGS	449.3272	−1.97046	0.376024	−5.24024	1.60×10^{-7}	1.84×10^{-6}	740.8543	638.4523	767.9604	200.8495	191.2869	156.5598
CYP18A1	4067.003	−4.92171	1.043565	−4.71625	2.40×10^{-6}	1.84×10^{-5}	5546.22	17214.23	861.7638	381.9184	292.3187	105.5661
JHDK	114.6794	3.68802	1.231562	2.994587	0.002748	0.012737	9.260678	0.827011	39.45002	260.1914	330.0373	48.30989
Dib	458.558	3.152753	1.053624	2.992294	0.002769	0.012737	15.74315	99.24129	163.0601	1337.475	142.7916	993.0366
MevK	1924.799	−2.17903	0.83428	−2.61186	0.009005	0.034519	3388.482	5342.489	728.5104	701.4518	1013.012	374.849
SAD	181.1887	−1.24983	0.590351	−2.11709	0.034252	0.112542	151.8751	366.3657	247.2201	123.2486	68.70164	129.721
MevPK	188.5536	−1.28228	0.678881	−1.88882	0.058916	0.169385	282.4507	116.6085	402.3902	98.90318	157.6096	73.35946
phm	102.6528	−1.03913	0.649937	−1.59881	0.109862	0.280758	94.45892	189.3855	130.6234	83.68731	30.98309	86.77888
JHAMT	4.358029	−3.30588	2.154608	−1.53433	0.124949	0.287382	17.59529	0	6.13667	1.521587	0	0.894628
HMGR	828.3447	−0.89039	0.605893	−1.46955	0.141684	0.296248	1132.581	1587.861	507.5902	674.0632	646.6036	421.3696
FDH	2683.016	0.664594	0.538831	1.2334	0.217427	0.416734	1988.268	1238.862	2999.955	3213.593	4108.627	2548.794
IPPI	1015.949	0.340298	0.35267	0.964917	0.334586	0.533151	917.7332	905.5767	867.0238	1150.32	971.2525	1283.791
FPPS	3947.969	0.447559	0.47661	0.939046	0.347707	0.533151	3248.646	3097.155	3674.988	6156.343	4638.034	2872.649
SHD	97.308	0.560599	0.580805	0.965209	0.33444	0.533151	87.97644	95.10623	52.60003	101.9464	168.3864	77.8326
AACT	2336.706	−0.21883	0.438328	−0.49924	0.617613	0.692154	2580.025	2889.575	2070.688	2128.701	2750.76	1600.489
MevPPD	14316.34	0.342529	0.489529	0.699711	0.484108	0.692154	10611.81	13348.78	13913.58	21452.86	9856.664	16714.33
FPPP	135.4362	0.419572	0.736565	0.569634	0.568926	0.692154	165.7661	35.56146	146.4034	115.6406	206.1049	143.1404
Epoxidase	19.14208	−.48478	1.012148	−0.47896	0.631967	0.692154	20.37349	6.616086	40.32669	12.1727	6.735455	28.62808
JHE	483.2224	0.510775	0.982535	0.519854	0.603165	0.692154	111.1281	760.0228	324.3668	1052.938	126.6265	524.2518
spook	304.4963	−1.0462	1.89308	−0.55264	0.580508	0.692154	12.03888	1177.663	41.20335	339.314	0	256.7581
JHEH	333.8166	−0.21356	0.662186	−0.32251	0.747065	0.781022	337.0887	205.9257	532.1369	272.3641	486.2998	169.0846
Nvd	47.44215	−0.22423	1.556433	−0.14407	0.885447	0.885447	0.926068	149.6889	2.630001	50.21238	60.61909	20.57643

insects

MDPI
